# Supplementary material for: Identification of Candidate Lung Function-Related Plasma Proteins to Pinpoint Drug Targets for Common Pulmonary Diseases: A Comprehensive Multi-Omics Integration Analysis
Source: Curr Issues Mol Biol. 2025 Mar 1;47(3):167. doi: 10.3390/cimb47030167 (PMC11941423; doi:10.3390/cimb47030167)
Supplement: Supplementary file 1 [file cimb-47-00167-s001.zip › cimb-3428330-supplementary.pdf]

Supplementary Materials

Figure S1: The LocusZoom plot for FEV1 and ASPN

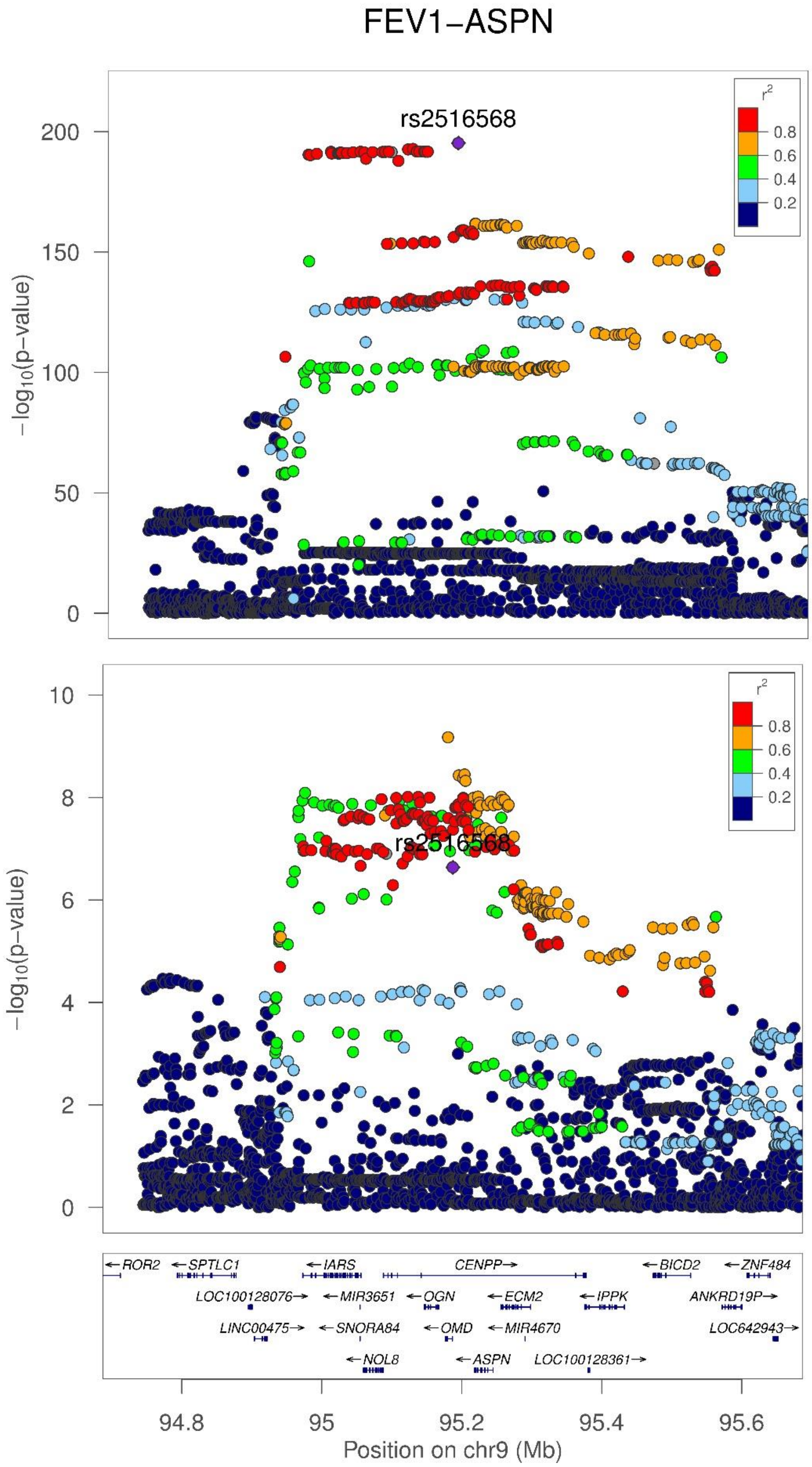

Figure S2: The LocusZoom plot for FEV1 and EFEMP1

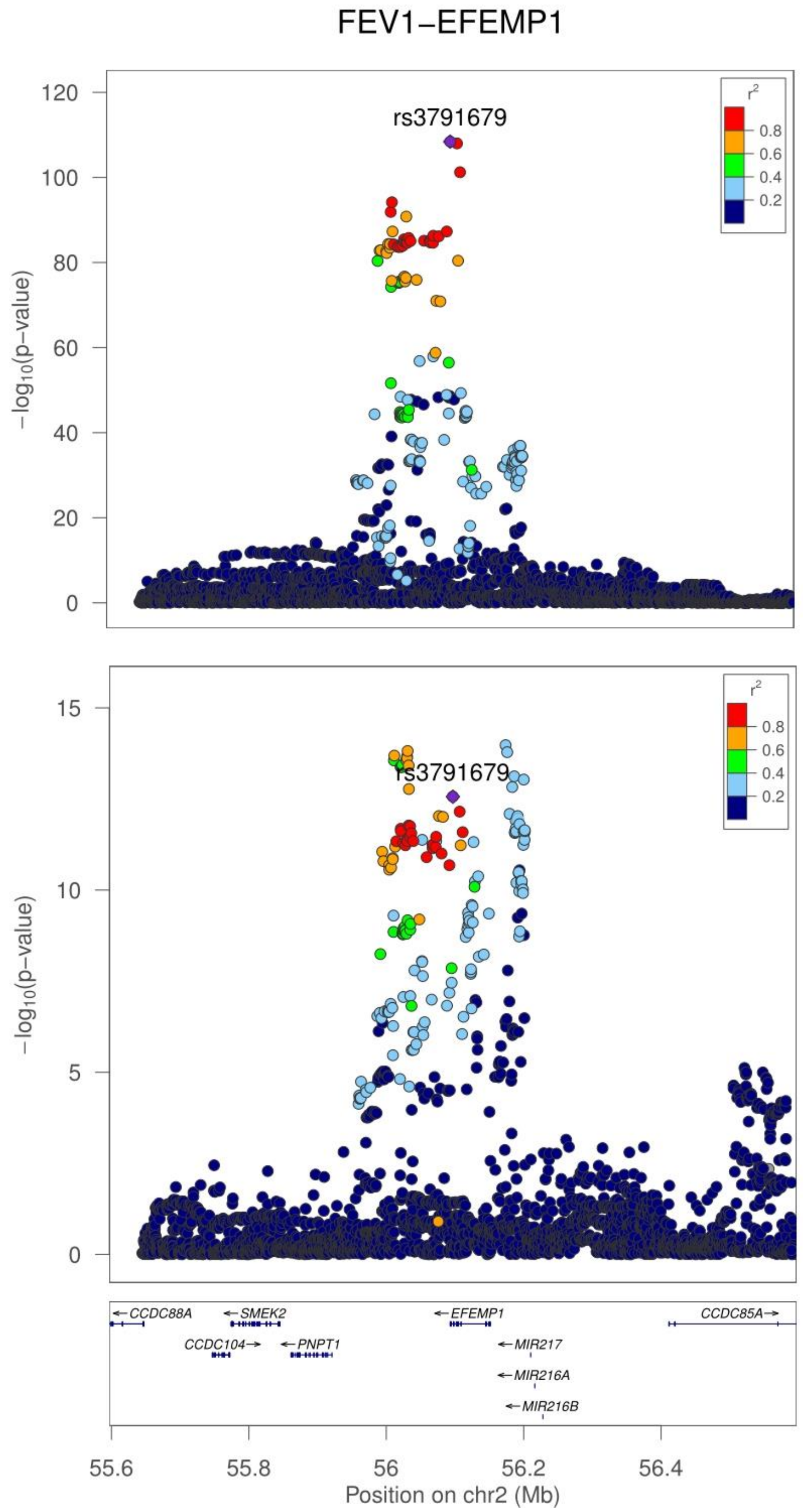

Figure S3: The LocusZoom plot for FEV1 and FKBP4

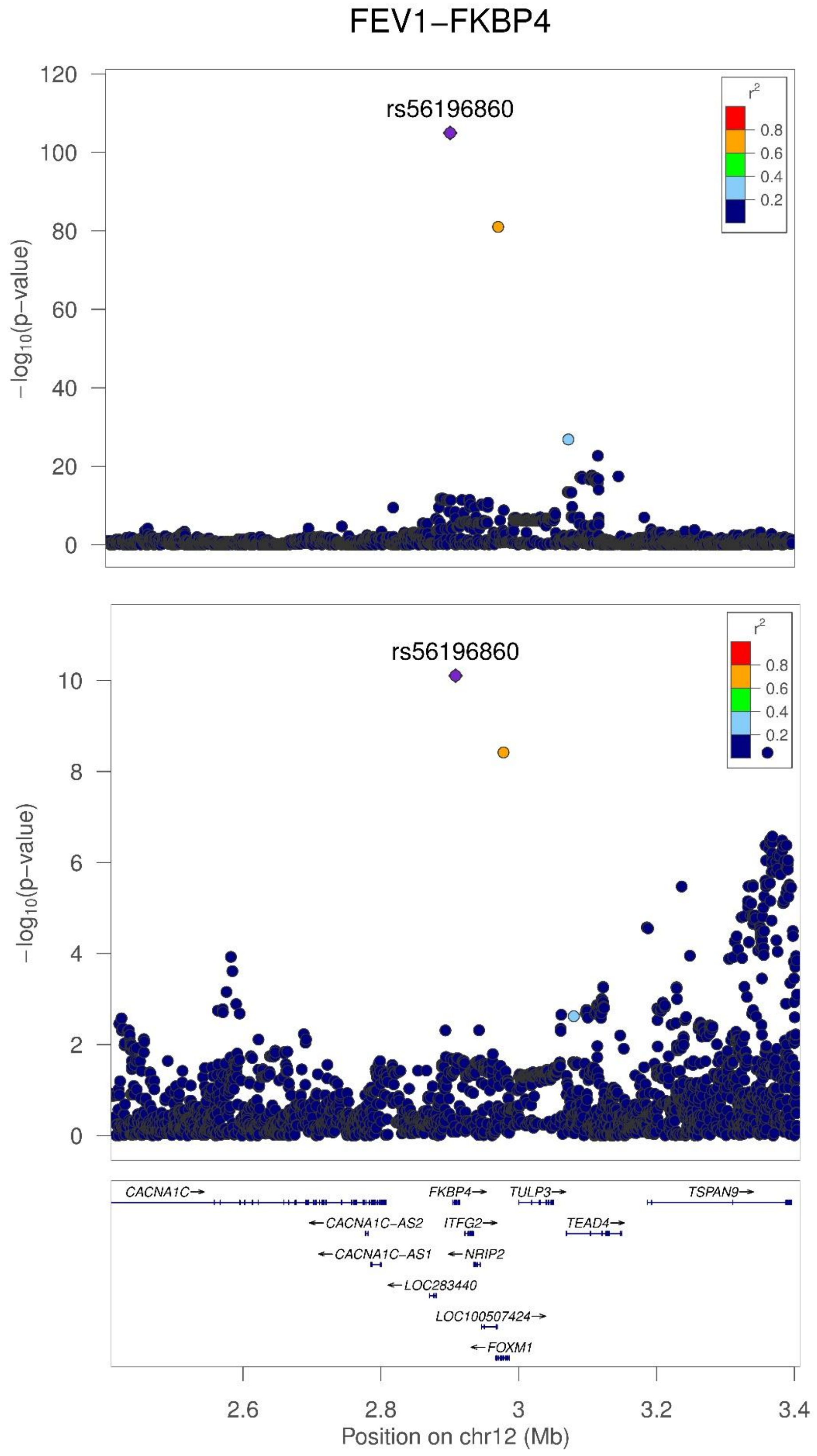

Figure S4: The LocusZoom plot for FEV1 and FN1

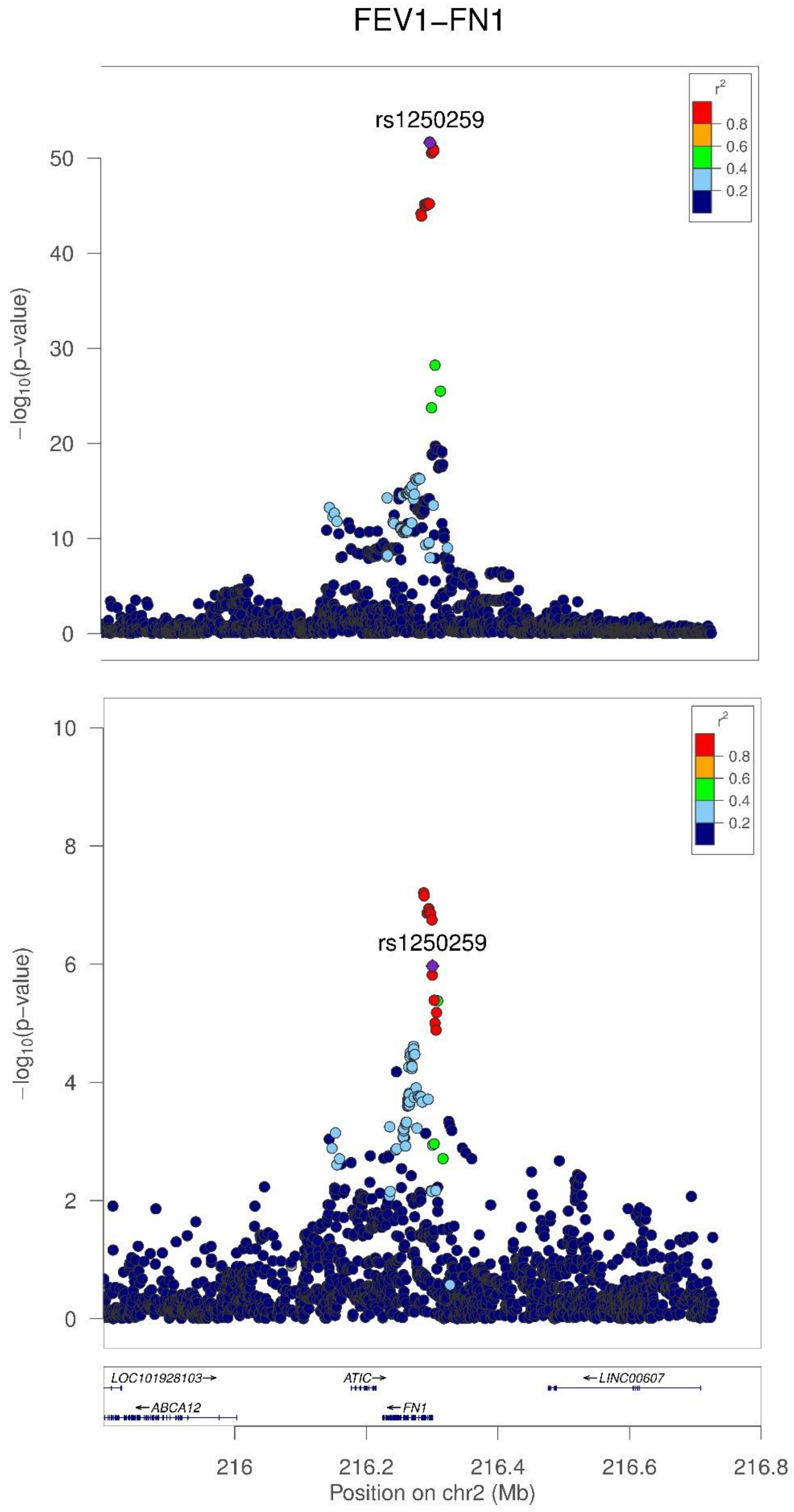

Figure S5: The LocusZoom plot for FEV1 and GM2A

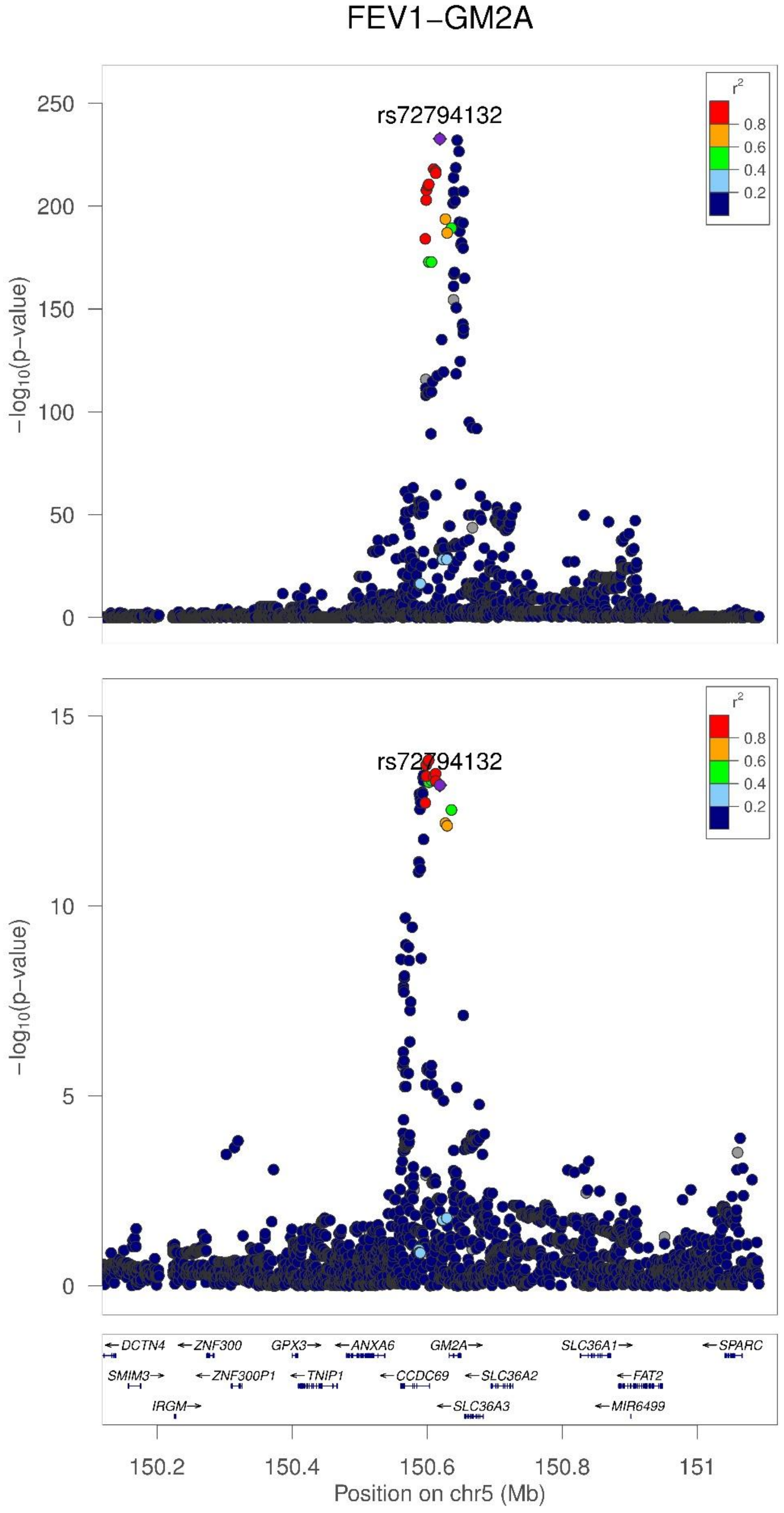

Figure S6: The LocusZoom plot for FEV1 and GMPR2

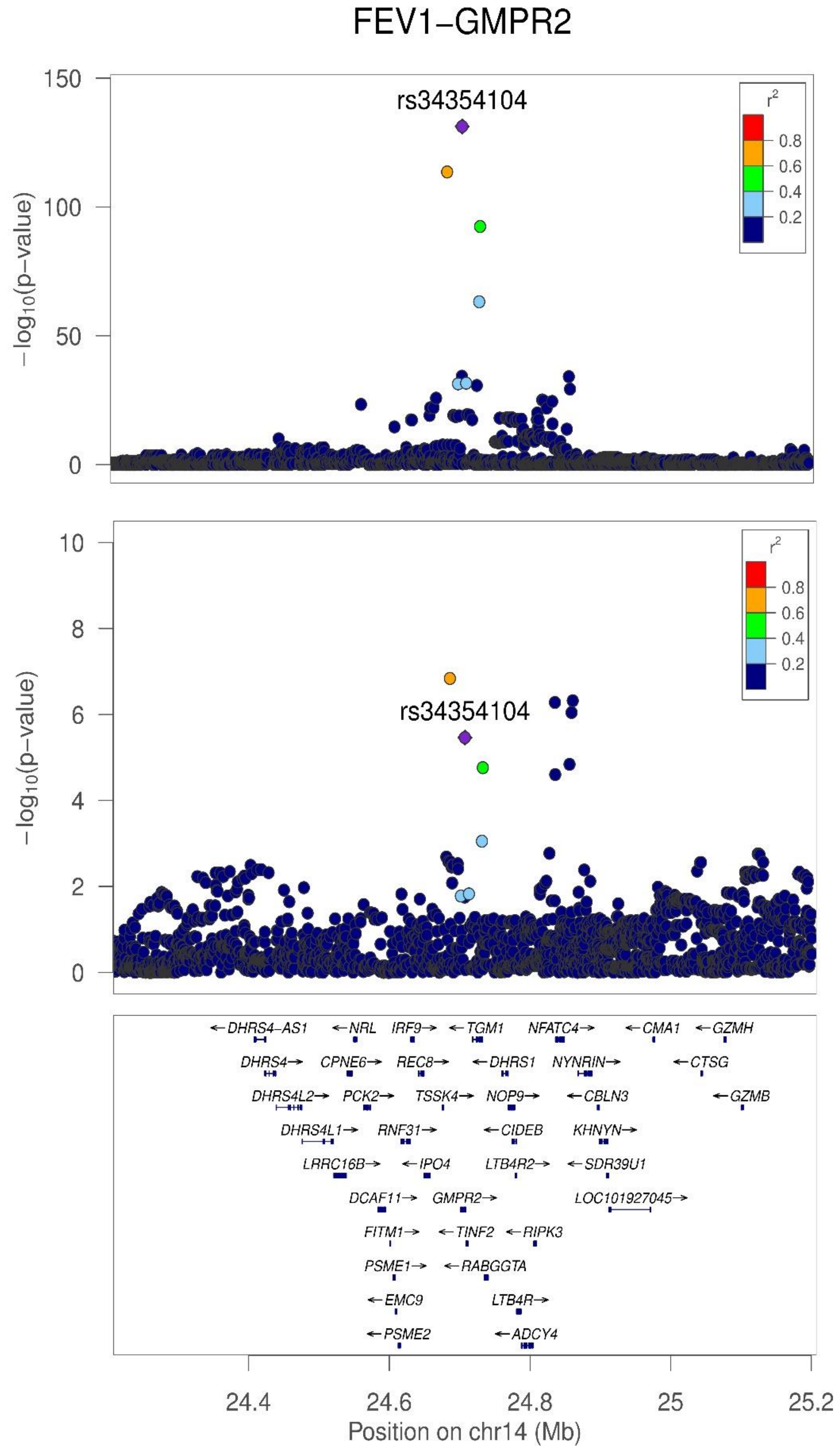

Figure S7: The LocusZoom plot for FEV1 and KYNU

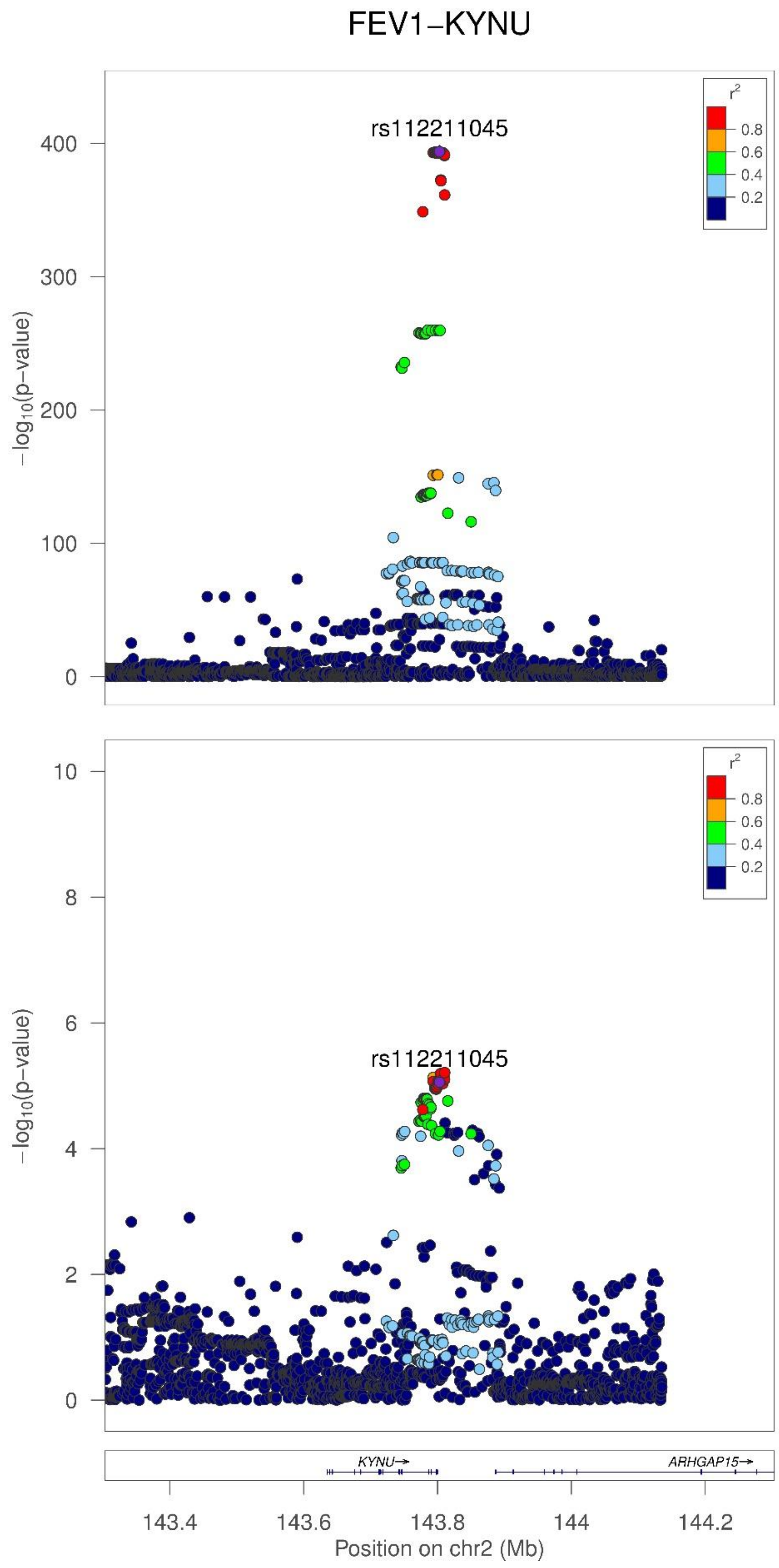

Figure S8: The LocusZoom plot for FEV1 and OGN

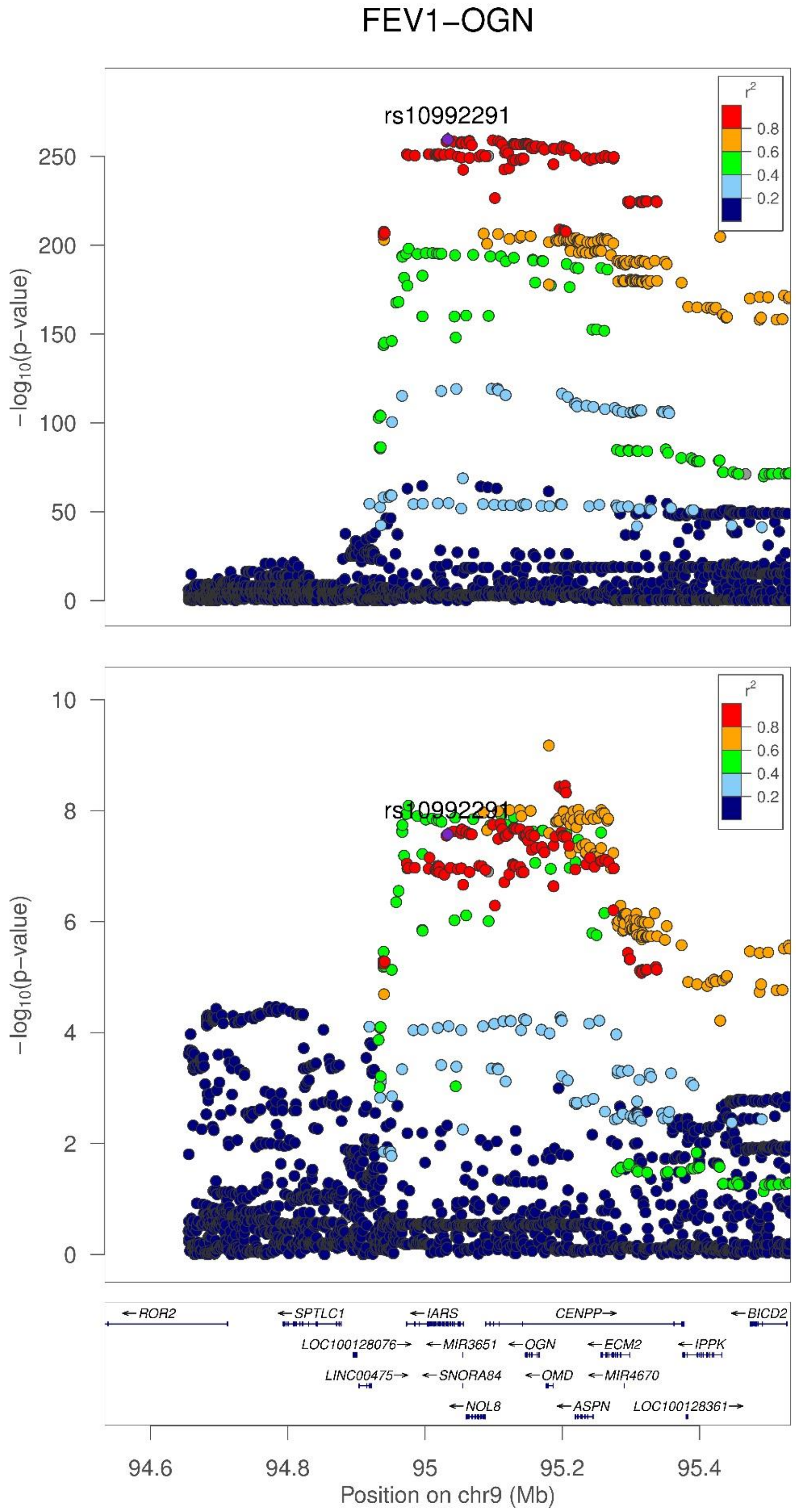

Figure S9: The LocusZoom plot for FEV1 and SCARF2

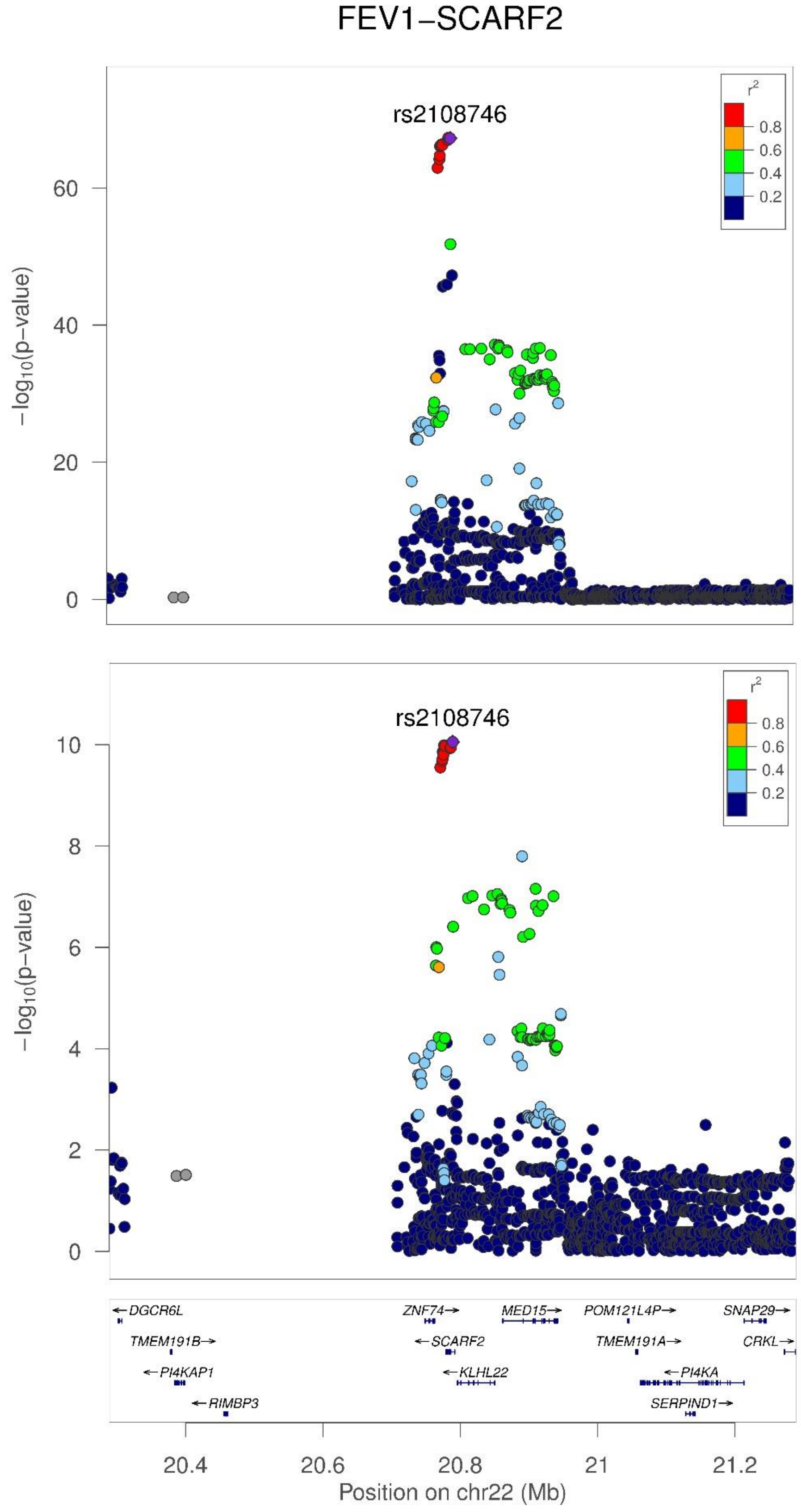

Figure S10: The LocusZoom plot for FEV1 and SELM

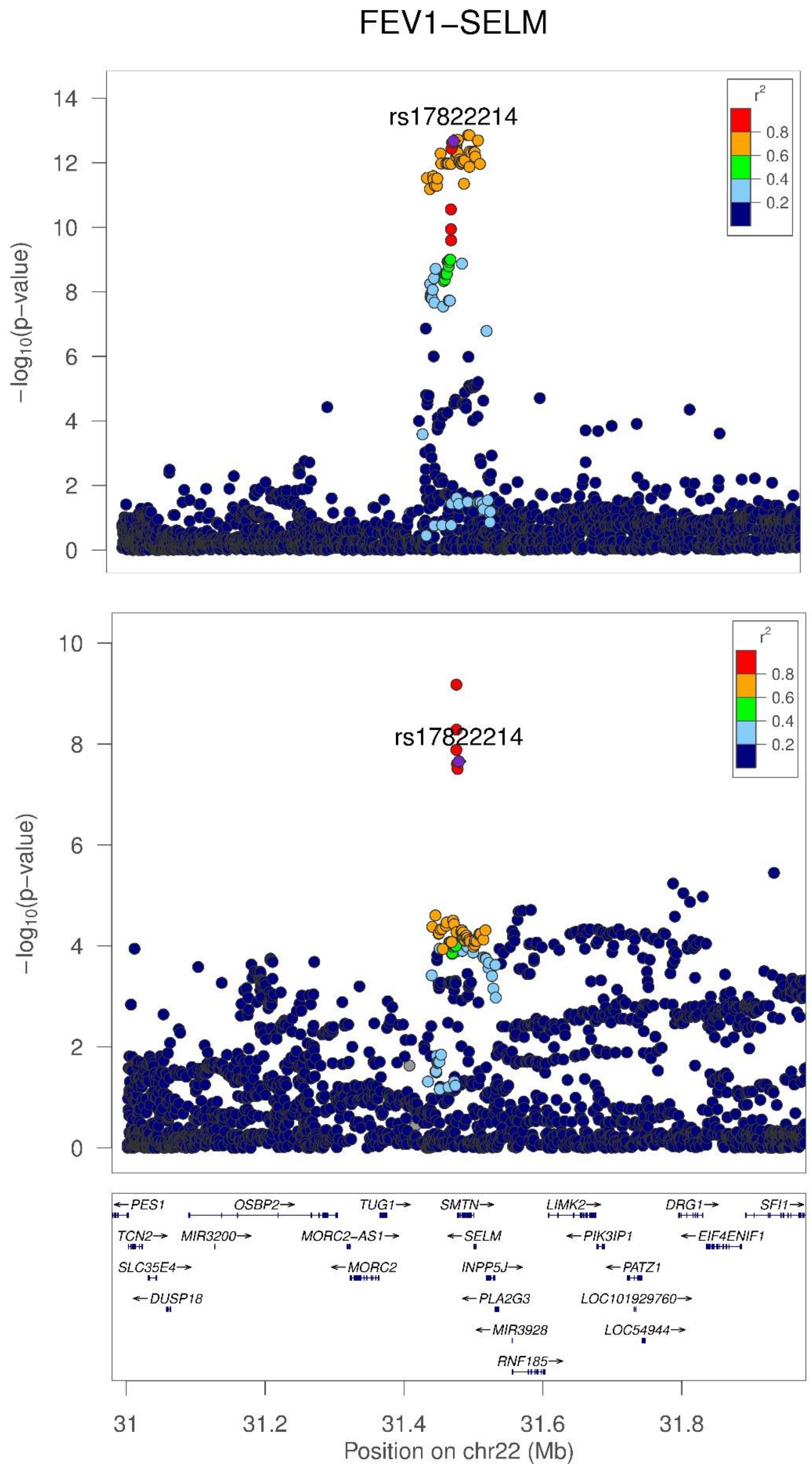

Figure S11: The LocusZoom plot for FEV1 and SFRP1

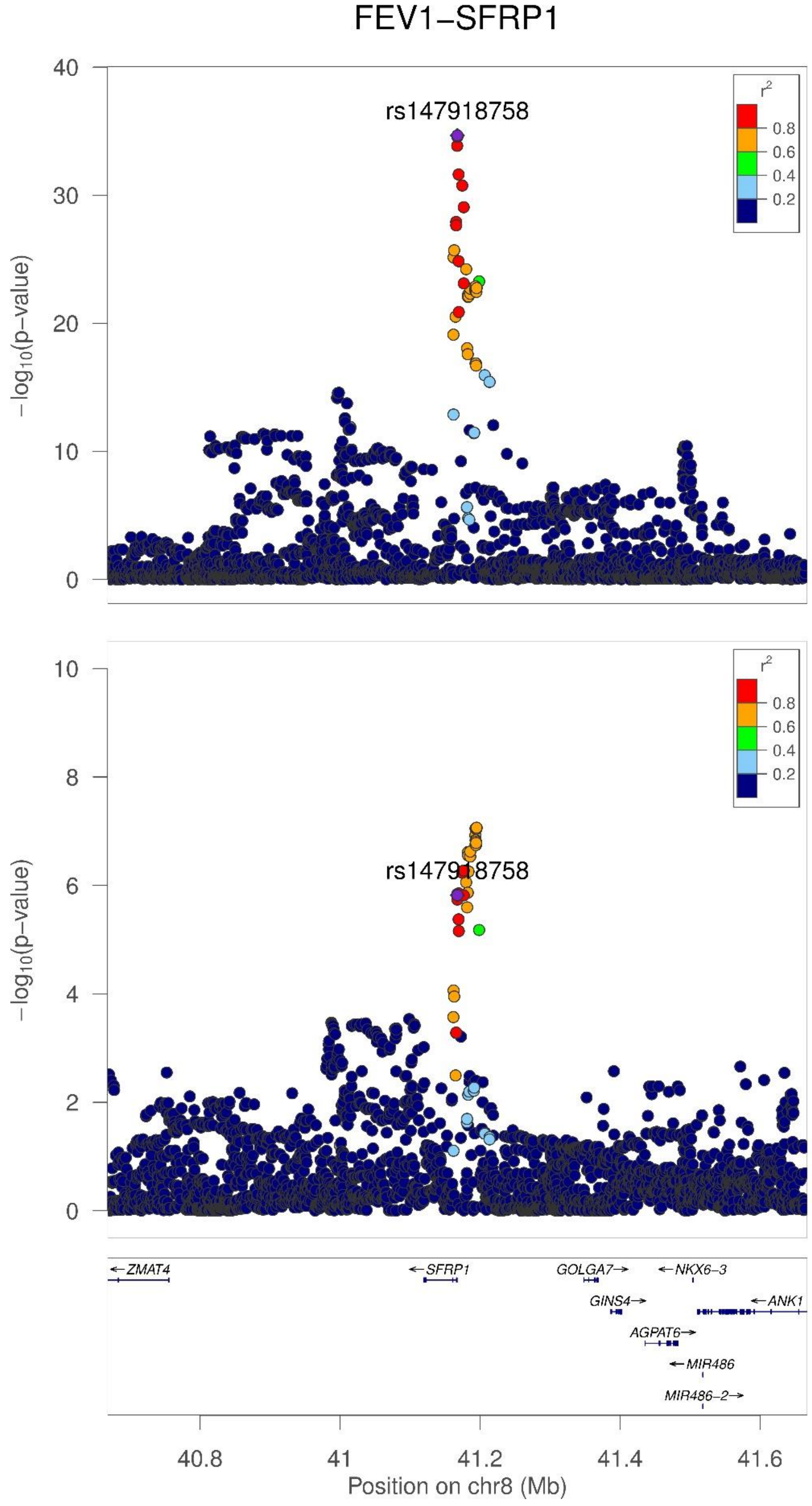

Figure S12: The LocusZoom plot for FEV1 and TESC

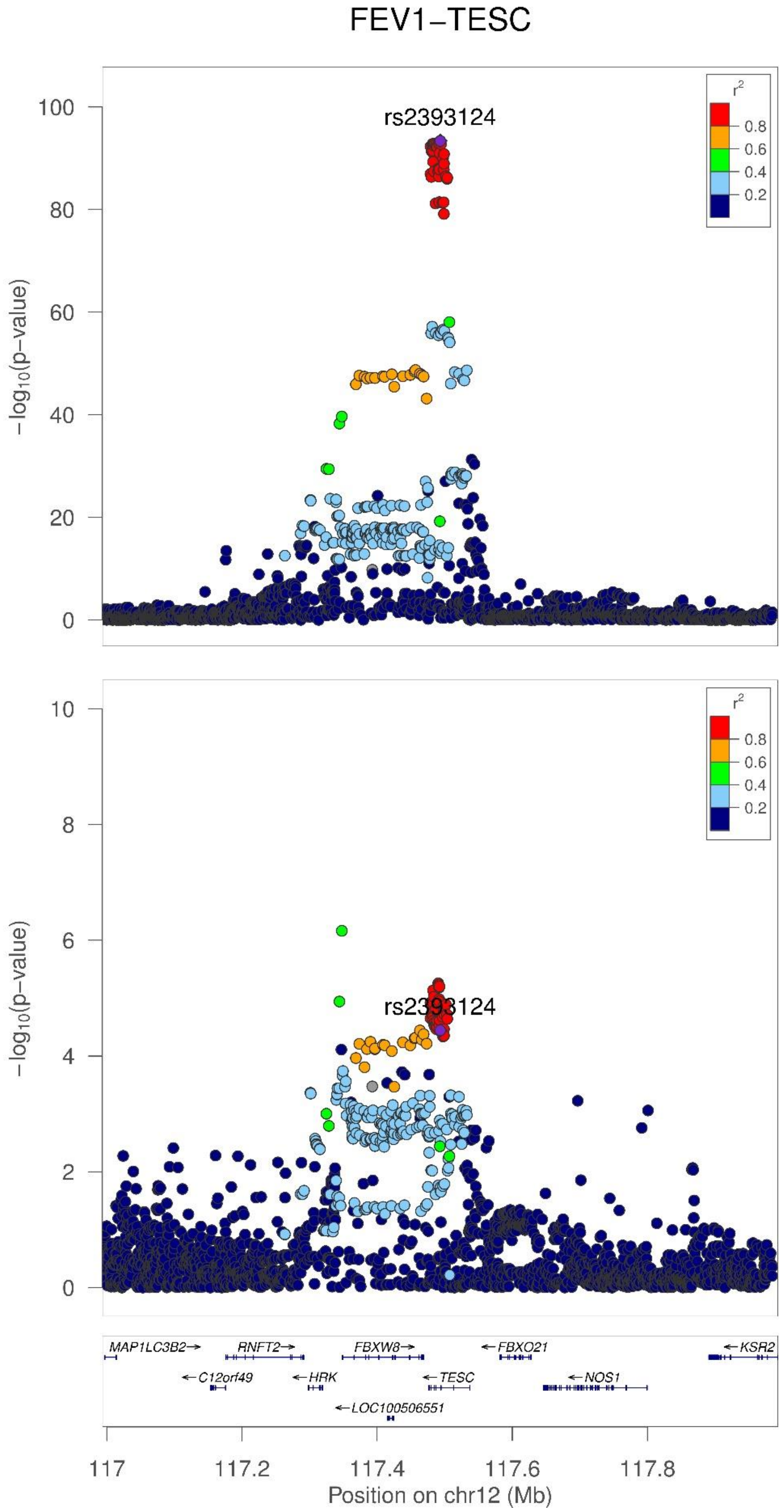

Figure S13: The LocusZoom plot for FVC and CA3

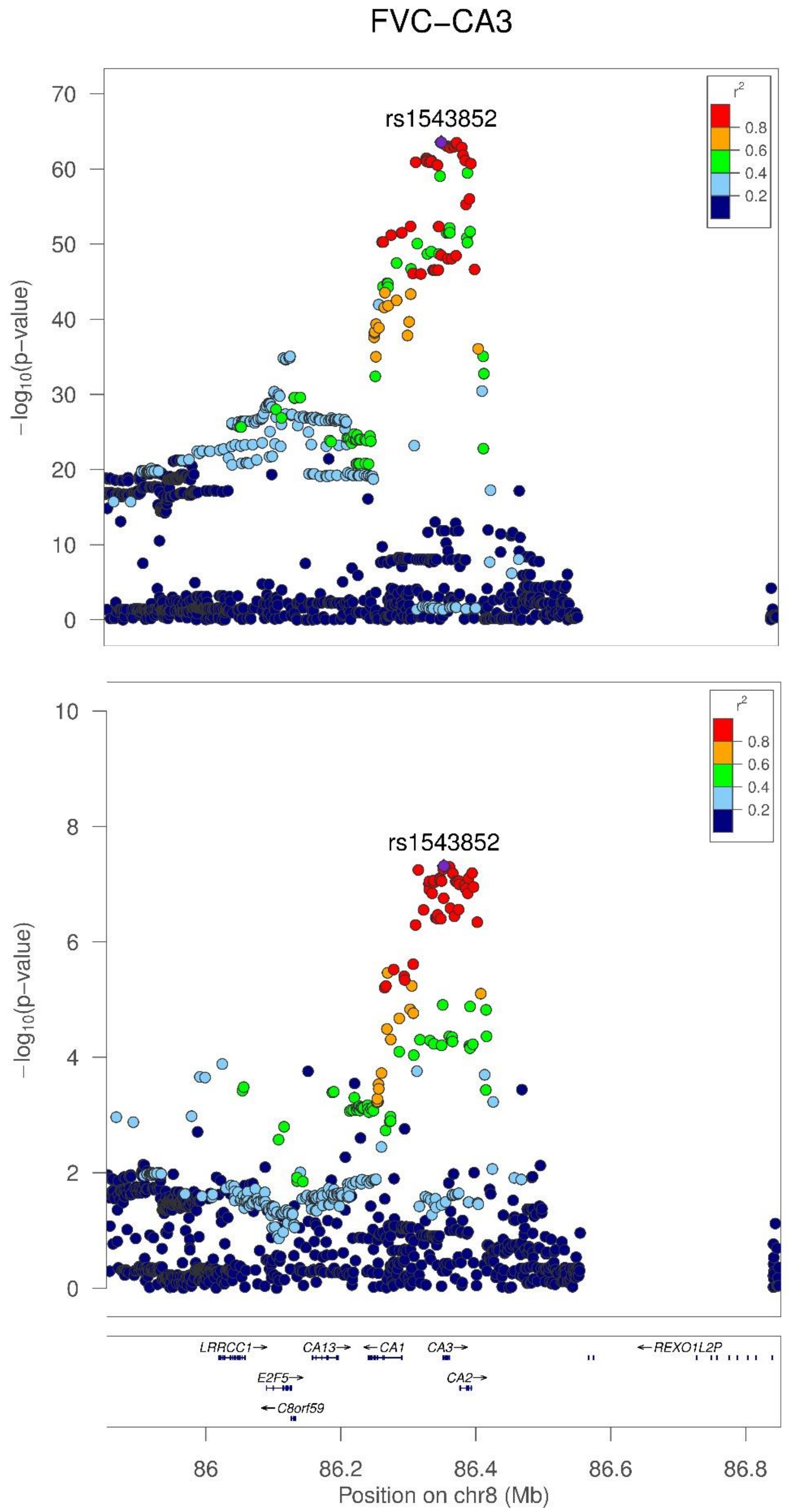

Figure S14: The LocusZoom plot for FVC and CCDC126

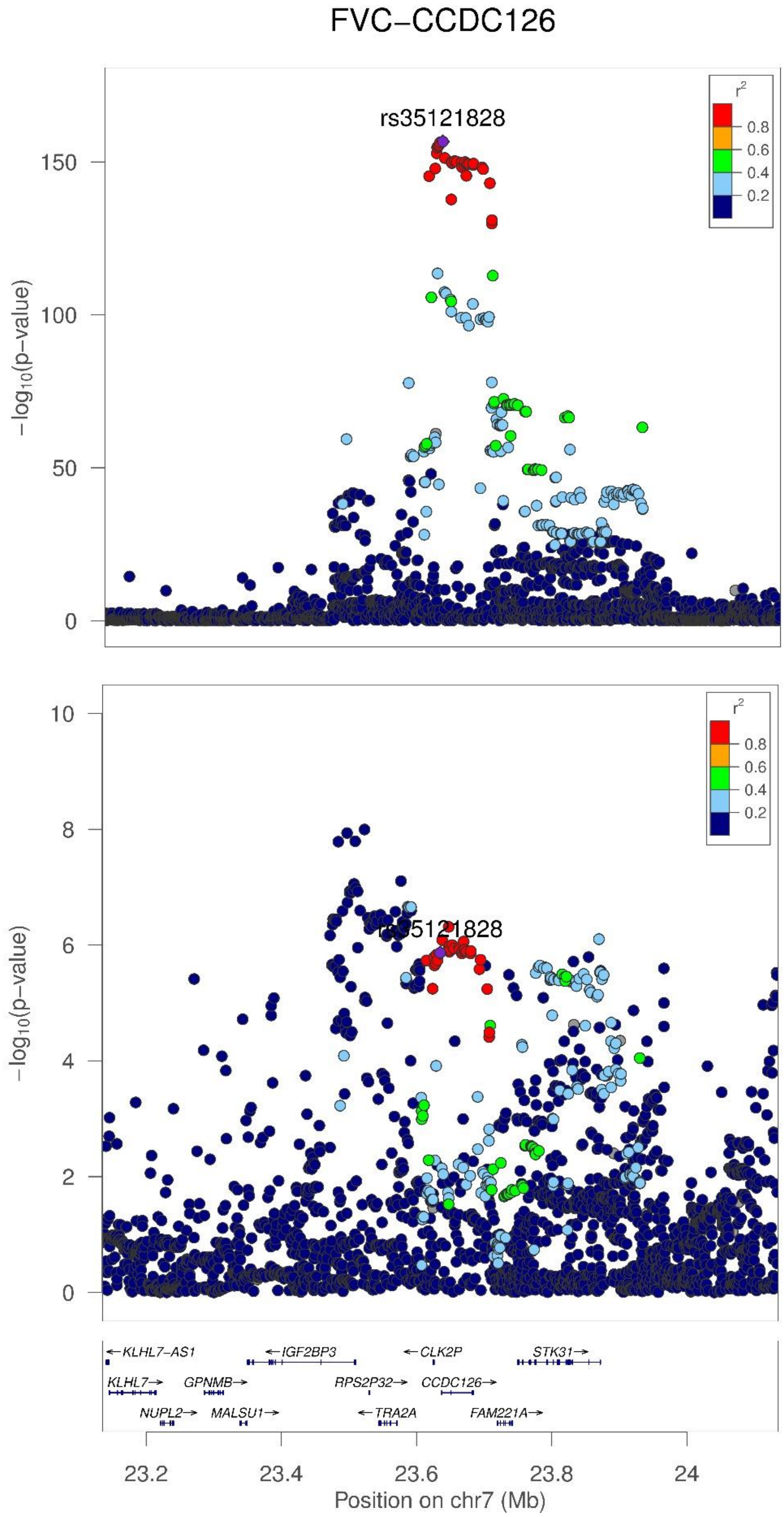

Figure S15: The LocusZoom plot for FVC and DNER

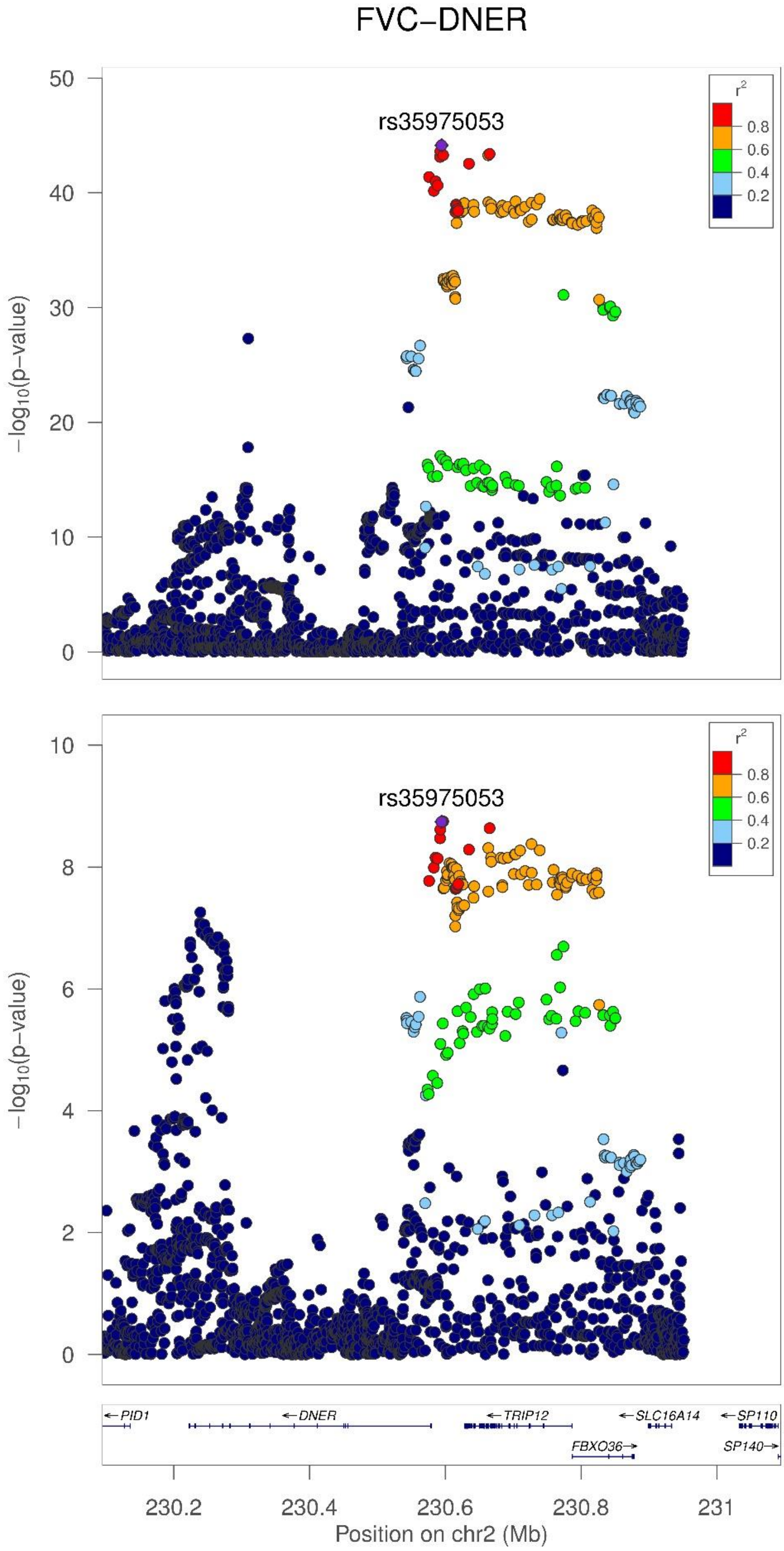

Figure S16: The LocusZoom plot for FVC and EFEMP1

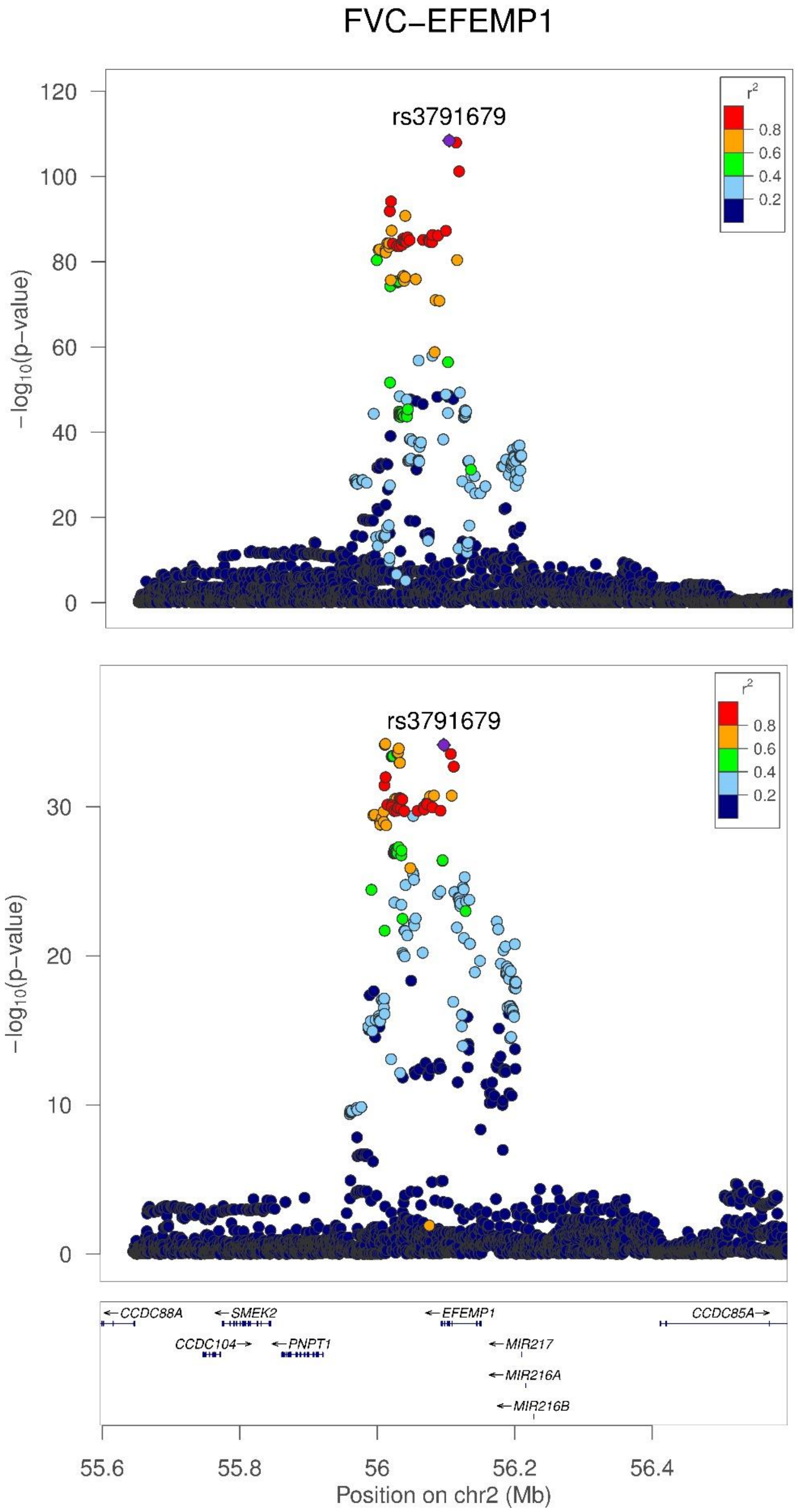

Figure S17: The LocusZoom plot for FVC and FER

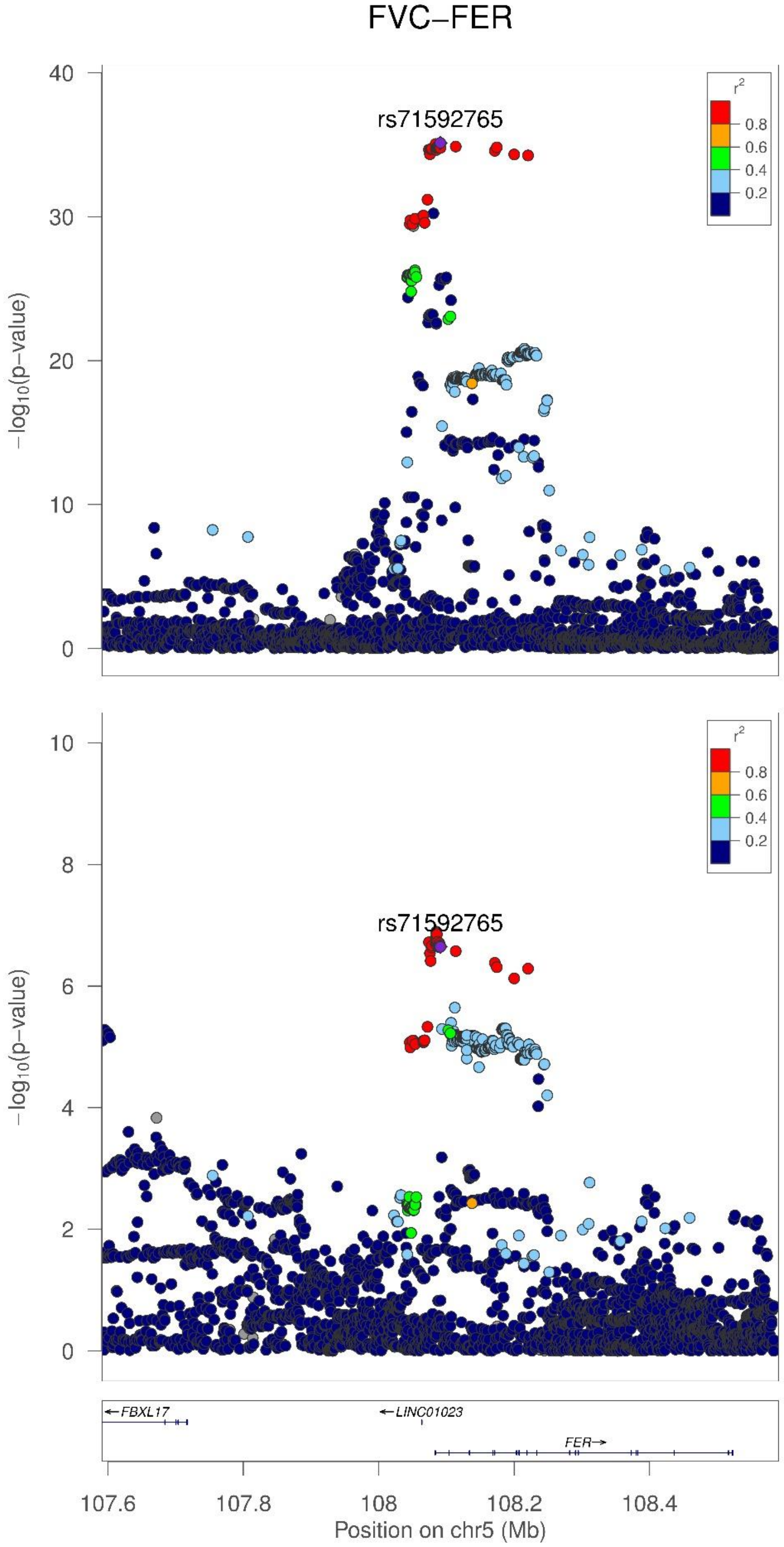

Figure S18: The LocusZoom plot for FVC and FKBP4

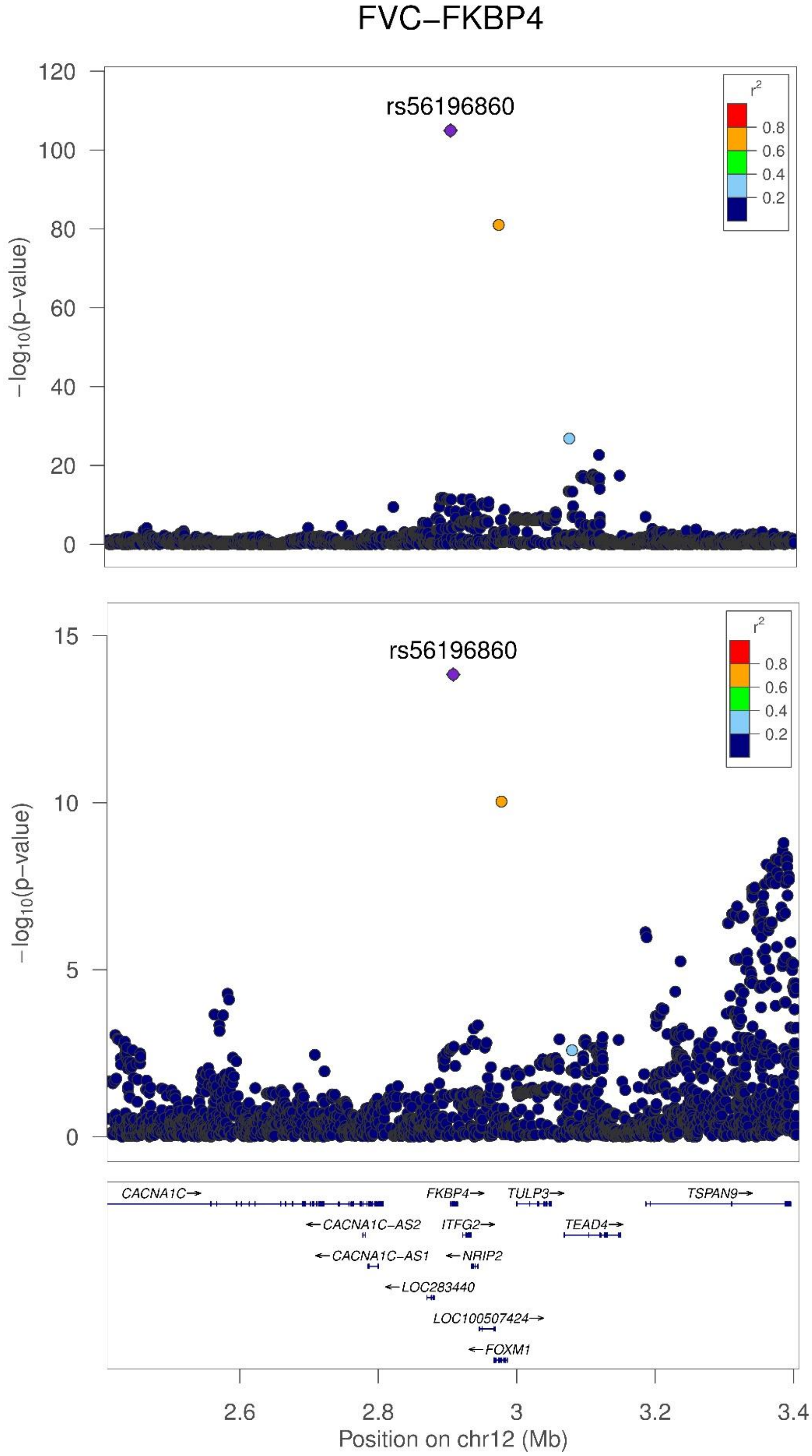

Figure S19: The LocusZoom plot for FVC and GMPR2

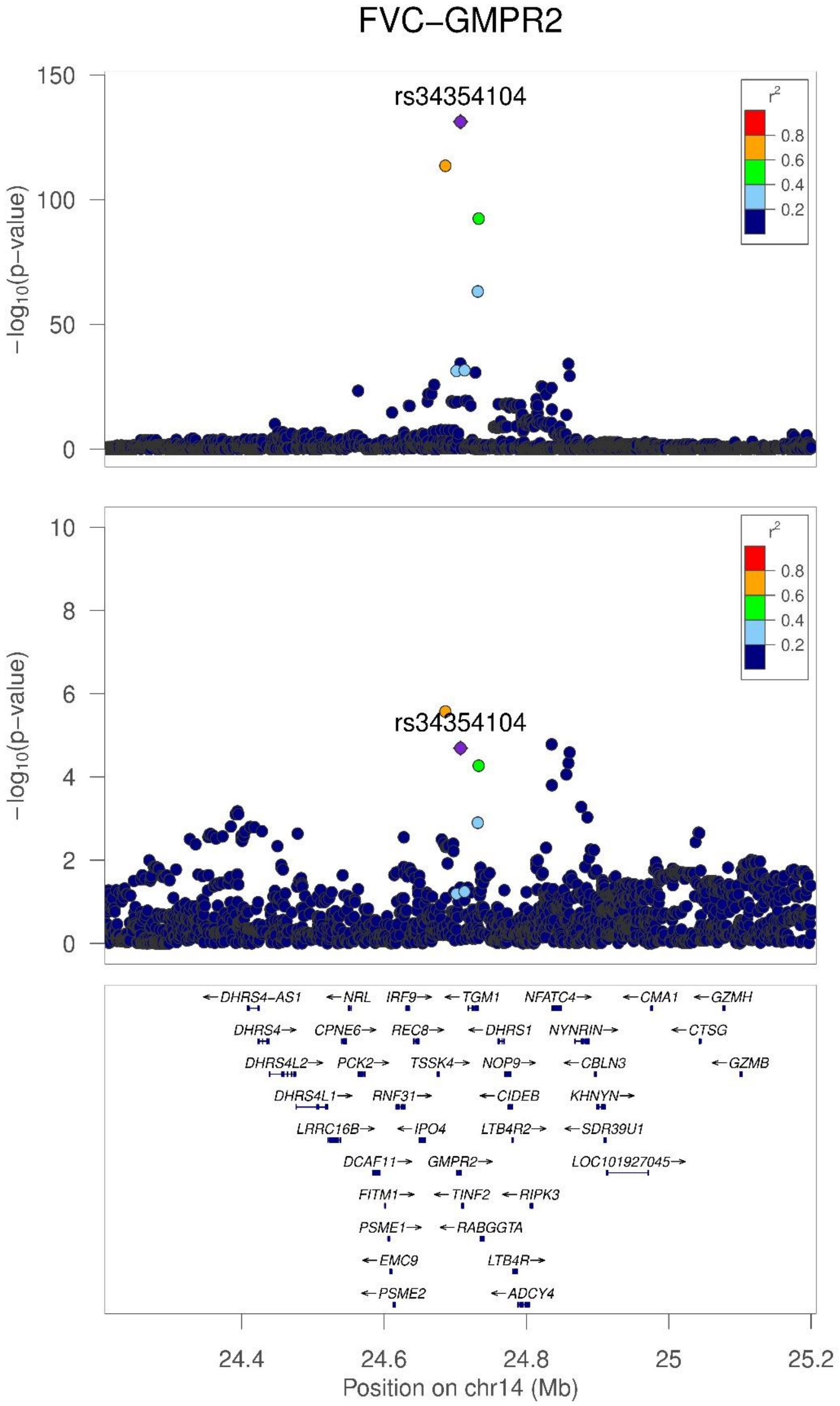

Figure S20: The LocusZoom plot for FVC and IL17RD

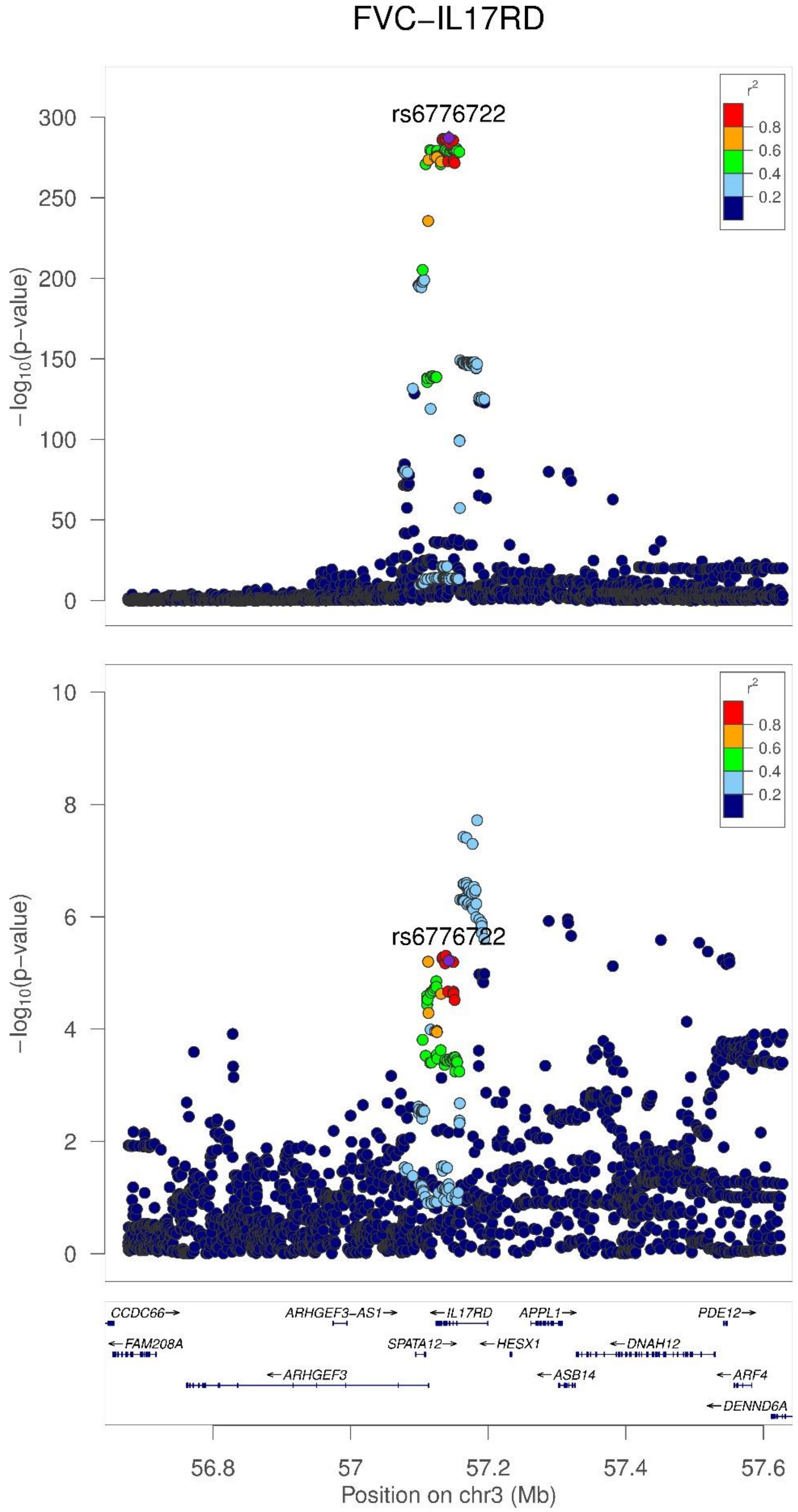

Figure S21: The LocusZoom plot for FVC and MRC2

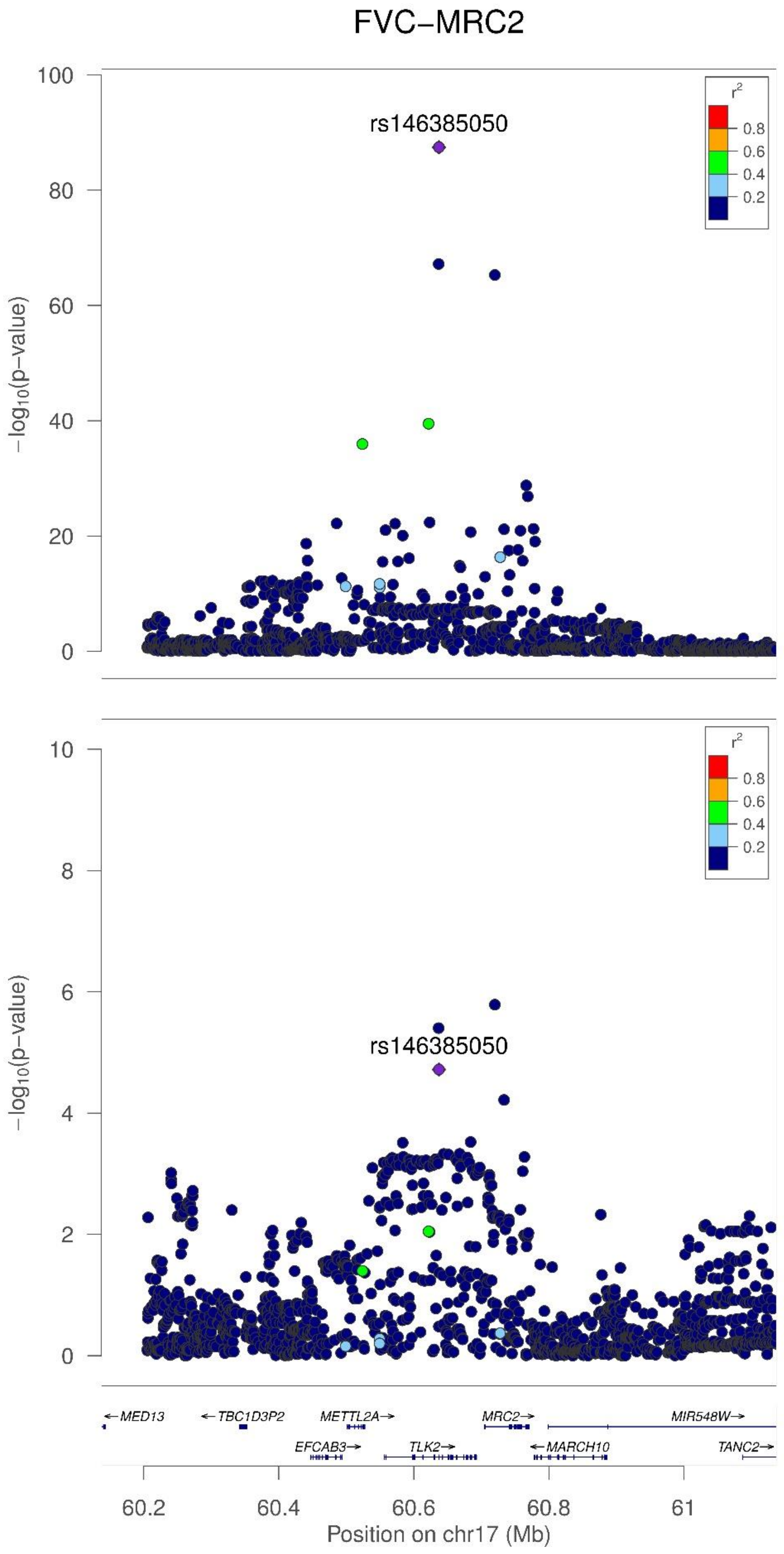

Figure S22: The LocusZoom plot for FVC and OGN

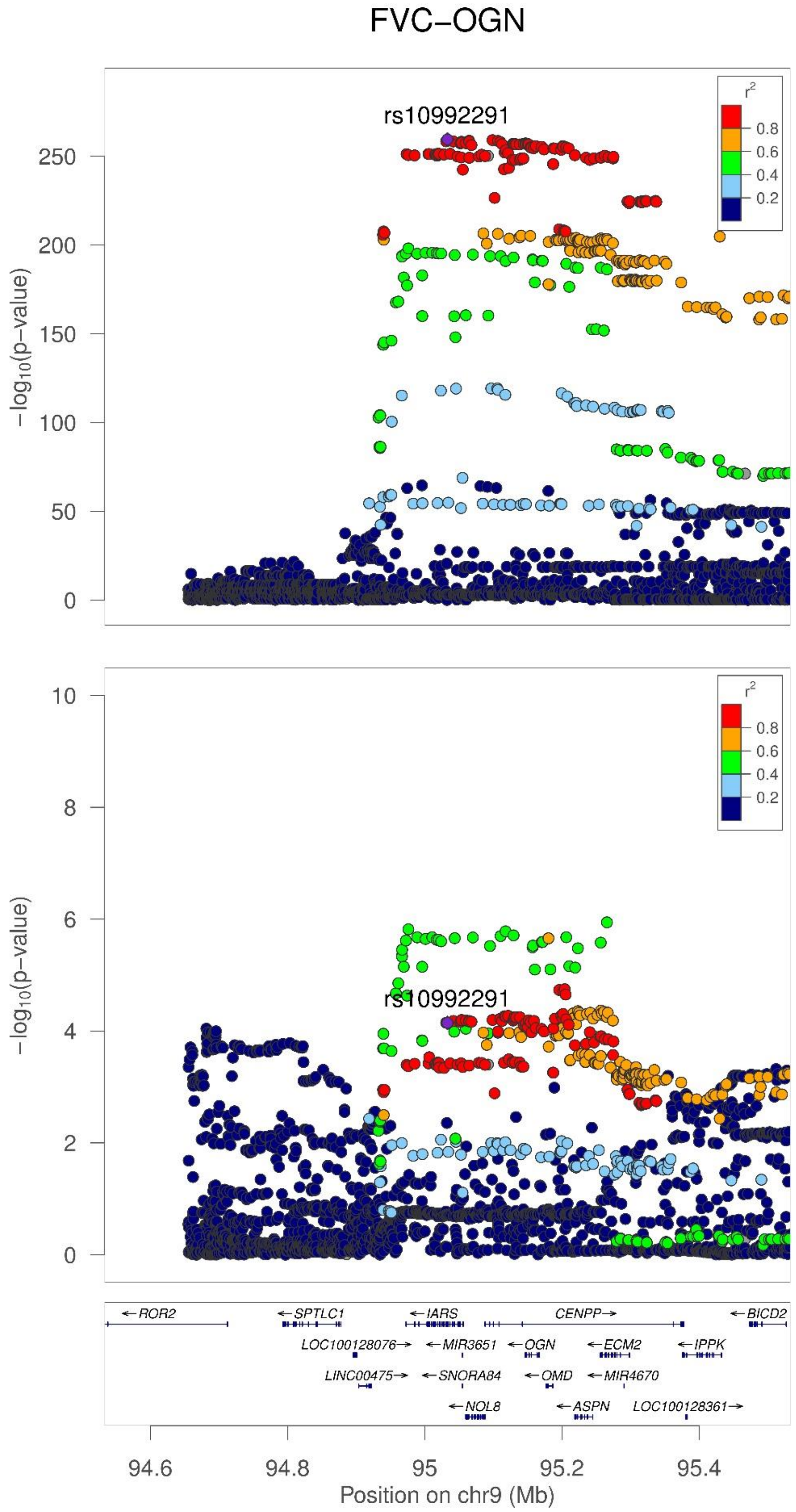

Figure S23: The LocusZoom plot for FVC and PARK7

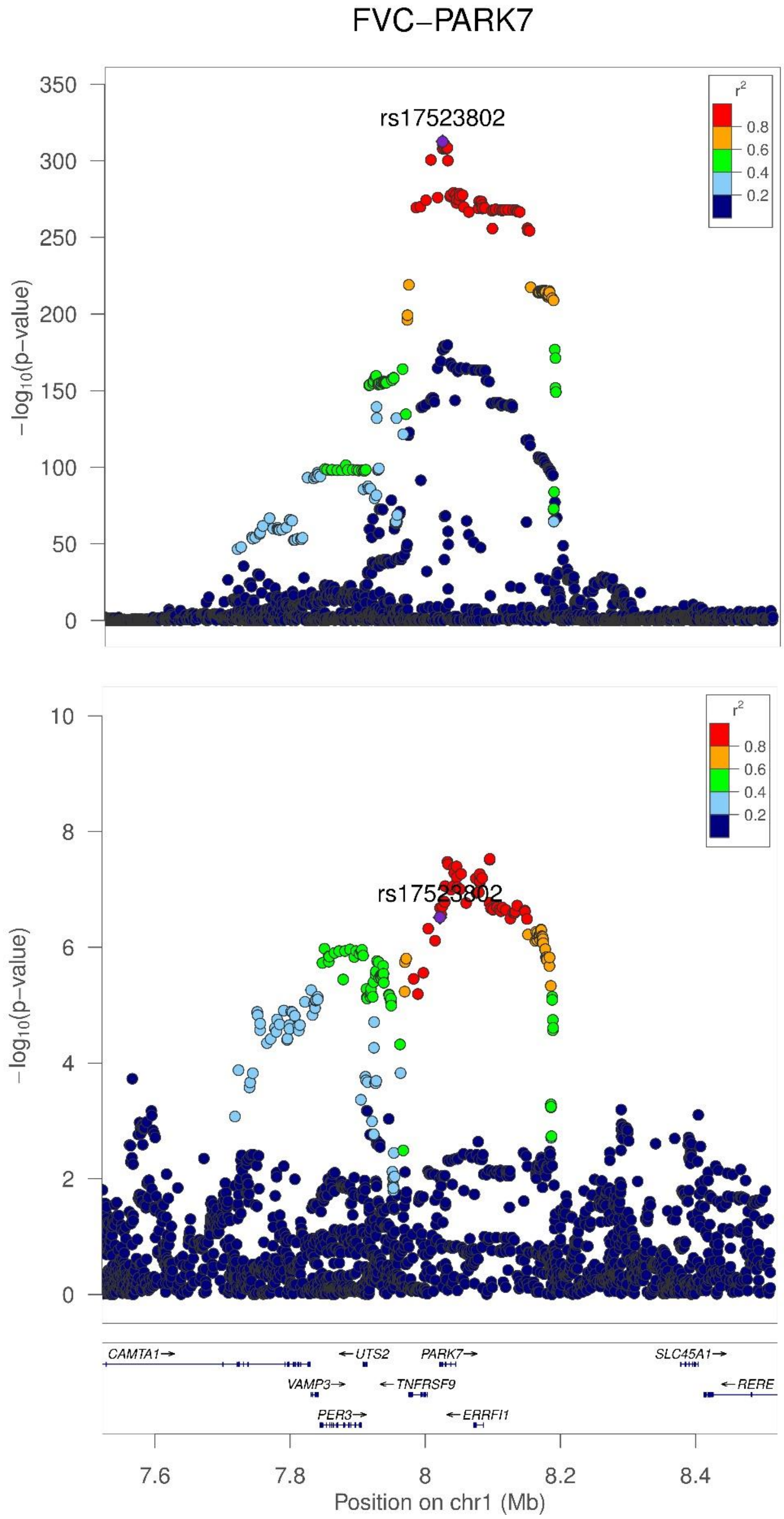

Figure S24: The LocusZoom plot for FVC and RHOC

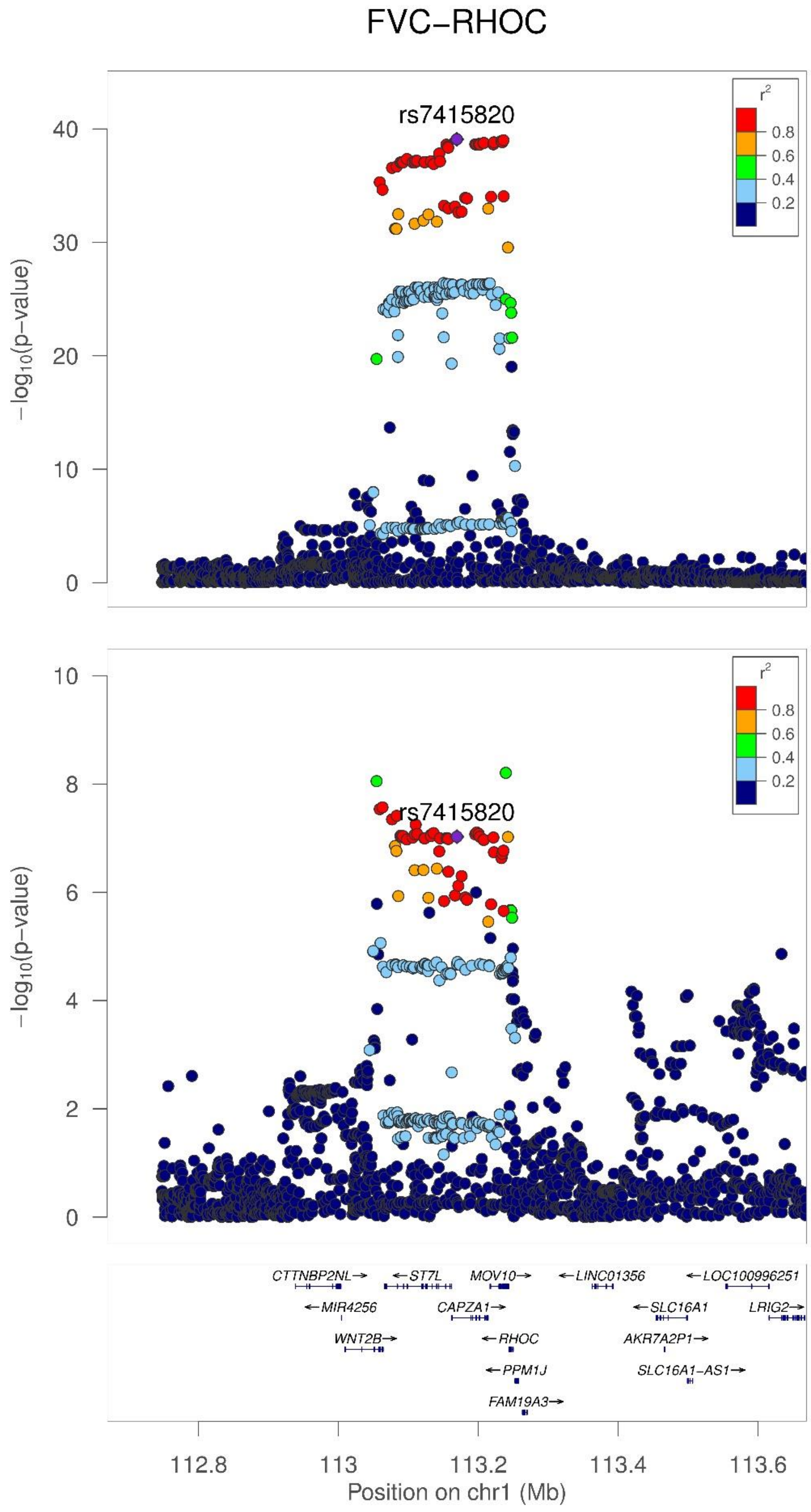

Figure S25: The LocusZoom plot for FVC and TESC

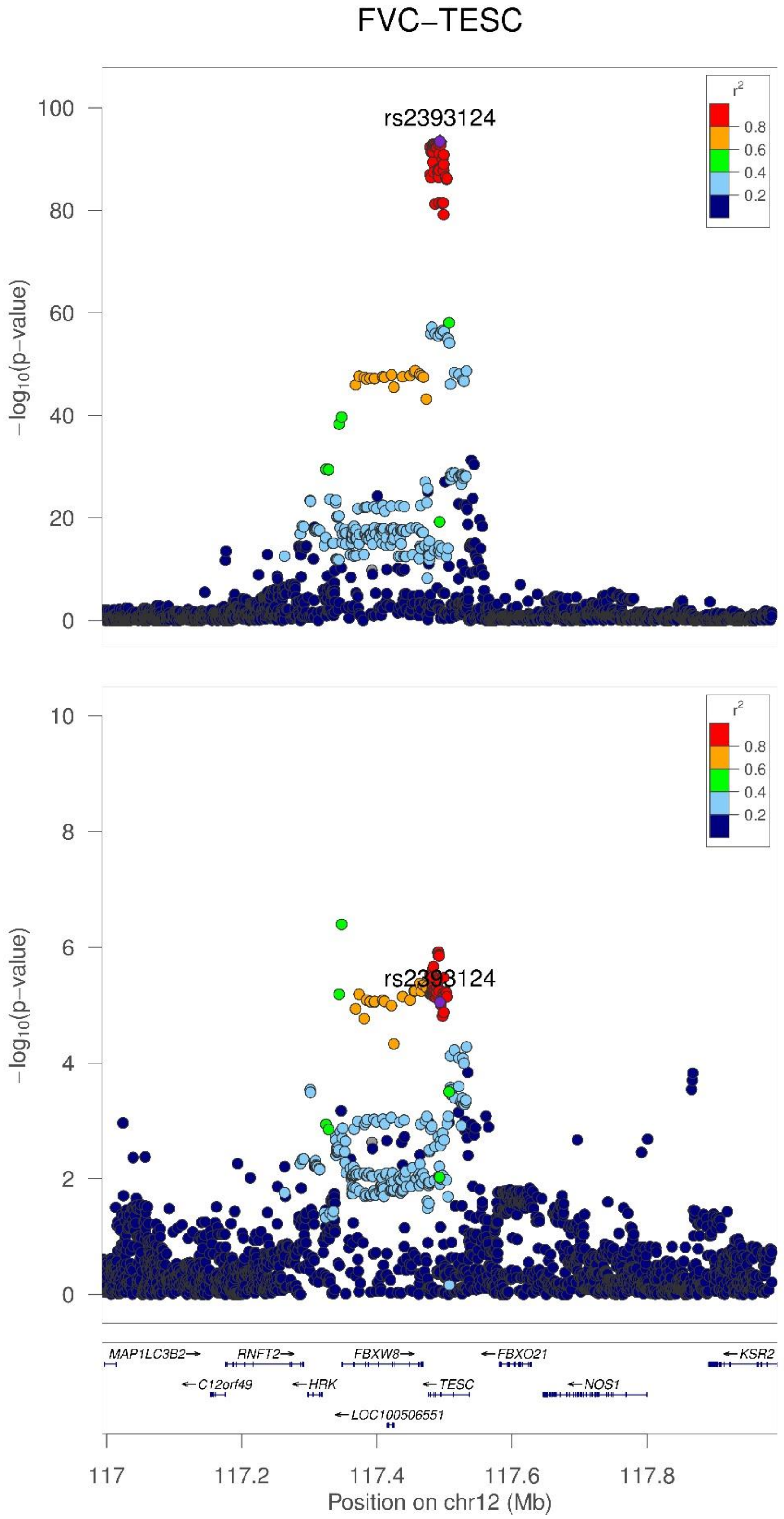

Figure S26: The LocusZoom plot for FVC and TRIL

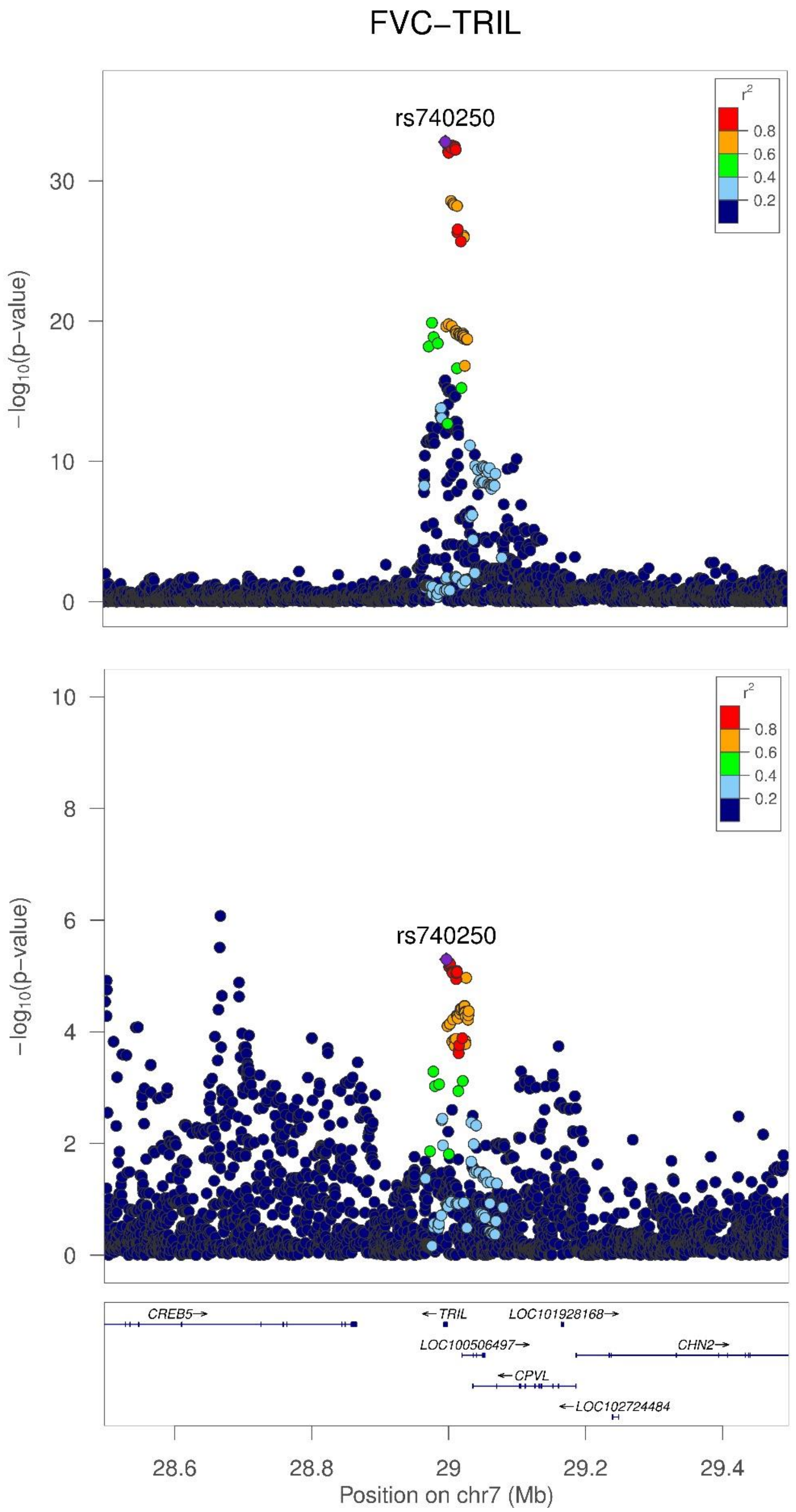

Figure S27: The LocusZoom plot for PEF and ACAN

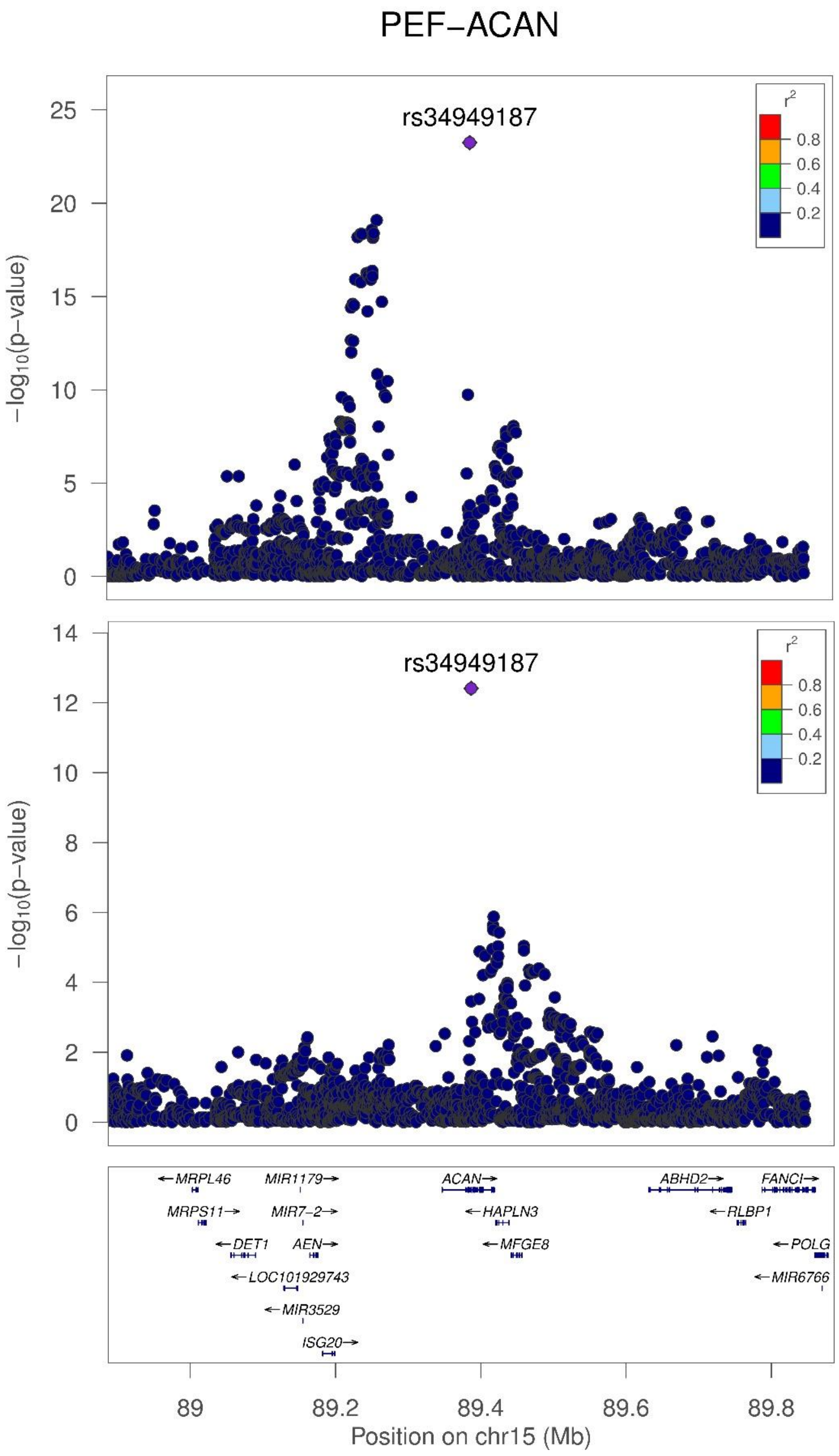

Figure S28: The LocusZoom plot for PEF and AGER

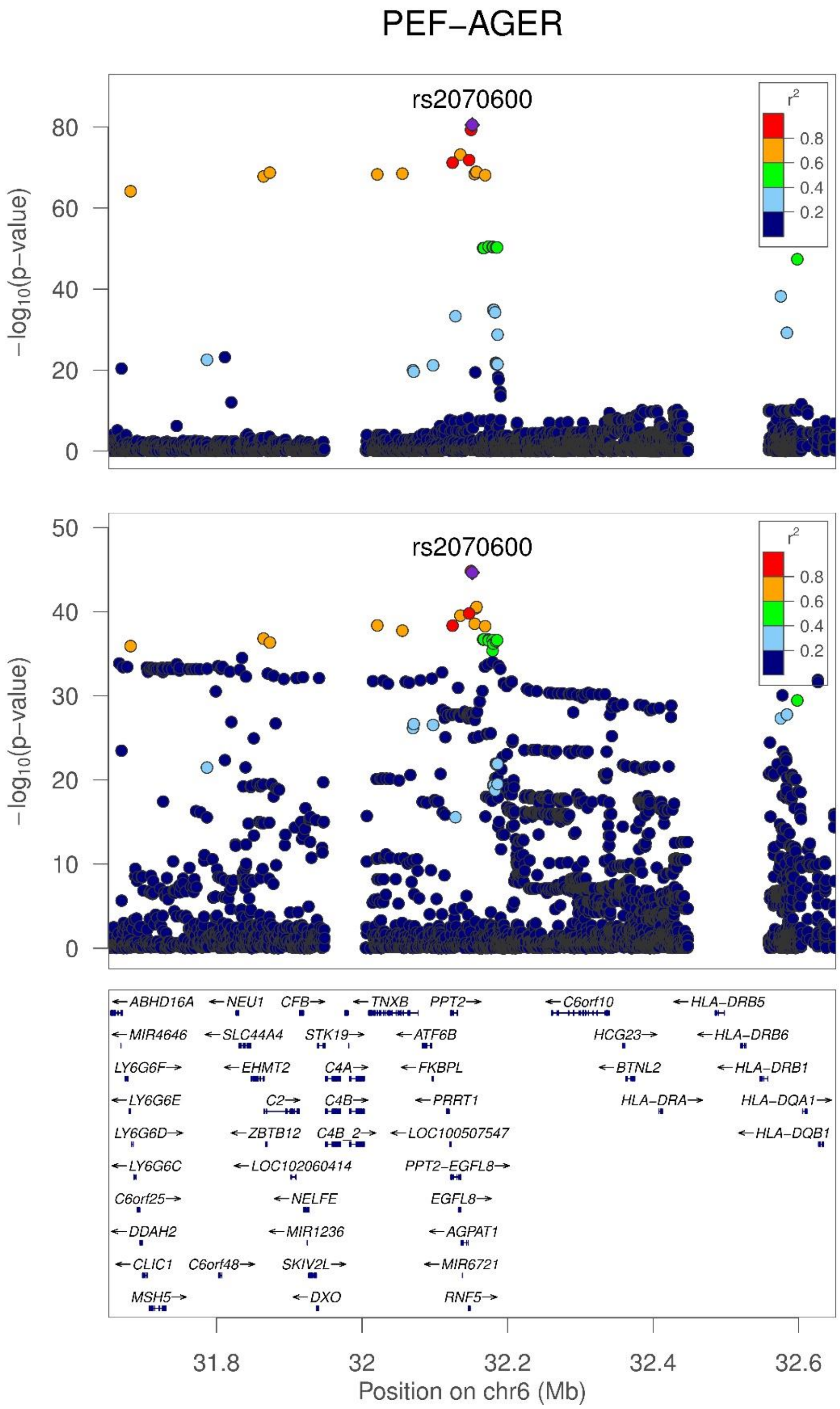

Figure S29: The LocusZoom plot for PEF and CACYBP

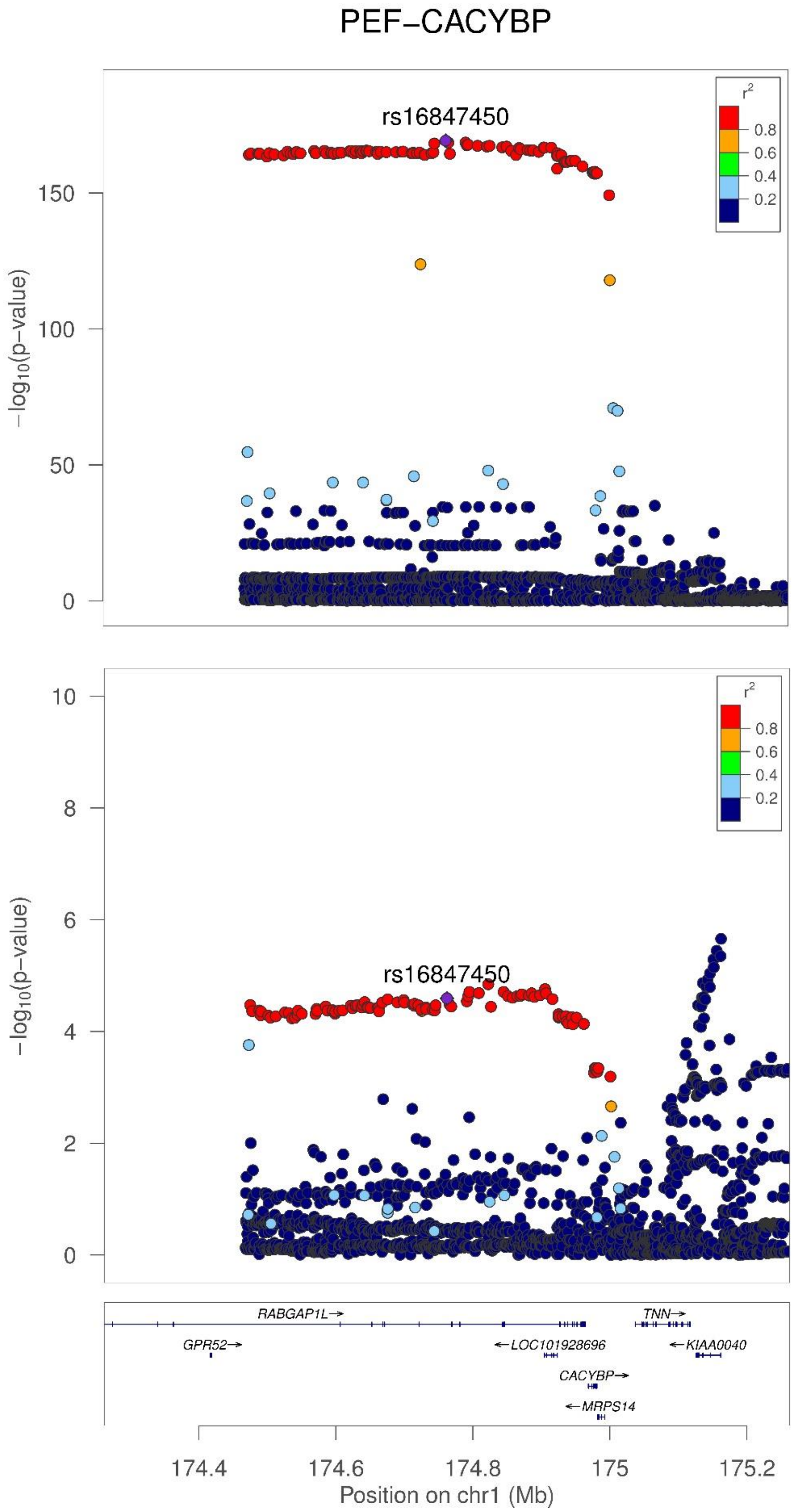

Figure S30: The LocusZoom plot for PEF and FKBP4

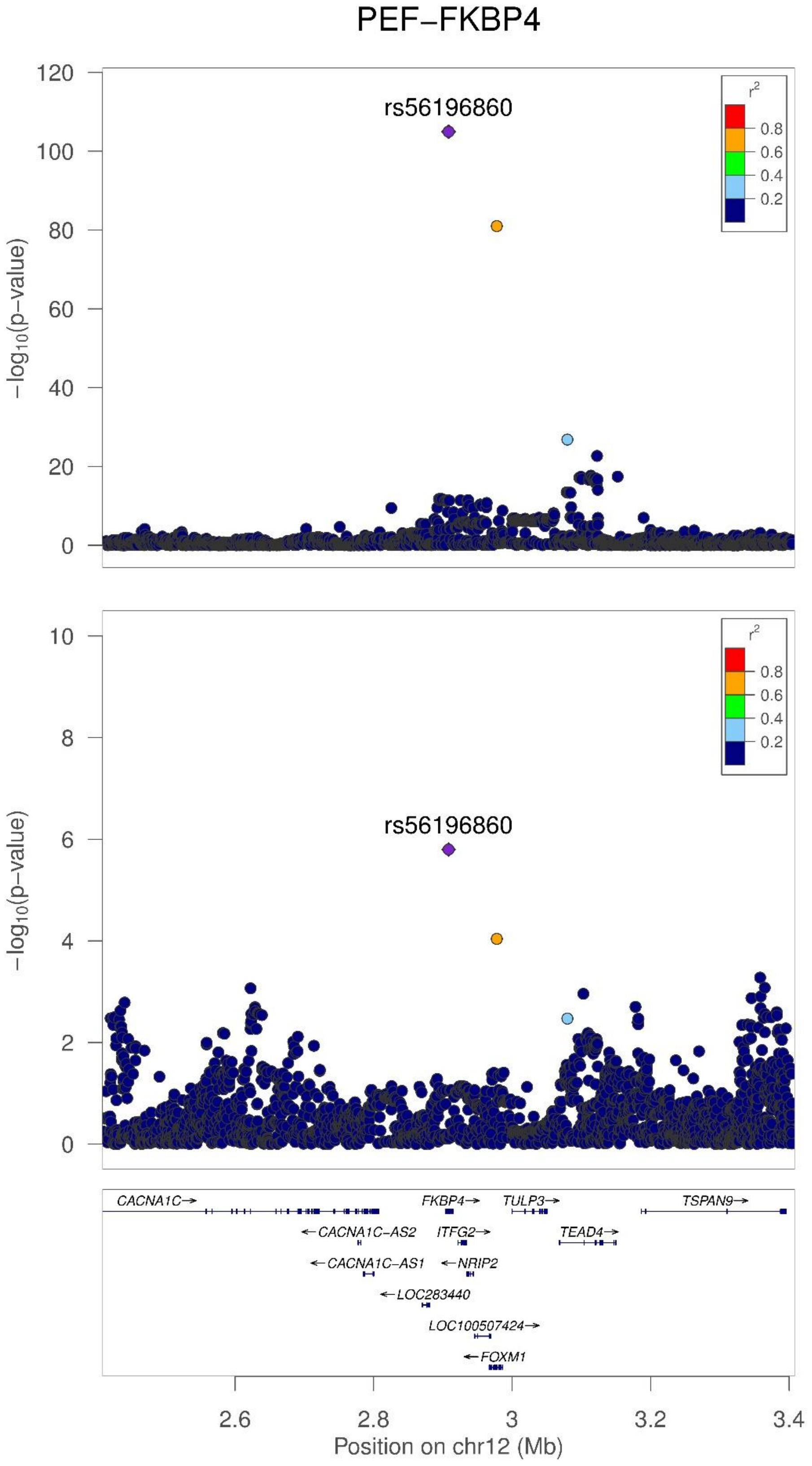

Figure S31: The LocusZoom plot for PEF and FN1

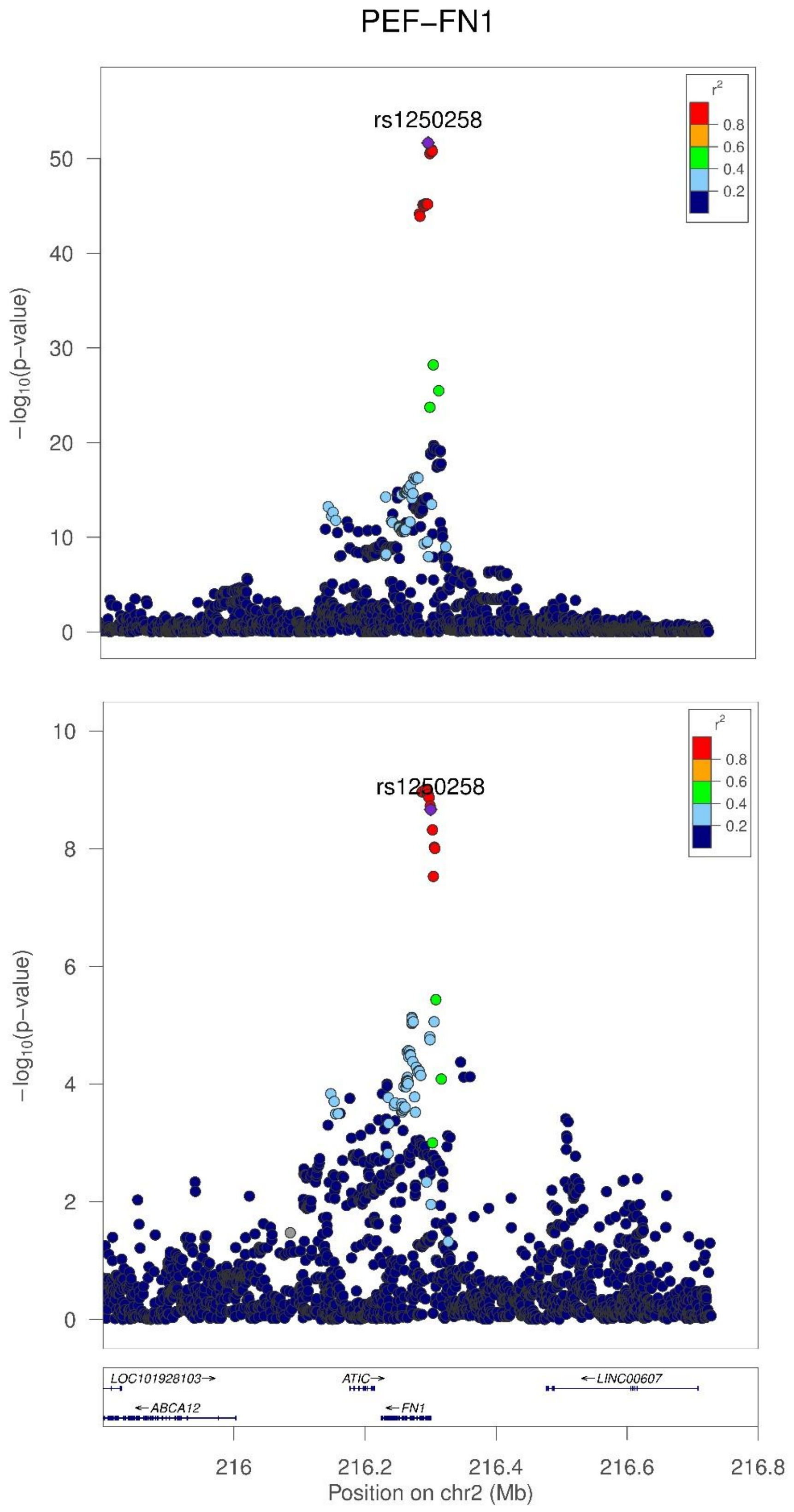

Figure S32: The LocusZoom plot for PEF and GM2A

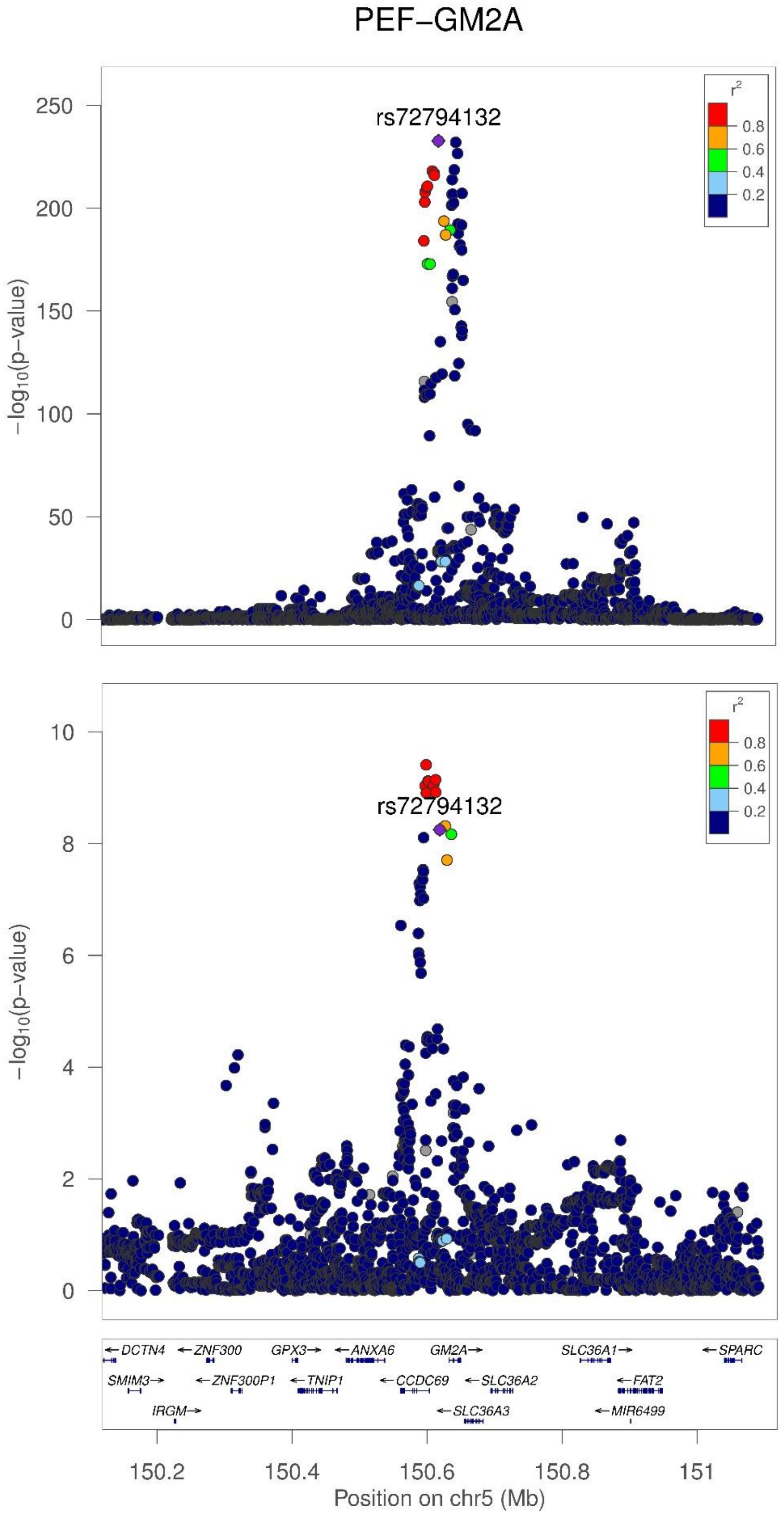

Figure S33: The LocusZoom plot for PEF and GSS

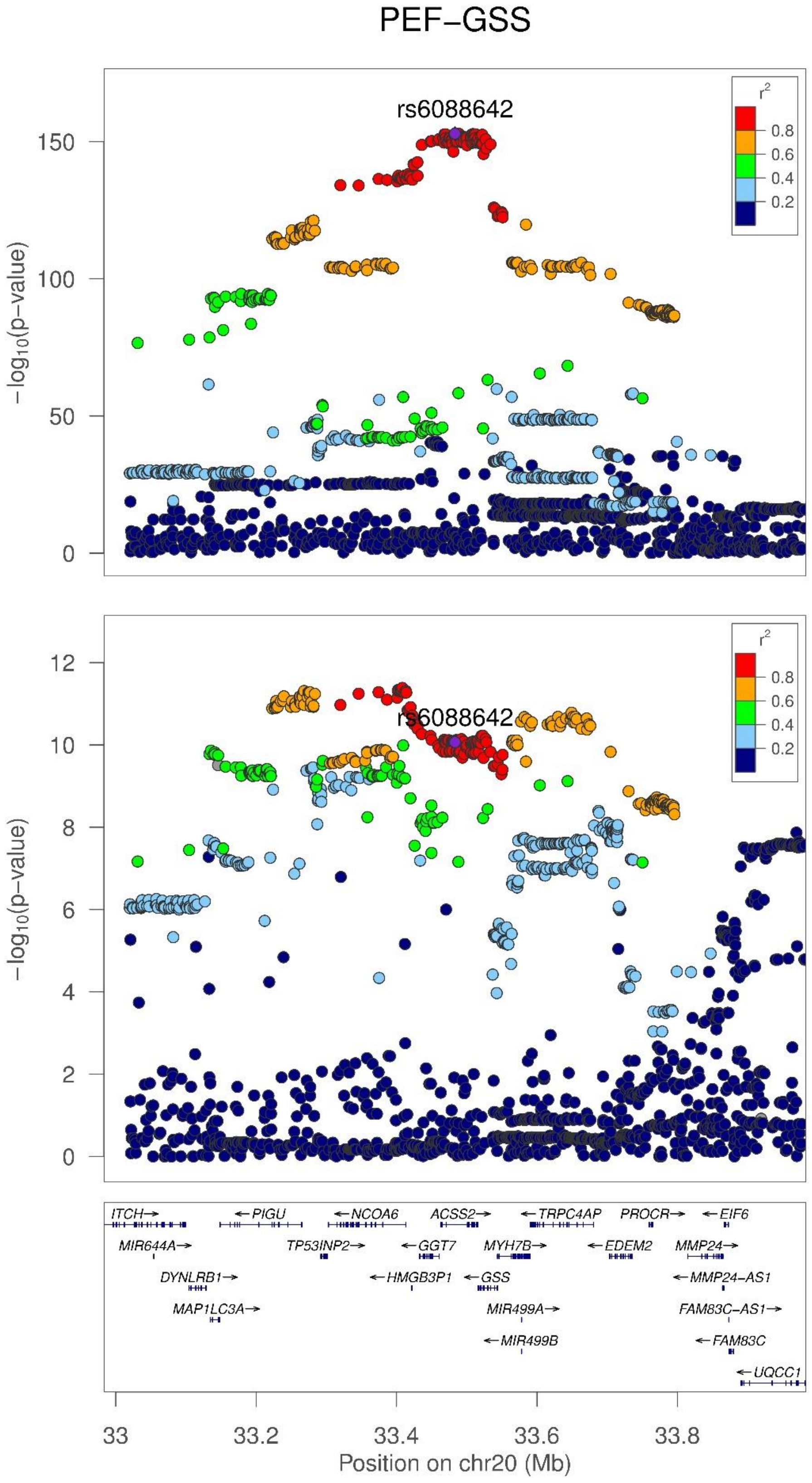

Figure S34: The LocusZoom plot for PEF and HAPLN1

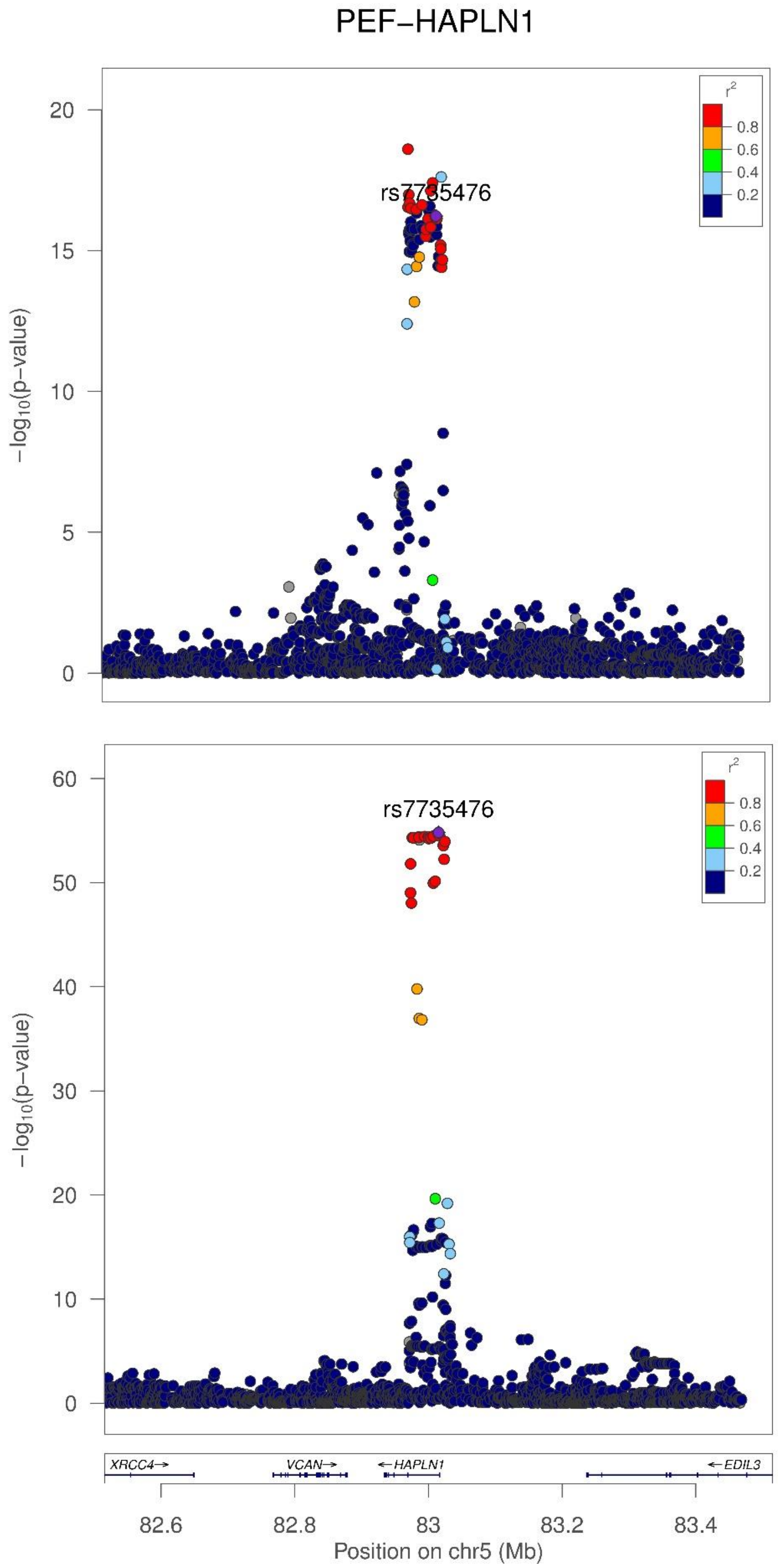

Figure S35: The LocusZoom plot for PEF and LILRB2

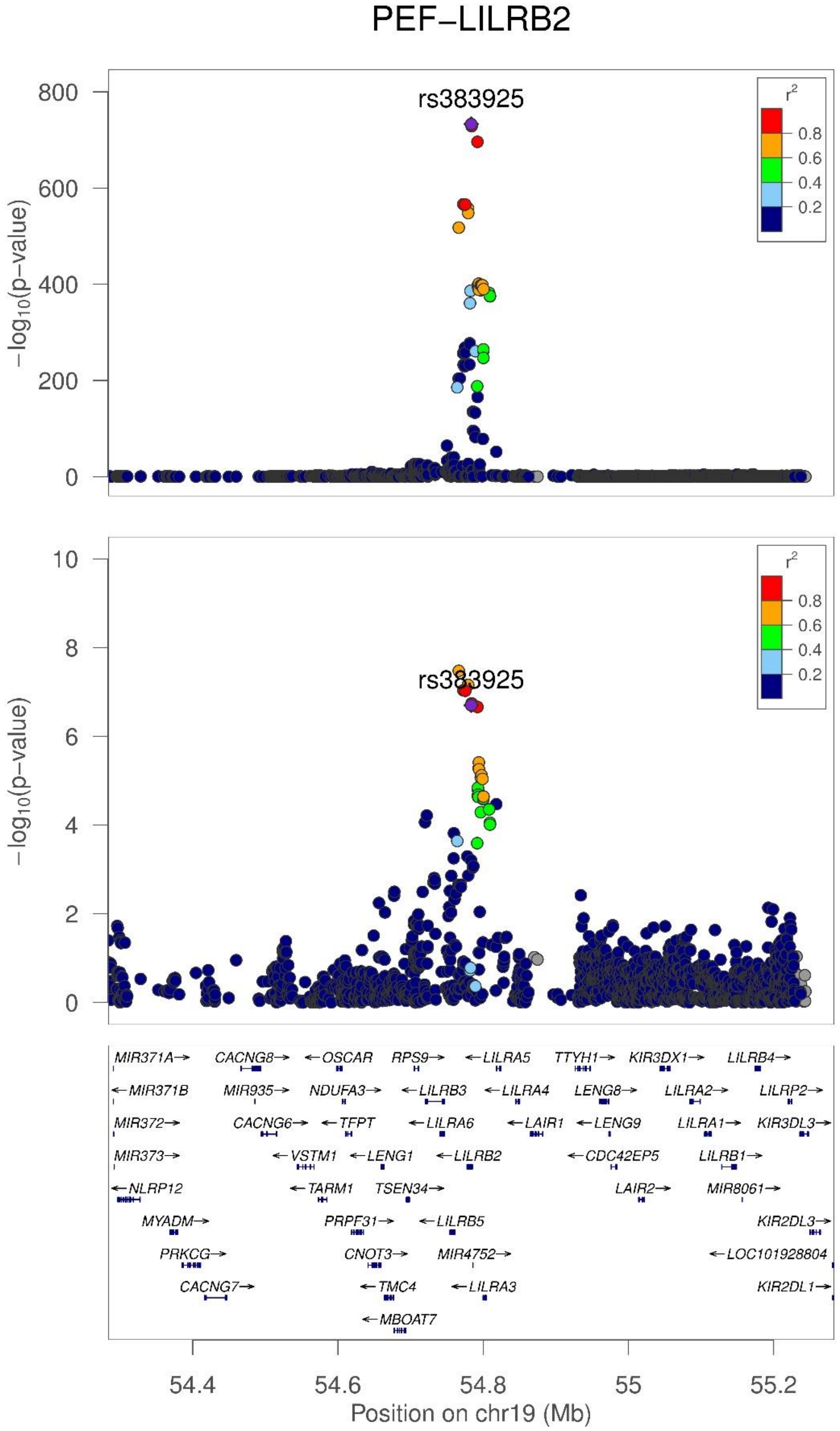

Figure S36: The LocusZoom plot for PEF and LTBP4

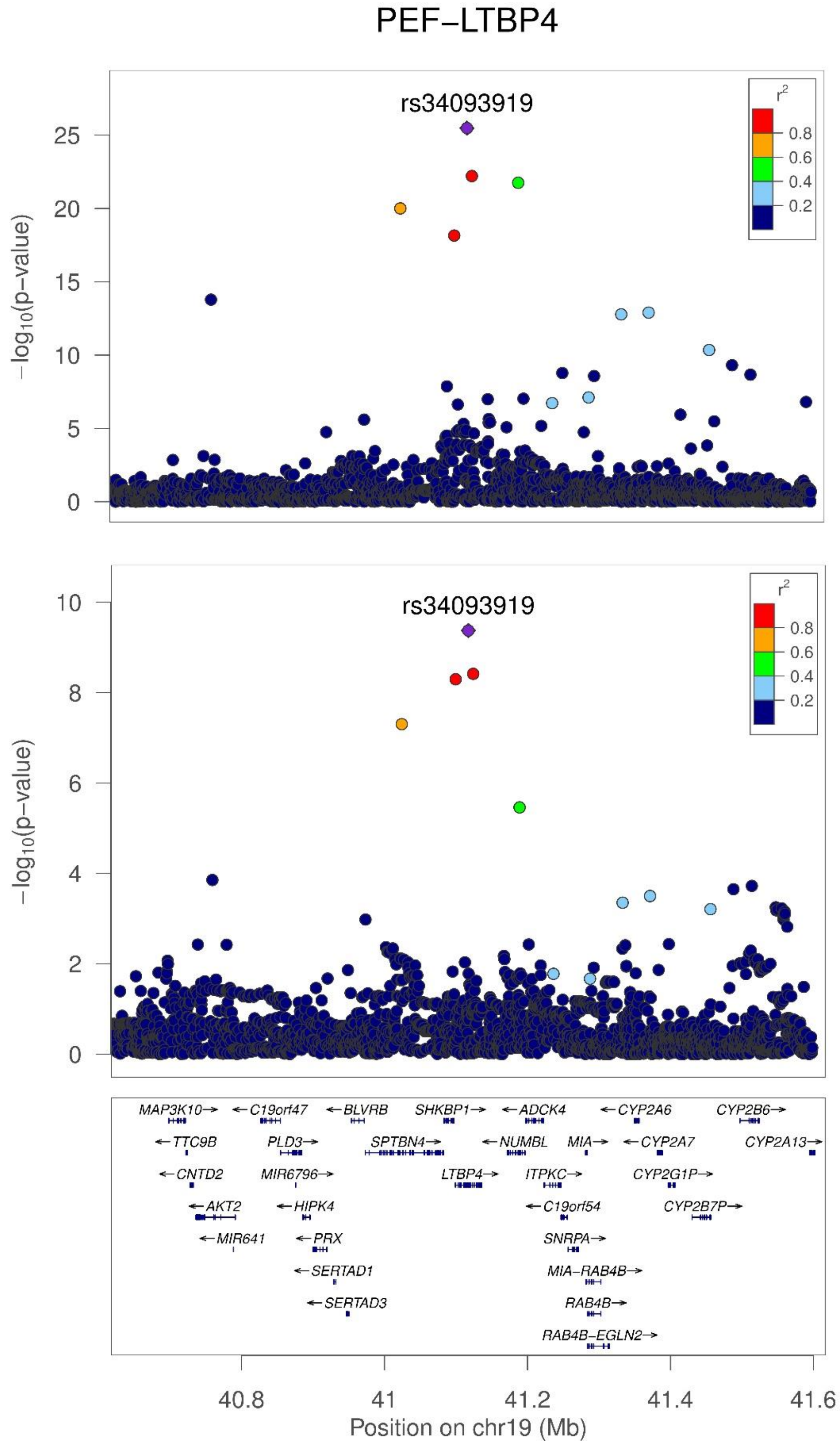

Figure S37: The LocusZoom plot for PEF and NOG

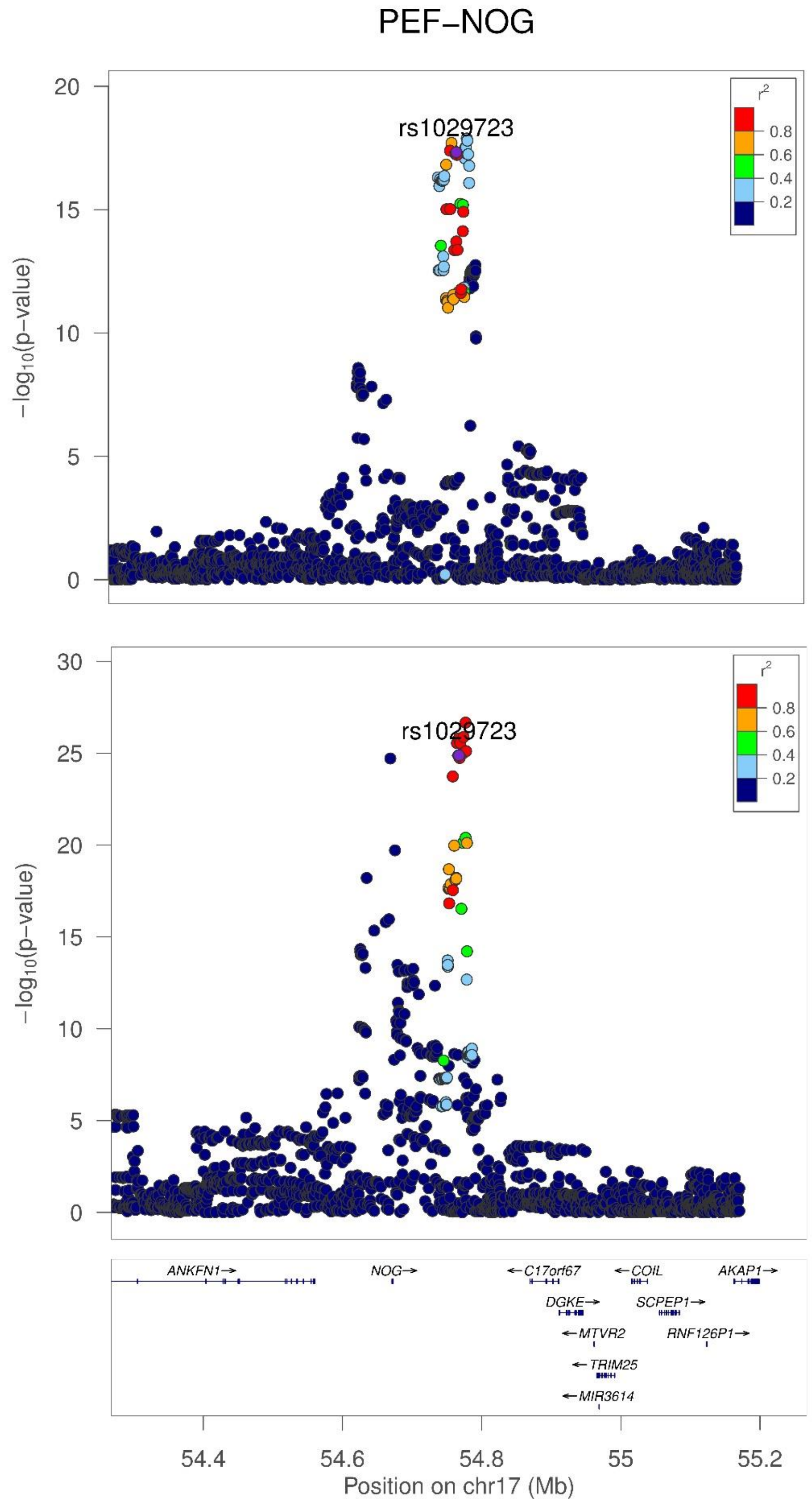

Figure S38: The LocusZoom plot for PEF and NPNT

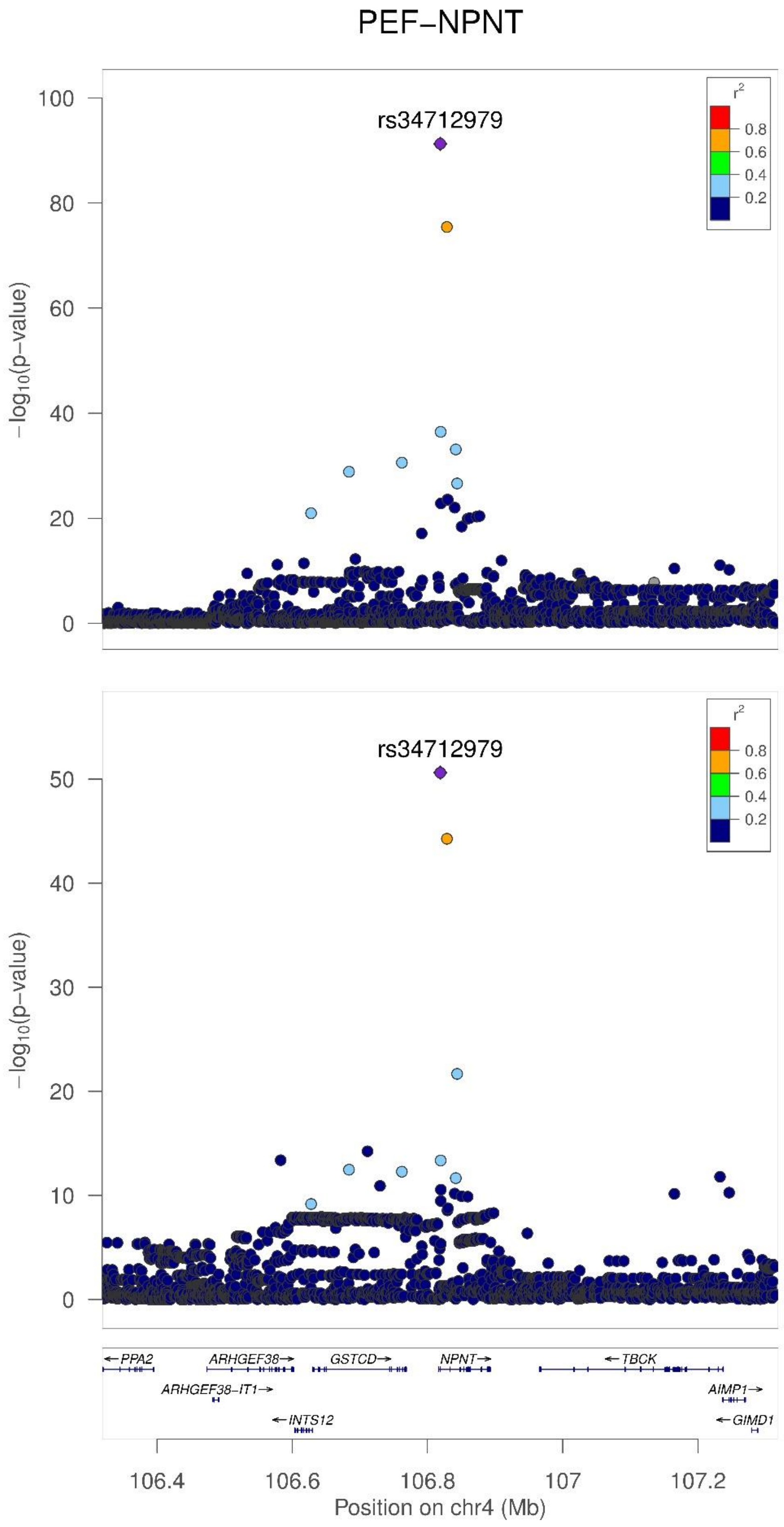

Figure S39: The LocusZoom plot for PEF and SCARF2

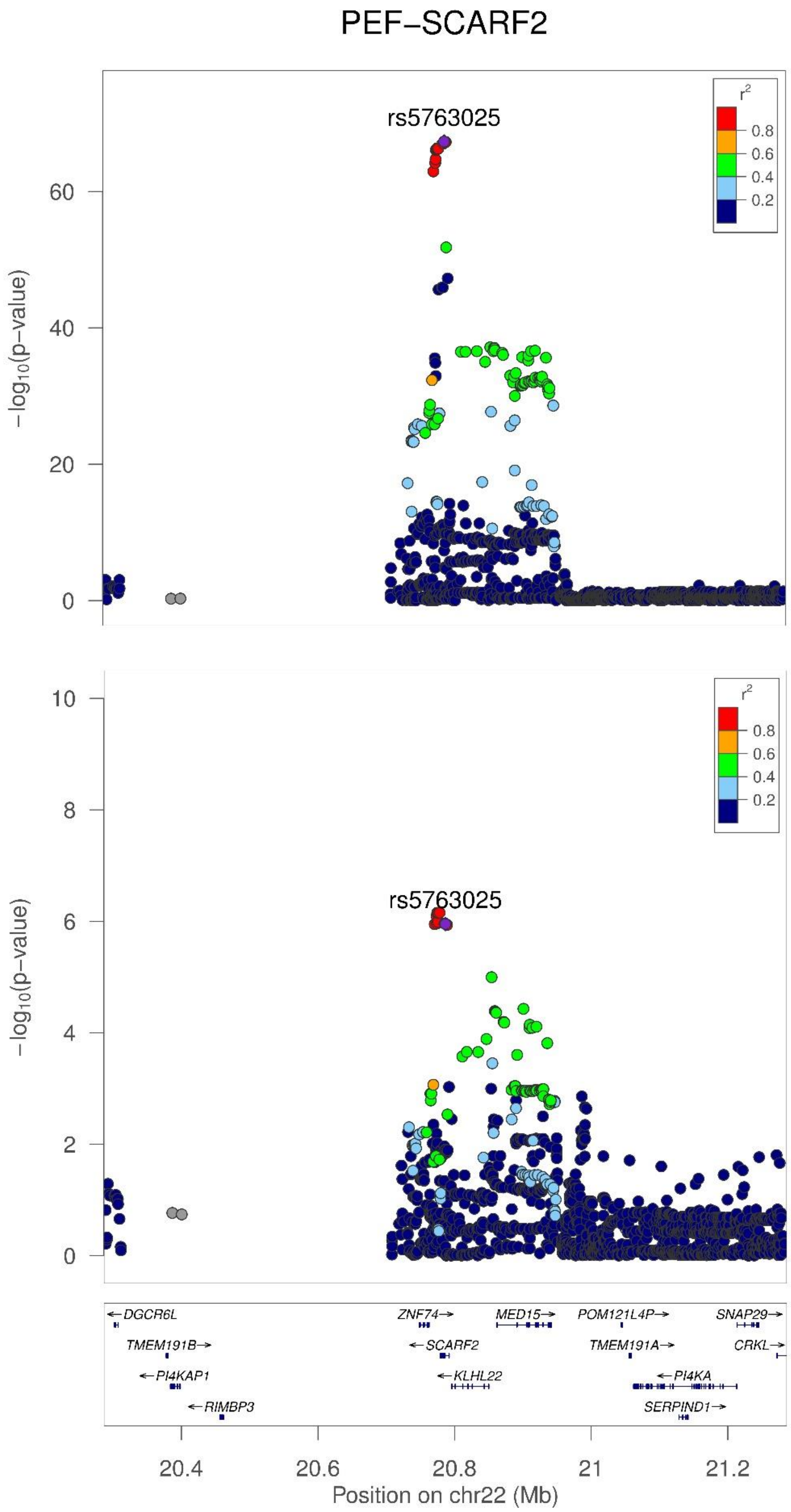

Figure S40: The LocusZoom plot for PEF and XPNPEP1

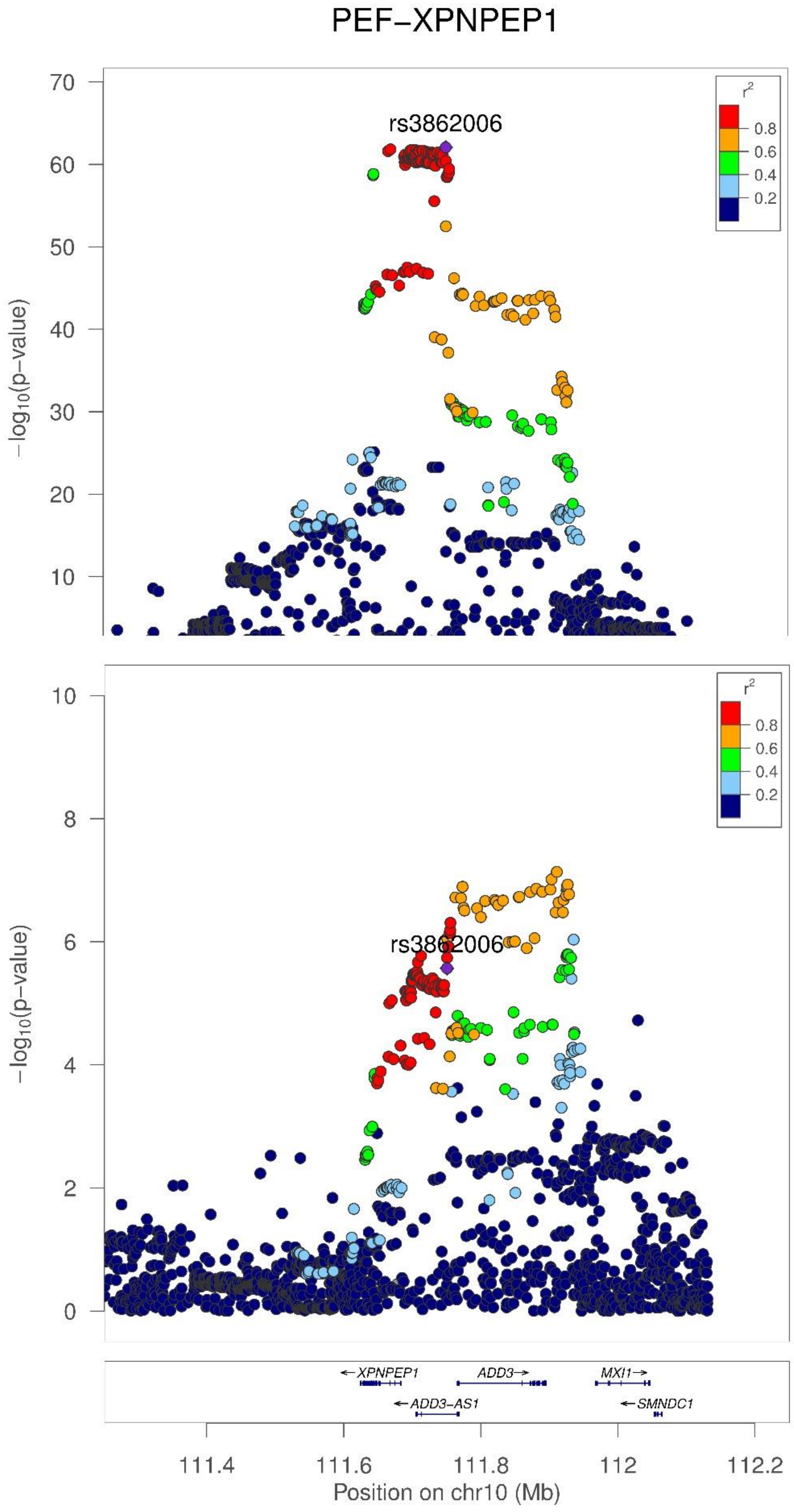

Figure S41: The LocusZoom plot for FEV1/FVC and AGER

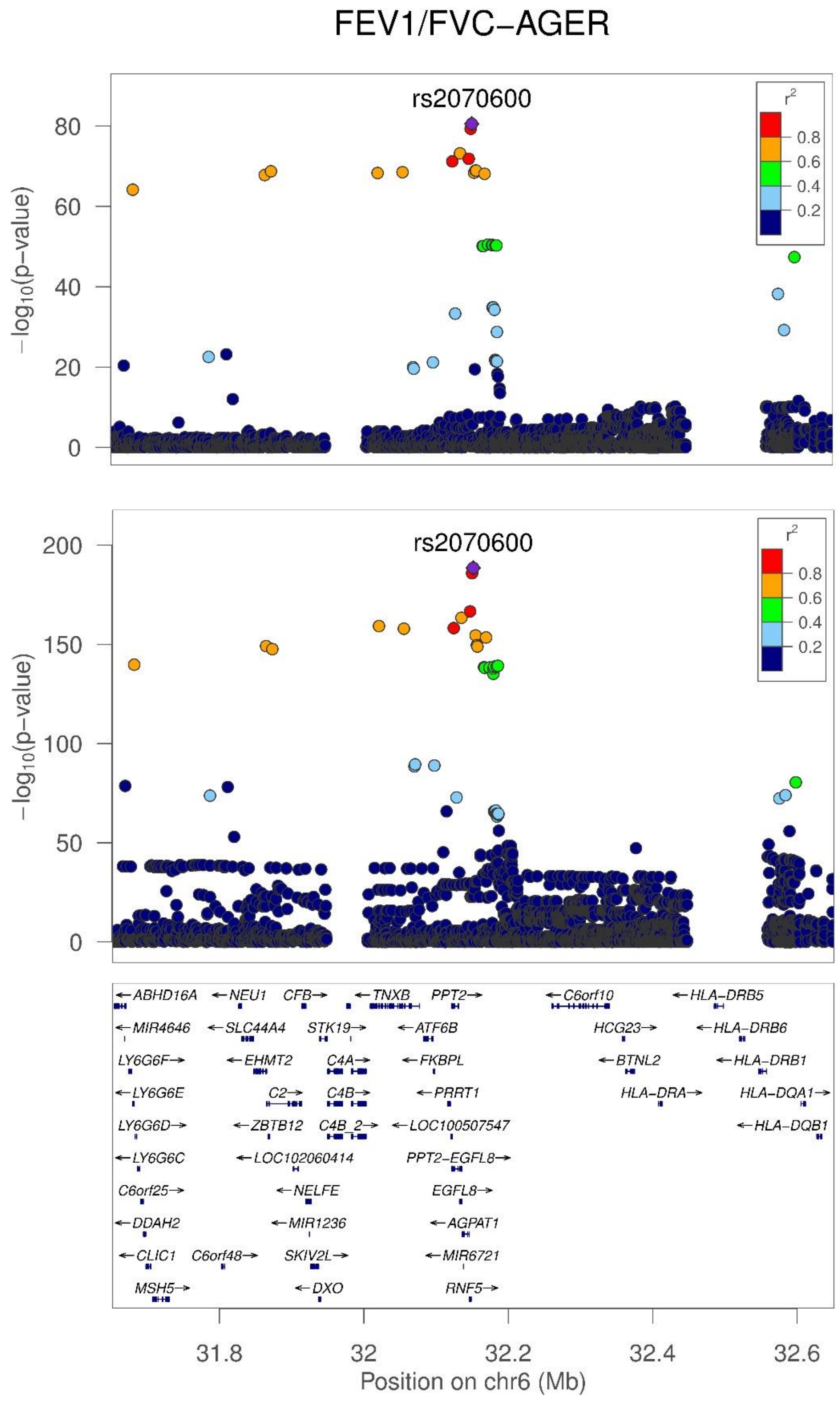

Figure S42: The LocusZoom plot for FEV1/FVC and ARFIP1

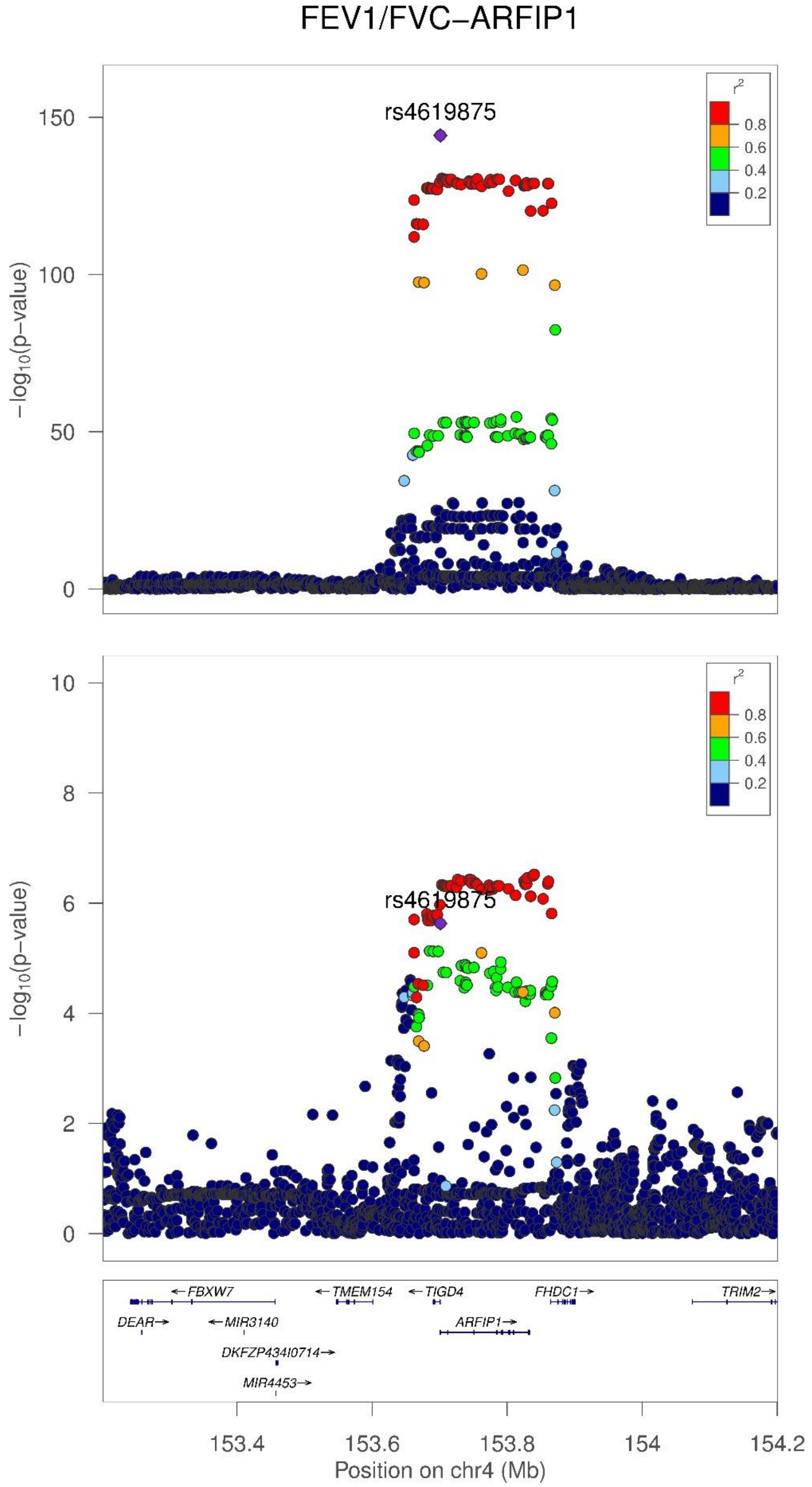

Figure S43: The LocusZoom plot for FEV1/FVC and BOC

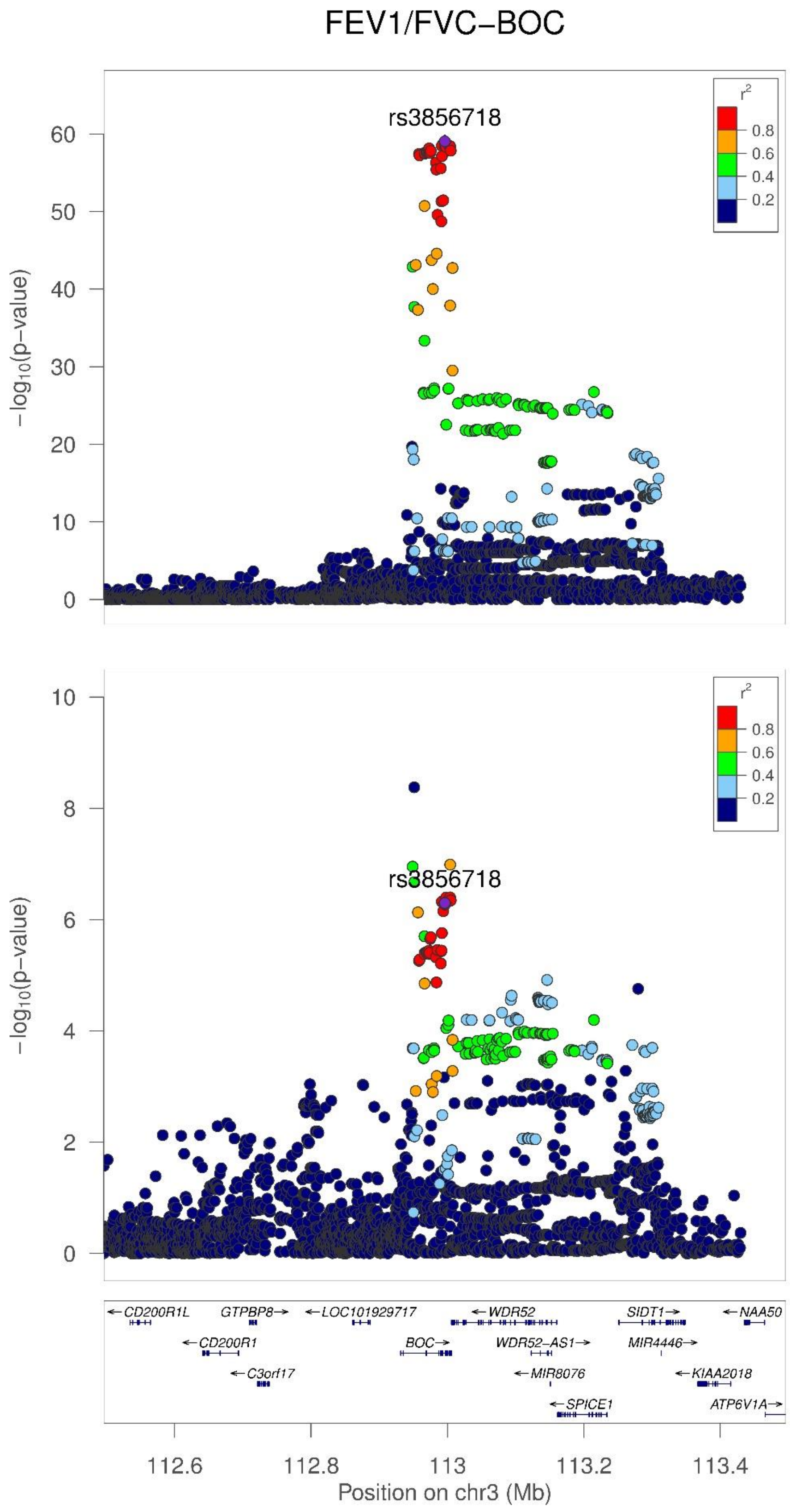

Figure S44: The LocusZoom plot for FEV1/FVC and BTN3A3

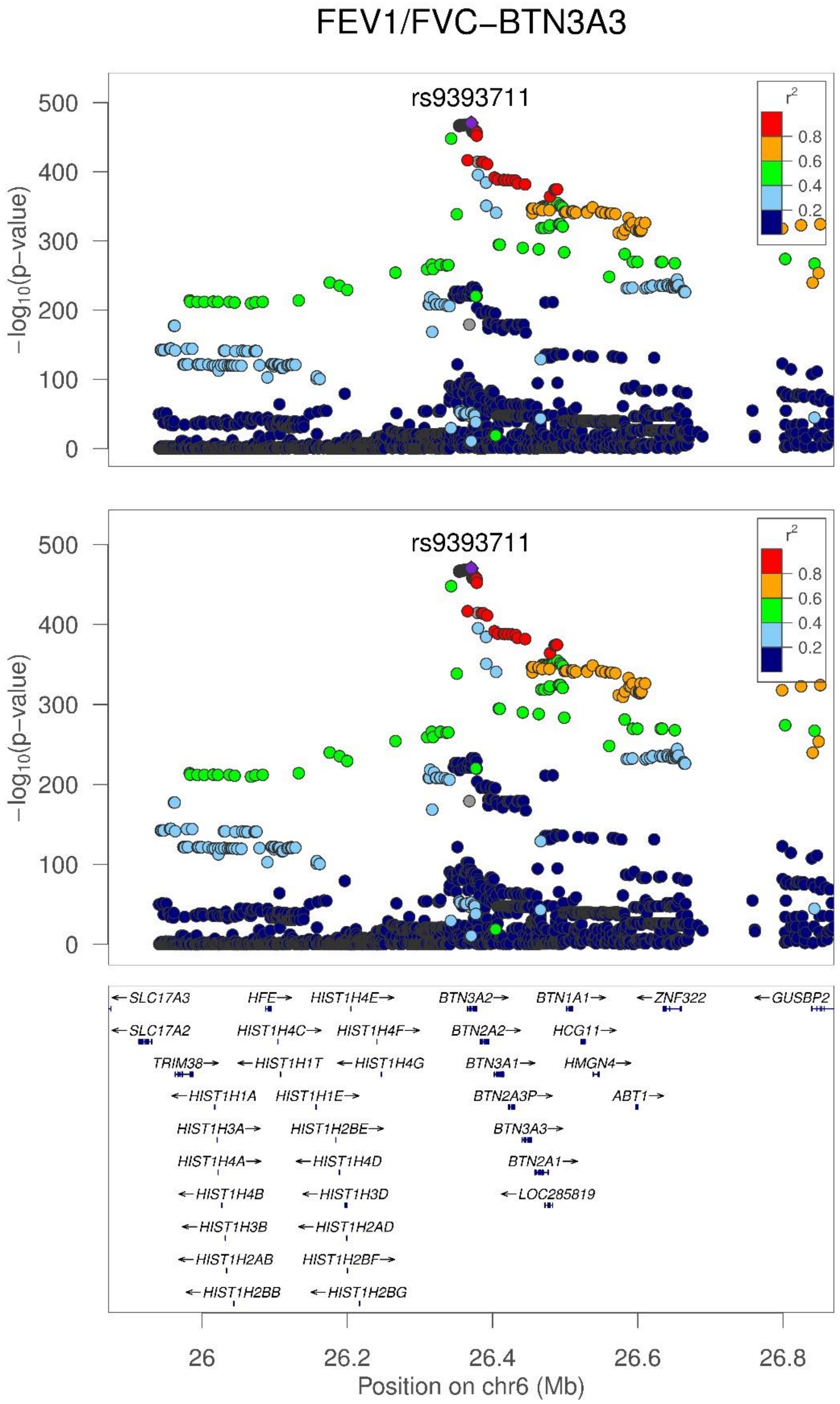

Figure S45: The LocusZoom plot for FEV1/FVC and COL6A3

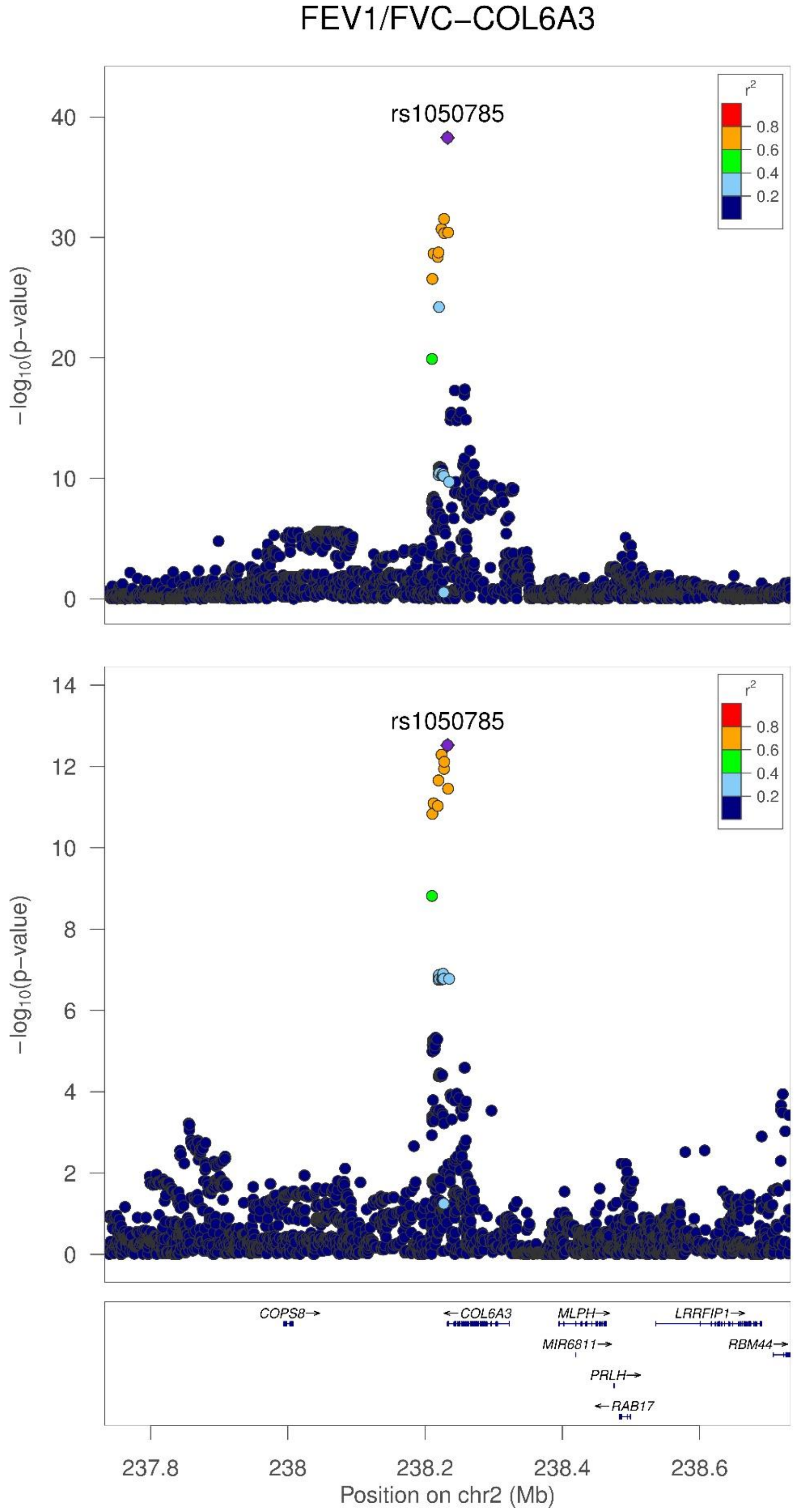

Figure S46: The LocusZoom plot for FEV1/FVC and EFEMP1

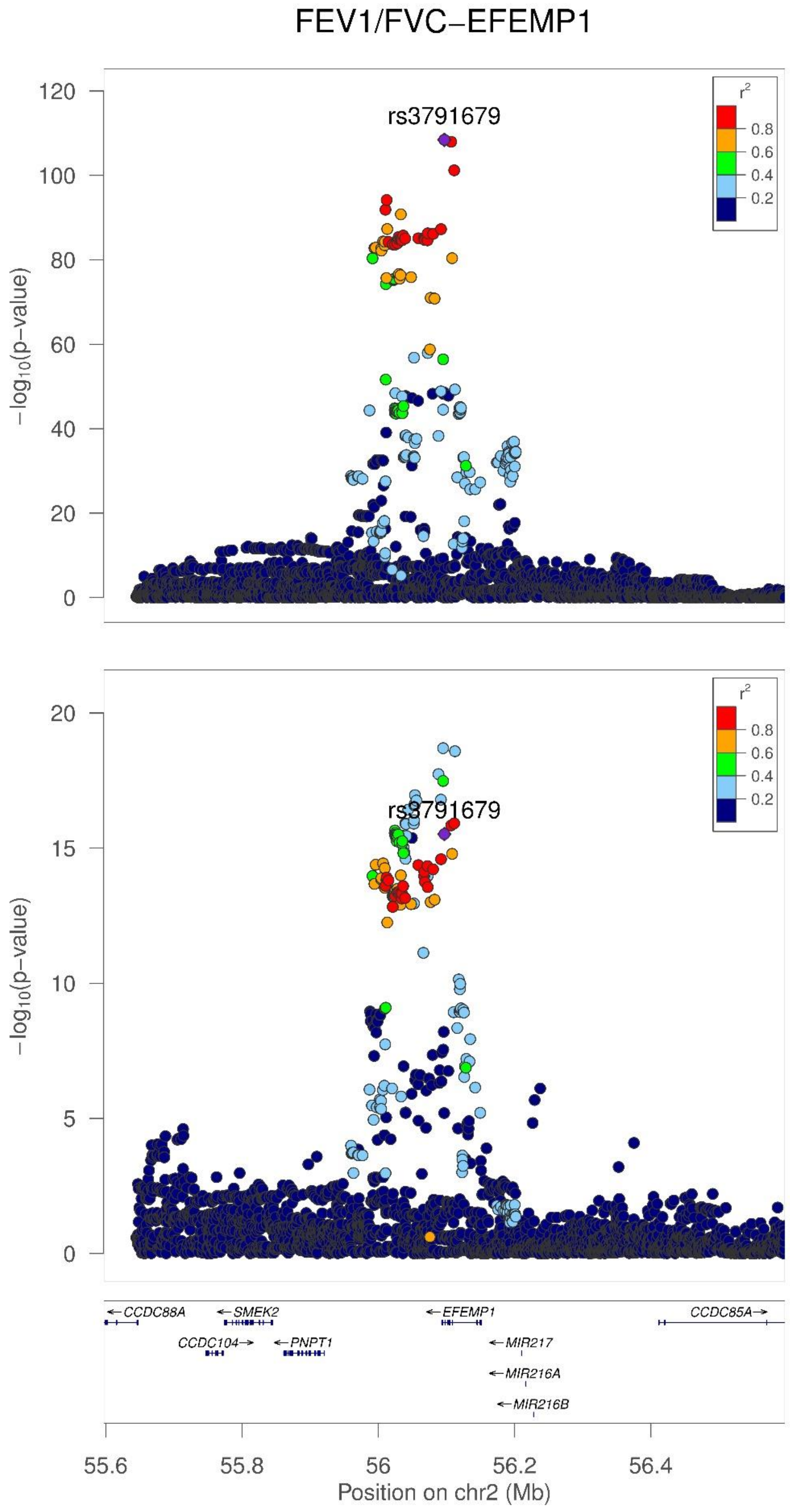

Figure S47: The LocusZoom plot for FEV1/FVC and FAM213A

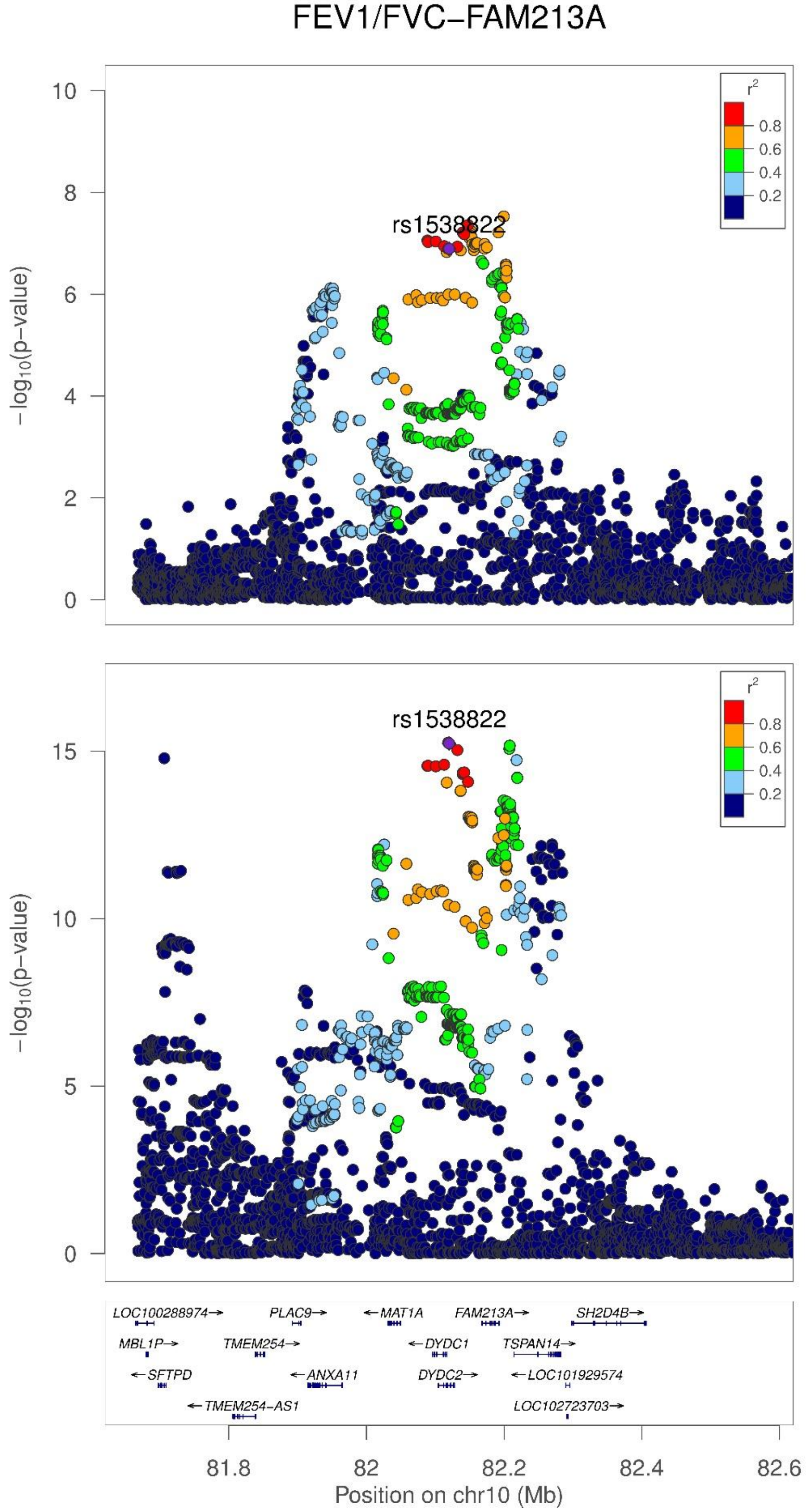

Figure S48: The LocusZoom plot for FEV1/FVC and GM2A

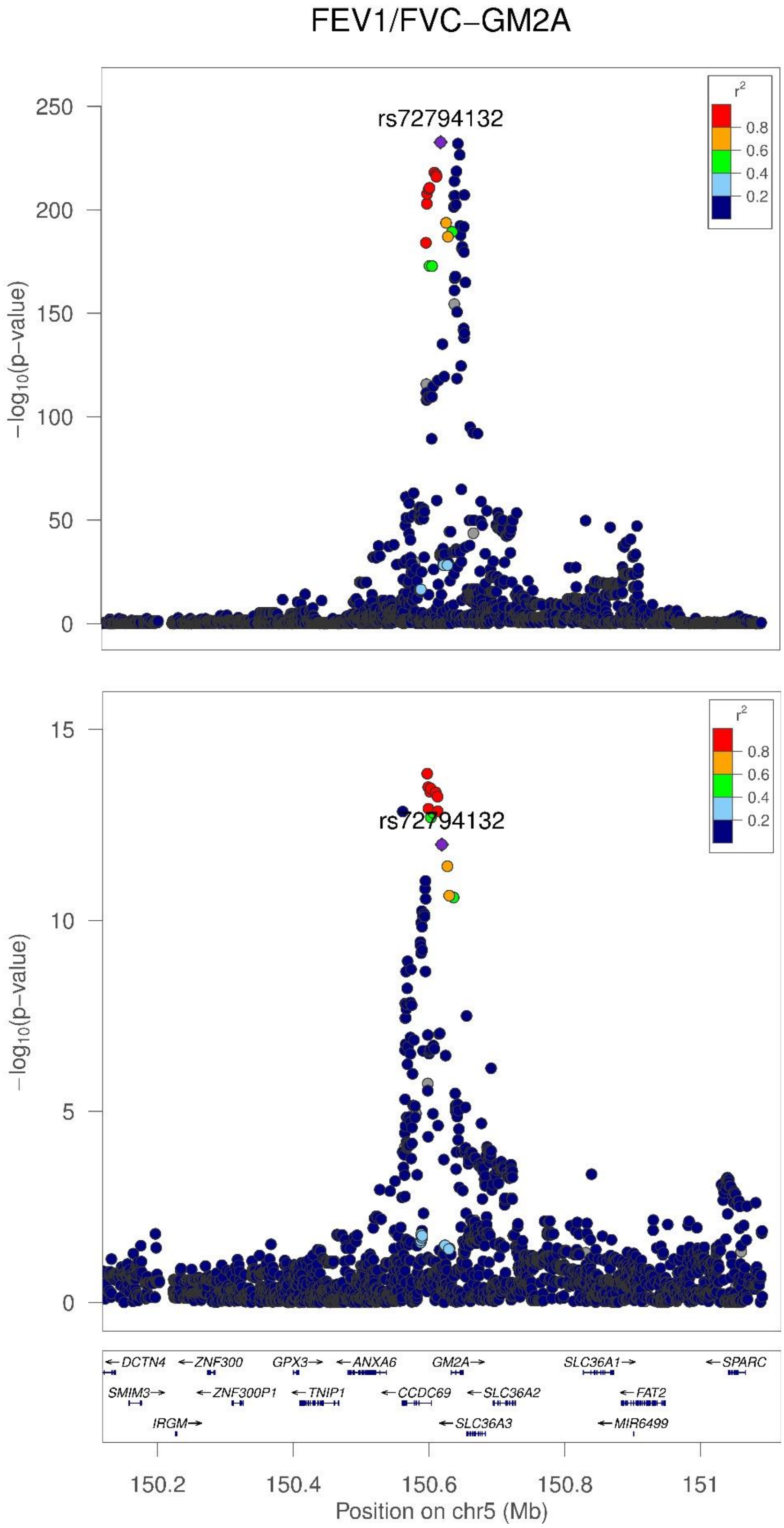

Figure S49: The LocusZoom plot for FEV1/FVC and HP

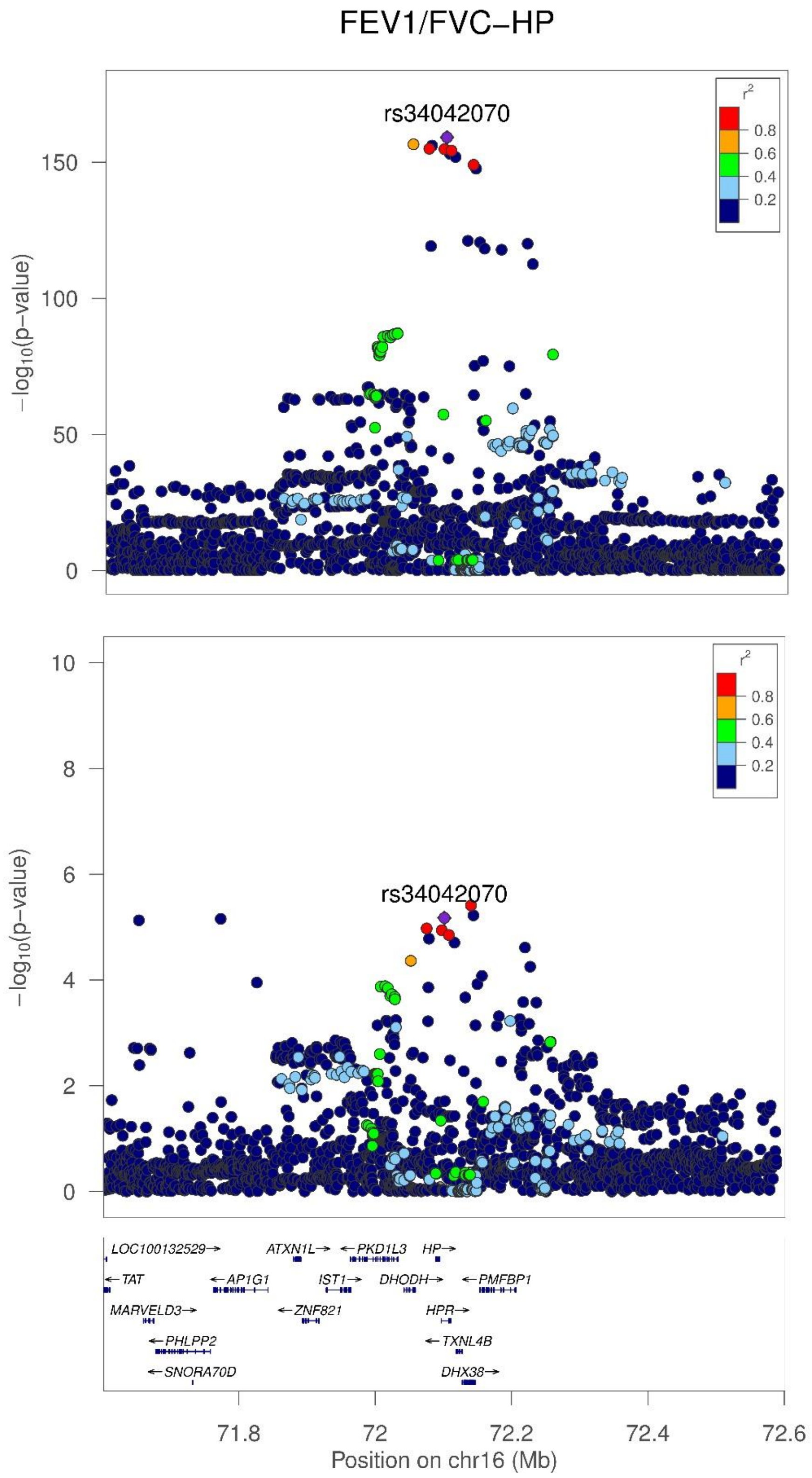

Figure S50: The LocusZoom plot for FEV1/FVC and IL1RL1

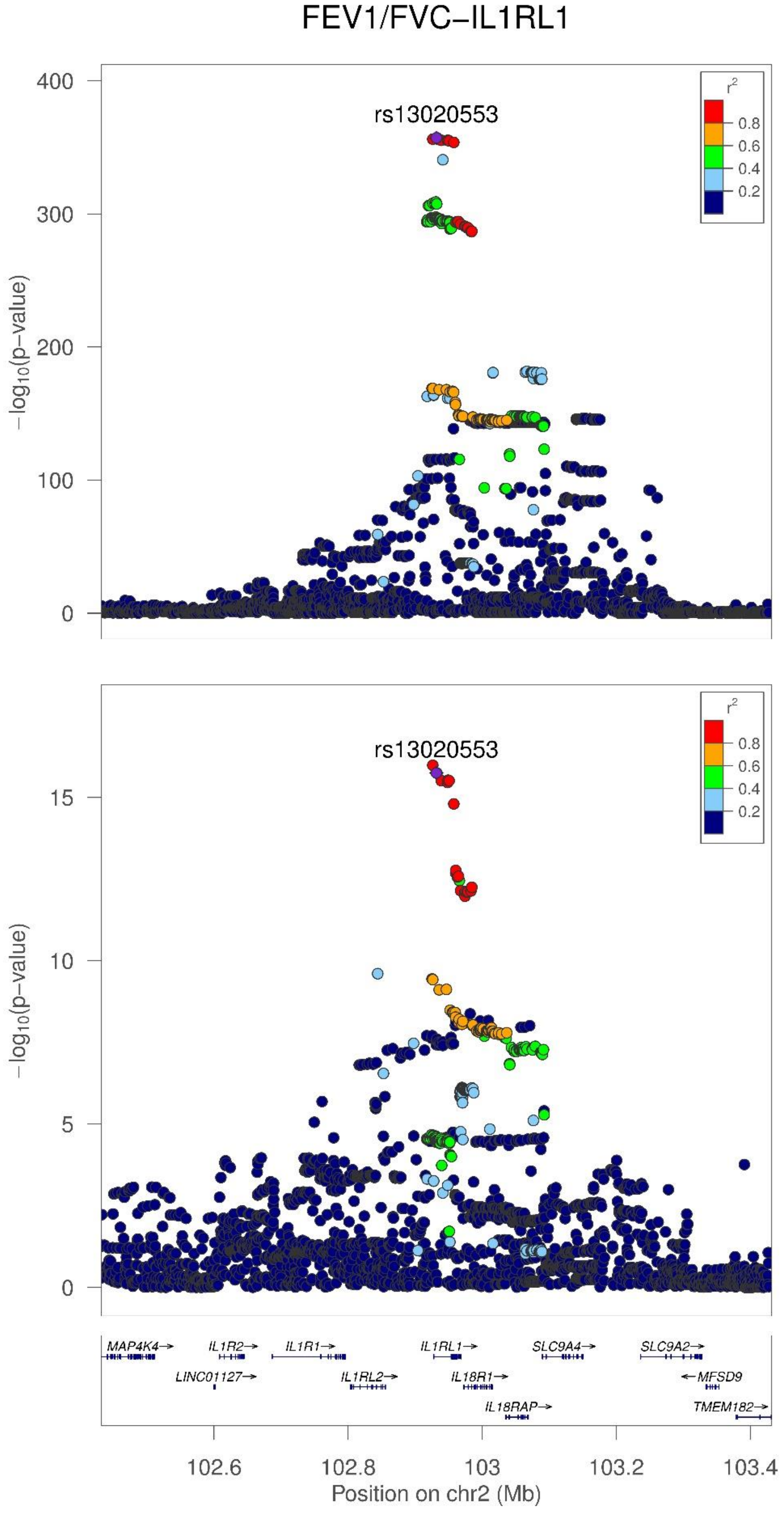

Figure S51: The LocusZoom plot for FEV1/FVC and LTBP4

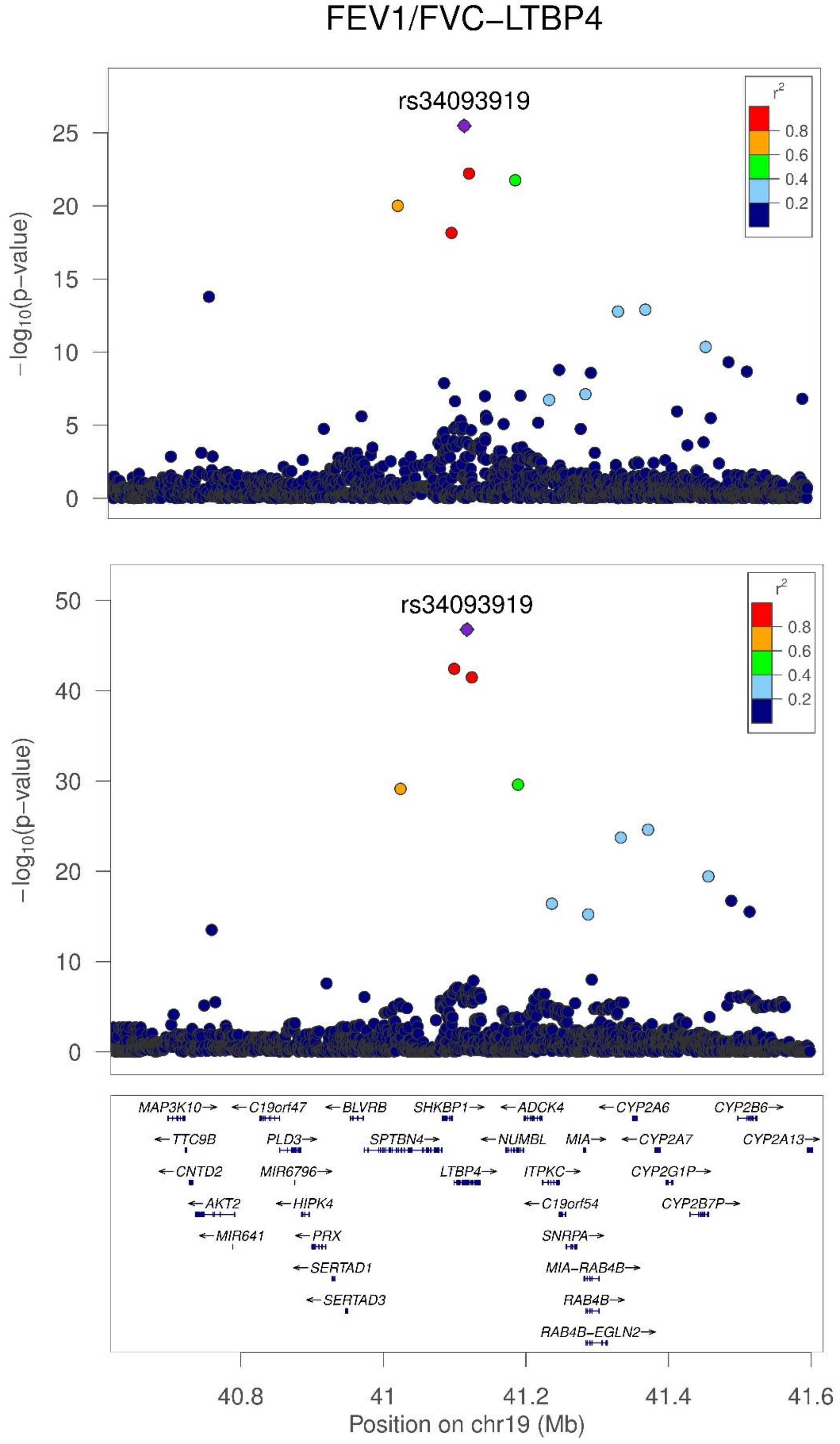

Figure S52: The LocusZoom plot for FEV1/FVC and MANSC4

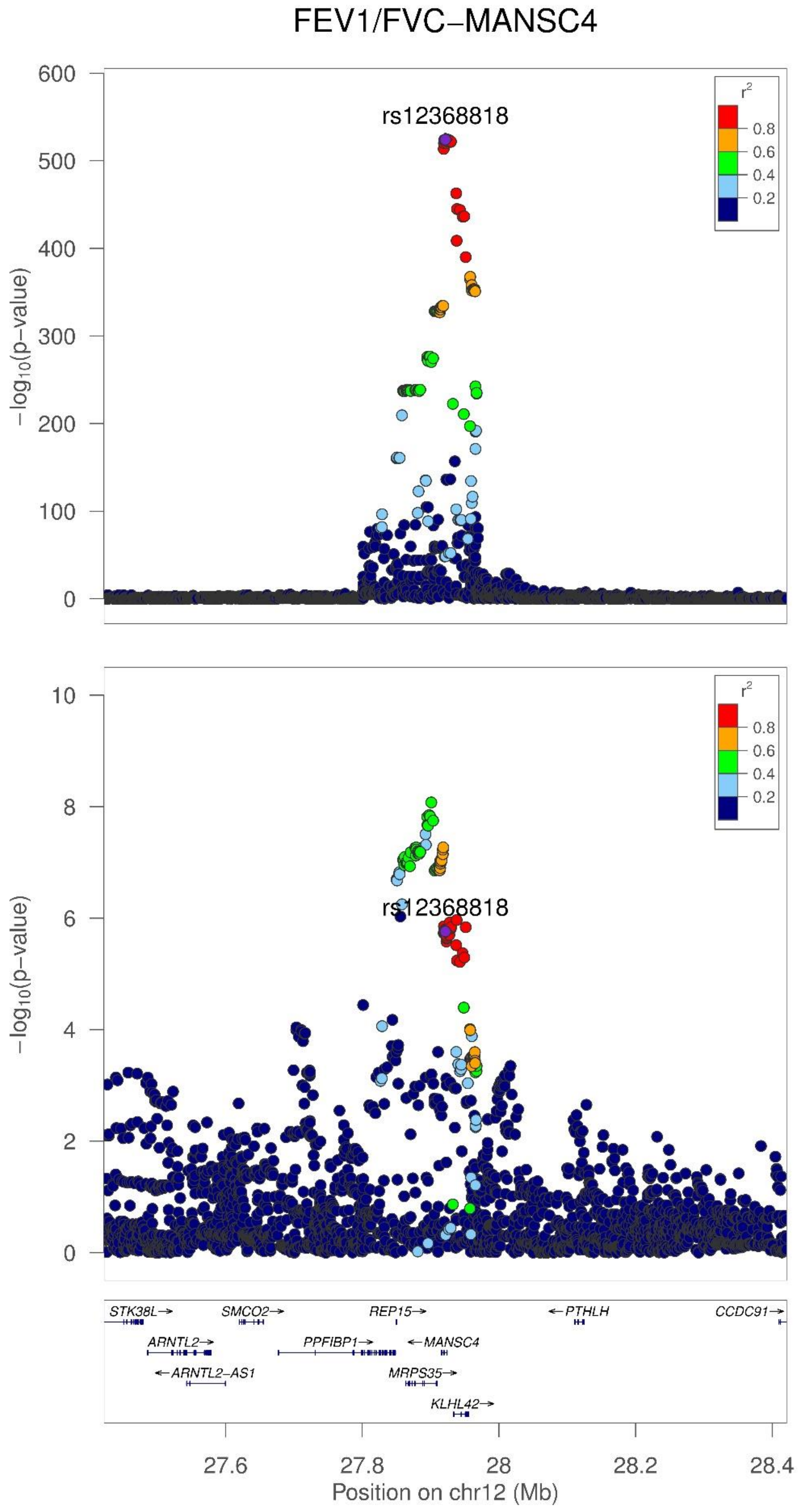

Figure S53: The LocusZoom plot for FEV1/FVC and MAPK3

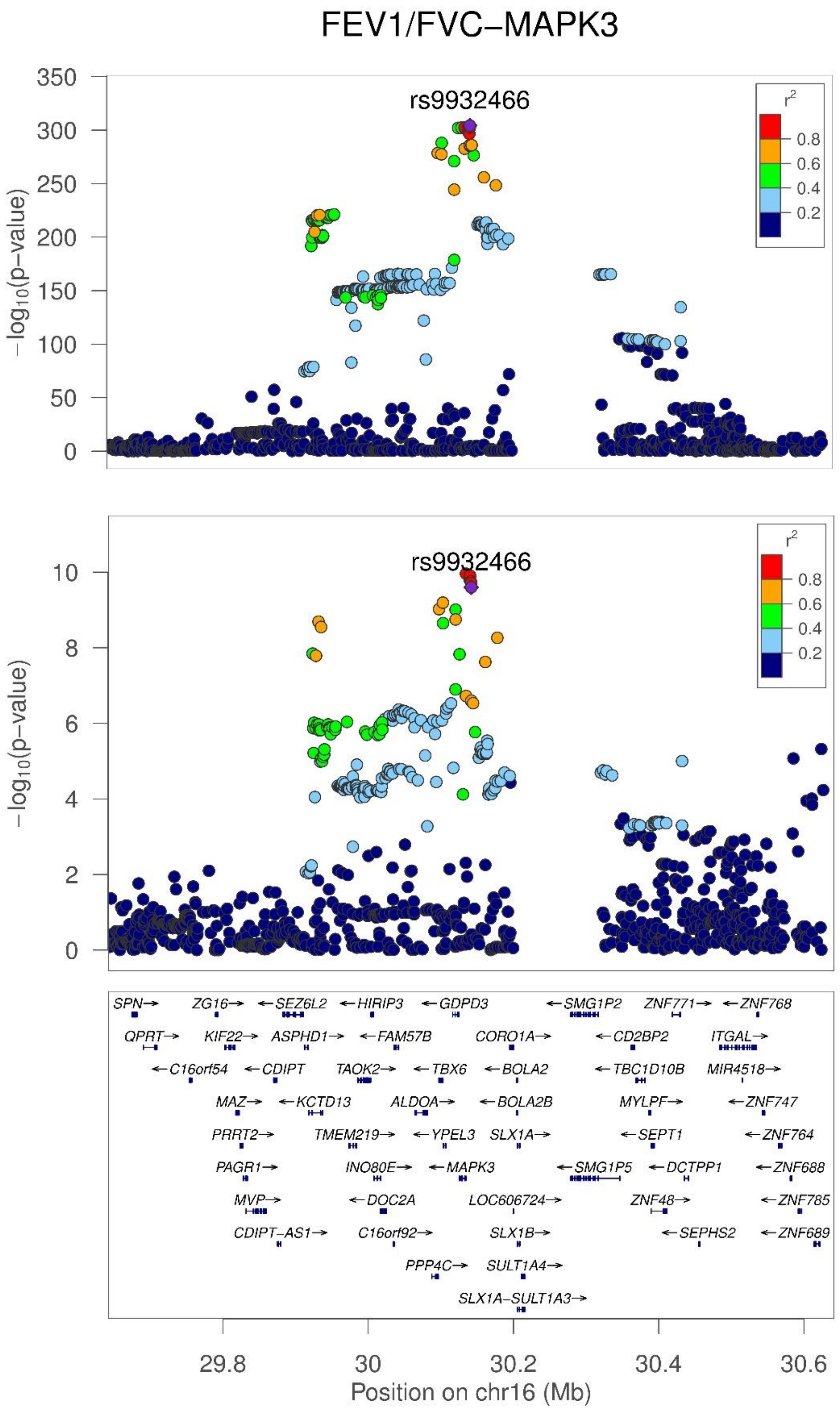

Figure S54: The LocusZoom plot for FEV1/FVC and NID2

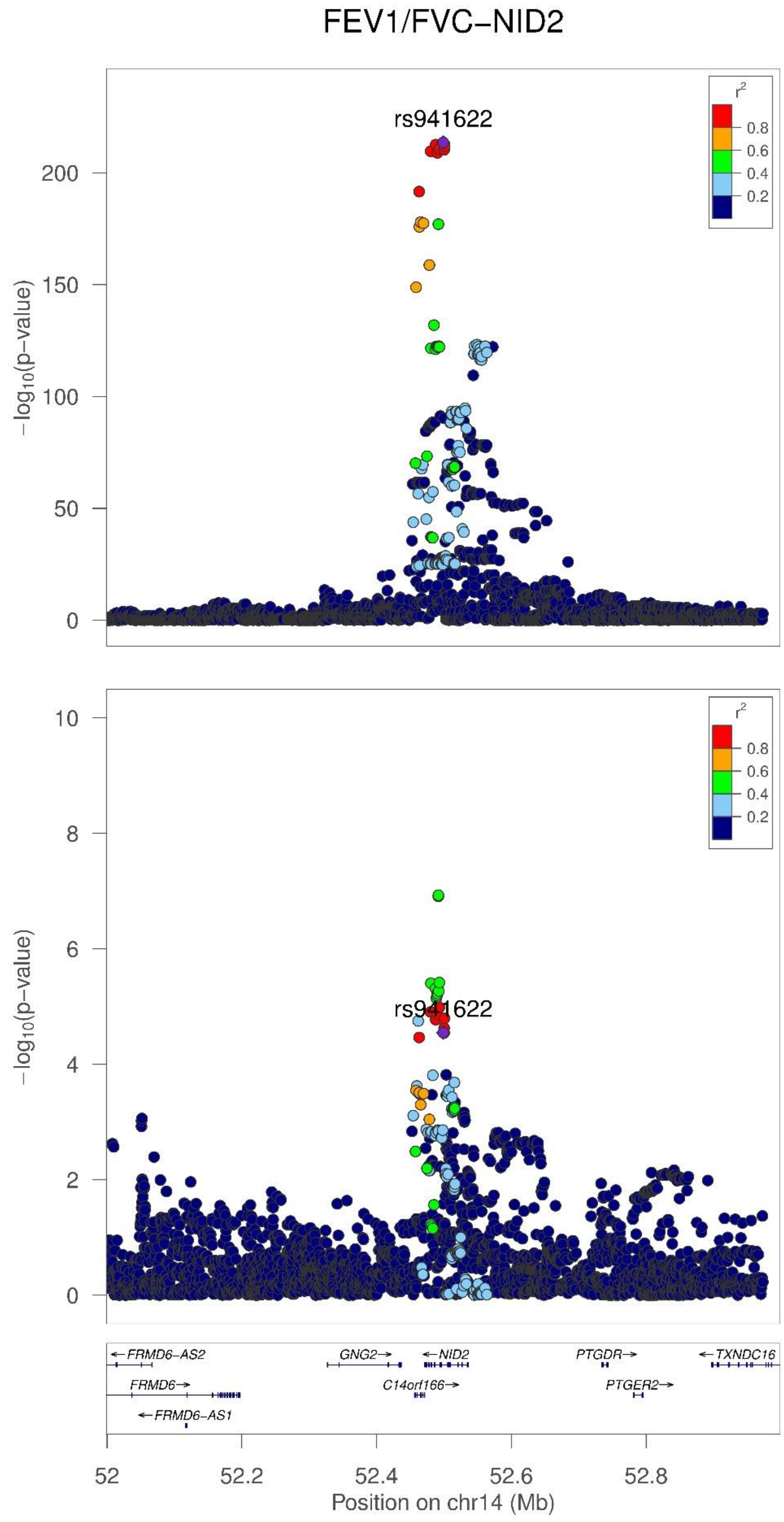

Figure S55: The LocusZoom plot for FEV1/FVC and NOG

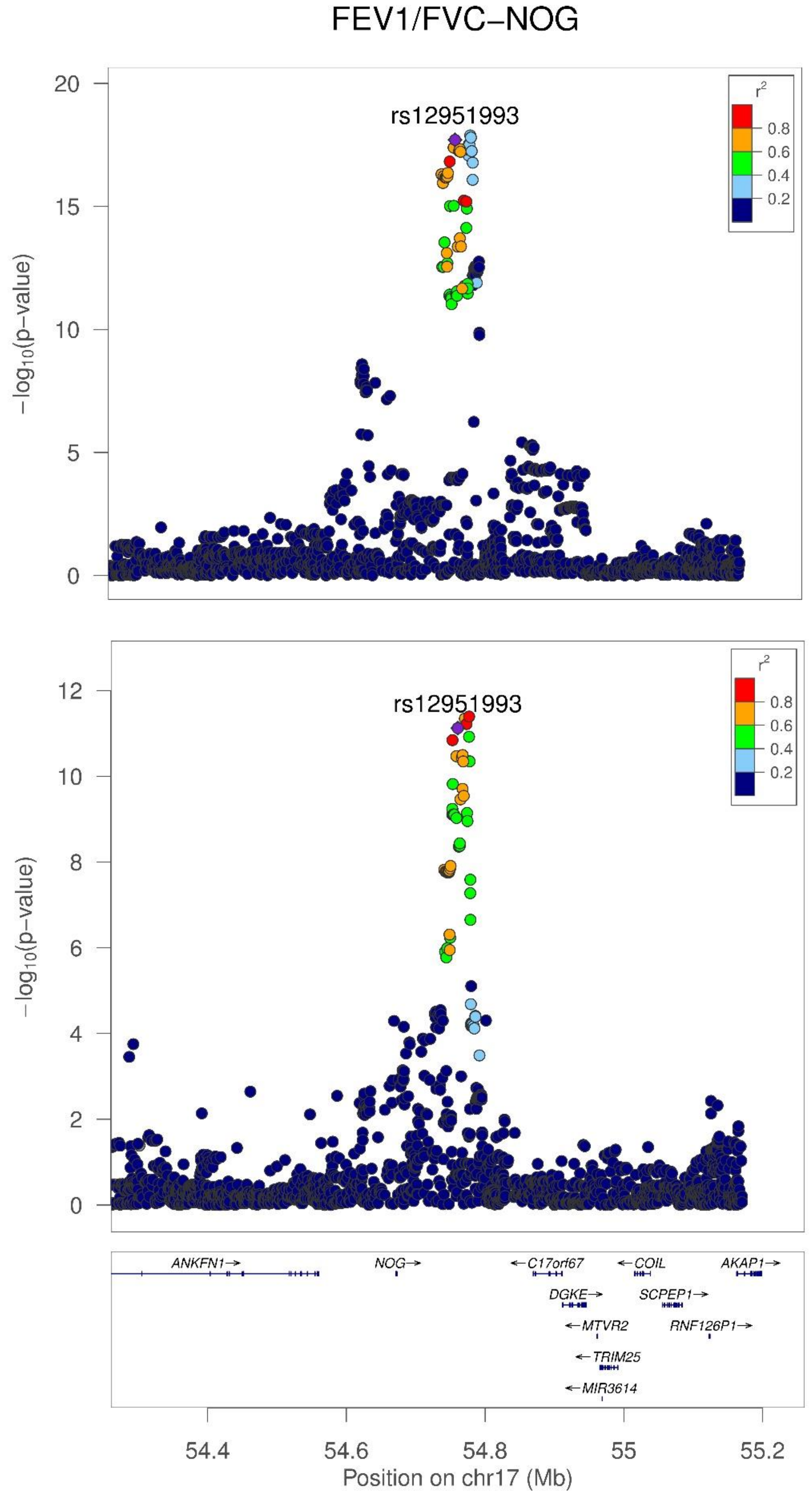

Figure S56: The LocusZoom plot for FEV1/FVC and NPNT

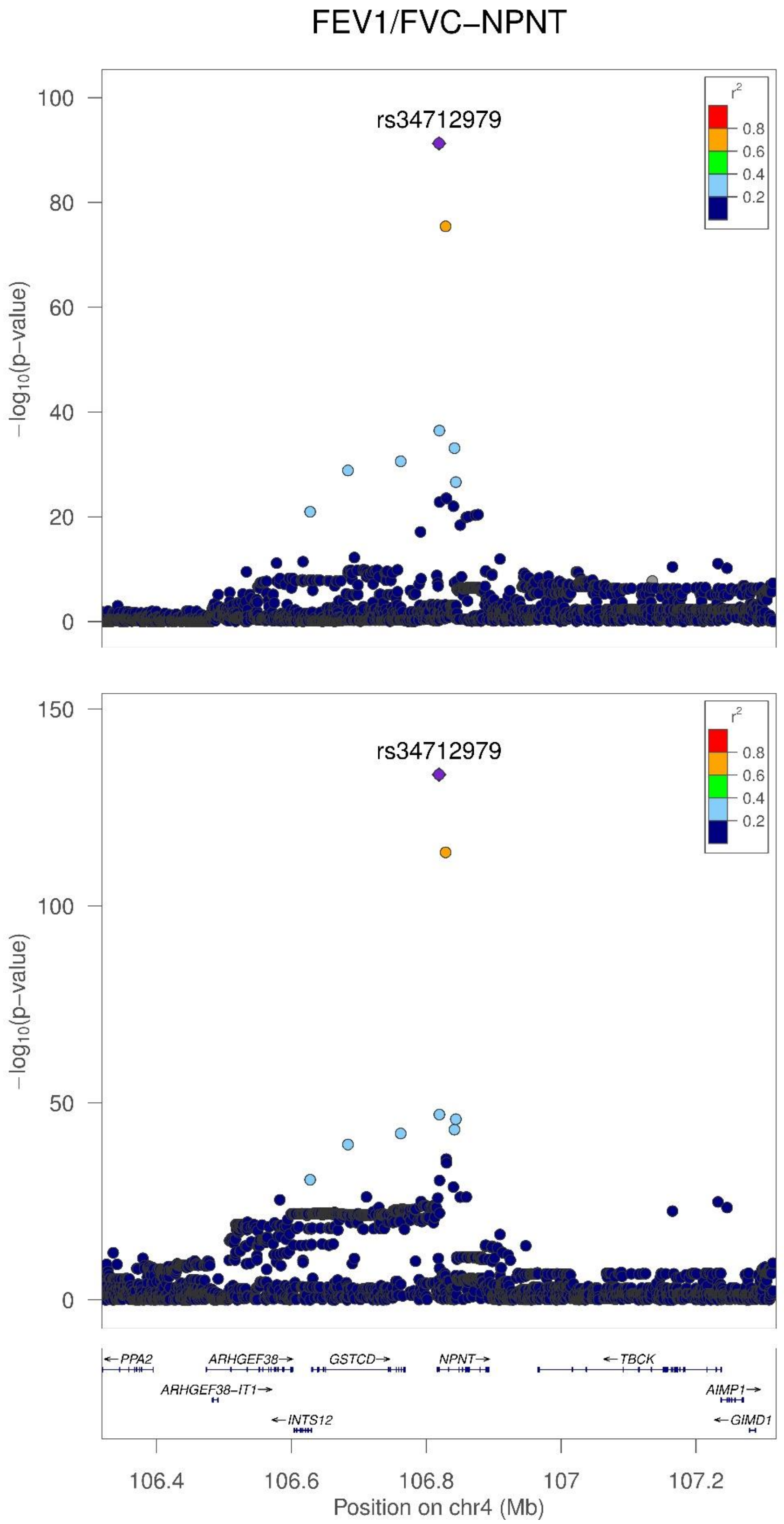

Figure S57: The LocusZoom plot for FEV1/FVC and PAPPA

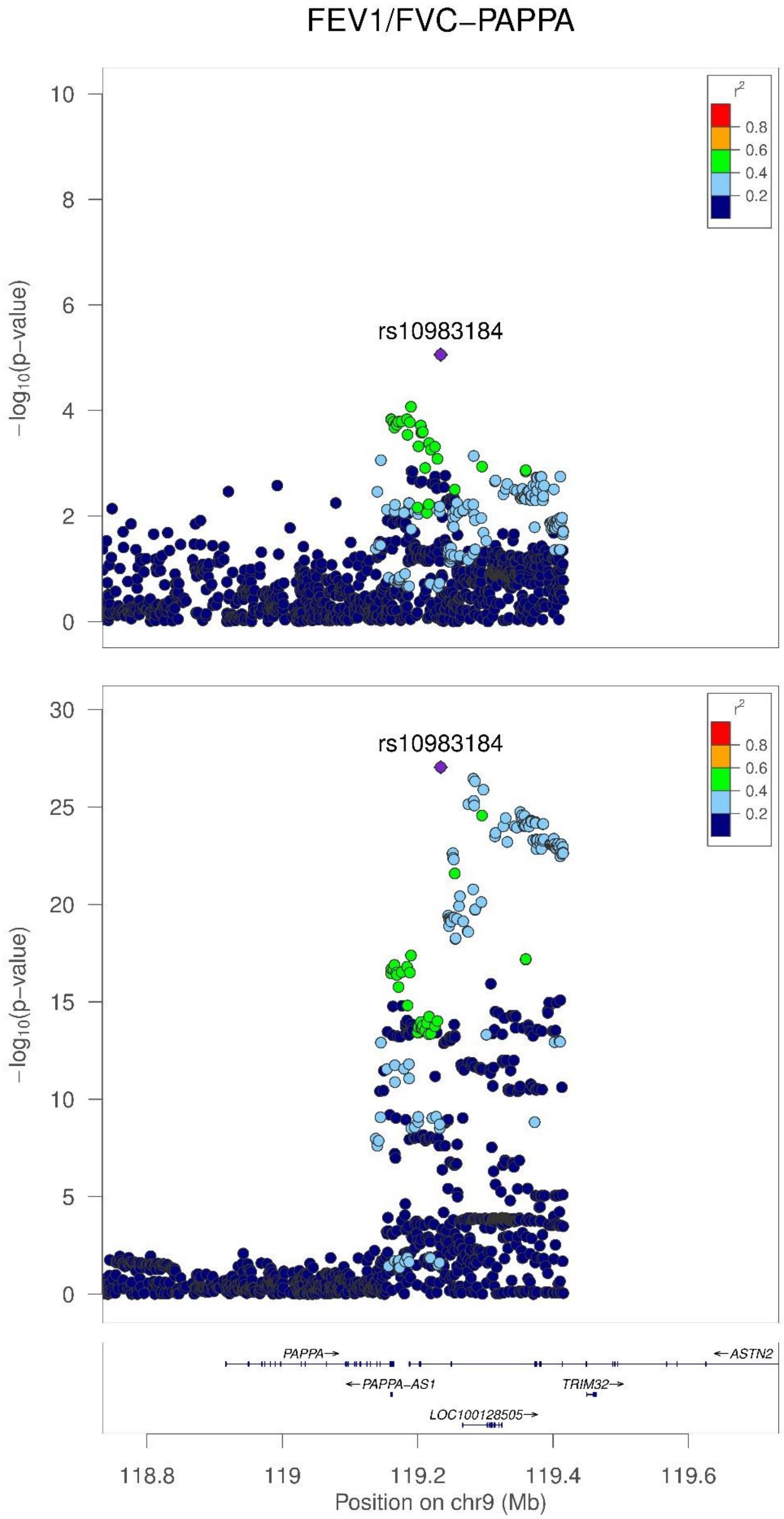

Figure S58: The LocusZoom plot for FEV1/FVC and SCARF2

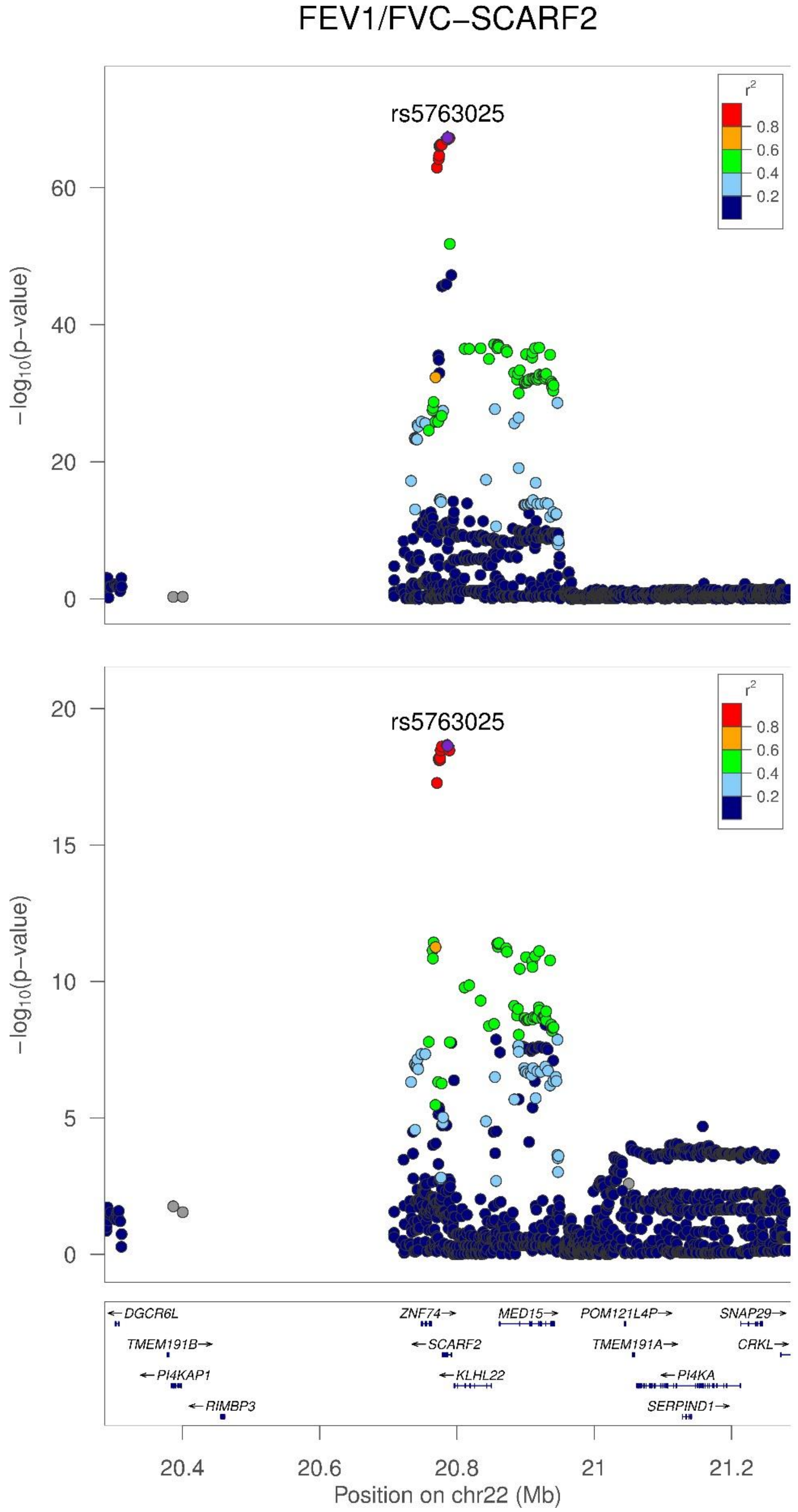

Figure S59: The LocusZoom plot for FEV1/FVC and SERPING1

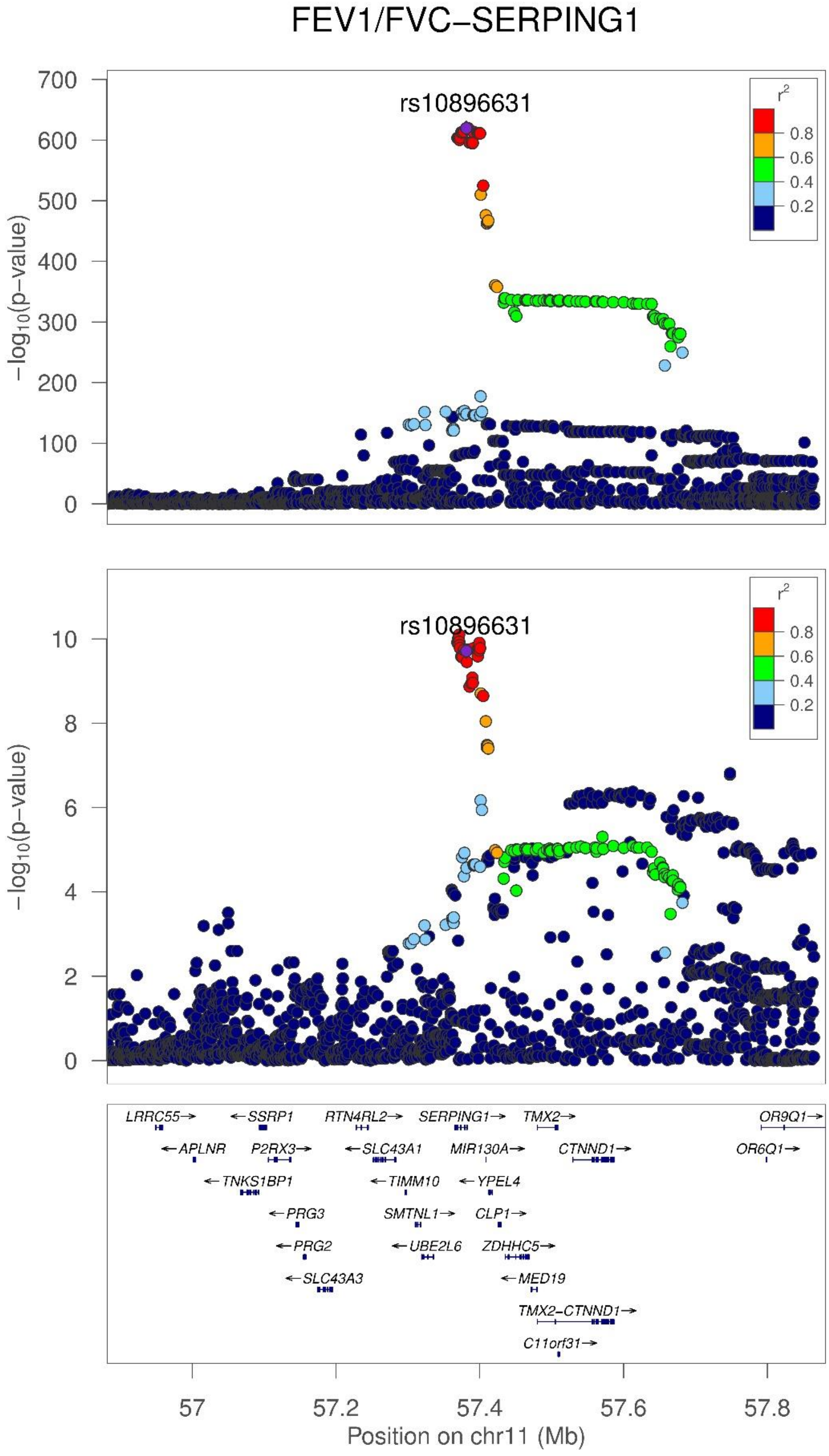

Figure S60: The LocusZoom plot for FEV1/FVC and TMEM2

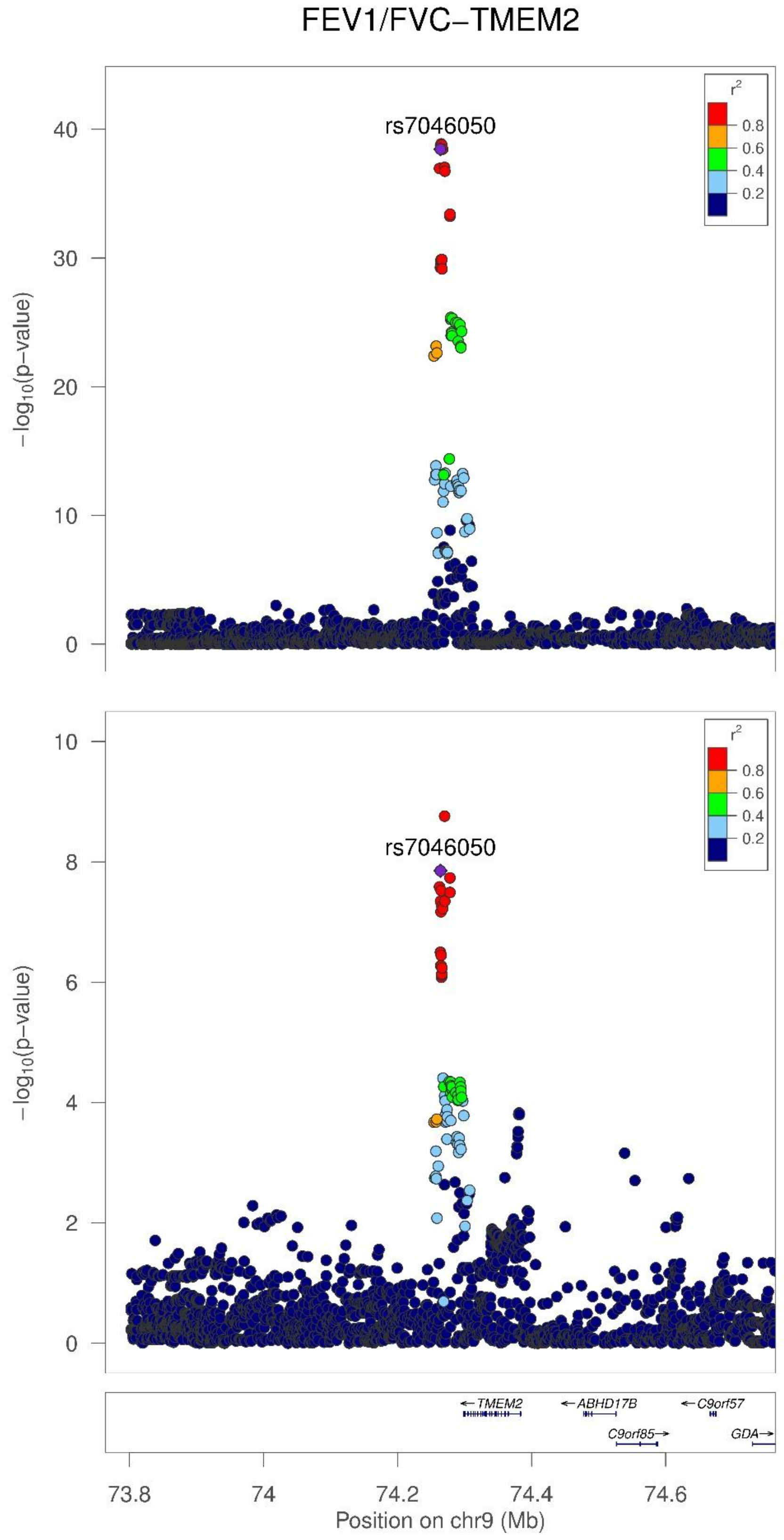

Figure S61: The LocusZoom plot for FEV1/FVC and XPNPEP1

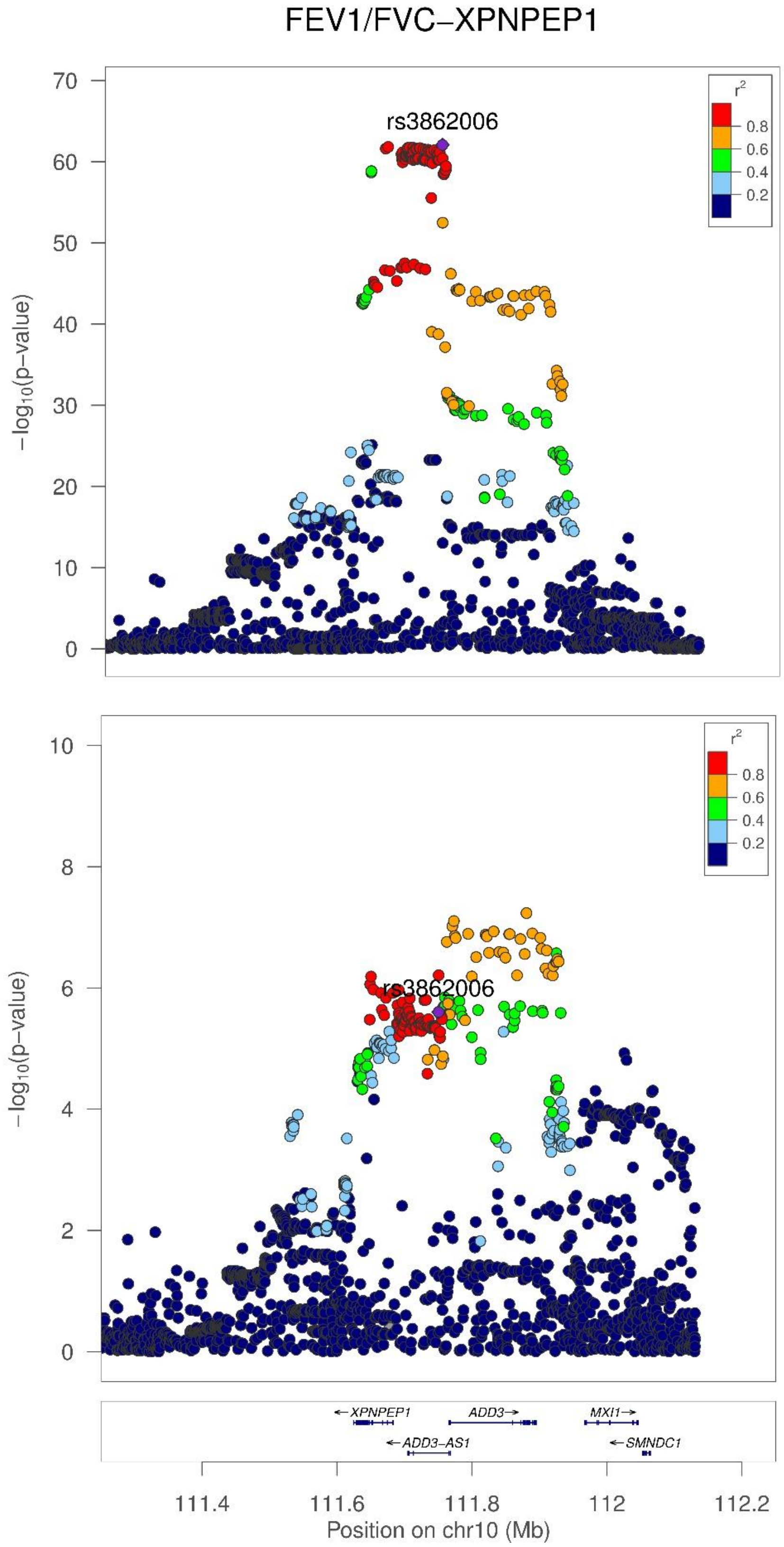

Figure S62: Results of leave-one-out analyses for FEV1 in the discovery MR analyses.

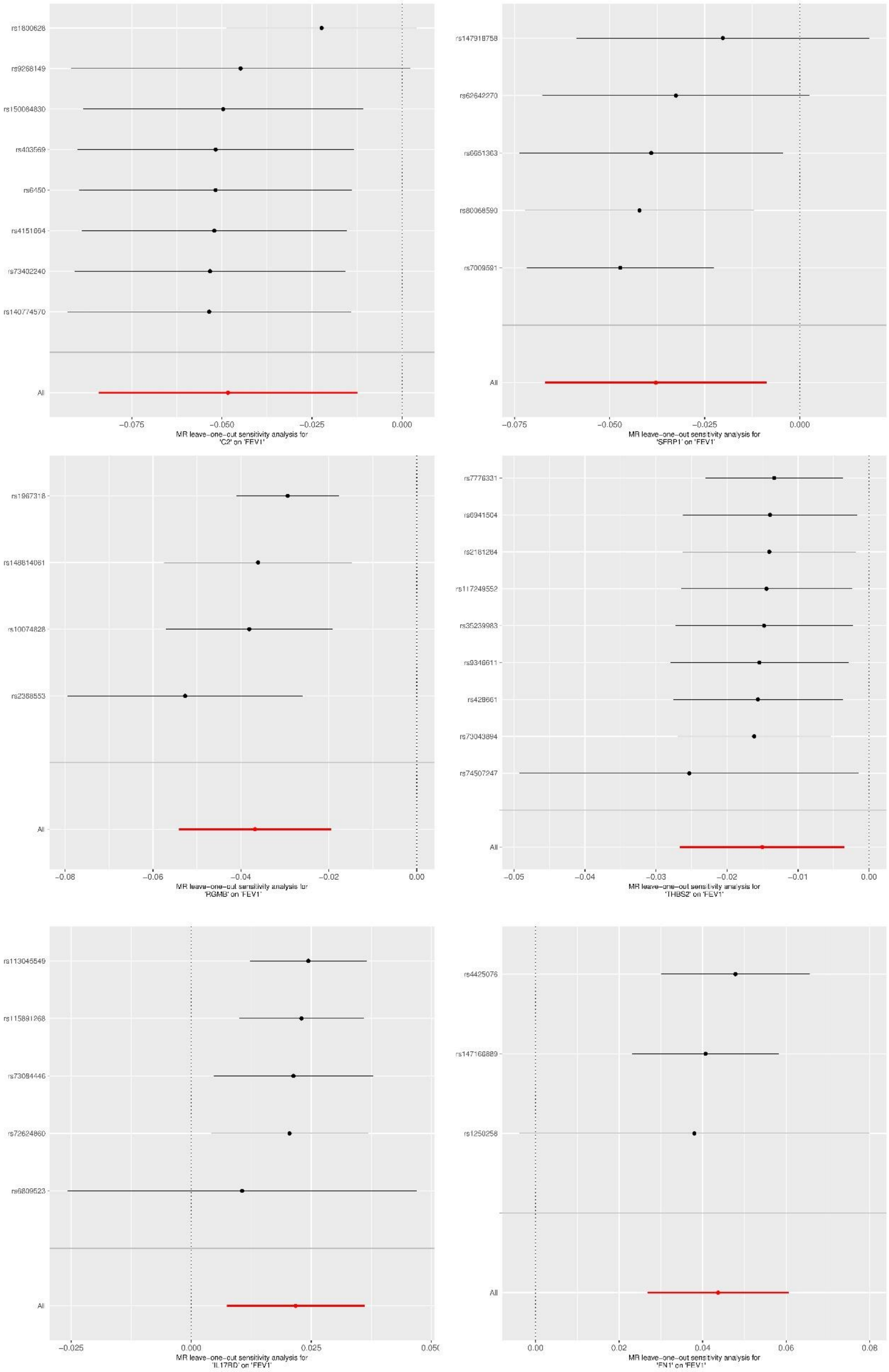

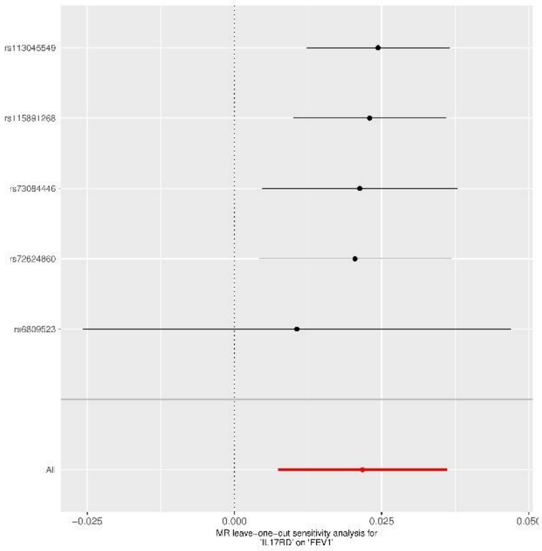
rs113045549
rs115891268
rs73084446
rs72624860
rs6809523
All
-0.025
0.000
0.025
0.050
MR leave-one-out sensitivity analysis for
'IL17RD' on 'FEV1'

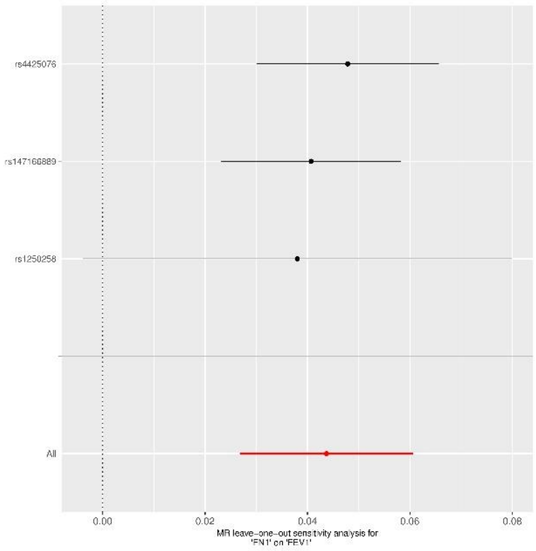
rs4425076
rs147166889
rs1250258
All
0.00
0.02
0.04
0.06
0.08
MR leave-one-out sensitivity analysis for
'FN1' on 'FEV1'

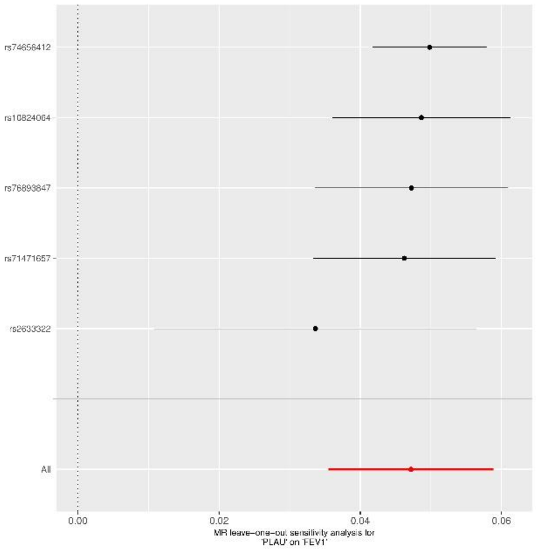
rs74656412
rs10824064
rs76893847
rs71471657
rs2633322
All
0.00
0.02
0.04
0.06
MR leave-one-out sensitivity analysis for
'PLAU' on 'FEV1'

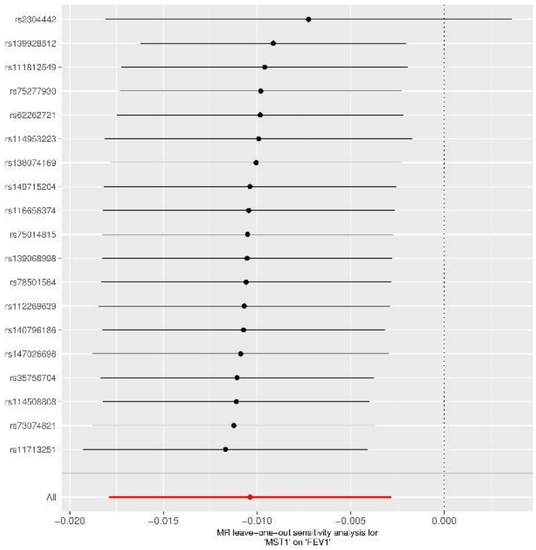
rs2304442
rs139928512
rs111812549
rs75277930
rs62262721
rs114953223
rs138074169
rs149715204
rs116656374
rs75014815
rs139068998
rs78501564
rs112269639
rs140796186
rs147026698
rs35756704
rs114508808
rs73074821
rs11713251
All
-0.020
-0.015
-0.010
-0.005
0.000
MR leave-one-out sensitivity analysis for
'MST1' on 'FEV1'

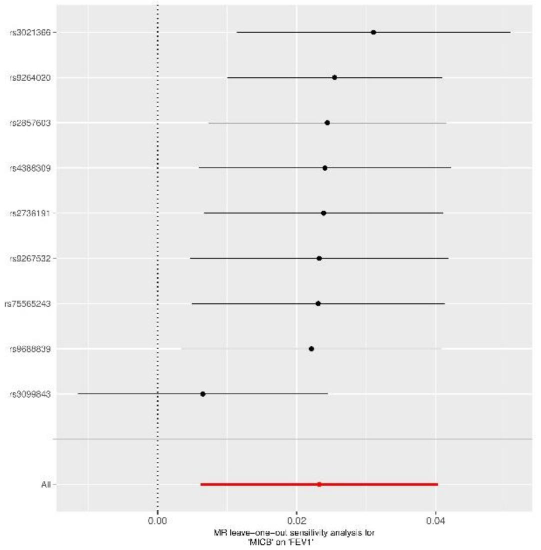
rs3021366
rs9264020
rs2857603
rs4388309
rs2736191
rs9267532
rs75565243
rs9688839
rs3099843
All
0.00
0.02
0.04
MR leave-one-out sensitivity analysis for
'MICB' on 'FEV1'

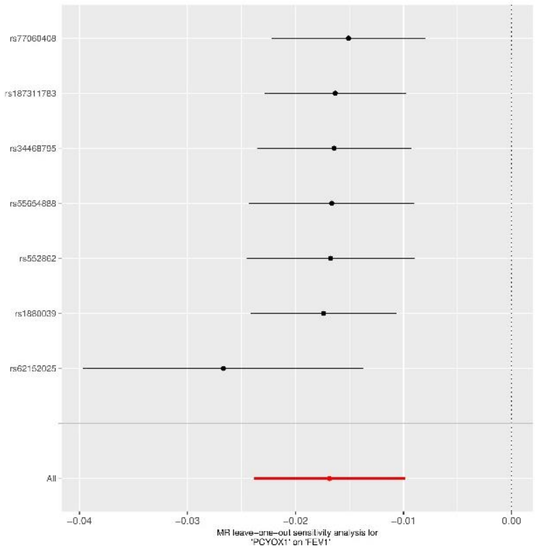
rs77060408
rs187311783
rs34468795
rs55654888
rs552862
rs1880039
rs62152025
All
-0.04
-0.03
-0.02
-0.01
0.00
MR leave-one-out sensitivity analysis for
'PCYOX1' on 'FEV1'

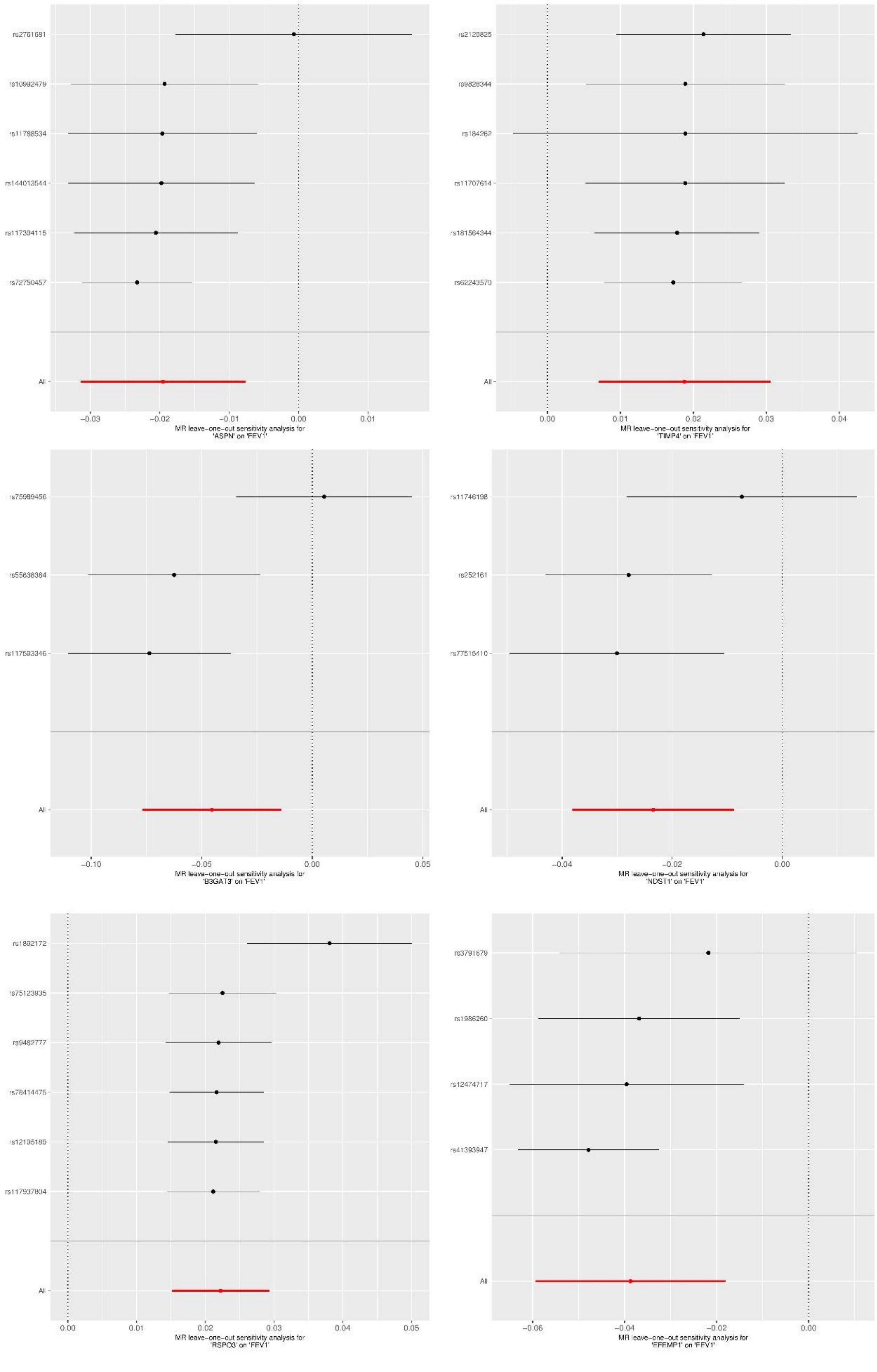
MR leave-one-out sensitivity analysis for 'ASPN' on 'FEV1'
MR leave-one-out sensitivity analysis for 'TIMP4' on 'FEV1'
MR leave-one-out sensitivity analysis for 'B3GAT3' on 'FEV1'
MR leave-one-out sensitivity analysis for 'NDST1' on 'FEV1'
MR leave-one-out sensitivity analysis for 'RSPO3' on 'FEV1'
MR leave-one-out sensitivity analysis for 'EFEMP1' on 'FEV1'

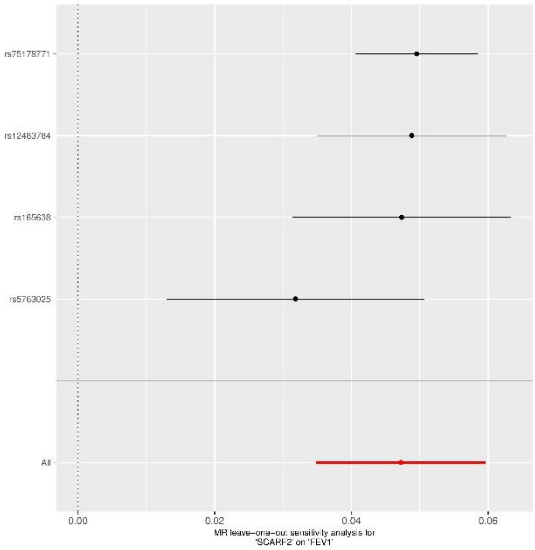
rs75178771
rs12483784
rs165638
rs5763025
All
0.00
0.02
0.04
0.06
MR leave-one-out sensitivity analysis for
'SCARF2' on 'FEV1'

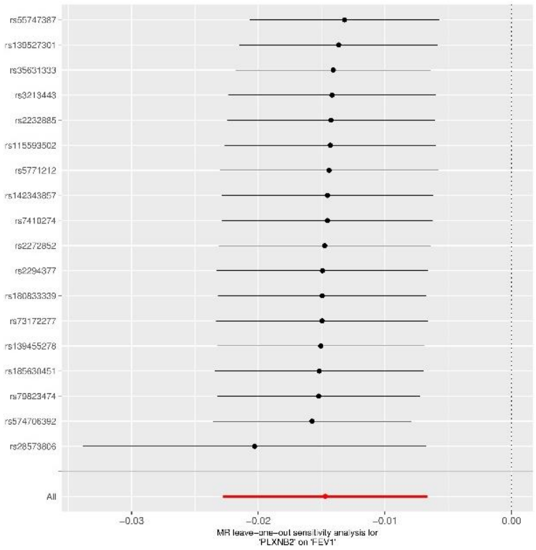
rs55747387
rs139527301
rs35631333
rs3213443
rs2232885
rs115593502
rs5771212
rs142343857
rs7410274
rs2272852
rs2294377
rs180833339
rs73172277
rs139455278
rs185630451
rs79823474
rs574706392
rs28573806
All
−0.03
−0.02
−0.01
0.00
MR leave-one-out sensitivity analysis for
'PLXNB2' on 'FEV1'

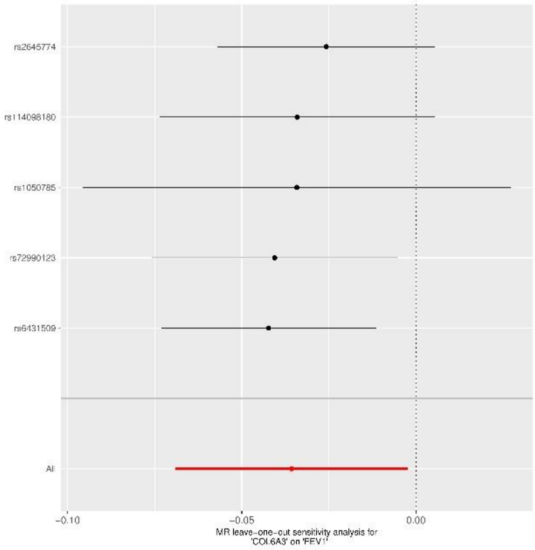
rs2645774
rs114098180
rs1050785
rs72990123
rs6431509
All
−0.10
−0.05
0.00
MR leave-one-out sensitivity analysis for
'COL6A3' on 'FEV1'

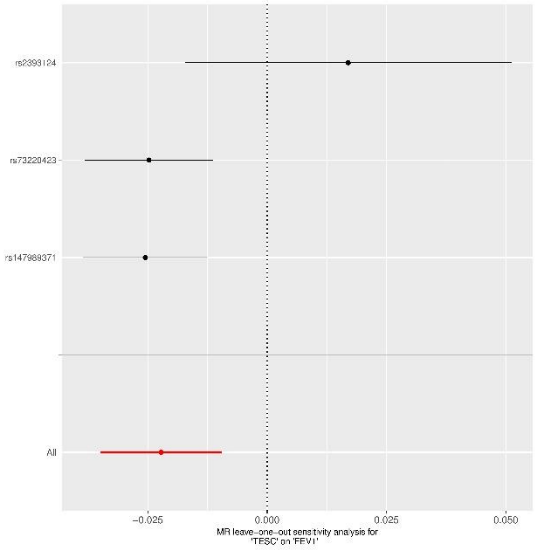
rs2393124
rs73220423
rs147989371
All
−0.025
0.000
0.025
0.050
MR leave-one-out sensitivity analysis for
'TESC' on 'FEV1'

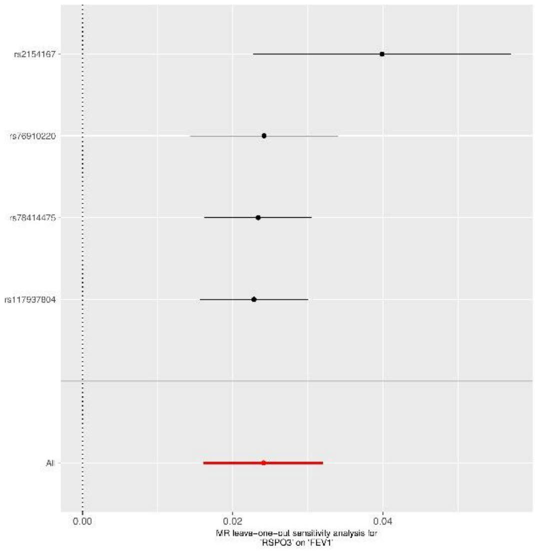
rs2154167
rs76910220
rs78414475
rs117937804
All
0.00
0.02
0.04
MR leave-one-out sensitivity analysis for
'RSPO3' on 'FEV1'

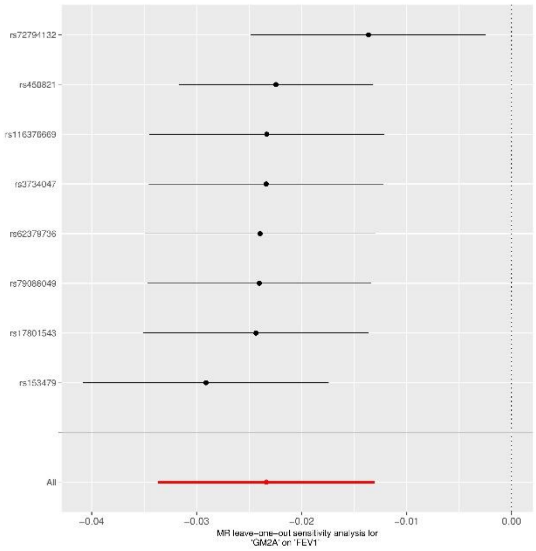
rs72794132
rs450821
rs116376669
rs3734047
rs62379736
rs79086049
rs17801543
rs153479
All
−0.04
−0.03
−0.02
−0.01
0.00
MR leave-one-out sensitivity analysis for
'GM2A' on 'FEV1'

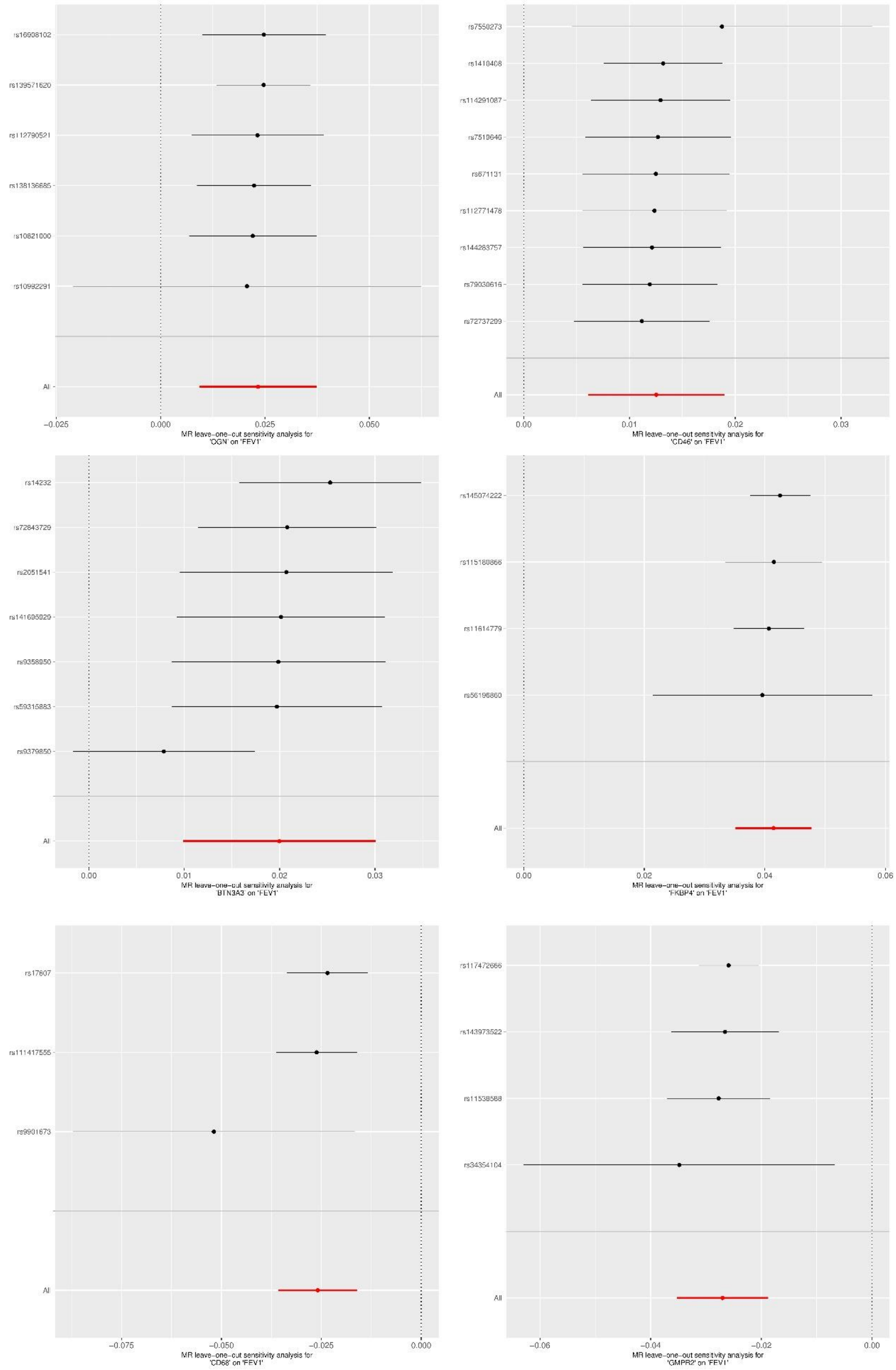

rs16908102
rs139571620
rs112790521
rs138136685
rs10821000
rs10992291
All
−0.025
0.000
0.025
0.050
MR leave−one−out sensitivity analysis for
'OGN' on 'FEV1'
rs7550273
rs1410408
rs114291087
rs7519646
rs671131
rs112771478
rs144283757
rs79030616
rs72737299
All
0.00
0.01
0.02
0.03
MR leave−one−out sensitivity analysis for
'CD46' on 'FEV1'
rs14232
rs72843729
rs2051541
rs141695029
rs9358950
rs59315883
rs9379850
All
0.00
0.01
0.02
0.03
MR leave−one−out sensitivity analysis for
'BTN3A3' on 'FEV1'
rs145074222
rs115180866
rs11614779
rs56196860
All
0.00
0.02
0.04
0.06
MR leave−one−out sensitivity analysis for
'FKBP4' on 'FEV1'
rs17607
rs111417555
rs9901673
All
−0.075
−0.050
−0.025
0.000
MR leave−one−out sensitivity analysis for
'CD68' on 'FEV1'
rs117472666
rs143973522
rs11538588
rs34354104
All
−0.06
−0.04
−0.02
0.00
MR leave−one−out sensitivity analysis for
'GMPR2' on 'FEV1'

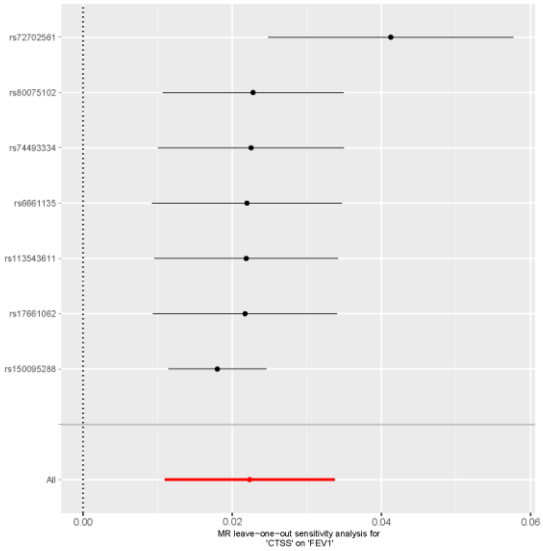
rs72702561
rs80075102
rs74493334
rs6661135
rs113543611
rs17661062
rs150095288
All
0.00
0.02
0.04
0.06
MR leave−one−out sensitivity analysis for
'CTSS' on 'FEV1'

Figure S63: Results of leave-one-out analyses for FVC in the discovery MR analyses.

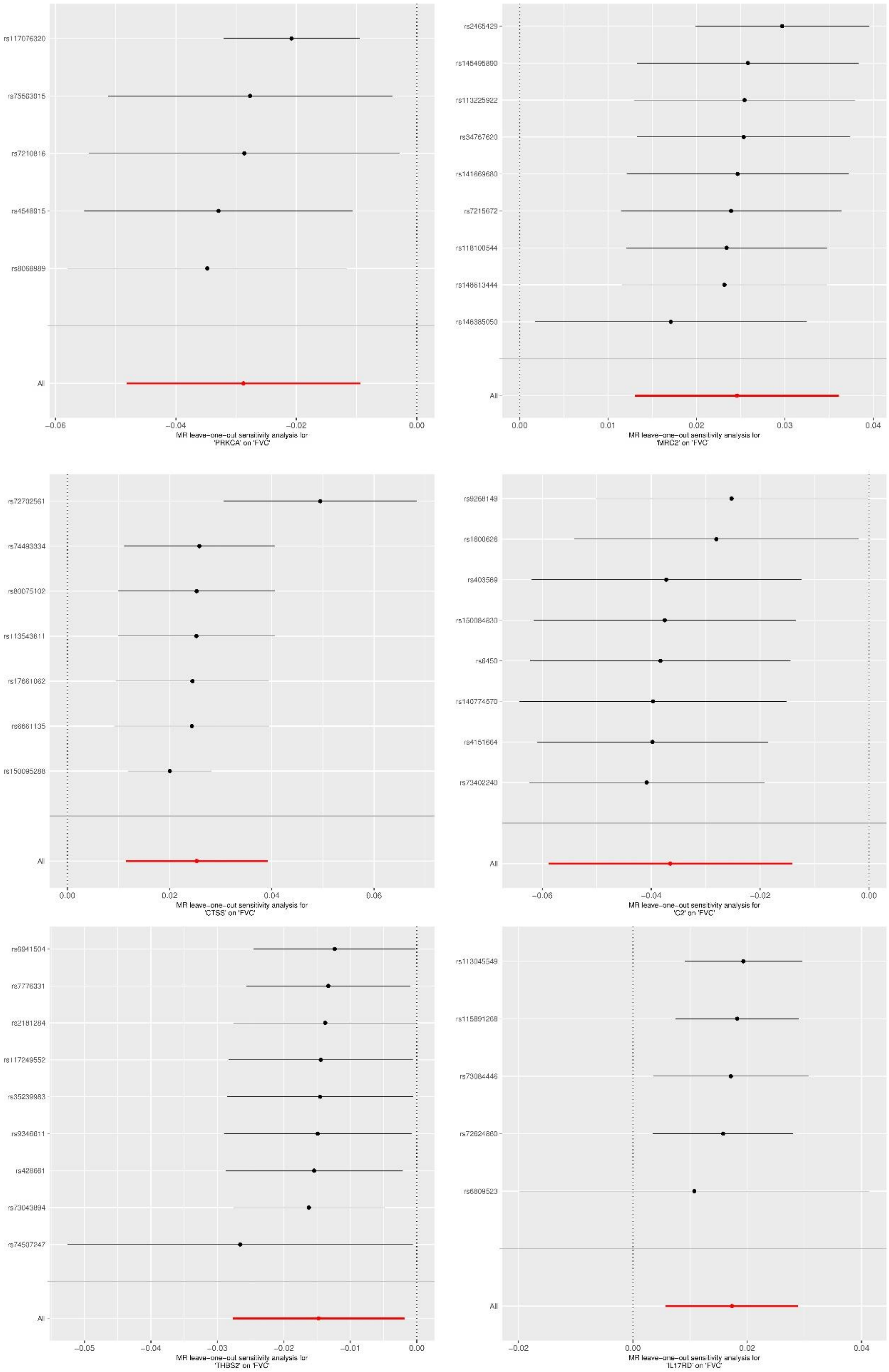

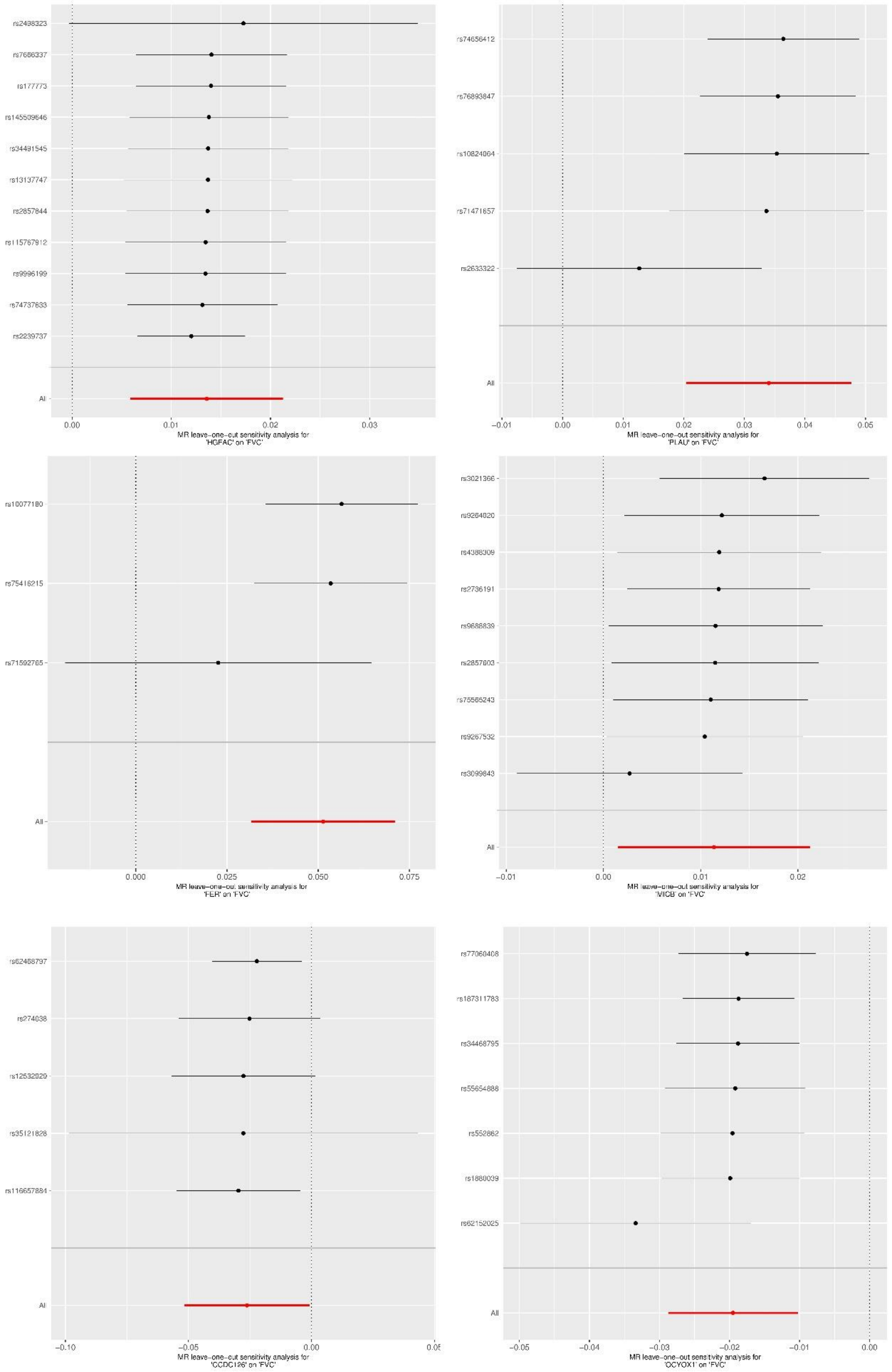
MR leave−one−out sensitivity analysis for 'HGFAC' on 'FVC'
MR leave−one−out sensitivity analysis for 'PLAU' on 'FVC'
MR leave−one−out sensitivity analysis for 'FER' on 'FVC'
MR leave−one−out sensitivity analysis for 'MICB' on 'FVC'
MR leave−one−out sensitivity analysis for 'CCDC126' on 'FVC'
MR leave−one−out sensitivity analysis for 'OCIAD1' on 'FVC'

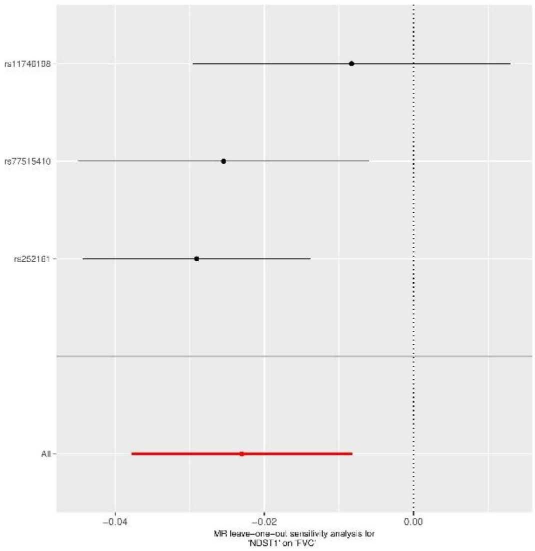
rs11740198
rs77515410
rs252161
All
−0.04
−0.02
0.00
MR leave−one−out sensitivity analysis for
'NDST1' on 'FVC'

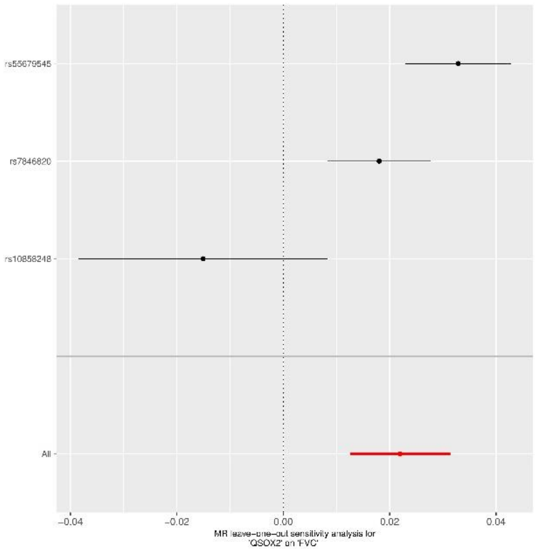
rs55679545
rs7846820
rs10858248
All
−0.04
−0.02
0.00
0.02
0.04
MR leave−one−out sensitivity analysis for
'QSOX2' on 'FVC'

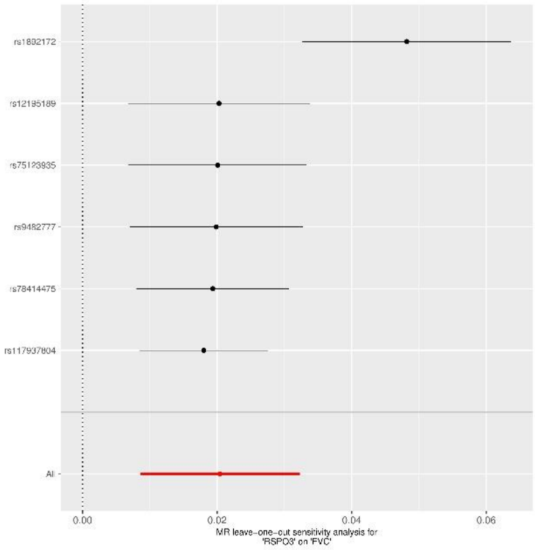
rs1892172
rs12195189
rs75123935
rs9482777
rs78414475
rs117937804
All
0.00
0.02
0.04
0.06
MR leave−one−out sensitivity analysis for
'RSPO3' on 'FVC'

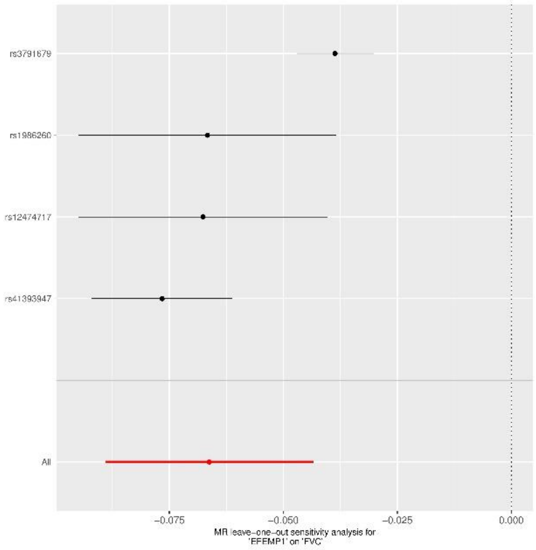
rs3791679
rs1986260
rs12474717
rs41393947
All
−0.075
−0.050
−0.025
0.000
MR leave−one−out sensitivity analysis for
'EFEMP1' on 'FVC'

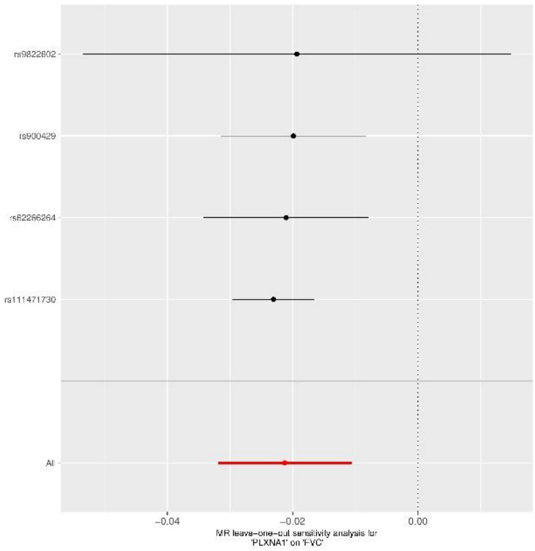
rs9822802
rs900429
rs62266264
rs111471730
All
−0.04
−0.02
0.00
MR leave−one−out sensitivity analysis for
'PLXNA1' on 'FVC'

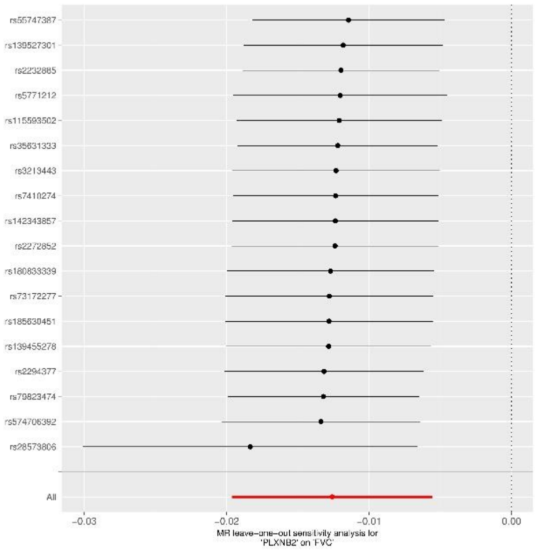
rs55747387
rs139527301
rs2232885
rs5771212
rs115593502
rs35631333
rs3213443
rs7410274
rs142343857
rs2272852
rs180833339
rs73172277
rs185630451
rs139455278
rs2294377
rs79823474
rs574706392
rs28573806
All
−0.03
−0.02
−0.01
0.00
MR leave−one−out sensitivity analysis for
'PLXNB2' on 'FVC'

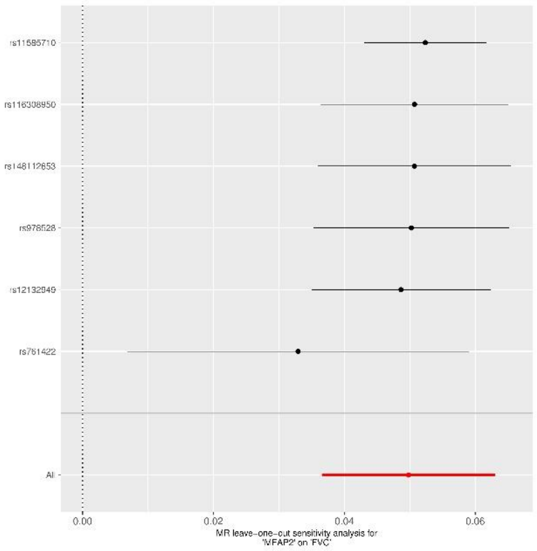
rs11585710
rs116308950
rs148112653
rs978528
rs12132949
rs761422
All
0.00
0.02
0.04
0.06
MR leave−one−out sensitivity analysis for
'MFAP2' on 'FVC'

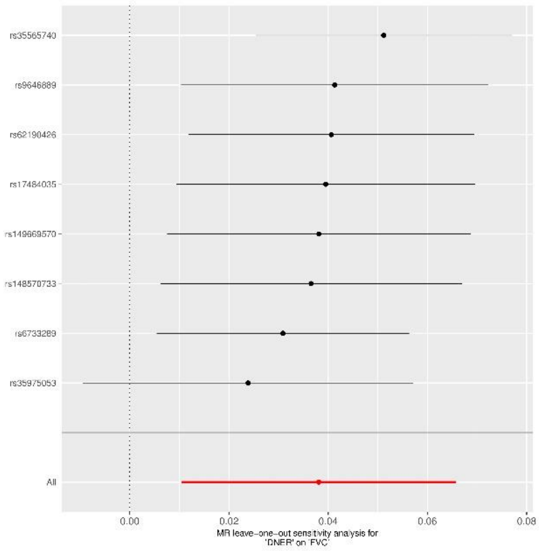
rs35565740
rs9646889
rs62190426
rs17484035
rs149669570
rs148570733
rs6733289
rs35975053
All
0.00
0.02
0.04
0.06
0.08
MR leave−one−out sensitivity analysis for
'DNER' on 'FVC'

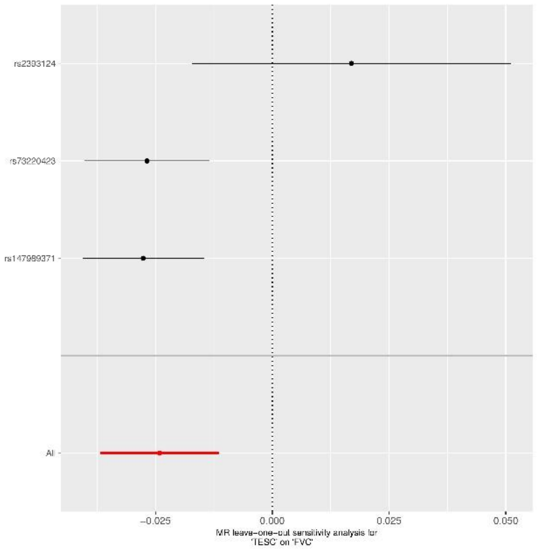
rs2393124
rs73220423
rs147989371
All
−0.025
0.000
0.025
0.050
MR leave−one−out sensitivity analysis for
'TESC' on 'FVC'

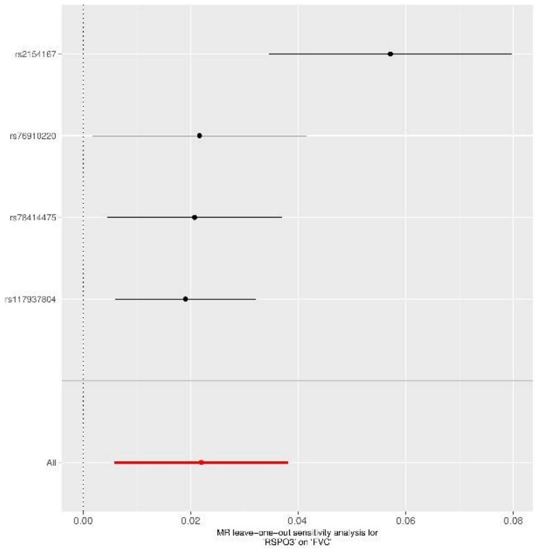
rs2154167
rs76910220
rs78414475
rs117937804
All
0.00
0.02
0.04
0.06
0.08
MR leave−one−out sensitivity analysis for
'RSPO3' on 'FVC'

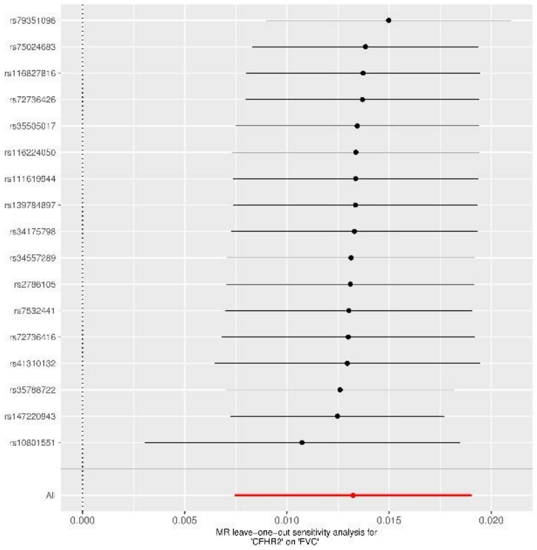
rs79351096
rs75024683
rs116827816
rs72736426
rs35505017
rs116224050
rs111619944
rs139784897
rs34175798
rs34557289
rs2786105
rs7532441
rs72736416
rs41310132
rs35786722
rs147220943
rs10801551
All
0.000
0.005
0.010
0.015
0.020
MR leave−one−out sensitivity analysis for
'CFHR2' on 'FVC'

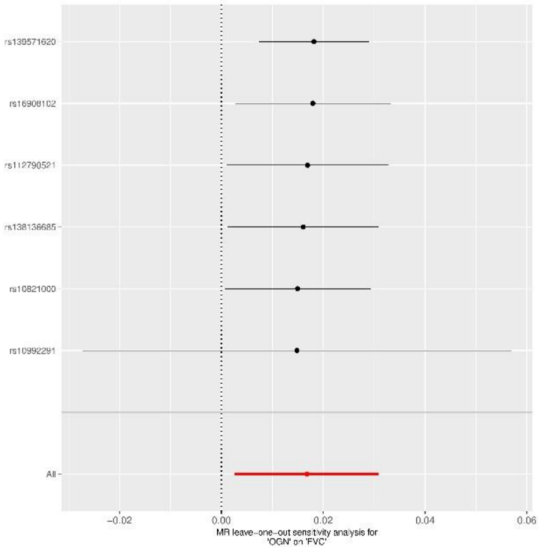
rs139571620
rs16908102
rs112790521
rs138136685
rs10821000
rs10992291
All
−0.02
0.00
0.02
0.04
0.06
MR leave−one−out sensitivity analysis for
'OGN' on 'FVC'

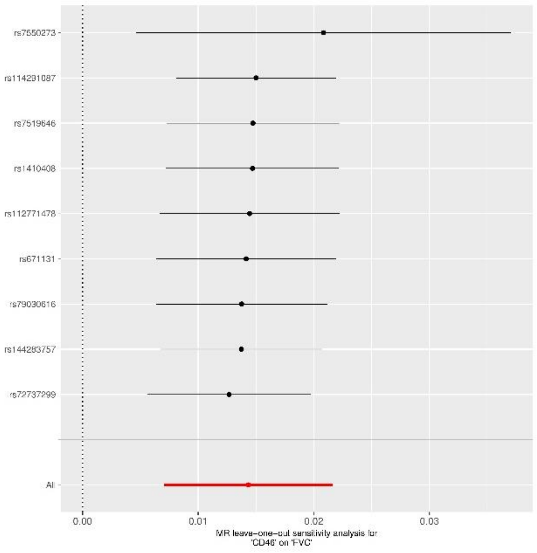

rs7550273
rs114291087
rs7519646
rs1410408
rs112771478
rs671131
rs79030616
rs144283757
rs72737299
All
0.00
0.01
0.02
0.03
MR leave–one–out sensitivity analysis for
'CD46' on 'FVC'

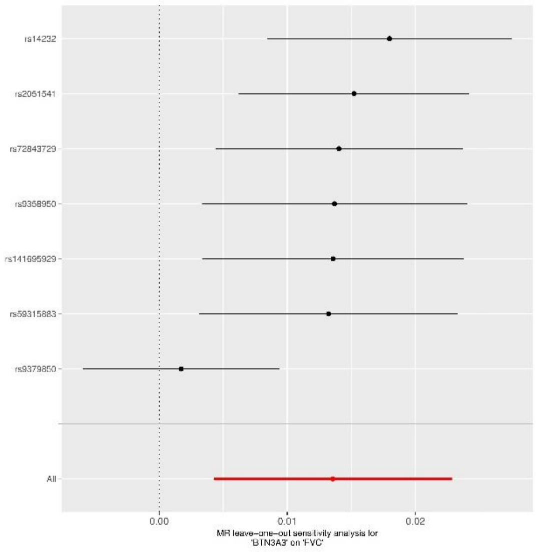

rs14232
rs2051541
rs72843729
rs9358950
rs141695929
rs59315883
rs9379850
All
0.00
0.01
0.02
MR leave–one–out sensitivity analysis for
'BTN3A3' on 'FVC'

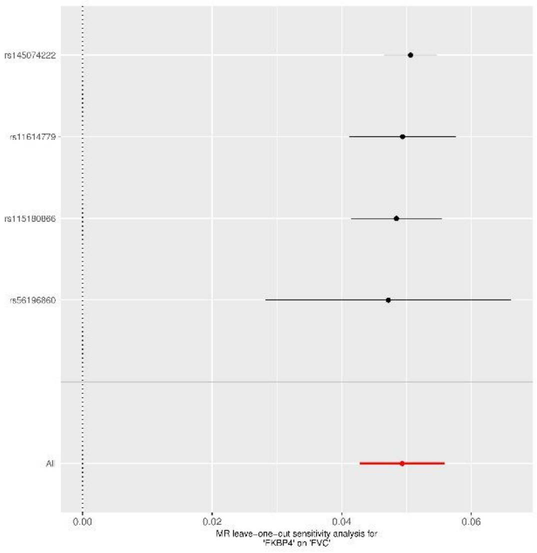

rs145074222
rs11614779
rs115180866
rs56196860
All
0.00
0.02
0.04
0.06
MR leave–one–out sensitivity analysis for
'FKBP4' on 'FVC'

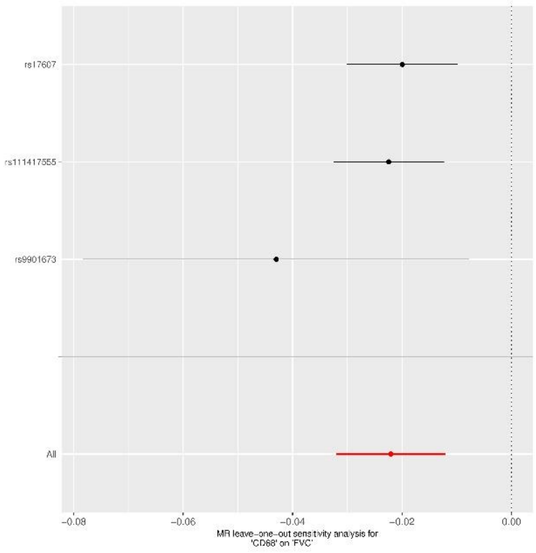

rs17607
rs111417555
rs9901673
All
−0.08
−0.06
−0.04
−0.02
0.00
MR leave–one–out sensitivity analysis for
'CD68' on 'FVC'

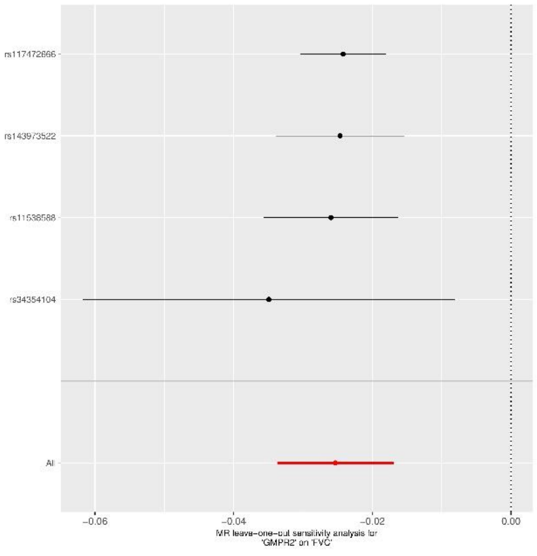

rs117472666
rs143973522
rs11538588
rs34354104
All
−0.06
−0.04
−0.02
0.00
MR leave–one–out sensitivity analysis for
'GMPR2' on 'FVC'

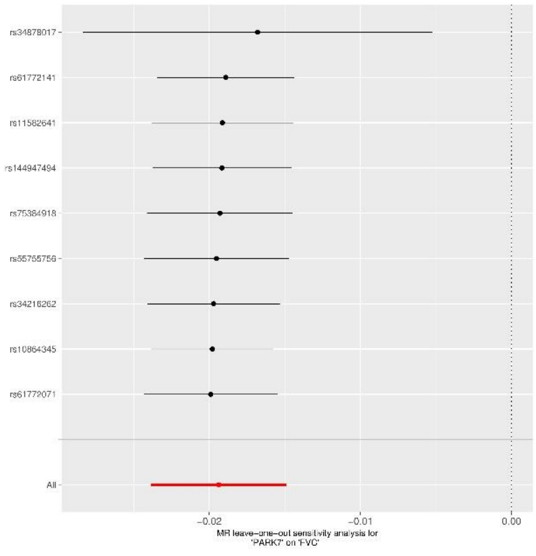

rs34878017
rs61772141
rs11582641
rs144947494
rs75384918
rs55755756
rs34216262
rs10864345
rs61772071
All
−0.02
−0.01
0.00
MR leave–one–out sensitivity analysis for
'PARK7' on 'FVC'

Figure S64: Results of leave-one-out analyses for PEF in the discovery MR analyses.

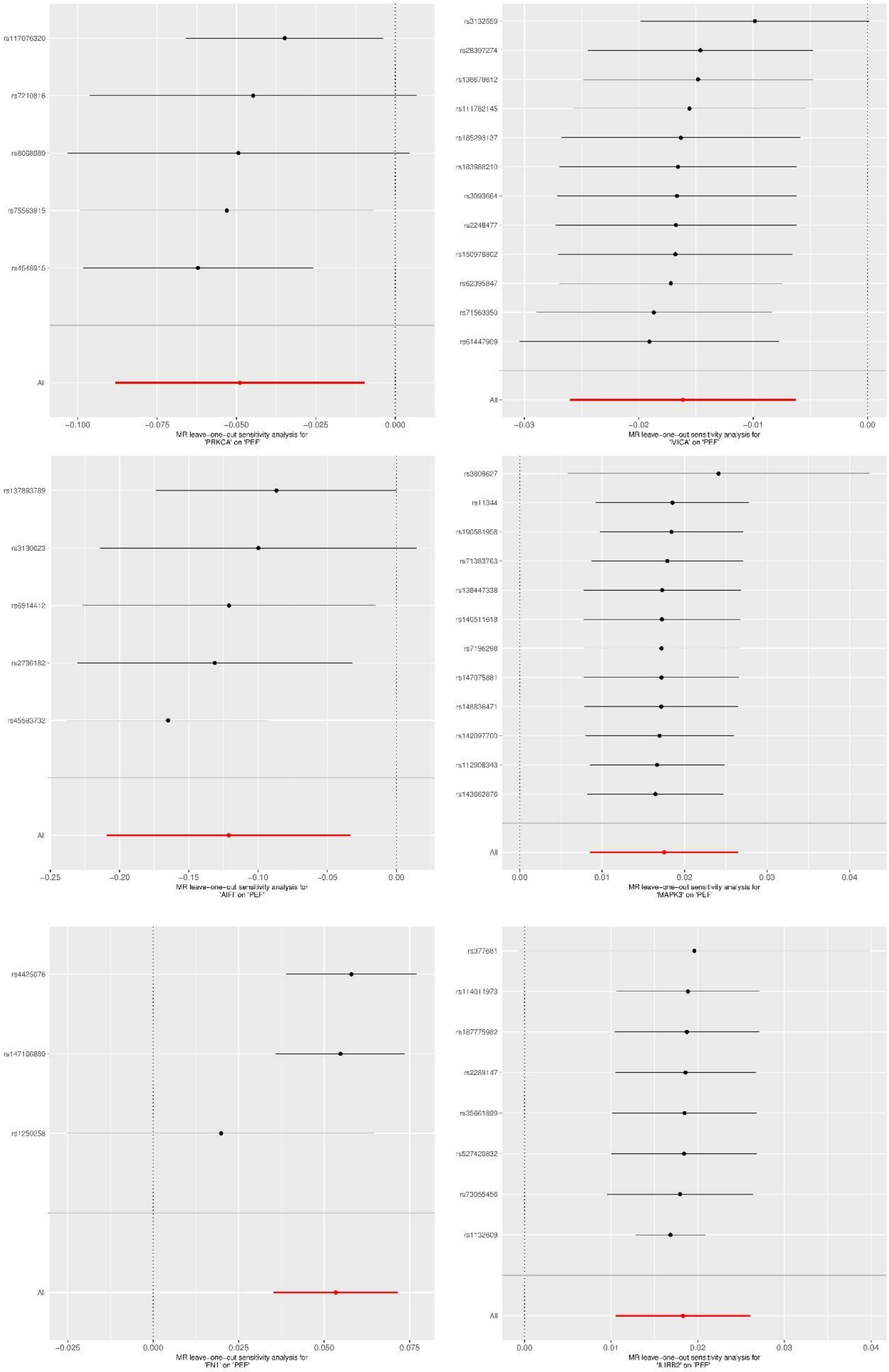

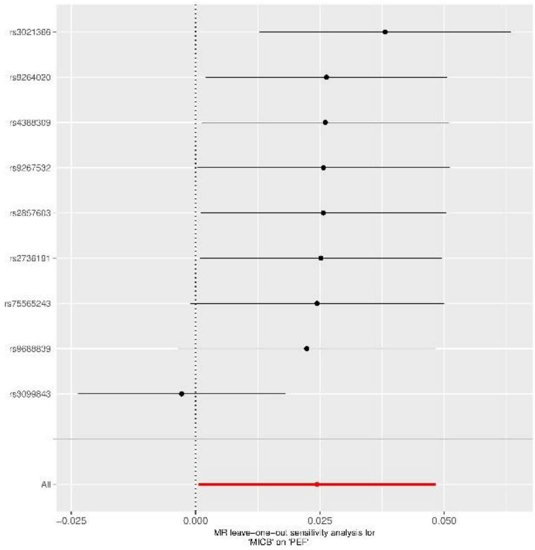
rs3021366
rs9264020
rs4388309
rs9267532
rs2857603
rs2736191
rs75565243
rs9688839
rs3099843
All
−0.025
0.000
0.025
0.050
MR leave−one−out sensitivity analysis for
'MICB' on 'PEF'

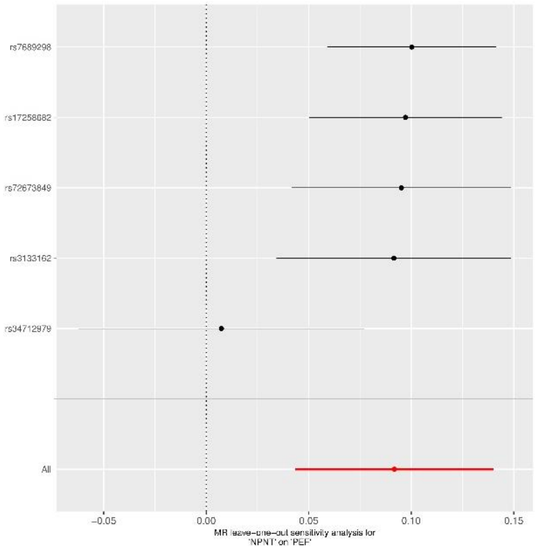
rs7689298
rs17258082
rs72673849
rs3133162
rs34712979
All
−0.05
0.00
0.05
0.10
0.15
MR leave−one−out sensitivity analysis for
'NPNT' on 'PEF'

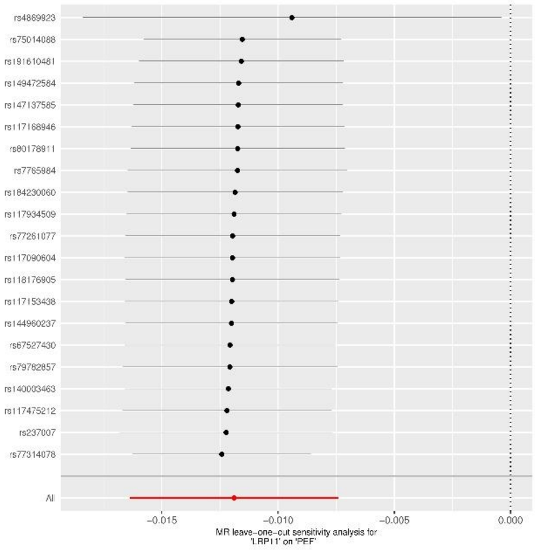
rs4869923
rs75014088
rs191610481
rs149472584
rs147137585
rs117168946
rs80178911
rs7765984
rs184230060
rs117934509
rs77261077
rs117090604
rs118176905
rs117153438
rs144960237
rs67527430
rs79782857
rs140003463
rs117475212
rs237007
rs77314078
All
−0.015
−0.010
−0.005
0.000
MR leave−one−out sensitivity analysis for
'LRP11' on 'PEF'

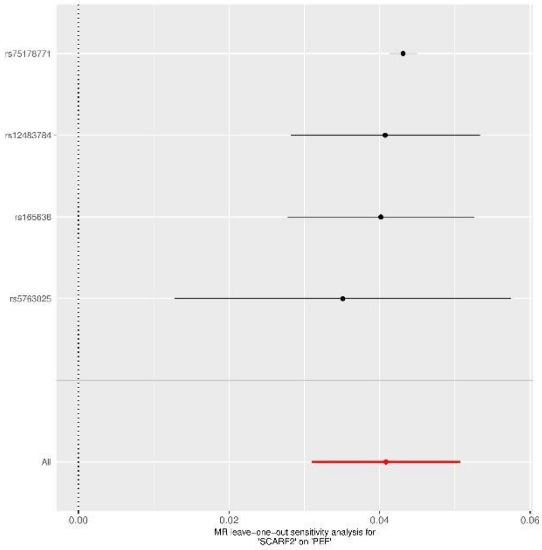
rs75178771
rs12483784
rs165638
rs5763025
All
0.00
0.02
0.04
0.06
MR leave−one−out sensitivity analysis for
'SCARF2' on 'PEF'

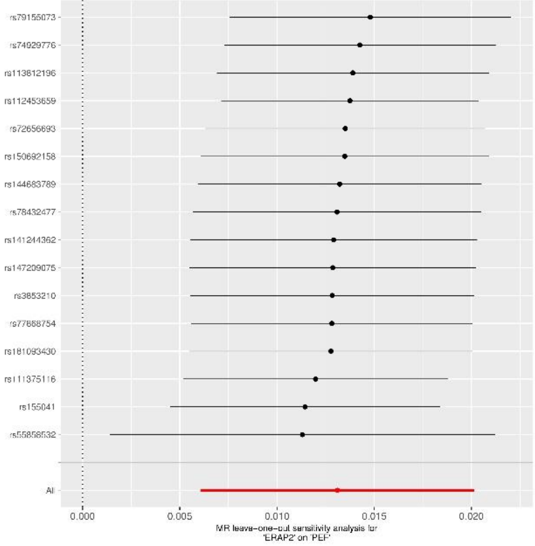
rs79155073
rs74929776
rs113812196
rs112453659
rs72656693
rs150692158
rs144683789
rs78432477
rs141244362
rs147209075
rs3853210
rs77888754
rs181093430
rs111375116
rs155041
rs55858532
All
0.000
0.005
0.010
0.015
0.020
MR leave−one−out sensitivity analysis for
'ERAP2' on 'PEF'

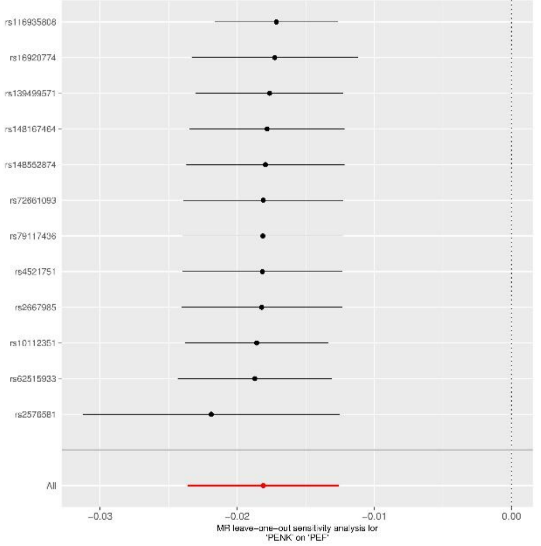
rs116935808
rs16920774
rs139499571
rs148167464
rs148552874
rs72861093
rs79117436
rs4521751
rs2667985
rs10112351
rs62515933
rs2576581
All
−0.03
−0.02
−0.01
0.00
MR leave−one−out sensitivity analysis for
'PENK' on 'PEF'

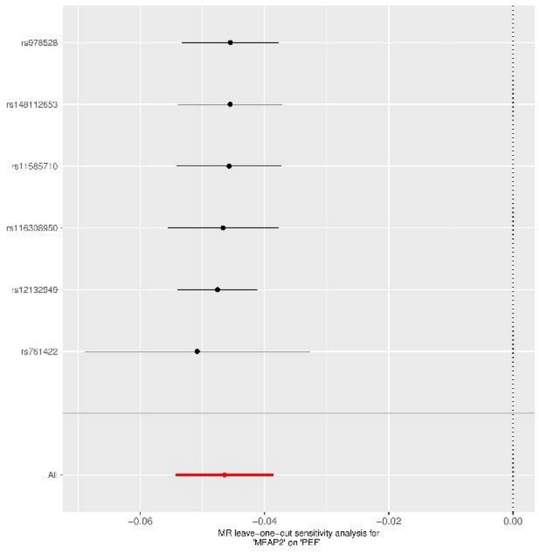
rs978528
rs148112653
rs11585710
rs116308950
rs12132849
rs761422
All
−0.06
−0.04
−0.02
0.00
MR leave−one−out sensitivity analysis for
'MFAP2' on 'PEF'

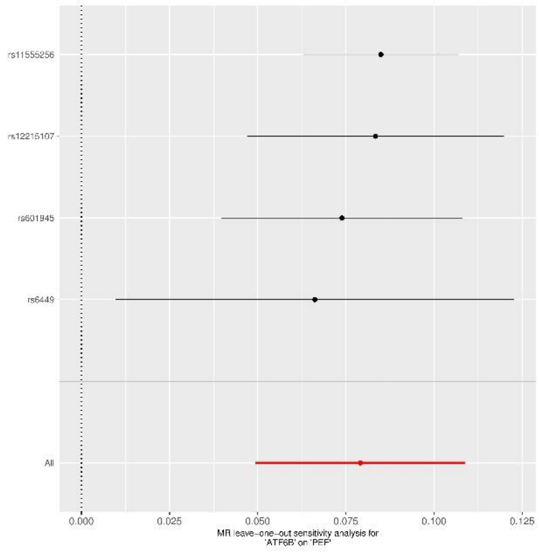
rs11555256
rs12215107
rs601945
rs6449
All
0.000
0.025
0.050
0.075
0.100
0.125
MR leave−one−out sensitivity analysis for
'ATF6B' on 'PEF'

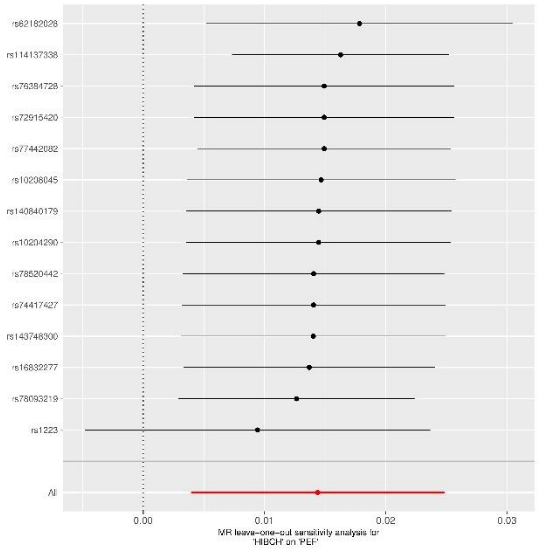
rs62182028
rs114137338
rs76384728
rs72915420
rs77442082
rs10208045
rs140840179
rs10204290
rs78520442
rs74417427
rs143748300
rs16832277
rs78093219
rs1223
All
0.00
0.01
0.02
0.03
MR leave−one−out sensitivity analysis for
'HIBCH' on 'PEF'

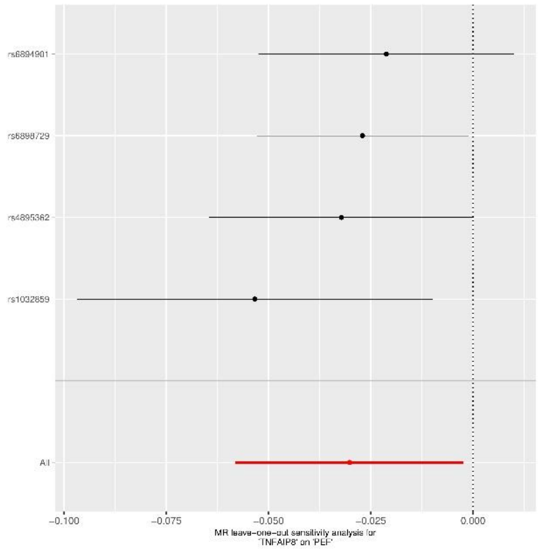
rs6894901
rs6898729
rs4895362
rs1032859
All
−0.100
−0.075
−0.050
−0.025
0.000
MR leave−one−out sensitivity analysis for
'TNFAIP8' on 'PEF'

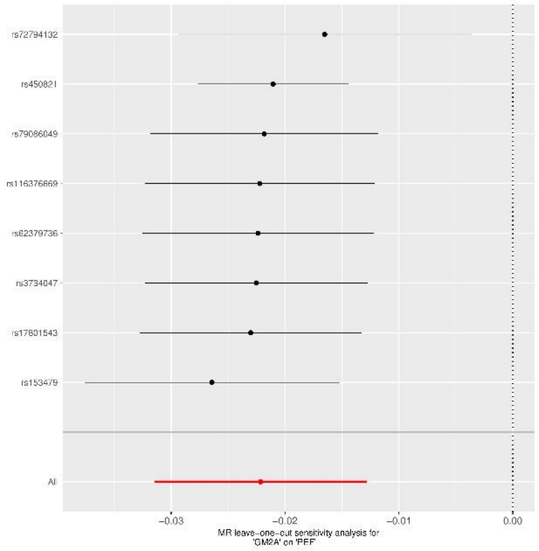
rs72794132
rs450821
rs79086049
rs116376669
rs62379736
rs3734047
rs17801543
rs153479
All
−0.03
−0.02
−0.01
0.00
MR leave−one−out sensitivity analysis for
'GM2A' on 'PEF'

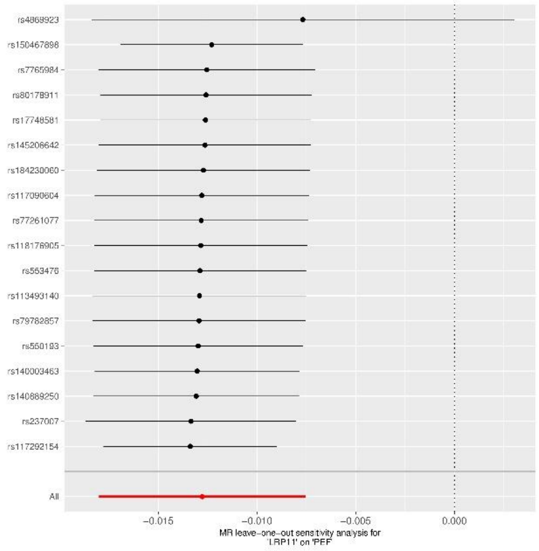
rs4869923
rs150467898
rs7765984
rs80178911
rs17748581
rs145206642
rs184230060
rs117090604
rs77261077
rs118176905
rs553478
rs113493140
rs79782857
rs550193
rs140003463
rs140889250
rs237007
rs117292154
All
−0.015
−0.010
−0.005
0.000
MR leave−one−out sensitivity analysis for
'LRP11' on 'PEF'

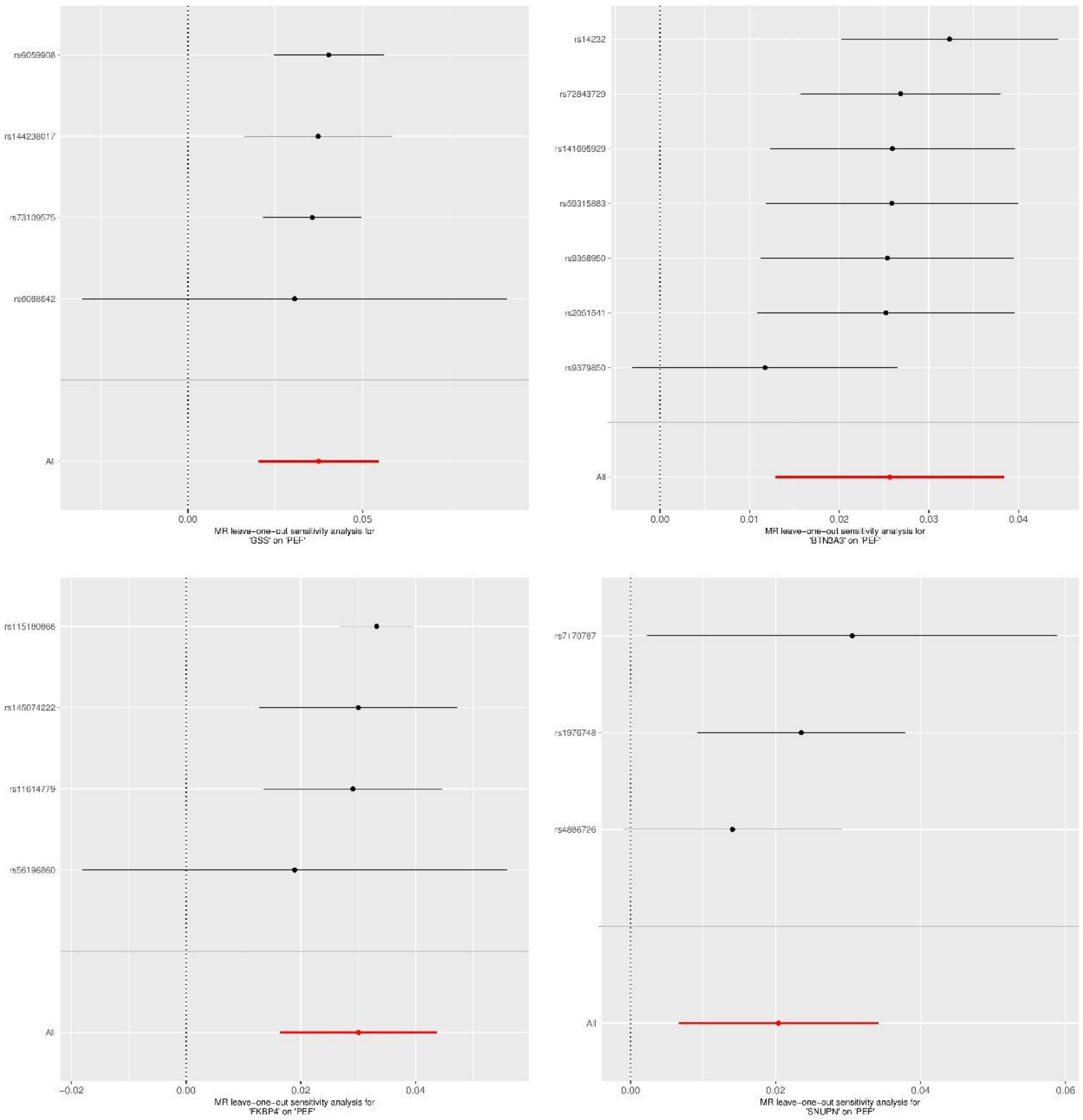
rs6059908
rs144238017
rs73109575
rs6088642
All
0.00
0.05
MR leave−one−out sensitivity analysis for
'GSS' on 'PEF'
rs14232
rs72843729
rs141695929
rs59315883
rs9358950
rs2051541
rs9379850
All
0.00
0.01
0.02
0.03
0.04
MR leave−one−out sensitivity analysis for
'BTN3A3' on 'PEF'
rs115180866
rs145074222
rs11614779
rs56196860
All
−0.02
0.00
0.02
0.04
MR leave−one−out sensitivity analysis for
'FKBP4' on 'PEF'
rs7170787
rs1976748
rs4886726
All
0.00
0.02
0.04
0.06
MR leave−one−out sensitivity analysis for
'SNUPN' on 'PEF'

Figure S65: Results of leave-one-out analyses for FEV1/FVC in the discovery MR analyses.

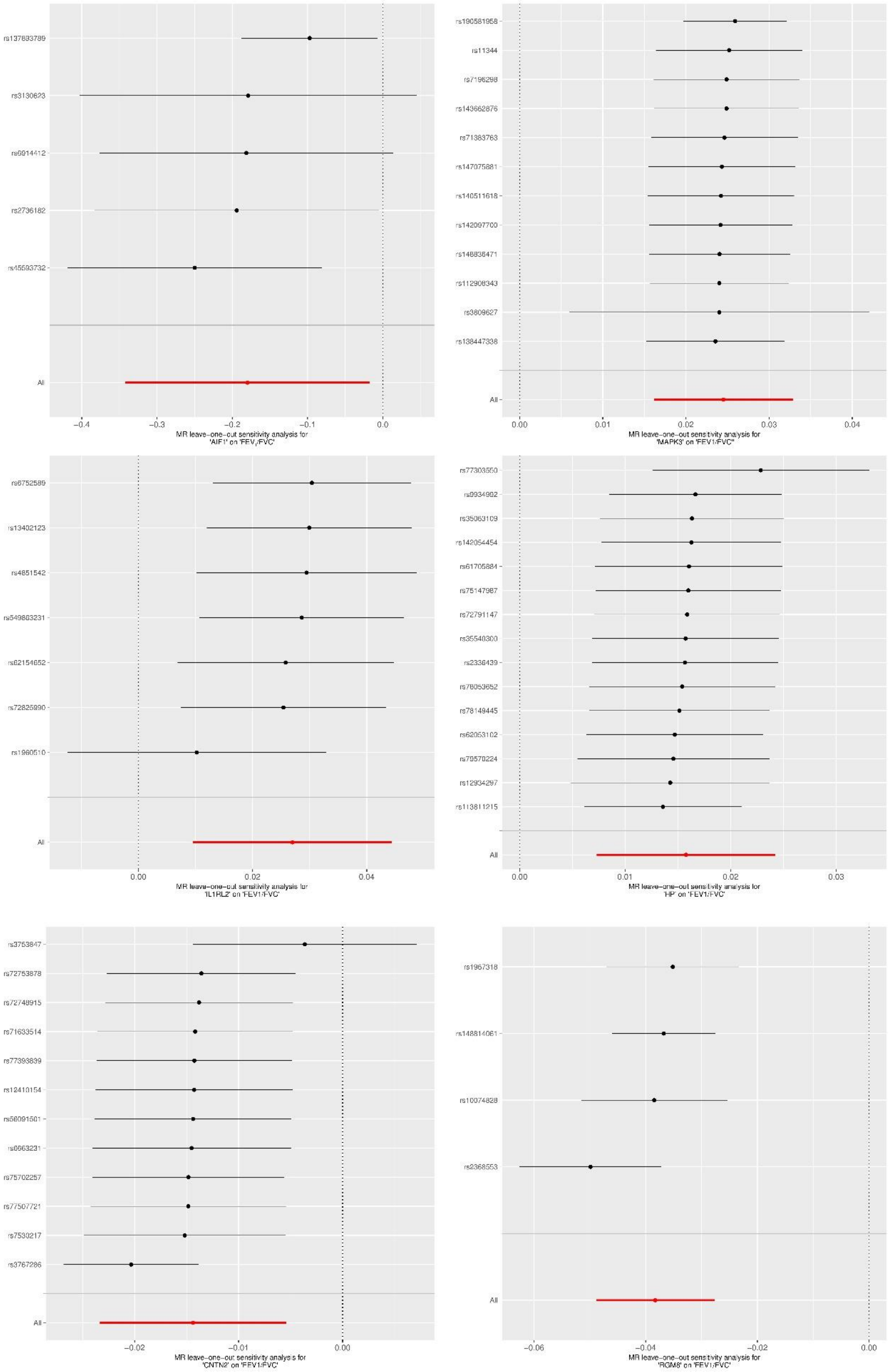

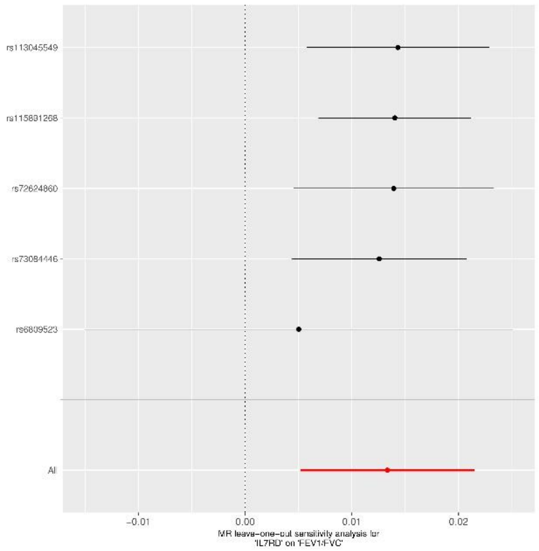
rs113045549
rs115891268
rs72624860
rs73084446
rs6809523
All
−0.01
0.00
0.01
0.02
MR leave−one−out sensitivity analysis for
'IL7RD' on 'FEV1/FVC'

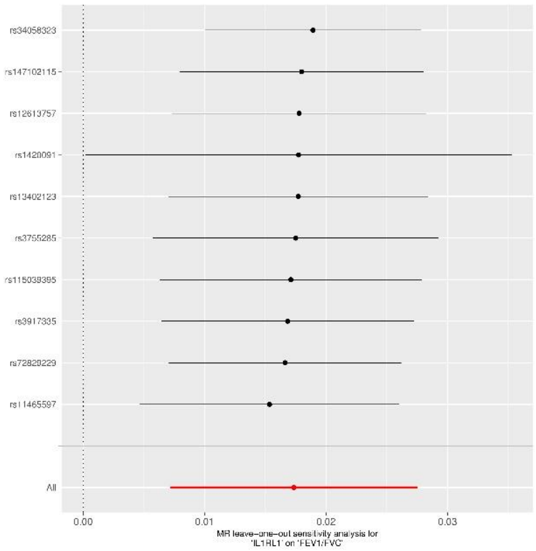
rs34058323
rs147102115
rs12613757
rs1420091
rs13402123
rs3755285
rs115038395
rs3917335
rs72820229
rs11465597
All
0.00
0.01
0.02
0.03
MR leave−one−out sensitivity analysis for
'IL1RL1' on 'FEV1/FVC'

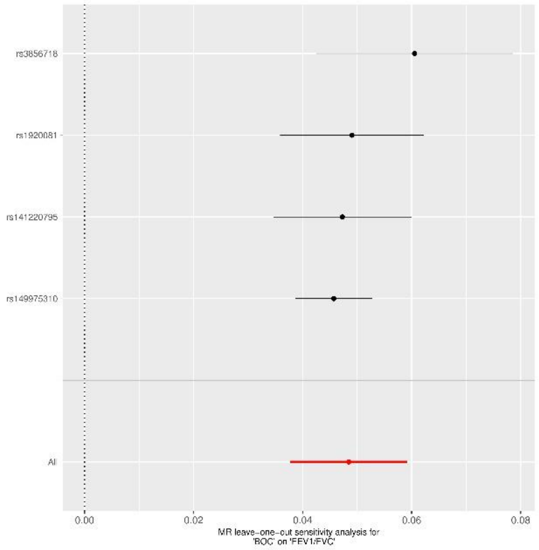
rs3856718
rs1920081
rs141220795
rs149975310
All
0.00
0.02
0.04
0.06
0.08
MR leave−one−out sensitivity analysis for
'BOC' on 'FEV1/FVC'

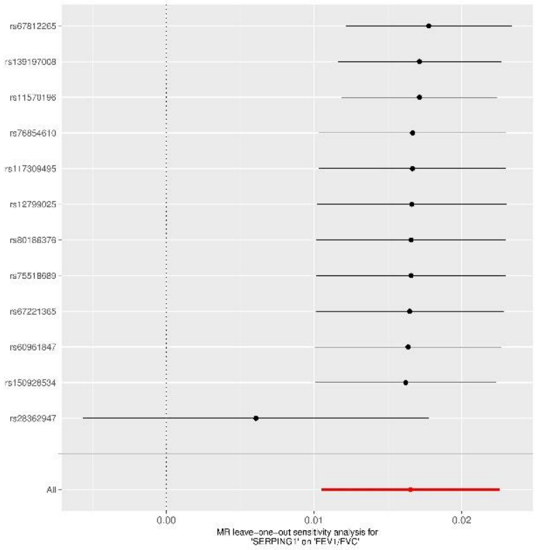
rs67812265
rs139197008
rs11570196
rs76854610
rs117309495
rs12799025
rs80186376
rs75518689
rs67221365
rs60961847
rs150928534
rs28362947
All
0.00
0.01
0.02
MR leave−one−out sensitivity analysis for
'SERPING1' on 'FEV1/FVC'

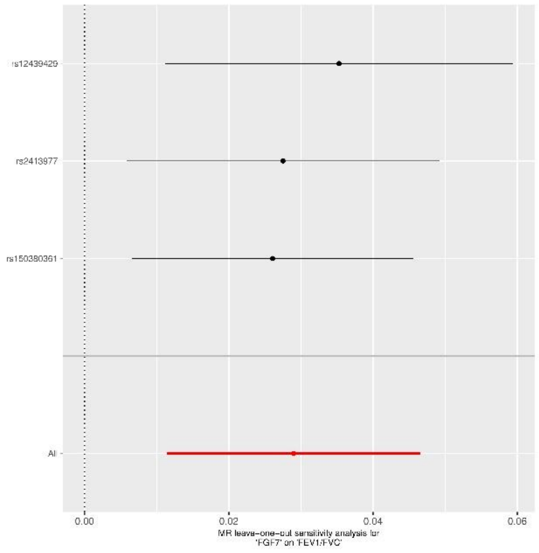
rs12439429
rs2413977
rs150380361
All
0.00
0.02
0.04
0.06
MR leave−one−out sensitivity analysis for
'FGF7' on 'FEV1/FVC'

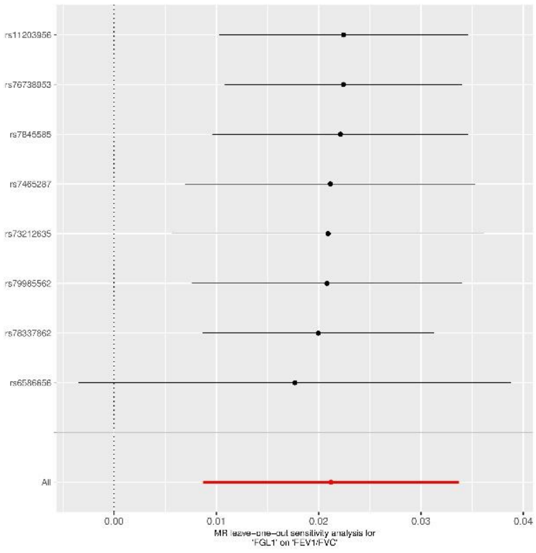
rs11203956
rs76738953
rs7845585
rs7465287
rs73212635
rs79985562
rs78337862
rs6586656
All
0.00
0.01
0.02
0.03
0.04
MR leave−one−out sensitivity analysis for
'FGL1' on 'FEV1/FVC'

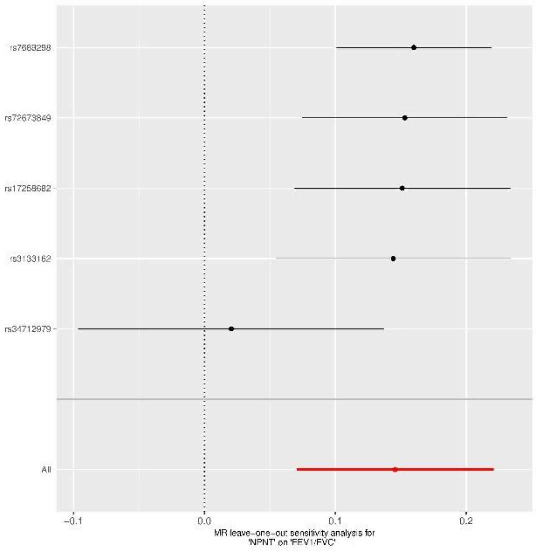

rs7663298
rs72673849
rs17258682
rs3133162
rs34712979
All
−0.1
0.0
0.1
0.2
MR leave−one−out sensitivity analysis for
'NPNT' on 'FEV1/FVC'

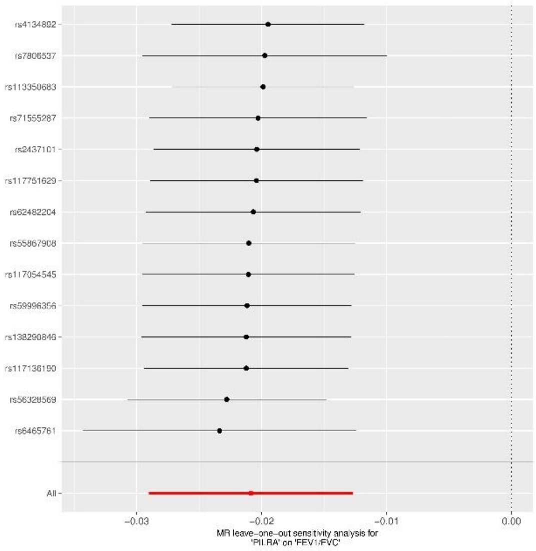

rs4134802
rs7806537
rs113350683
rs71555287
rs2437101
rs117751629
rs62482204
rs55867908
rs117054545
rs59998356
rs138298849
rs117136190
rs56328569
rs6465761
All
−0.03
−0.02
−0.01
0.00
MR leave−one−out sensitivity analysis for
'PILRA' on 'FEV1/FVC'

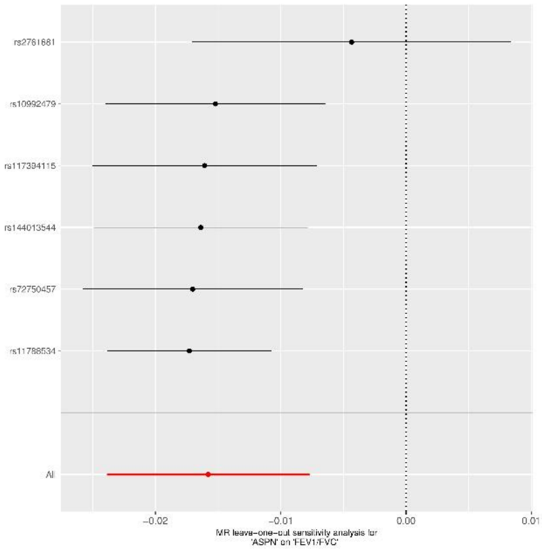

rs2761681
rs10992479
rs117394115
rs144013544
rs72750457
rs11788534
All
−0.02
−0.01
0.00
0.01
MR leave−one−out sensitivity analysis for
'ASPN' on 'FEV1/FVC'

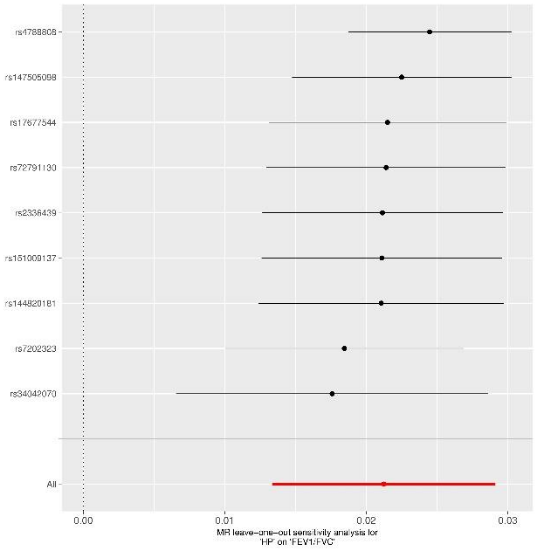

rs4788808
rs147505098
rs17677544
rs72791130
rs2336439
rs151009137
rs144820181
rs7202323
rs34042070
All
0.00
0.01
0.02
0.03
MR leave−one−out sensitivity analysis for
'HP' on 'FEV1/FVC'

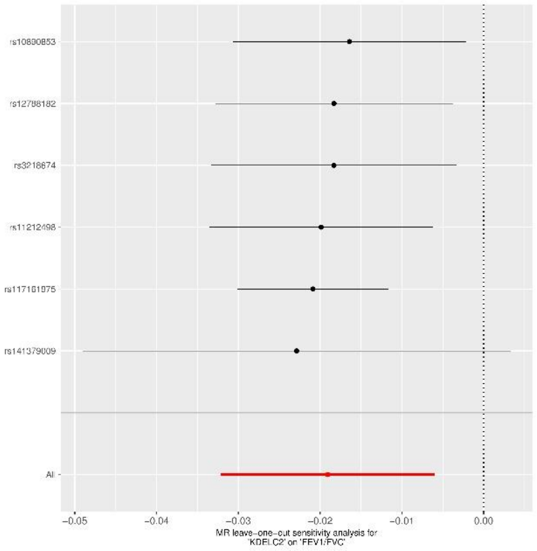

rs10890853
rs12788182
rs3218674
rs11212498
rs117161975
rs141379009
All
−0.05
−0.04
−0.03
−0.02
−0.01
0.00
MR leave−one−out sensitivity analysis for
'KDELC2' on 'FEV1/FVC'

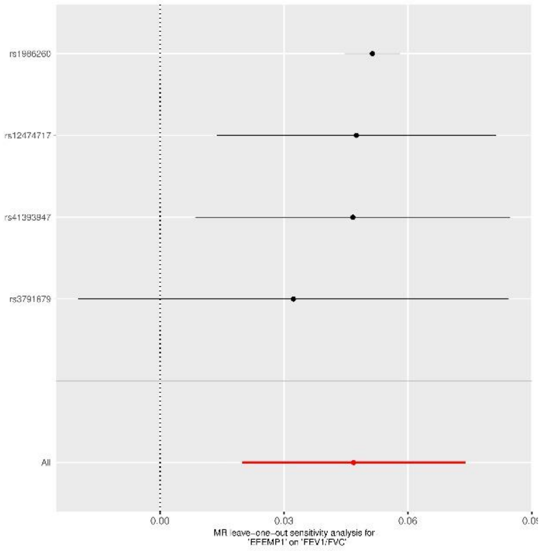

rs1986260
rs12474717
rs41393947
rs3791679
All
0.00
0.03
0.06
MR leave−one−out sensitivity analysis for
'EFEMP1' on 'FEV1/FVC'

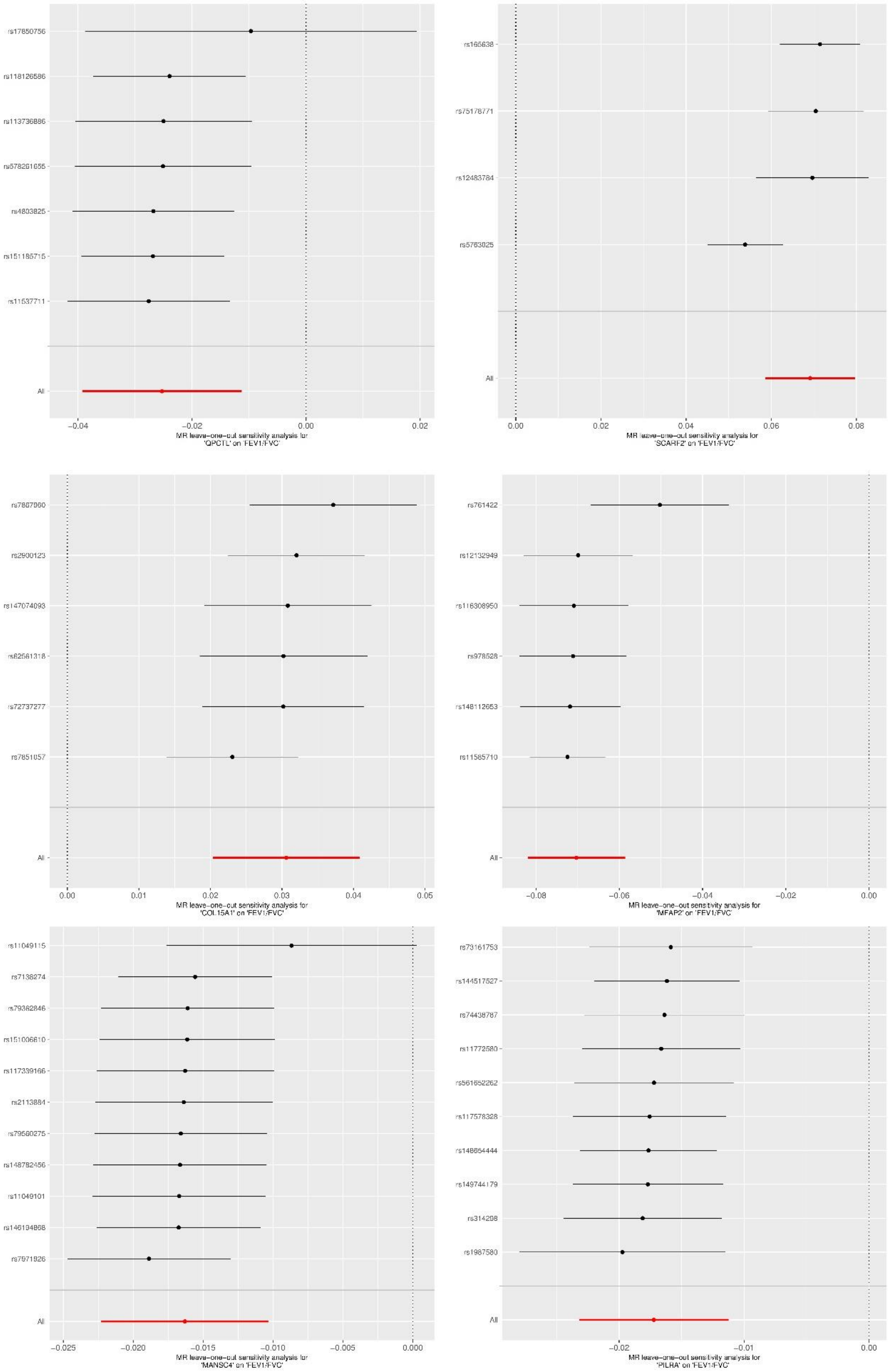
rs17850756
rs118126586
rs113736886
rs578261655
rs4803825
rs151185715
rs11537711
All
−0.04
−0.02
0.00
0.02
MR leave−one−out sensitivity analysis for
'QPCTL' on 'FEV1/FVC'
rs165638
rs75178771
rs12483784
rs5763025
All
0.00
0.02
0.04
0.06
0.08
MR leave−one−out sensitivity analysis for
'SCARF2' on 'FEV1/FVC'
rs7867960
rs2900123
rs147074093
rs62561318
rs72737277
rs7851057
All
0.00
0.01
0.02
0.03
0.04
0.05
MR leave−one−out sensitivity analysis for
'COL15A1' on 'FEV1/FVC'
rs761422
rs12132949
rs116308950
rs978528
rs148112653
rs11585710
All
−0.08
−0.06
−0.04
−0.02
0.00
MR leave−one−out sensitivity analysis for
'MFAP2' on 'FEV1/FVC'
rs11049115
rs7138274
rs79362846
rs151006610
rs117339166
rs2113884
rs79560275
rs148782456
rs11049101
rs146194868
rs7971926
All
−0.025
−0.020
−0.015
−0.010
−0.005
0.000
MR leave−one−out sensitivity analysis for
'MANSC4' on 'FEV1/FVC'
rs73161753
rs144517527
rs74438787
rs11772580
rs561652262
rs117578328
rs148654444
rs149744179
rs314298
rs1987580
All
−0.02
−0.01
0.00
MR leave−one−out sensitivity analysis for
'PILRA' on 'FEV1/FVC'

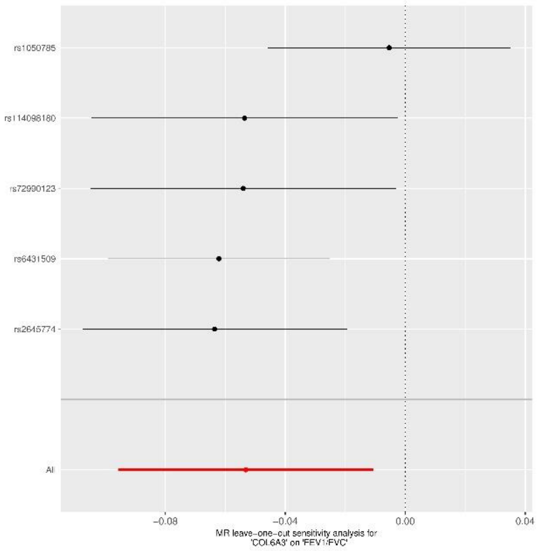
rs1050785
rs114098180
rs72990123
rs6431509
rs2645774
All
−0.08
−0.04
0.00
0.04
MR leave−one−out sensitivity analysis for
'COL6A3' on 'FEV1/FVC'

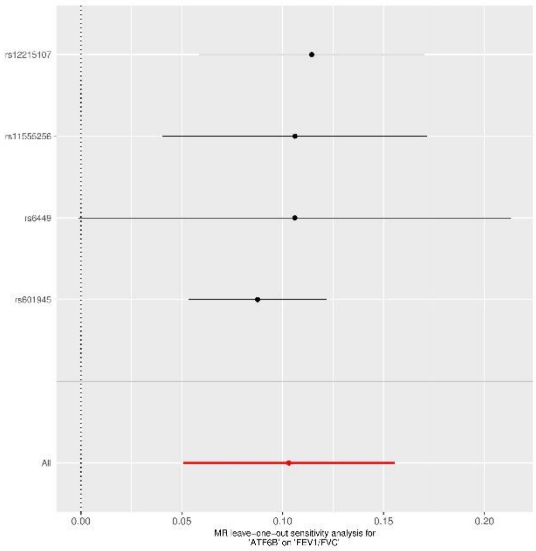
rs12215107
rs11555256
rs6449
rs601945
All
0.00
0.05
0.10
0.15
0.20
MR leave−one−out sensitivity analysis for
'ATF6B' on 'FEV1/FVC'

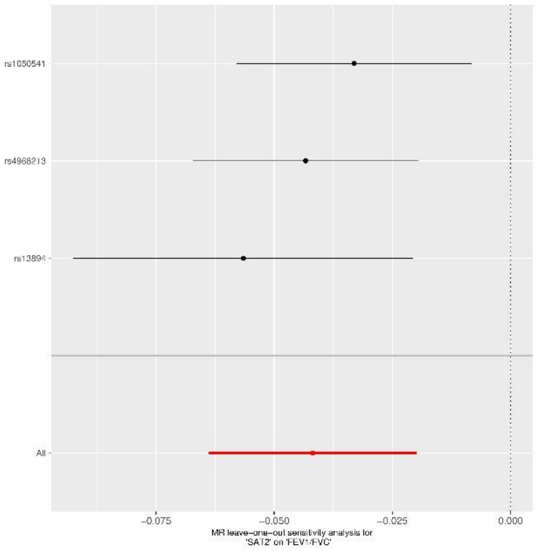
rs1050541
rs4968213
rs13894
All
−0.075
−0.050
−0.025
0.000
MR leave−one−out sensitivity analysis for
'SAT2' on 'FEV1/FVC'

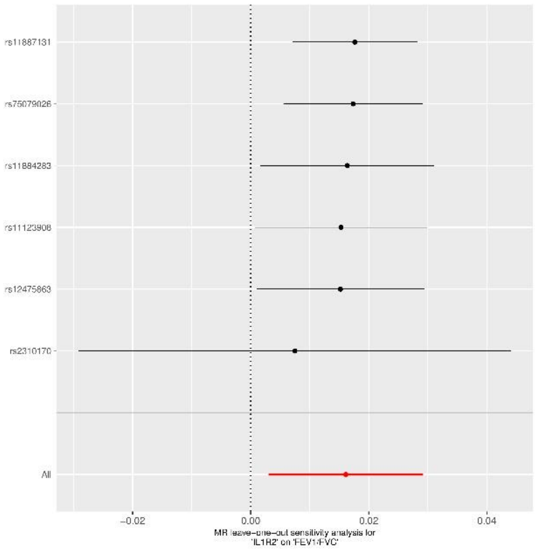
rs11887131
rs75079026
rs11884283
rs11123908
rs12475863
rs2310170
All
−0.02
0.00
0.02
0.04
MR leave−one−out sensitivity analysis for
'IL1R2' on 'FEV1/FVC'

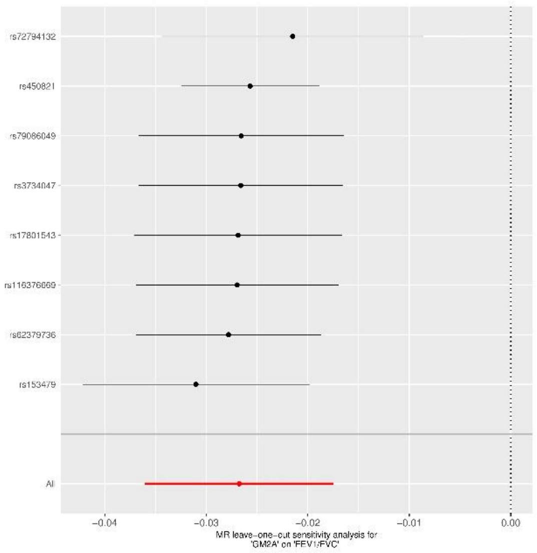
rs72794132
rs450821
rs79086049
rs3734047
rs17801543
rs116376669
rs62379736
rs153479
All
−0.04
−0.03
−0.02
−0.01
0.00
MR leave−one−out sensitivity analysis for
'GM2A' on 'FEV1/FVC'

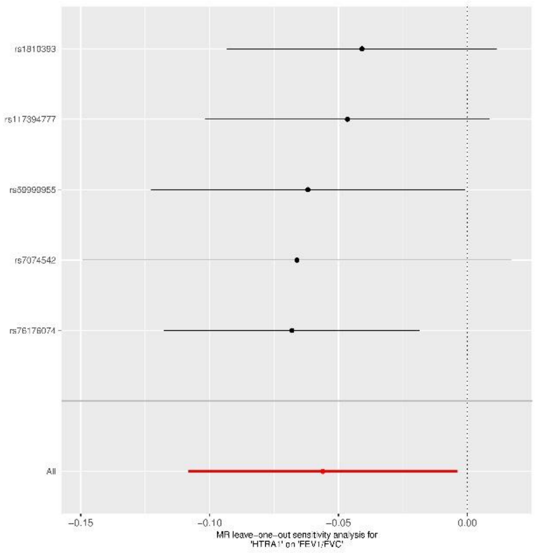
rs1810393
rs117394777
rs59990955
rs7074542
rs76176074
All
−0.15
−0.10
−0.05
0.00
MR leave−one−out sensitivity analysis for
'HTRA1' on 'FEV1/FVC'

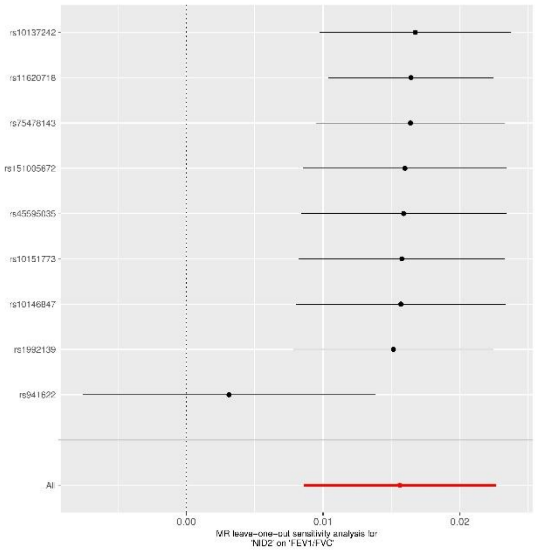
rs10137242
rs11620718
rs75478143
rs151005672
rs45595035
rs10151773
rs10146847
rs1992139
rs941622
All
0.00
0.01
0.02
MR leave−one−out sensitivity analysis for
'NID2' on 'FEV1/FVC'

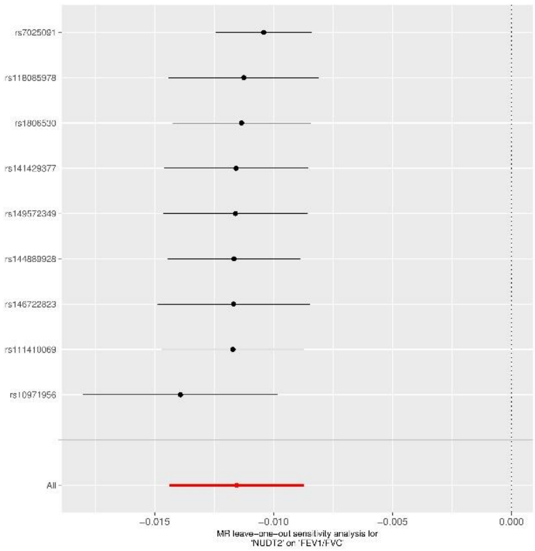
rs7025091
rs118085978
rs1806530
rs141429377
rs149572349
rs144889928
rs146722823
rs111410069
rs10971956
All
−0.015
−0.010
−0.005
0.000
MR leave−one−out sensitivity analysis for
'NUDT2' on 'FEV1/FVC'

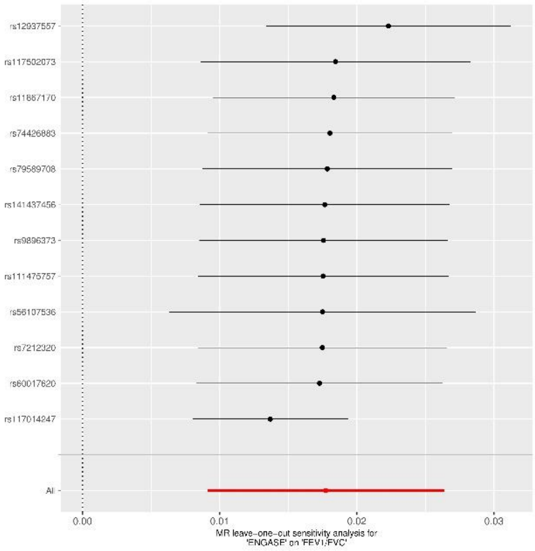
rs12937557
rs117502073
rs11867170
rs74426883
rs79589708
rs141437456
rs9896373
rs111475757
rs56107536
rs7212320
rs60017620
rs117014247
All
0.00
0.01
0.02
0.03
MR leave−one−out sensitivity analysis for
'ENGASE' on 'FEV1/FVC'

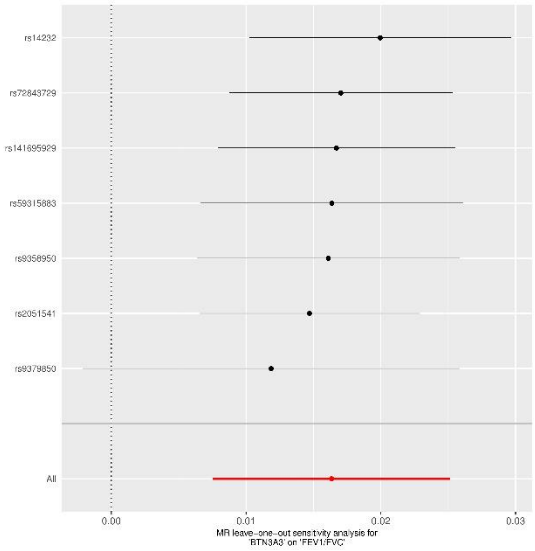
rs14232
rs72843729
rs141695929
rs59315883
rs9358950
rs2051541
rs9379850
All
0.00
0.01
0.02
0.03
MR leave−one−out sensitivity analysis for
'BTN3A3' on 'FEV1/FVC'

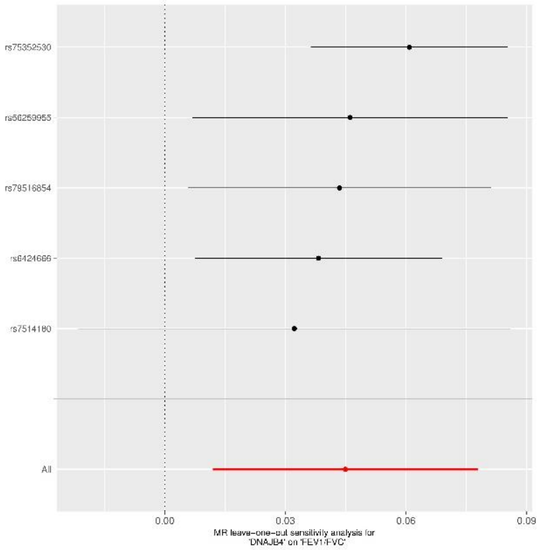
rs75352530
rs56259955
rs79516854
rs6424666
rs7514180
All
0.00
0.03
0.06
0.09
MR leave−one−out sensitivity analysis for
'DNAJB4' on 'FEV1/FVC'

Figure S66: Results of leave-one-out analyses for FEV1 in the replication MR analyses.

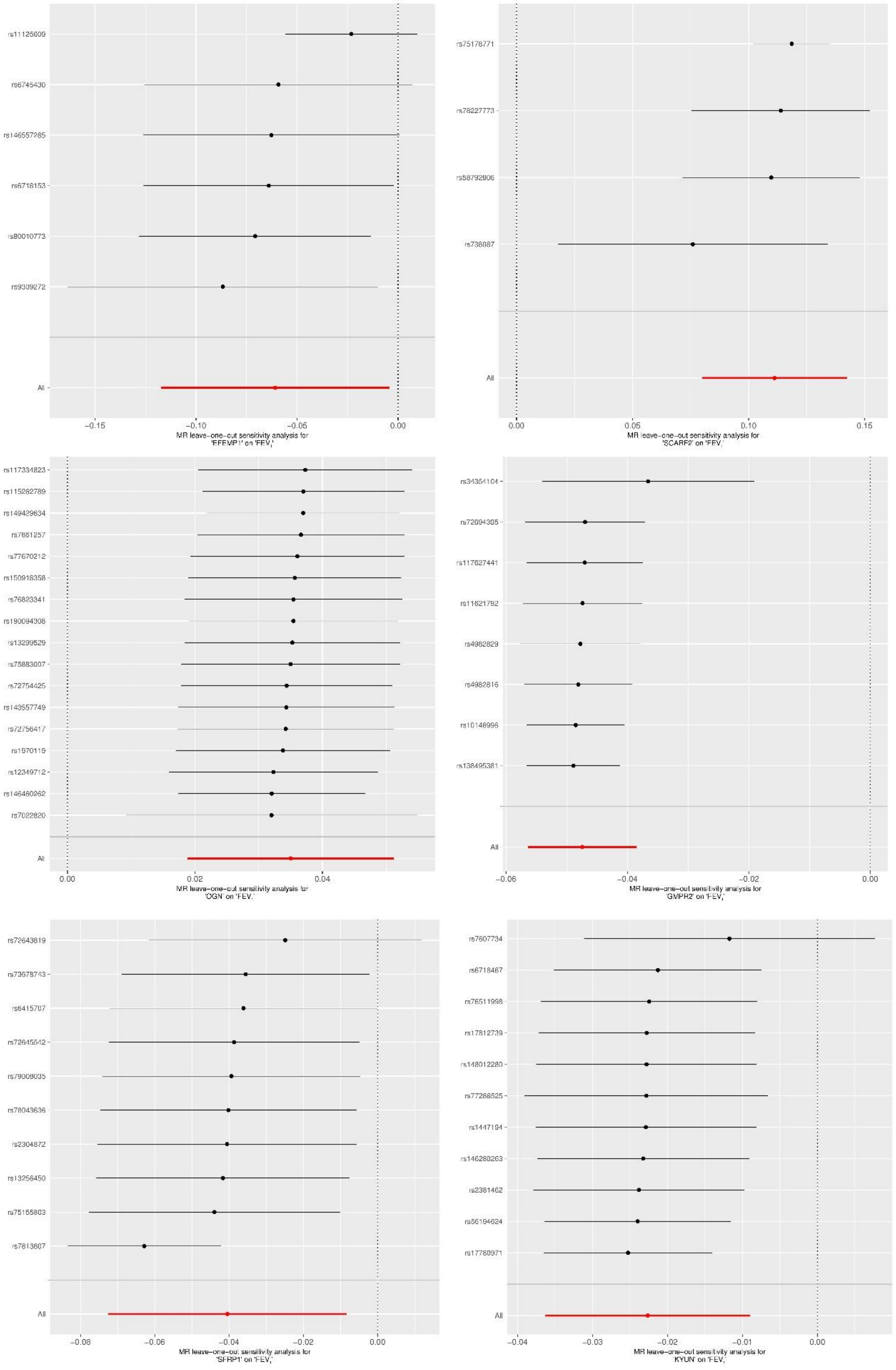

Figure S67: Results of leave-one-out analyses for FVC in the replication MR analyses.

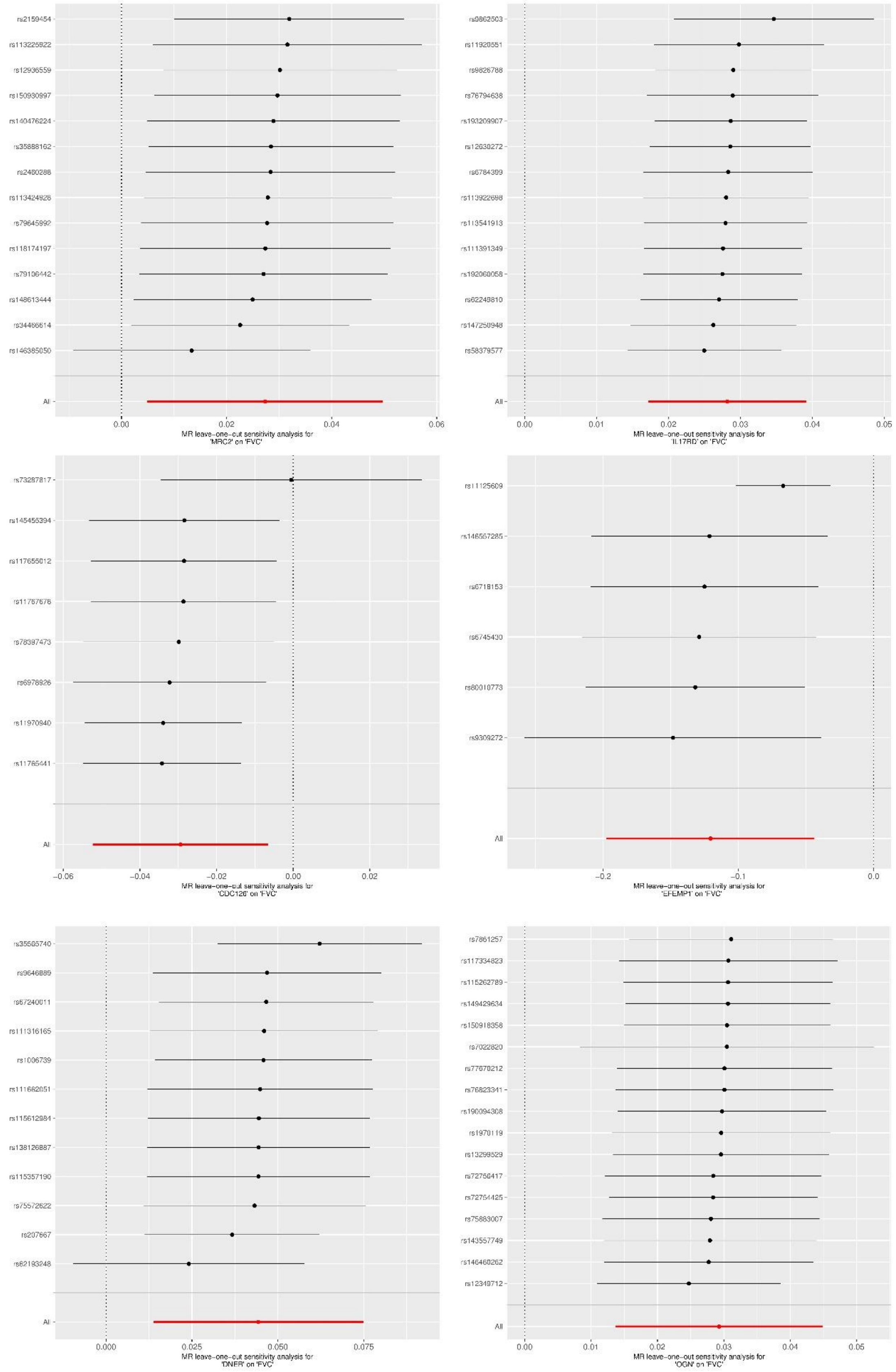

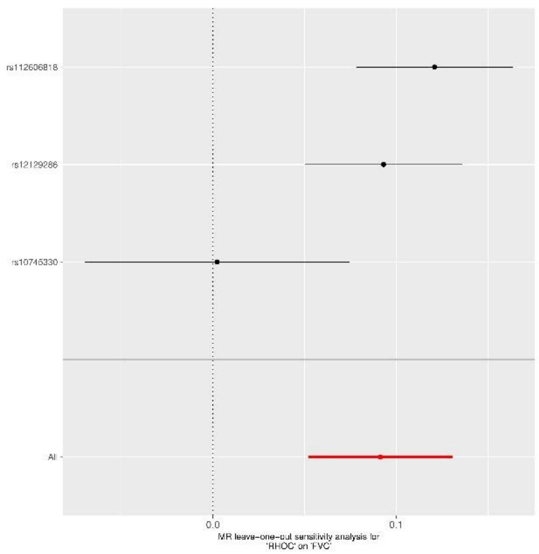
rs112606818
rs12129286
rs10745330
All
0.0
0.1
MR leave−one−out sensitivity analysis for
'RHOC' on 'FVC'

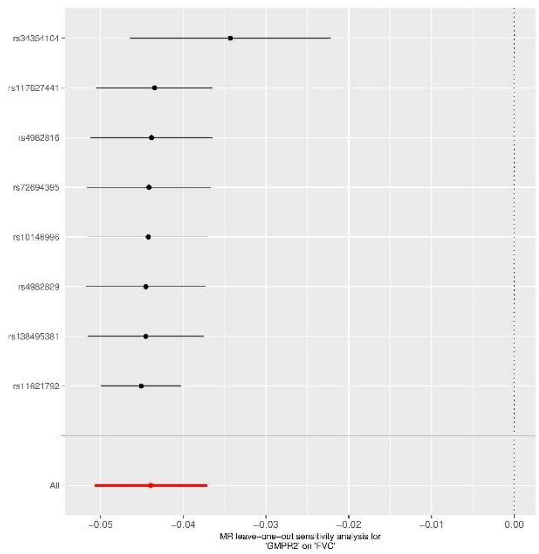
rs34354104
rs117627441
rs4982816
rs72694395
rs10146996
rs4982829
rs138495381
rs11621792
All
−0.05
−0.04
−0.03
−0.02
−0.01
0.00
MR leave−one−out sensitivity analysis for
'GMPR2' on 'FVC'

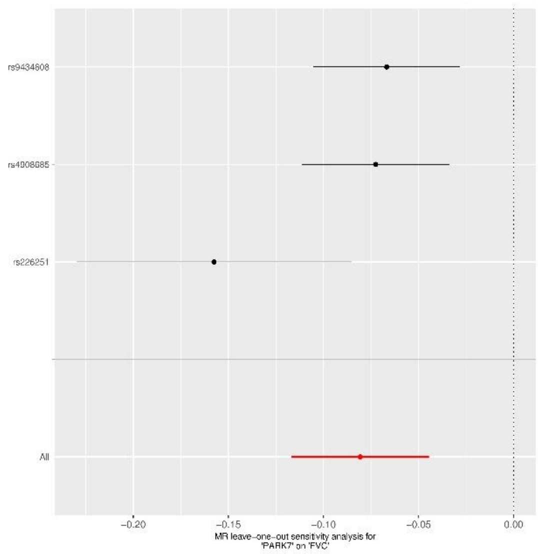
rs9434608
rs4908085
rs226251
All
−0.20
−0.15
−0.10
−0.05
0.00
MR leave−one−out sensitivity analysis for
'PARK7' on 'FVC'

Figure S68: Results of leave-one-out analyses for PEF in the replication MR analyses.

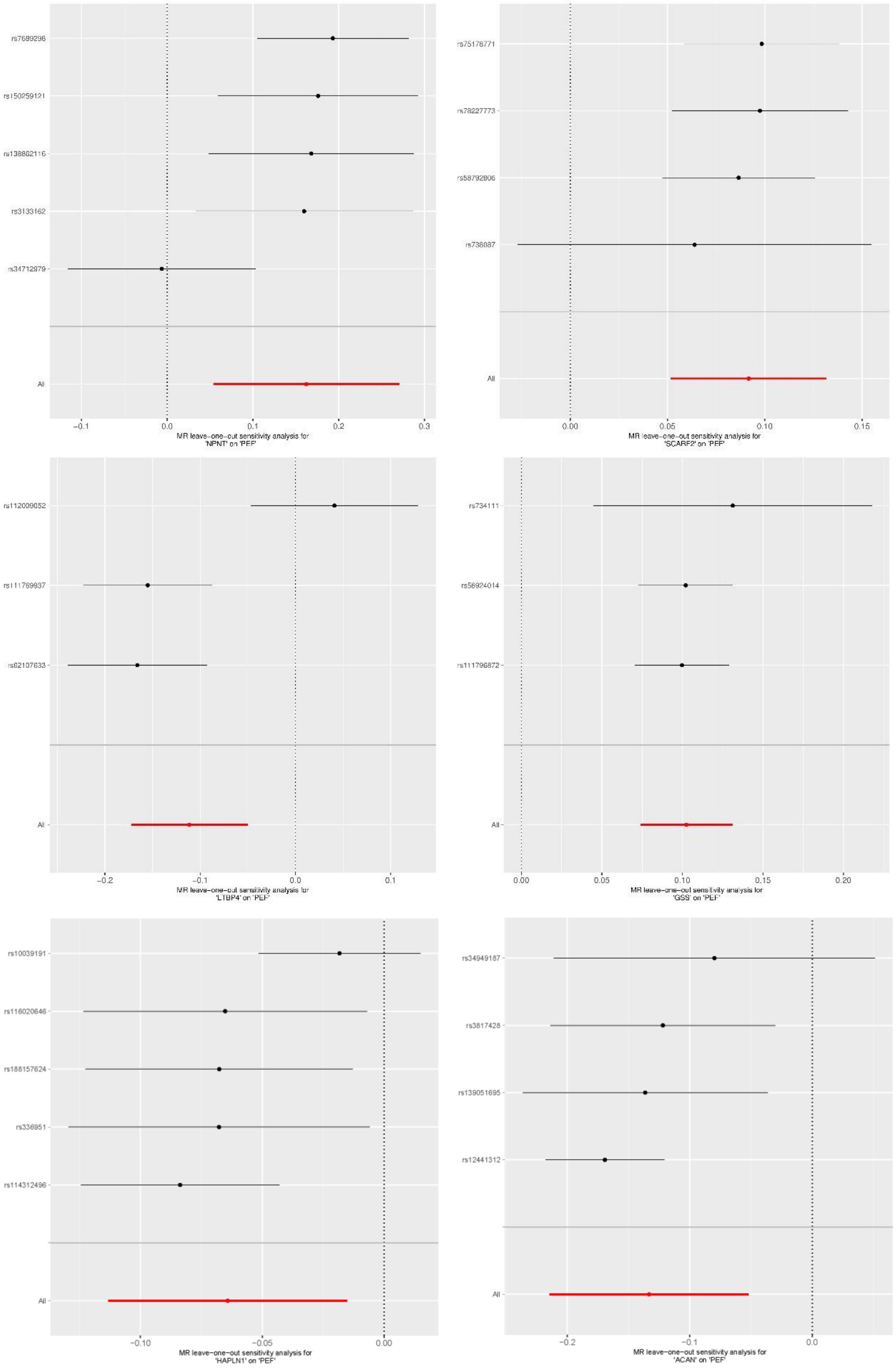

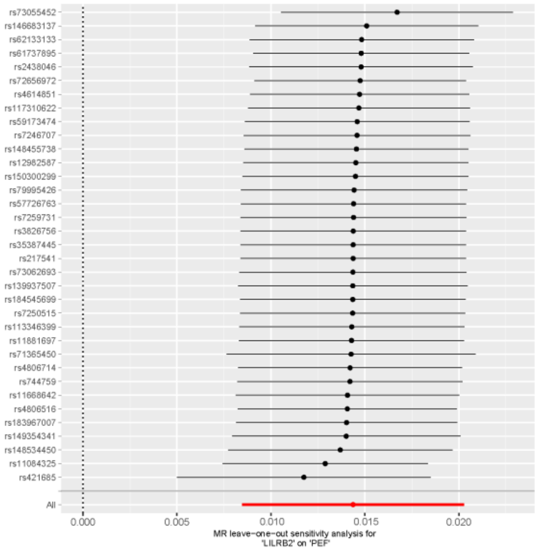
rs73055452
rs146683137
rs62133133
rs61737895
rs2438046
rs72656972
rs4614851
rs117310622
rs59173474
rs7246707
rs148455738
rs12982587
rs150300299
rs79995426
rs57726763
rs7259731
rs3826756
rs35387445
rs217541
rs73062693
rs139937507
rs184545699
rs7250515
rs113346399
rs11881697
rs71365450
rs4806714
rs744759
rs11668642
rs4806516
rs183967007
rs149354341
rs148534450
rs11084325
rs421685
All
0.000
0.005
0.010
0.015
0.020
MR leave−one−out sensitivity analysis for
'LILRB2' on 'PEF'

Figure S69: Results of leave-one-out analyses for FEV1/FVC in the replication MR analyses.

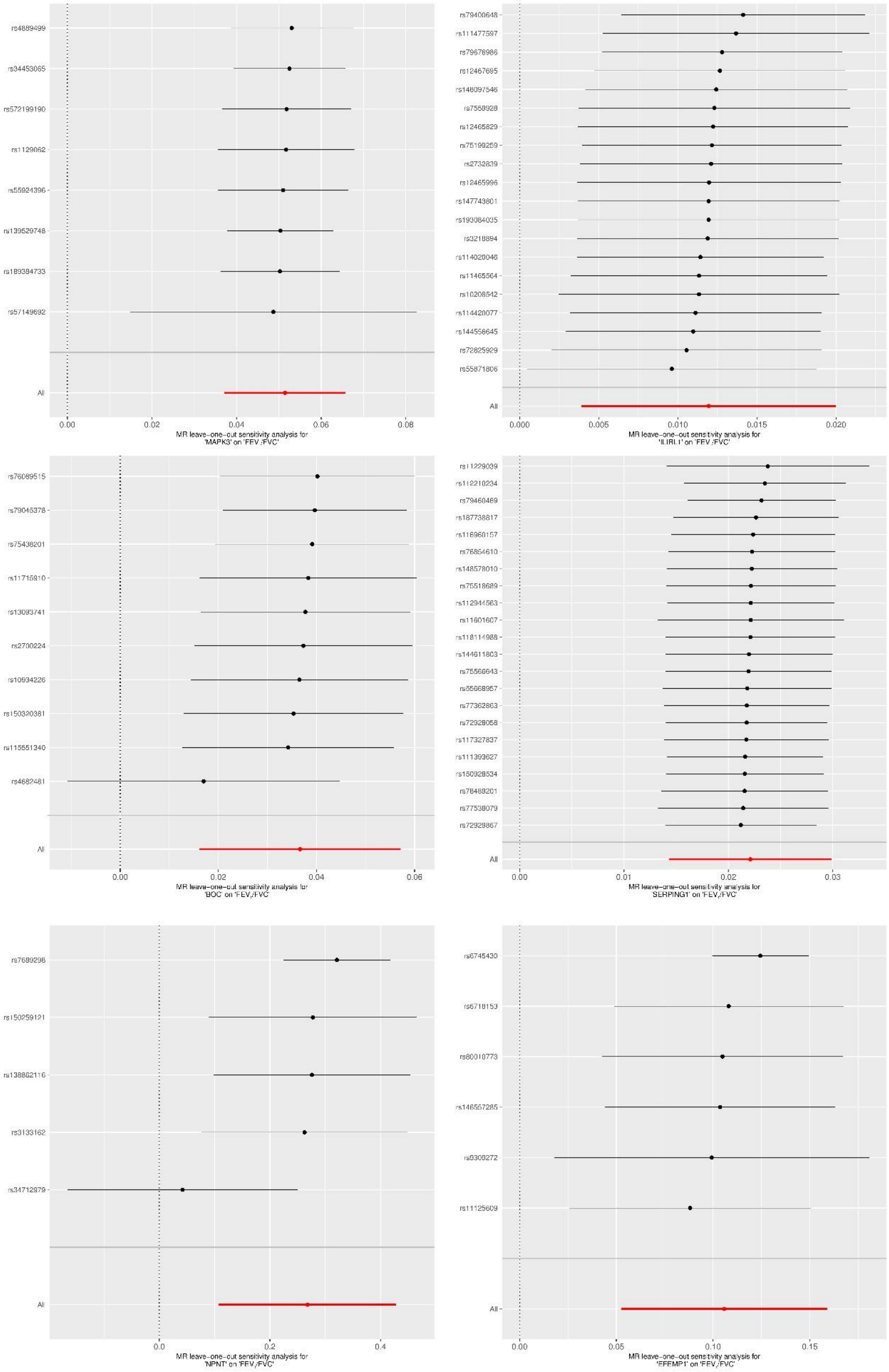

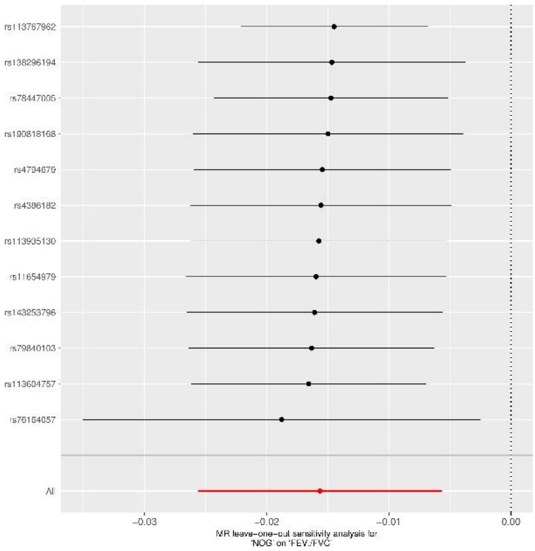

rs113767962
rs138296194
rs78447005
rs190818168
rs4794679
rs4386182
rs113935130
rs11654979
rs143253796
rs79840103
rs113604757
rs76164057
All
−0.03
−0.02
−0.01
0.00
MR leave−one−out sensitivity analysis for
'NOG' on 'FEV1/FVC'

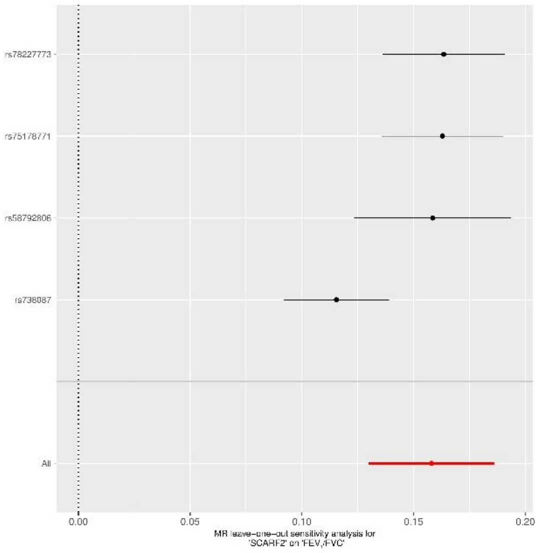

rs78227773
rs75178771
rs58792806
rs738087
All
0.00
0.05
0.10
0.15
0.20
MR leave−one−out sensitivity analysis for
'SCARF2' on 'FEV1/FVC'

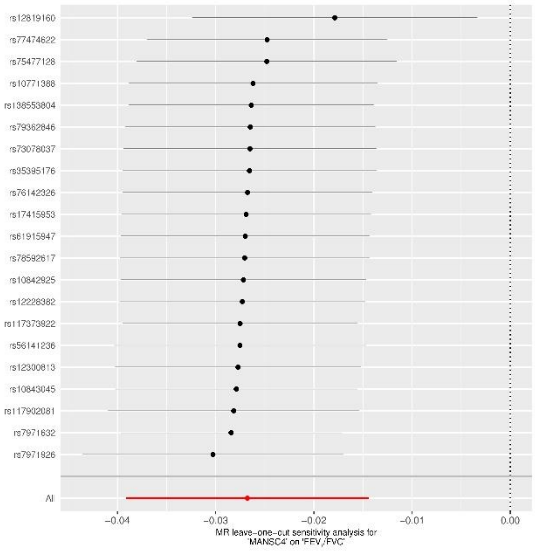

rs12819160
rs77474622
rs75477128
rs10771388
rs138553804
rs79362846
rs73078037
rs35395176
rs76142326
rs17415953
rs61915947
rs78592617
rs10842925
rs12228382
rs117373922
rs56141236
rs12300813
rs10843045
rs117902081
rs7971632
rs7971926
All
−0.04
−0.03
−0.02
−0.01
0.00
MR leave−one−out sensitivity analysis for
'MANSC4' on 'FEV1/FVC'

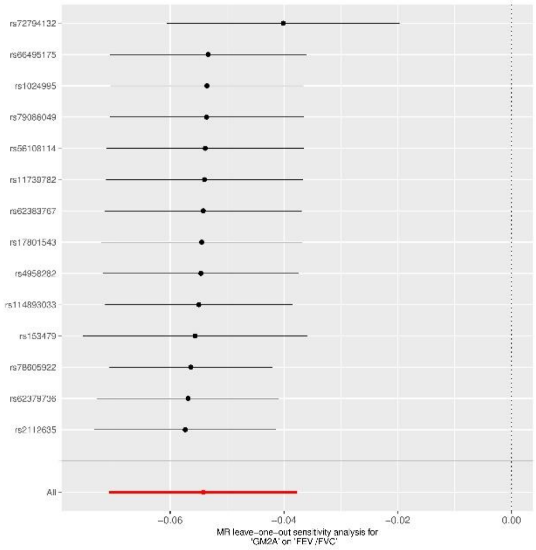

rs72794132
rs66495175
rs1024995
rs79086049
rs56108114
rs11739782
rs62383767
rs17801543
rs4958282
rs114893033
rs153479
rs78605922
rs62379736
rs2112635
All
−0.06
−0.04
−0.02
0.00
MR leave−one−out sensitivity analysis for
'GM2A' on 'FEV1/FVC'

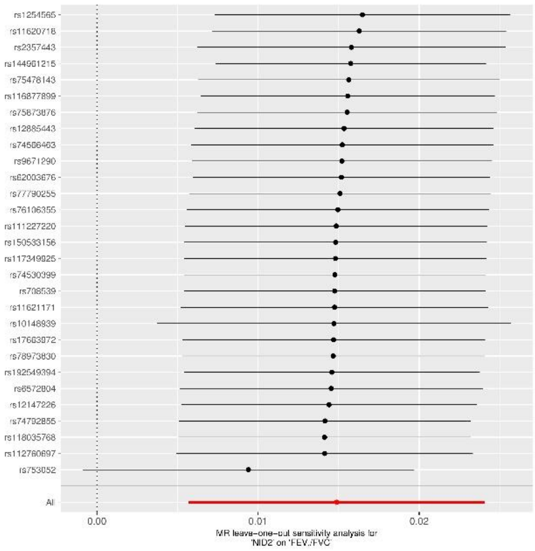

rs1254565
rs11620718
rs2357443
rs144961215
rs75478143
rs116877899
rs75873876
rs12885443
rs74586463
rs9671290
rs62003676
rs77790255
rs76106355
rs111227220
rs150533156
rs117349825
rs74530399
rs708539
rs11621171
rs10148939
rs17663872
rs78973830
rs192549394
rs6572804
rs12147226
rs74792855
rs118035768
rs112760697
rs753052
All
0.00
0.01
0.02
MR leave−one−out sensitivity analysis for
'NID2' on 'FEV1/FVC'

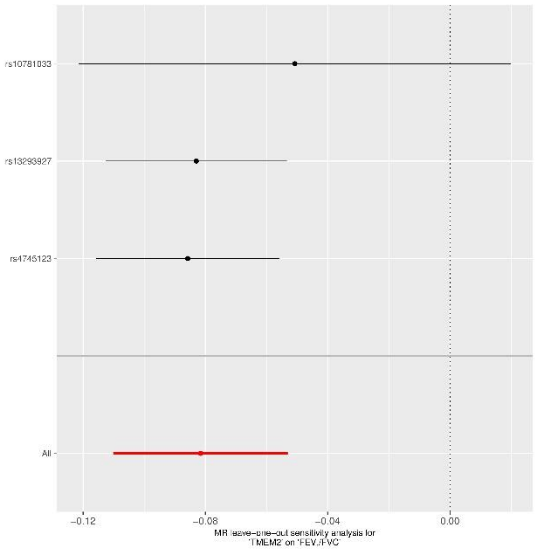

rs10781033
rs13293827
rs4745123
All
−0.12
−0.08
−0.04
0.00
MR leave−one−out sensitivity analysis for
'TMEM2' on 'FEV1/FVC'

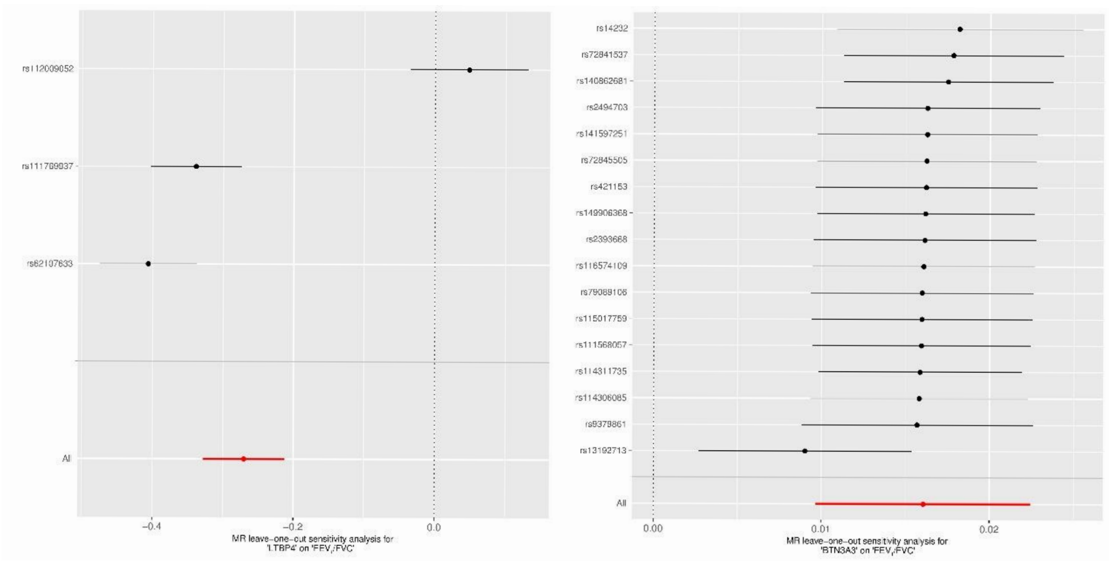
rs112009052
rs111769037
rs62107633
All
−0.4
−0.2
0.0
MR leave−one−out sensitivity analysis for
'LTBP4' on 'FEV1/FVC'
rs14232
rs72841537
rs140862681
rs2494703
rs141597251
rs72845505
rs421153
rs149906368
rs2393668
rs116574109
rs79089106
rs115017759
rs111568057
rs114311735
rs114306085
rs9379861
rs13192713
All
0.00
0.01
0.02
MR leave−one−out sensitivity analysis for
'BTN3A3' on 'FEV1/FVC'

Table S1 Details of the data sources

Trait	Sample Size	Covariate	GWAS catalog	Ancestry	PMID
FVC	N=400,102	UKBB: age, age^2, sex, height,	GCST007429	European	30804560
FEV1	N=400,102	smoking status	GCST007432	European	30804560
FEV1/FVC	N=400,102	SpiroMeta: age, age^2, sex and	GCST007431	European	30804560
PEF	N=345,328	height	GCST007430	European	30804560
Asthma	46,087 cases vs 428,511 controls	sex, age, BMI, assessment center, ethnicity, batch and the first 20 PCs	GCST90038616	NR (U.K.)	33959723
COPD	21,077 cases vs 179,689 controls	age, sex, genotyping array, and the first 10 PCs	GCST90016586	European (U.K.)	33106845
Discovery pQTL	N=7,213	sex, age, study site and 10 genetic PCs		European	35501419
Validation pQTL	N=35,559	sex,age		Iceland	34857953

The summary data for lung function index were from a meta-analysis based on UK Biobank and SpiroMeta. ‘Asthma’ cases were defined by self-reported diseases (field ‘20002’) in UK Biobank. COPD cases were defined based on prebronchodilator spirometry following the modified Global Initiative for Chronic Obstructive Lung Disease criteria for moderate airflow limitation: FEV1 less than 80% of predicted value and the ratio of FEV1/FVC less than 0.7. FEV1: Forced Expired Volume in 1 second, FVC: Forced Vital Capacity, PEF: Peak Expiratory Flow, COPD: Chronic Obstructive Pulmonary Disease, PC: Principal Components.

Table S2 Detail significant results of PWAS

Lung Function	Protein	Gene	CHR	HSQ	BEST.GWAS.ID	PQTL.ID	PWAS.P
FEV1	SeqId_6342_10	NPNT	4	0.1387	rs34712979	rs34712979	1.36e-75
FEV1	SeqId_3186_2	C2	6	0.2212	rs433061	rs115204832	4.43e-13
FEV1	SeqId_10833_64	HHIP	4	0.0287	rs11724319	rs11727676	2.50e-43
FEV1	SeqId_6897_38	B3GAT3	11	0.0641	rs11553576	rs7122950	4.49e-21
FEV1	SeqId_5102_55	MICB	6	0.4333	rs3132470	rs3094011	3.67e-20
FEV1	SeqId_4148_49	PAPPA	9	0.0033	rs10983184	rs10983184	5.11e-14
FEV1	SeqId_17692_2	BTN3A3	6	0.4582	rs68112369	rs9393711	8.39e-14
FEV1	SeqId_17722_5	FKBP4	12	0.1300	rs56196860	rs56196860	1.12e-12
FEV1	SeqId_10974_20	SPINK7	5	0.1395	rs10068891	rs9784645	2.13e-12
FEV1	SeqId_9017_58	LCT	2	0.3410	rs2011946	rs191079	4.40e-12
FEV1	SeqId_8480_29	EFEMP1	2	0.0747	rs3791679	rs3791679	4.42e-12
FEV1	SeqId_3181_50	CTSS	1	0.2077	rs11204675	rs41271951	2.19e-11
FEV1	SeqId_14112_40	RELT	11	0.0061	rs73542929	rs56801796	1.10e-10
FEV1	SeqId_3376_49	IL17RD	3	0.1275	rs9823220	rs6776722	1.62e-10
FEV1	SeqId_18884_22	DNAJB4	1	0.0884	rs1968027	rs7514180	2.00e-10
FEV1	SeqId_15441_6	GM2A	5	0.2528	rs72794132	rs72794132	6.49e-10
FEV1	SeqId_12675_14	DARS	2	0.0326	rs218174	rs2304371	9.25e-10
FEV1	SeqId_4158_54	PLAU	10	0.2607	rs2227552	rs2633322	9.68e-10
FEV1	SeqId_9837_60	NQO1	16	0.1382	rs74029403	rs57488237	1.69e-09
FEV1	SeqId_2849_49	AIF1	6	0.0449	rs2524075	rs3130623	2.22e-09
FEV1	SeqId_8974_172	COL15A1	9	0.1004	rs10760580	rs10819566	2.36e-09
FEV1	SeqId_8956_96	SCARF2	22	0.0798	rs2108746	rs5763025	3.60e-09
FEV1	SeqId_17224_12	OGN	9	0.0668	rs10115290	rs10992291	5.43e-09

FEV1	SeqId_16015_19	GPT	8	0.0114	rs4977190	rs4244610	1.17e-08
FEV1	SeqId_3331_8	RGMB	5	0.0687	rs2545671	rs2368553	3.81e-08
FEV1	SeqId_18922_27	CD68	17	0.0697	rs72827565	rs9901673	4.75e-08
FEV1	SeqId_4125_52	AGER	6	0.1132	rs3130491	rs2070600	5.78e-08
FEV1	SeqId_17761_2	NUDT5	10	0.0066	rs4747968	rs7099650	3.78e-25
FEV1	SeqId_7757_5	HLA-DQA2	6	0.1767	rs9270965	rs9272014	1.56e-07
FEV1	SeqId_9216_100	PLXNB2	22	0.2840	rs73188911	rs28573806	2.61e-07
FEV1	SeqId_3339_33	THBS2	6	0.2337	rs6605526	rs74507247	3.02e-07
FEV1	SeqId_8427_118	RSPO3	6	0.0841	rs6925607	rs1892172	5.74e-07
FEV1	SeqId_15336_7	SELM	22	0.0128	rs17822214	rs117551362	7.69e-07
FEV1	SeqId_13094_75	RSPO3	6	0.0808	rs537875844	rs2154167	9.50e-07
FEV1	SeqId_6451_64	ASPN	9	0.2094	rs2181712	rs2761681	1.12e-06
FEV1	SeqId_19446_1	GMPR2	14	0.1787	rs2766	rs34354104	1.64e-06
FEV1	SeqId_3435_53	FN1	2	0.0432	rs1250258	rs1250258	2.22e-06
FEV1	SeqId_9950_229	LAG3	12	0.0313	rs1882545	rs3782735	3.72e-06
FEV1	SeqId_4407_10	MST1	3	0.4310	rs9858542	rs3197999	6.44e-06
FEV1	SeqId_15513_108	PRSS8	16	0.0124	rs11865499	rs1060506	7.16e-06
FEV1	SeqId_11196_31	COL6A3	2	0.0608	rs2645766	rs1050785	1.13e-05
FEV1	SeqId_3221_54	SFRP1	8	0.0822	rs72643819	rs72643820	1.55e-05
FEV1	SeqId_6431_68	PCYOX1	2	0.3655	rs2706762	rs2706762	1.84e-05
FEV1	SeqId_6927_7	NDST1	5	0.0569	rs11740077	rs11746198	1.91e-05
FEV1	SeqId_5744_12	MENT	1	0.0072	rs61321900	rs34725710	1.94e-05
FEV1	SeqId_12831_21	TESC	12	0.0507	rs7960638	rs10850746	1.95e-05
FEV1	SeqId_4961_17	ANXA2	15	0.0404	rs17845226	rs12440452	1.99e-05
FEV1	SeqId_6462_12	TIMP4	3	0.0758	rs6809597	rs184262	1.99e-05

FEV1	SeqId_17682_1	CD46	1	0.1037	rs2724360	rs2488252	2.50e-05
FEV1	SeqId_4559_64	KYNU	2	0.1265	rs57940394	rs112211045	3.06e-05
FEV1/FVC	SeqId_4125_52	AGER	6	0.1132	rs2070600	rs2070600	9.29e-173
FEV1/FVC	SeqId_6342_10	NPNT	4	0.1387	rs34712979	rs34712979	7.84e-116
FEV1/FVC	SeqId_2849_49	AIF1	6	0.0449	rs137893789	rs3130623	2.71e-99
FEV1/FVC	SeqId_3186_2	C2	6	0.2212	rs9267812	rs115204832	1.96e-13
FEV1/FVC	SeqId_9294_45	MFAP2	1	0.0780	rs3170740	rs761422	1.56e-49
FEV1/FVC	SeqId_7757_5	HLA-DQA2	6	0.1767	rs9272261	rs9272014	9.17e-32
FEV1/FVC	SeqId_13133_73	LTBP4	19	0.0388	rs34093919	rs34093919	1.53e-28
FEV1/FVC	SeqId_4148_49	PAPPA	9	0.0033	rs10983184	rs10983184	9.05e-28
FEV1/FVC	SeqId_11387_3	ATF6B	6	0.1741	rs41268920	rs6449	1.32e-25
FEV1/FVC	SeqId_5102_55	MICB	6	0.4333	rs2256974	rs3094011	8.17e-24
FEV1/FVC	SeqId_10974_20	SPINK7	5	0.1395	rs2400510	rs9784645	1.52e-22
FEV1/FVC	SeqId_8956_96	SCARF2	22	0.0798	rs361566	rs5763025	1.19e-19
FEV1/FVC	SeqId_4209_60	VTA1	6	0.0463	rs263178	rs225628	1.29e-16
FEV1/FVC	SeqId_8480_29	EFEMP1	2	0.0747	rs59985551	rs3791679	1.41e-16
FEV1/FVC	SeqId_10833_64	HHIP	4	0.0287	rs62346060	rs11727676	4.51e-38
FEV1/FVC	SeqId_18884_22	DNAJB4	1	0.0884	rs661038	rs7514180	3.20e-15
FEV1/FVC	SeqId_14112_40	RELT	11	0.0061	rs73542929	rs56801796	3.82e-13
FEV1/FVC	SeqId_17761_2	NUDT5	10	0.0066	rs4747968	rs7099650	8.80e-30
FEV1/FVC	SeqId_3041_55	MRC2	17	0.2726	rs12452590	rs146385050	1.09e-11
FEV1/FVC	SeqId_4234_8	IL1RL1	2	0.2920	rs12470864	rs950880	3.28e-11
FEV1/FVC	SeqId_15441_6	GM2A	5	0.2528	rs72794132	rs72794132	4.37e-11
FEV1/FVC	SeqId_8409_3	RSPO2	8	0.0023	rs640068	rs640068	1.25e-10
FEV1/FVC	SeqId_5581_28	FGL1	8	0.1758	rs10093134	rs3739406	1.68e-10

FEV1/FVC	SeqId_4479_14	SERPING1	11	0.2804	rs76525968	rs10896631	2.32e-10
FEV1/FVC	SeqId_2855_49	MAPK3	16	0.0840	rs61764202	rs9932466	3.93e-10
FEV1/FVC	SeqId_8974_172	COL15A1	9	0.1004	rs10819385	rs10819566	7.80e-10
FEV1/FVC	SeqId_9769_48	DNER	2	0.0654	rs11688074	rs34661502	1.13e-09
FEV1/FVC	SeqId_11196_31	COL6A3	2	0.0608	rs1050785	rs1050785	1.39e-09
FEV1/FVC	SeqId_6402_8	PILRA	7	0.3708	rs35305377	rs1859788	7.64e-09
FEV1/FVC	SeqId_10816_150	PILRA	7	0.4249	rs35305377	rs1859788	8.92e-09
FEV1/FVC	SeqId_7905_30	HP	16	0.2961	rs2287997	rs11648003	2.04e-08
FEV1/FVC	SeqId_3054_3	HP	16	0.4105	rs2287997	rs77303550	2.19e-08
FEV1/FVC	SeqId_8992_1	TMEM2	9	0.0362	rs1410988	rs1410988	4.67e-08
FEV1/FVC	SeqId_4487_1	FGF7	15	0.0968	rs17478785	rs7161744	5.03e-08
FEV1/FVC	SeqId_2813_11	AGRP	16	0.0500	rs28765128	rs114322795	5.69e-08
FEV1/FVC	SeqId_4328_2	BOC	3	0.0711	rs998914	rs3856718	6.47e-08
FEV1/FVC	SeqId_2611_72	TYRO3	15	0.0595	rs9944249	rs1200353	1.10e-07
FEV1/FVC	SeqId_3331_8	RGMB	5	0.0687	rs992592	rs2368553	1.63e-07
FEV1/FVC	SeqId_8778_3	NOG	17	0.0243	rs12951993	rs227715	1.80e-07
FEV1/FVC	SeqId_16781_2	ENGASE	17	0.3110	rs117014247	rs56107536	1.95e-07
FEV1/FVC	SeqId_18319_7	PDHX	11	0.0772	rs2915221	rs2915221	2.13e-07
FEV1/FVC	SeqId_19207_119	ADK	10	0.0108	rs4745724	rs7099085	2.25e-07
FEV1/FVC	SeqId_8269_327	ARSK	5	0.0159	rs2548129	rs956274	2.46e-07
FEV1/FVC	SeqId_9578_263	MANSC4	12	0.1474	rs34344711	rs12368818	4.84e-07
FEV1/FVC	SeqId_15594_47	HTRA1	10	0.0535	rs1810393	rs7074542	5.11e-07
FEV1/FVC	SeqId_12469_19	MAPRE1	20	0.0847	rs853848	rs414049	5.54e-07
FEV1/FVC	SeqId_13423_94	FAM213A	10	0.0071	rs4933379	rs10788623	6.04e-07
FEV1/FVC	SeqId_3376_49	IL17RD	3	0.1275	rs9823220	rs6776722	8.00e-07

FEV1/FVC	SeqId_13488_3	ARFIP1	4	0.0497	rs6830470	rs4619875	1.02e-06
FEV1/FVC	SeqId_17692_2	BTN3A3	6	0.4582	rs68112369	rs9393711	1.28e-06
FEV1/FVC	SeqId_4240_31	PKM2	15	0.0043	rs28379368	rs28379368	2.36e-06
FEV1/FVC	SeqId_13544_9	HMHA1	19	0.0576	rs3752243	rs2240051	4.04e-06
FEV1/FVC	SeqId_7922_5	ADM	11	0.0061	rs11042855	rs2923098	4.26e-06
FEV1/FVC	SeqId_15314_49	CLIC4	1	0.0505	rs9438876	rs4649017	5.08e-06
FEV1/FVC	SeqId_10924_258	CYB5D2	17	0.1055	rs2054038	rs183282334	5.10e-06
FEV1/FVC	SeqId_8296_117	KDELC2	11	0.2874	rs74911261	rs141379009	7.12e-06
FEV1/FVC	SeqId_2743_5	SHH	7	0.0242	rs11773097	rs872723	7.27e-06
FEV1/FVC	SeqId_16060_99	NID2	14	0.1024	rs2516599	rs941622	8.49e-06
FEV1/FVC	SeqId_12524_18	SAT2	17	0.0287	rs12942088	rs13894	8.91e-06
FEV1/FVC	SeqId_3296_92	CNTN2	1	0.3491	rs10900444	rs3753847	9.72e-06
FEV1/FVC	SeqId_3003_29	NCR3	6	0.2762	rs3130632	rs986475	1.15e-05
FEV1/FVC	SeqId_14133_93	IL1R2	2	0.1493	rs1997502	rs2310170	1.23e-05
FEV1/FVC	SeqId_2994_71	IL1RL2	2	0.1156	rs2302621	rs1960510	1.29e-05
FEV1/FVC	SeqId_3481_87	XPNPEP1	10	0.0337	rs2419313	rs3862006	1.32e-05
FEV1/FVC	SeqId_2617_56	ERBB3	12	0.0423	rs7312770	rs2292238	1.33e-05
FEV1/FVC	SeqId_8866_53	QPCTL	19	0.1449	rs17850756	rs17850756	1.37e-05
FEV1/FVC	SeqId_6451_64	ASPN	9	0.2094	rs2761681	rs2761681	1.62e-05
FEV1/FVC	SeqId_16583_8	NUDT2	9	0.3187	rs10972139	rs10971956	3.41e-05
FVC	SeqId_8480_29	EFEMP1	2	0.0747	rs3791679	rs3791679	2.16e-32
FVC	SeqId_3186_2	C2	6	0.2212	rs7776257	rs115204832	3.57e-05
FVC	SeqId_9294_45	MFAP2	1	0.0780	rs3170740	rs761422	1.85e-23
FVC	SeqId_17722_5	FKBP4	12	0.1300	rs56196860	rs56196860	5.24e-18
FVC	SeqId_4125_52	AGER	6	0.1132	rs2070600	rs2070600	6.75e-17

FVC	SeqId_6897_38	B3GAT3	11	0.0641	rs11553576	rs7122950	1.52e-16
FVC	SeqId_6342_10	NPNT	4	0.1387	rs72669966	rs34712979	7.55e-15
FVC	SeqId_9017_58	LCT	2	0.3410	rs2011946	rs191079	1.13e-14
FVC	SeqId_3181_50	CTSS	1	0.2077	rs11204675	rs41271951	1.87e-14
FVC	SeqId_10833_64	HHIP	4	0.0287	rs11727676	rs11727676	1.02e-15
FVC	SeqId_12675_14	DARS	2	0.0326	rs218174	rs2304371	8.95e-12
FVC	SeqId_3041_55	MRC2	17	0.2726	rs12452590	rs146385050	2.51e-11
FVC	SeqId_9769_48	DNER	2	0.0654	rs7592697	rs34661502	7.47e-10
FVC	SeqId_16015_19	GPT	8	0.0114	rs4251691	rs4244610	4.24e-09
FVC	SeqId_17692_2	BTN3A3	6	0.4582	rs12174639	rs9393711	7.27e-09
FVC	SeqId_9837_60	NQO1	16	0.1382	rs60472033	rs57488237	1.86e-08
FVC	SeqId_17764_108	RHOC	1	0.0367	rs7415820	rs7415820	1.83e-07
FVC	SeqId_15584_9	CFHR2	1	0.6689	rs1963607	rs4085749	2.17e-07
FVC	SeqId_3799_11	CA3	8	0.0404	rs1543852	rs1543852	3.02e-07
FVC	SeqId_10396_6	MCL1	1	0.0924	rs4971041	rs190596489	3.08e-07
FVC	SeqId_3339_33	THBS2	6	0.2337	rs9505933	rs74507247	3.43e-07
FVC	SeqId_19523_215	PARK7	1	0.1126	rs12740409	rs17523802	3.87e-07
FVC	SeqId_3617_80	HGFAC	4	0.5248	rs10937929	rs2498323	4.65e-07
FVC	SeqId_4158_54	PLAU	10	0.2607	rs2227552	rs2633322	5.17e-07
FVC	SeqId_4220_39	FER	5	0.0378	rs79536589	rs79536589	5.67e-07
FVC	SeqId_6388_21	CCDC126	7	0.0898	rs13227748	rs35121828	1.14e-06
FVC	SeqId_5676_54	ASIP	20	0.1139	rs62212343	rs6059655	2.12e-06
FVC	SeqId_12831_21	TESC	12	0.0507	rs7960638	rs10850746	2.38e-06
FVC	SeqId_9216_100	PLXNB2	22	0.2840	rs11547731	rs28573806	2.40e-06
FVC	SeqId_5102_55	MICB	6	0.4333	rs2429722	rs3094011	3.71e-06

FVC	SeqId_6431_68	PCYOX1	2	0.3655	rs62152025	rs2706762	3.79e-06
FVC	SeqId_5744_12	MENT	1	0.0072	rs61321900	rs34725710	4.52e-06
FVC	SeqId_3376_49	IL17RD	3	0.1275	rs3968279	rs6776722	4.71e-06
FVC	SeqId_15594_47	HTRA1	10	0.0535	rs55928386	rs7074542	5.03e-06
FVC	SeqId_2849_49	AIF1	6	0.0449	rs137893789	rs3130623	6.27e-06
FVC	SeqId_6527_1	TRIL	7	0.0325	rs740250	rs740250	9.55e-06
FVC	SeqId_17682_1	CD46	1	0.1037	rs2724360	rs2488252	1.10e-05
FVC	SeqId_2644_11	PRKCA	17	0.0988	rs8071250	rs61762372	1.40e-05
FVC	SeqId_19446_1	GMPR2	14	0.1787	rs2766	rs34354104	1.51e-05
FVC	SeqId_9348_1	C1RL	12	0.2853	rs7959658	rs146111161	1.69e-05
FVC	SeqId_9005_16	PLXNA1	3	0.0655	rs76804717	rs9822602	1.77e-05
FVC	SeqId_6927_7	NDST1	5	0.0569	rs11740077	rs11746198	1.80e-05
FVC	SeqId_18922_27	CD68	17	0.0697	rs6608	rs9901673	1.88e-05
FVC	SeqId_9950_229	LAG3	12	0.0313	rs2532503	rs3782735	2.13e-05
FVC	SeqId_17224_12	OGN	9	0.0668	rs1760318	rs10992291	2.17e-05
FVC	SeqId_13094_75	RSPO3	6	0.0808	rs6925607	rs2154167	3.05e-05
FVC	SeqId_8397_147	QSOX2	9	0.1603	rs12684650	rs10858248	3.16e-05
FVC	SeqId_8427_118	RSPO3	6	0.0841	rs6925607	rs1892172	3.44e-05
FVC	SeqId_17761_2	NUDT5	10	0.0066	rs7916879	rs7099650	1.15e-07
PEF	SeqId_2849_49	AIF1	6	0.0449	rs190452998	rs3130623	1.64e-44
PEF	SeqId_6342_10	NPNT	4	0.1387	rs34712979	rs34712979	1.55e-42
PEF	SeqId_3186_2	C2	6	0.2212	rs204999	rs115204832	1.95e-10
PEF	SeqId_4125_52	AGER	6	0.1132	rs9391855	rs2070600	1.13e-37
PEF	SeqId_10833_64	HHIP	4	0.0287	rs11724319	rs11727676	5.76e-70
PEF	SeqId_3196_6	HAPLN1	5	0.0308	rs6886442	rs6886442	1.44e-24

PEF	SeqId_4148_49	PAPPA	9	0.0033	rs10983184	rs10983184	8.28e-23
PEF	SeqId_3363_31	CSK	15	0.0857	rs34933034	rs34933034	1.36e-17
PEF	SeqId_17692_2	BTN3A3	6	0.4582	rs9379851	rs9393711	4.56e-17
PEF	SeqId_9294_45	MFAP2	1	0.0780	rs2284748	rs761422	4.88e-17
PEF	SeqId_5102_55	MICB	6	0.4333	rs3132470	rs3094011	6.51e-16
PEF	SeqId_11387_3	ATF6B	6	0.1741	rs41268920	rs6449	9.13e-16
PEF	SeqId_7757_5	HLA-DQA2	6	0.1767	rs9273072	rs9272014	7.58e-15
PEF	SeqId_8778_3	NOG	17	0.0243	rs10852990	rs227715	4.18e-12
PEF	SeqId_10974_20	SPINK7	5	0.1395	rs2216653	rs9784645	1.41e-10
PEF	SeqId_17761_2	NUDT5	10	0.0066	rs4747968	rs7099650	2.11e-26
PEF	SeqId_18884_22	DNAJB4	1	0.0884	rs1968027	rs7514180	5.96e-10
PEF	SeqId_15526_33	GSS	20	0.1223	rs2424999	rs6088642	1.66e-09
PEF	SeqId_3435_53	FN1	2	0.0432	rs1250258	rs1250258	2.27e-09
PEF	SeqId_12563_2	TNFAIP8	5	0.0723	rs56329027	rs1035376	4.72e-09
PEF	SeqId_3366_51	ECM1	1	0.2616	rs3754217	rs3737240	7.97e-09
PEF	SeqId_10521_10	MXRA8	1	0.0099	rs1240709	rs1240713	1.10e-08
PEF	SeqId_15441_6	GM2A	5	0.2528	rs72794132	rs72794132	9.64e-08
PEF	SeqId_8956_96	SCARF2	22	0.0798	rs362031	rs5763025	4.88e-07
PEF	SeqId_3280_49	ACAN	15	0.0356	rs34949187	rs34949187	4.95e-07
PEF	SeqId_17722_5	FKBP4	12	0.1300	rs56196860	rs56196860	6.54e-07
PEF	SeqId_2730_58	MICA	6	0.4117	rs9263514	rs2523496	2.05e-06
PEF	SeqId_5091_28	LILRB2	19	0.3334	rs4083825	rs383925	2.79e-06
PEF	SeqId_2644_11	PRKCA	17	0.0988	rs9908758	rs61762372	4.49e-06
PEF	SeqId_17765_3	SNUPN	15	0.0601	rs28372916	rs7170787	7.11e-06
PEF	SeqId_4188_1	AKR7A2	1	0.3336	rs2088829	rs11800204	7.40e-06

PEF	SeqId_15376_134	CTSE	1	0.0254	rs11801695	rs78554767	7.59e-06
PEF	SeqId_13133_73	LTBP4	19	0.0388	rs34093919	rs34093919	8.36e-06
PEF	SeqId_12396_19	HIBCH	2	0.1426	rs2582746	rs291466	8.74e-06
PEF	SeqId_15472_16	LRP11	6	0.2421	rs9322193	rs3805753	1.54e-05
PEF	SeqId_3481_87	XPNPEP1	10	0.0337	rs2419313	rs3862006	1.59e-05
PEF	SeqId_8960_3	ERAP2	5	0.2187	rs38033	rs2910686	1.96e-05
PEF	SeqId_2855_49	MAPK3	16	0.0840	rs28529403	rs9932466	2.23e-05
PEF	SeqId_18235_16	PGP	16	0.0973	rs13331451	rs116977380	2.59e-05
PEF	SeqId_15314_49	CLIC4	1	0.0505	rs9438876	rs4649017	2.69e-05
PEF	SeqId_6713_4	LRP11	6	0.2142	rs4816	rs1889471	2.73e-05
PEF	SeqId_12432_23	CACYBP	1	0.0508	rs61828144	rs16847450	2.98e-05
PEF	SeqId_9076_25	PENK	8	0.1014	rs2576594	rs2576581	3.38e-05

Proteins are detected at the at a Benferroni corrected P-value threshold of 0.05/1348. Effects of plasma proteins on lung functions are reported to be beta value, indicating the extent of the quantitative trait changes per one SD change in protein abundance. HSQ is the heritability of the gene, and BEST.GWAS.ID represents the rsID of the most significant GWAS SNP in locus while PQTL.ID is the rsID of the best pOTL in the locus. PWAS.P is the p-value from PWAS.

Table S3 Detail significant results of PWAS for Asthma and COPD

Protein	Gene	Outcome	CHR	HSQ	BEST.GWAS.ID	PQTL.ID	PWAS.P
SeqId_13942_140	SPSB1	Asthma	1	0.00872	rs4908782	rs4908782	1.84E-05
SeqId_15314_49	CLIC4	Asthma	1	0.05049	rs115941677	rs4649017	1.79E-05
SeqId_15602_43	IL6R	Asthma	1	0.38332	rs12133641	rs4129267	6.98E-07
SeqId_14133_93	IL1R2	Asthma	2	0.14928	rs1997502	rs2310170	6.70E-07
SeqId_2991_9	IL1R1	Asthma	2	0.05475	rs12470864	rs2287047	5.07E-07
SeqId_2994_71	IL1RL2	Asthma	2	0.11557	rs2302621	rs1960510	2.33E-10
SeqId_4234_8	IL1RL1	Asthma	2	0.29196	rs12470864	rs950880	1.80E-26
SeqId_16324_38	TLR1	Asthma	4	0.03415	rs5743618	rs5743618	5.84E-19
SeqId_6342_10	NPNT	Asthma	4	0.13865	rs34712979	rs34712979	2.24E-05
SeqId_11387_3	ATF6B	Asthma	6	0.17412	rs2395175	rs6449	9.32E-18
SeqId_2730_58	MICA	Asthma	6	0.41169	rs2844518	rs2523496	1.75E-17
SeqId_3003_29	NCR3	Asthma	6	0.27617	rs3130932	rs986475	1.97E-14
SeqId_3186_2	C2	Asthma	6	0.22122	rs9391858	rs115204832	1.38E-11
SeqId_4125_52	AGER	Asthma	6	0.1132	rs2070600	rs2070600	1.71E-26
SeqId_5102_55	MICB	Asthma	6	0.43328	rs3131642	rs3094011	1.13E-27
SeqId_7757_5	HLA-DQA2	Asthma	6	0.17668	rs2133036	rs9272014	8.13E-16
SeqId_17692_2	BTN3A3	Asthma	6	0.4582	rs9393708	rs9393711	6.35E-07
SeqId_14246_50	DOK2	Asthma	8	0.01841	rs34215892	rs2054713	2.40E-05
SeqId_8255_34	MRVI1	Asthma	11	0.04729	rs4909945	rs1075768	5.12E-06
SeqId_2617_56	ERBB3	Asthma	12	0.04228	rs7312770	rs2292238	1.30E-11
SeqId_15596_7	HEXIM1	Asthma	17	0.02004	rs75016201	rs12051846	2.18E-08
SeqId_18922_27	CD68	Asthma	17	0.06969	rs72827590	rs9901673	1.14E-05
SeqId_9343_16	IL2RB	Asthma	22	0.01255	rs228953	rs228953	2.10E-05

SeqId_10833_64	HHIP	COPD	4	0.0287	rs62346060	rs11727676	6.77E-11
SeqId_6342_10	NPNT	COPD	4	0.13865	rs34712979	rs34712979	1.65E-24
SeqId_2849_49	AIF1	COPD	6	0.04494	rs2524075	rs3130623	9.71E-07
SeqId_3186_2	C2	COPD	6	0.22122	rs433061	rs115204832	1.23E-06
SeqId_4125_52	AGER	COPD	6	0.1132	rs3130491	rs2070600	1.81E-06
SeqId_5102_55	MICB	COPD	6	0.43328	rs3132470	rs3094011	4.74E-09
SeqId_7757_5	HLA-DQA2	COPD	6	0.17668	rs9272251	rs9272014	6.20E-06
SeqId_17761_2	NUDT5	COPD	10	0.00659	rs7916879	rs7099650	1.94E-06

Proteins are detected at the at a Benferroni corrected P-value threshold of 0.05/1348. HSQ is the heritability of the gene, and BEST.GWAS.ID represents the rsID of the most significant GWAS SNP in locus while PQTL.ID is the rsID of the best pOTL in the locus. PWAS.P is the p-value from PWAS.

Table S4 Detail significant results in discovery MR analysis

Lung Function	Protein	Gene	SNPN	F-statistics	PVE	Effect (95% CI)	P value	FDR
FEV1	SeqId_10833_64	HHIP	2	65.8726	0.0179	-0.1631(-0.1869, -0.1393)	4.12e-41	2.14e-39
FEV1	SeqId_10974_20	SPINK7	2	168.2607	0.0446	0.0534(0.0378, 0.0691)	2.31e-11	2.00e-10
FEV1	SeqId_11196_31	COL6A3	5	57.0257	0.0381	-0.0357(-0.0692, -0.0023)	3.63e-02	4.04e-02
FEV1	SeqId_12675_14	DARS	1	401.3465	0.0527	0.0486(0.0321, 0.0650)	6.63e-09	3.54e-08
FEV1	SeqId_12831_21	TESC	3	176.3887	0.0684	-0.0222(-0.0350, -0.0095)	6.18e-04	1.02e-03
FEV1	SeqId_13094_75	RSPO3	4	246.7566	0.1204	0.0241(0.0145, 0.0336)	7.73e-07	2.47e-06
FEV1	SeqId_14112_40	RELT	1	60.4971	0.0083	0.1117(0.0748, 0.1486)	3.01e-09	1.74e-08
FEV1	SeqId_15336_7	SELM	1	55.1246	0.0076	-0.0756(-0.1141, -0.0372)	1.15e-04	2.32e-04
FEV1	SeqId_15441_6	GM2A	8	370.6939	0.2916	-0.0234(-0.0337, -0.0131)	8.68e-06	2.18e-05
FEV1	SeqId_15513_108	PRSS8	1	46.7607	0.0064	-0.1274(-0.1683, -0.0866)	9.63e-10	6.07e-09
FEV1	SeqId_16015_19	GPT	1	69.5305	0.0096	-0.0787(-0.1116, -0.0459)	2.66e-06	7.37e-06
FEV1	SeqId_17224_12	OGN	6	283.1512	0.1908	0.0233(0.0093, 0.0374)	1.15e-03	1.78e-03
FEV1	SeqId_17682_1	CD46	9	37.6390	0.0449	0.0125(0.0053, 0.0198)	6.92e-04	1.12e-03
FEV1	SeqId_17692_2	BTN3A3	7	284.8008	0.2167	0.0200(0.0099, 0.0300)	1.02e-04	2.09e-04
FEV1	SeqId_17722_5	FKBP4	4	161.9969	0.0825	0.0414(0.0299, 0.0530)	1.75e-12	1.66e-11
FEV1	SeqId_17761_2	NUDT5	1	36.1105	0.0050	-0.1106(-0.1555, -0.0657)	1.39e-06	4.26e-06
FEV1	SeqId_18922_27	CD68	3	293.7953	0.1089	-0.0259(-0.0358, -0.0160)	3.15e-07	1.11e-06
FEV1	SeqId_19446_1	GMPR2	4	185.3720	0.0933	-0.0270(-0.0373, -0.0167)	2.83e-07	1.03e-06
FEV1	SeqId_3181_50	CTSS	7	63.7592	0.0583	0.0223(0.0110, 0.0337)	1.18e-04	2.33e-04
FEV1	SeqId_3186_2	C2	8	100.9503	0.1008	-0.0483(-0.0842, -0.0124)	8.34e-03	1.04e-02
FEV1	SeqId_3221_54	SFRP1	5	65.1600	0.0433	-0.0378(-0.0670, -0.0087)	1.10e-02	1.34e-02
FEV1	SeqId_3331_8	RGMB	4	168.4401	0.0855	-0.0368(-0.0541, -0.0195)	3.14e-05	7.02e-05
FEV1	SeqId_3339_33	THBS2	9	230.9489	0.2239	-0.0151(-0.0266, -0.0035)	1.09e-02	1.34e-02

FEV1	SeqId_3376_49	IL17RD	5	55.4456	0.0370	0.0217(0.0074, 0.0361)	2.97e-03	4.11e-03
FEV1	SeqId_3435_53	FN1	3	96.2398	0.0385	0.0437(0.0268, 0.0607)	4.29e-07	1.42e-06
FEV1	SeqId_4125_52	AGER	2	203.0782	0.0533	-0.0395(-0.0520, -0.0271)	4.33e-10	3.10e-09
FEV1	SeqId_4148_49	PAPPA	1	19.8221	0.0027	0.2368(0.1741, 0.2995)	1.36e-13	1.57e-12
FEV1	SeqId_4158_54	PLAU	5	135.7527	0.0861	0.0472(0.0355, 0.0589)	2.54e-15	3.30e-14
FEV1	SeqId_4407_10	MST1	19	103.4160	0.2146	-0.0104(-0.0179, -0.0028	7.07e-03	9.08e-03
FEV1	SeqId_4559_64	KYNU	9	29.3088	0.0353	-0.0146(-0.0212, -0.0079)	1.97e-05	4.61e-05
FEV1	SeqId_4961_17	ANXA2	4	78.1870	0.0416	0.0208(0.0049, 0.0367)	1.03e-02	1.27e-02
FEV1	SeqId_5102_55	MICB	9	229.1481	0.2226	0.0233(0.0062, 0.0403)	7.49e-03	9.50e-03
FEV1	SeqId_6431_68	PCYOX1	7	53.9704	0.0498	-0.0168(-0.0239, -0.0098)	2.53e-06	7.11e-06
FEV1	SeqId_6451_64	ASPN	6	229.2578	0.1603	-0.0195(-0.0314, -0.0076)	1.27e-03	1.93e-03
FEV1	SeqId_6462_12	TIMP4	6	181.2448	0.1311	0.0188(0.0070, 0.0306)	1.76e-03	2.56e-03
FEV1	SeqId_6897_38	B3GAT3	3	25.1405	0.0104	-0.0454(-0.0768, -0.0141)	4.55e-03	6.06e-03
FEV1	SeqId_6927_7	NDST1	3	119.5287	0.0474	-0.0234(-0.0381, -0.0087)	1.78e-03	2.57e-03
FEV1	SeqId_8427_118	RSPO3	6	221.0719	0.1555	0.0222(0.0138, 0.0307)	2.57e-07	9.54e-07
FEV1	SeqId_8480_29	EFEMP1	4	202.2568	0.1009	-0.0388(-0.0594, -0.0181)	2.40e-04	4.34e-04
FEV1	SeqId_8956_96	SCARF2	4	107.6079	0.0564	0.0472(0.0336, 0.0609)	1.21e-11	1.10e-10
FEV1	SeqId_9216_100	PLXNB2	18	51.2575	0.1137	-0.0147(-0.0228, -0.0066)	3.58e-04	6.10e-04
FEV1	SeqId_9950_229	LAG3	1	103.4595	0.0141	-0.0481(-0.0756, -0.0205)	6.34e-04	1.04e-03
FEV1/FVC	SeqId_10816_150	PILRA	10	185.6941	0.2050	-0.0172(-0.0232, -0.0112)	1.62e-08	7.85e-08
FEV1/FVC	SeqId_10833_64	HHIP	2	65.8726	0.0179	-0.1393(-0.1637, -0.1149)	4.25e-29	1.10e-27
FEV1/FVC	SeqId_10924_258	CYB5D2	2	235.6648	0.0614	0.0494(0.0361, 0.0626)	3.03e-13	3.15e-12
FEV1/FVC	SeqId_10974_20	SPINK7	2	168.2607	0.0446	0.0811(0.0654, 0.0969)	5.67e-24	1.18e-22
FEV1/FVC	SeqId_11196_31	COL6A3	5	57.0257	0.0381	-0.0532(-0.0958, -0.0106)	1.43e-02	1.71e-02
FEV1/FVC	SeqId_11387_3	ATF6B	4	91.4617	0.0483	0.1031(0.0506, 0.1555)	1.18e-04	2.32e-04

FEV1/FVC	SeqId_12524_18	SAT2	3	53.4521	0.02184	-0.0418(-0.0638, -0.0198)	1.92e-04	3.54e-04
FEV1/FVC	SeqId_13133_73	LTBP4	2	54.4312	0.0149	-0.1971(-0.2289, -0.1654)	5.24e-34	1.82e-32
FEV1/FVC	SeqId_13423_94	FAM213A	1	30.8668	0.0043	-0.1869(-0.2374, -0.1363)	4.17e-13	4.13e-12
FEV1/FVC	SeqId_13488_3	ARFIP1	2	377.1981	0.0947	0.0267(0.0159, 0.0375)	1.34e-06	4.15e-06
FEV1/FVC	SeqId_14112_40	RELT	1	60.4971	0.0083	0.1162(0.0793, 0.1531)	6.73e-10	4.51e-09
FEV1/FVC	SeqId_14133_93	IL1R2	6	228.6687	0.1599	0.0161(0.0030, 0.0292)	1.59e-02	1.87e-02
FEV1/FVC	SeqId_15314_49	CLIC4	2	213.3329	0.0559	0.0377(0.0242, 0.0513)	4.89e-08	2.12e-07
FEV1/FVC	SeqId_15441_6	GM2A	8	370.6939	0.2916	-0.0268(-0.0360, -0.0175)	1.64e-08	7.76e-08
FEV1/FVC	SeqId_15594_47	HTRA1	5	48.2137	0.0324	-0.0561(-0.1083, -0.0038)	3.56e-02	3.98e-02
FEV1/FVC	SeqId_16060_99	NID2	9	175.3256	0.1797	0.0156(0.0076, 0.0236)	1.44e-04	2.74e-04
FEV1/FVC	SeqId_16583_8	NUDT2	9	215.8704	0.2124	-0.0115(-0.0163, -0.0068)	1.60e-06	4.76e-06
FEV1/FVC	SeqId_16781_2	ENGASE	12	300.3612	0.3336	0.0177(0.0091, 0.0264)	5.71e-05	1.25e-04
FEV1/FVC	SeqId_17692_2	BTN3A3	7	284.8008	0.2167	0.0163(0.0075, 0.0251)	2.72e-04	4.83e-04
FEV1/FVC	SeqId_17761_2	NUDT5	1	36.1105	0.0050	-0.1036(-0.1505, -0.0567)	1.47e-05	3.55e-05
FEV1/FVC	SeqId_18884_22	D-JB4	5	119.3037	0.0764	0.0449(0.0120, 0.0778)	7.53e-03	9.49e-03
FEV1/FVC	SeqId_19207_119	ADK	1	35.7812	0.0049	-0.0654(-0.1128, -0.0179)	6.93e-03	9.13e-03
FEV1/FVC	SeqId_2617_56	ERBB3	2	115.8260	0.0311	0.0236(0.0045, 0.0427)	1.52e-02	1.80e-02
FEV1/FVC	SeqId_2743_5	SHH	2	59.9514	0.0164	0.0568(0.0280, 0.0855)	1.09e-04	2.22e-04
FEV1/FVC	SeqId_2813_11	AGRP	2	145.4630	0.0388	0.0357(0.0196, 0.0518)	1.38e-05	3.38e-05
FEV1/FVC	SeqId_2849_49	AIF1	5	57.9830	0.0387	-0.1798(-0.3421, -0.0175)	2.99e-02	3.38e-02
FEV1/FVC	SeqId_2855_49	MAPK3	12	35.9631	0.0565	0.0245(0.0161, 0.0329)	9.57e-09	4.86e-08
FEV1/FVC	SeqId_2994_71	IL1RL2	7	105.6663	0.0931	0.0270(0.0096, 0.0444)	2.40e-03	3.37e-03
FEV1/FVC	SeqId_3054_3	HP	15	261.3497	0.3526	0.0158(0.0073,0.0242)	2.61e-04	4.68e-04
FEV1/FVC	SeqId_3296_92	CNTN2	12	107.9605	0.1525	-0.0144(-0.0233, -0.0054)	1.70e-03	2.49e-03
FEV1/FVC	SeqId_3331_8	RGMB	4	168.4401	0.0855	-0.0383(-0.0498, -0.0268)	6.51e-11	5.20e-10

FEV1/FVC	SeqId_3376_49	IL17RD	5	55.4456	0.0370	0.0133(0.0052, 0.0215)	1.38e-03	2.06e-03
FEV1/FVC	SeqId_3481_87	XPNPEP1	1	290.9597	0.0388	-0.0411(-0.0583, -0.0239)	2.92e-06	7.99e-06
FEV1/FVC	SeqId_4125_52	AGER	2	203.0782	0.0533	-0.1860(-0.1987, -0.1733)	9.48e-182	1.97e-179
FEV1/FVC	SeqId_4148_49	PAPPA	1	19.8221	0.0027	0.3495(0.2867, 0.4122)	9.25e-28	2.14e-26
FEV1/FVC	SeqId_4234_8	IL1RL1	10	85.1931	0.1058	0.0173(0.0072, 0.0275)	8.24e-04	1.30e-03
FEV1/FVC	SeqId_4240_31	PKM2	1	28.7438	0.0040	0.1011(0.0464, 0.1558)	2.93e-04	5.12e-04
FEV1/FVC	SeqId_4328_2	BOC	4	96.7220	0.0509	0.0485(0.0336, 0.0633)	1.51e-10	1.16e-09
FEV1/FVC	SeqId_4479_14	SERPING1	12	62.8782	0.0949	0.0165(0.0105, 0.0226)	8.46e-08	3.59e-07
FEV1/FVC	SeqId_4487_1	FGF7	3	88.6192	0.0356	0.0290(0.0114, 0.0466)	1.23e-03	1.89e-03
FEV1/FVC	SeqId_5581_28	FGL1	8	106.1808	0.1055	0.0212(0.0087, 0.0337)	8.96e-04	1.40e-03
FEV1/FVC	SeqId_6342_10	NPNT	5	111.1515	0.0716	0.1457(0.0704, 0.2210)	1.50e-04	2.84e-04
FEV1/FVC	SeqId_6402_8	PILRA	14	167.5360	0.2458	-0.0209(-0.0290, -0.0127)	5.84e-07	1.90e-06
FEV1/FVC	SeqId_6451_64	ASPN	6	229.2578	0.1603	-0.0158(-0.0244, -0.0071)	3.45e-04	5.98e-04
FEV1/FVC	SeqId_7905_30	HP	9	197.7981	0.1982	0.0212(0.0133, 0.0291)	1.35e-07	5.29e-07
FEV1/FVC	SeqId_7922_5	ADM	2	25.4995	0.0070	0.0636(0.0242, 0.1029)	1.54e-03	2.27e-03
FEV1/FVC	SeqId_8269_327	ARSK	1	61.2160	0.0084	-0.0733(-0.1100, -0.0367)	8.85e-05	1.88e-04
FEV1/FVC	SeqId_8296_117	KDELC2	6	234.4938	0.1634	-0.0191(-0.0322, -0.0060)	4.33e-03	5.81e-03
FEV1/FVC	SeqId_8409_3	RSPO2	1	20.0471	0.0028	0.2124(0.1495, 0.2752)	3.47e-11	2.89e-10
FEV1/FVC	SeqId_8480_29	EFEMP1	4	202.2568	0.1009	0.0469(0.0198, 0.0739)	6.87e-04	1.12e-03
FEV1/FVC	SeqId_8778_3	NOG	1	100.2441	0.0137	0.0642(0.0324, 0.0959)	7.36e-05	1.60e-04
FEV1/FVC	SeqId_8866_53	QPCTL	7	157.6287	0.1328	-0.0253(-0.0393, -0.0113)	3.99e-04	6.75e-04
FEV1/FVC	SeqId_8956_96	SCARF2	4	107.6079	0.0564	0.0691(0.0551, 0.0832)	5.42e-22	9.39e-21
FEV1/FVC	SeqId_8974_172	COL15A1	6	185.8100	0.1340	0.0306(0.0203, 0.0409)	5.21e-09	2.85e-08
FEV1/FVC	SeqId_8992_1	TMEM2	1	177.6408	0.0240	-0.0605(-0.0817, -0.0392)	2.45e-08	1.13e-07
FEV1/FVC	SeqId_9294_45	MFAP2	6	137.5095	0.1027	-0.0703(-0.082, -0.0586)	6.44e-32	1.91e-30

FEV1/FVC	SeqId_9578_263	MANSC4	11	74.1433	0.1017	-0.0163(-0.0224, -0.0102)	1.61e-07	6.20e-07
FVC	SeqId_10396_6	MCL1	1	336.1462	0.0445	-0.0437(-0.0577, -0.0298)	8.34e-10	5.42e-09
FVC	SeqId_10833_64	HHIP	2	65.8726	0.0179	-0.1054(-0.1293, -0.0815)	5.18e-18	8.28e-17
FVC	SeqId_12675_14	DARS	1	401.3465	0.0527	0.0521(0.0357, 0.0686)	4.71e-10	3.26e-09
FVC	SeqId_12831_21	TESC	3	176.3887	0.0684	-0.0242(-0.0369, -0.0114)	1.98e-04	3.62e-04
FVC	SeqId_13094_75	RSPO3	4	246.7566	0.1204	0.0220(0.0058, 0.0381)	7.59e-03	9.51e-03
FVC	SeqId_15584_9	CFHR2	17	166.3626	0.2822	0.0132(0.0074, 0.0190)	7.98e-06	2.03e-05
FVC	SeqId_16015_19	GPT	1	69.5305	0.0096	-0.0890(-0.1232, -0.0547)	3.71e-07	1.24e-06
FVC	SeqId_17224_12	OGN	6	283.1512	0.1908	0.0168(0.0026, 0.0309)	2.03e-02	2.36e-02
FVC	SeqId_17682_1	CD46	9	37.6390	0.0449	0.0143(0.0070, 0.0216)	1.15e-04	2.30e-04
FVC	SeqId_17692_2	BTN3A3	7	284.8008	0.2167	0.0136(0.0043, 0.0229)	4.27e-03	5.76e-03
FVC	SeqId_17722_5	FKBP4	4	161.9969	0.0825	0.0494(0.0378, 0.0609)	4.64e-17	6.89e-16
FVC	SeqId_17761_2	NUDT5	1	36.1105	0.0050	-0.0697(-0.1146, -0.0248)	2.34e-03	3.31e-03
FVC	SeqId_17764_108	RHOC	2	102.9231	0.0278	0.0556(0.0357, 0.0756)	4.65e-08	2.10e-07
FVC	SeqId_18922_27	CD68	3	293.7953	0.1089	-0.0221(-0.0320, -0.0121)	1.33e-05	3.28e-05
FVC	SeqId_19446_1	GMPR2	4	185.3720	0.0933	-0.0253(-0.0358, -0.0148)	2.19e-06	6.34e-06
FVC	SeqId_19523_215	PARK7	9	34.7031	0.0416	-0.0194(-0.0265, -0.0123)	9.41e-08	3.76e-07
FVC	SeqId_2644_11	PRKCA	5	33.4352	0.0227	-0.0288(-0.0531, -0.0044)	2.05e-02	2.37e-02
FVC	SeqId_3041_55	MRC2	9	97.4304	0.1085	0.0246(0.0131, 0.0361)	2.92e-05	6.59e-05
FVC	SeqId_3181_50	CTSS	7	63.7592	0.0583	0.0253(0.0114, 0.0392)	3.54e-04	6.09e-04
FVC	SeqId_3186_2	C2	8	100.9503	0.1008	-0.0365(-0.0589, -0.0141)	1.39e-03	2.06e-03
FVC	SeqId_3339_33	THBS2	9	230.9489	0.2239	-0.0148(-0.0277, -0.0018)	2.54e-02	2.89e-02
FVC	SeqId_3376_49	IL17RD	5	55.4456	0.0370	0.0173(0.0057, 0.0289)	3.43e-03	4.72e-03
FVC	SeqId_3617_80	HGFAC	11	61.1805	0.0855	0.0136(0.0059, 0.0213)	5.36e-04	8.92e-04
FVC	SeqId_3799_11	CA3	1	298.3973	0.0397	-0.0455(-0.0618, -0.0292)	4.81e-08	2.13e-07

FVC	SeqId_4125_52	AGER	2	203.0782	0.0533	0.0495(0.0369, 0.0622)	1.80e-14	2.20e-13
FVC	SeqId_4158_54	PLAU	5	135.7527	0.0861	0.0340(0.0204, 0.0477)	1.06e-06	3.34e-06
FVC	SeqId_4220_39	FER	3	69.5799	0.0281	0.0513(0.0316, 0.0711)	3.44e-07	1.19e-06
FVC	SeqId_5102_55	MICB	9	229.1481	0.2226	0.0114(0.0015, 0.0213)	2.45e-02	2.80e-02
FVC	SeqId_5676_54	ASIP	2	595.7611	0.1418	0.0212(0.0130, 0.0293)	3.46e-07	1.18e-06
FVC	SeqId_6388_21	CCDC126	5	191.7302	0.1174	-0.0262(-0.0516, -0.0008)	4.29e-02	4.75e-02
FVC	SeqId_6431_68	PCYOX1	7	53.9704	0.0498	-0.0195(-0.0287, -0.0103)	3.57e-05	7.90e-05
FVC	SeqId_6527_1	TRIL	2	86.4571	0.0234	0.0448(0.0240, 0.0655)	2.32e-05	5.29e-05
FVC	SeqId_6927_7	NDST1	3	119.5287	0.0474	-0.0230(-0.0378, -0.0082)	2.32e-03	3.31e-03
FVC	SeqId_8397_147	QSOX2	3	348.5960	0.1267	0.0220(0.0125, 0.0314)	4.99e-06	1.30e-05
FVC	SeqId_8427_118	RSPO3	6	221.0719	0.1555	0.0204(0.0086, 0.0323)	7.47e-04	1.19e-03
FVC	SeqId_8480_29	EFEMP1	4	202.2568	0.1009	-0.0662(-0.0890, -0.0434)	1.23e-08	6.09e-08
FVC	SeqId_9005_16	PLX-1	4	198.8107	0.0994	-0.0212(-0.0319, -0.0106)	9.80e-05	2.04e-04
FVC	SeqId_9216_100	PLXNB2	18	51.2575	0.1137	-0.0126(-0.0196, -0.0055)	4.65e-04	7.80e-04
FVC	SeqId_9294_45	MFAP2	6	137.5095	0.1027	0.0498(0.0365, 0.0630)	1.65e-13	1.80e-12
FVC	SeqId_9348_1	C1RL	2	25.0624	0.0069	-0.0468(-0.0841, -0.0095)	1.39e-02	1.67e-02
FVC	SeqId_9769_48	DNER	8	71.0413	0.0731	0.0381(0.0104, 0.0658)	7.06e-03	9.13e-03
FVC	SeqId_9950_229	LAG3	1	103.4595	0.0141	-0.0363(-0.0639, -0.0088)	9.79e-03	1.21e-02
PEF	SeqId_10521_10	MXRA8	1	56.7214	0.0078	-0.0768(-0.1164, -0.0372)	1.42e-04	2.74e-04
PEF	SeqId_10833_64	HHIP	2	65.8726	0.0179	-0.2151(-0.2407, -0.1895)	6.72e-61	6.99e-59
PEF	SeqId_10974_20	SPINK7	2	168.2607	0.0446	0.0539(0.0371, 0.0707)	3.19e-10	2.37e-09
PEF	SeqId_11387_3	ATF6B	4	91.4617	0.0483	0.0791(0.0493, 0.1089)	1.93e-07	7.29e-07
PEF	SeqId_12396_19	HIBCH	14	125.4556	0.1961	0.0144(0.0039, 0.0249)	6.96e-03	9.11e-03
PEF	SeqId_12432_23	CACYBP	2	459.0801	0.1130	-0.0231(-0.0336, -0.0125)	1.88e-05	4.45e-05
PEF	SeqId_12563_2	TNFAIP8	4	153.0152	0.0783	-0.0302(-0.0581, -0.0023)	3.40e-02	3.82e-02

PEF	SeqId_13133_73	LTBP4	2	54.4312	0.0149	-0.0933(-0.1275, -0.0592)	8.61e-08	3.58e-07
PEF	SeqId_15314_49	CLIC4	2	213.3329	0.0559	0.0282(0.0136, 0.0427)	1.56e-04	2.93e-04
PEF	SeqId_15441_6	GM2A	8	370.6939	0.2916	-0.0222(-0.0315, -0.0128)	3.16e-06	8.44e-06
PEF	SeqId_15472_16	LRP11	18	52.1185	0.1154	-0.0128(-0.0180, -0.0075)	1.69e-06	4.96e-06
PEF	SeqId_15526_33	GSS	4	220.8335	0.1092	0.0374(0.0202, 0.0546)	1.99e-05	4.59e-05
PEF	SeqId_17692_2	BTN3A3	7	284.8008	0.2167	0.0257(0.0129, 0.0384)	8.12e-05	1.74e-04
PEF	SeqId_17722_5	FKBP4	4	161.9969	0.0825	0.0301(0.0164, 0.0438)	1.62e-05	3.88e-05
PEF	SeqId_17761_2	NUDT5	1	36.1105	0.0050	-0.1514(-0.2002, -0.1026)	1.20e-09	7.35e-09
PEF	SeqId_17765_3	SNUPN	3	165.4368	0.0644	0.0204(0.0066, 0.0342)	3.73e-03	5.07e-03
PEF	SeqId_18235_16	PGP	2	245.9564	0.0639	-0.0267(-0.0401, -0.0133)	9.78e-05	2.05e-04
PEF	SeqId_2644_11	PRKCA	5	33.4352	0.0227	-0.0489(-0.0882, -0.0096)	1.48e-02	1.76e-02
PEF	SeqId_2730_58	MICA	12	276.6398	0.3156	-0.0161(-0.0260, -0.0063)	1.36e-03	2.05e-03
PEF	SeqId_2849_49	AIF1	5	57.9830	0.0387	-0.1212(-0.2092, -0.0332)	6.97e-03	9.06e-03
PEF	SeqId_2855_49	MAPK3	12	35.9631	0.0565	0.0175(0.0085, 0.0265)	1.35e-04	2.62e-04
PEF	SeqId_3196_6	HAPLN1	2	55.7960	0.0152	-0.2024(-0.2318, -0.1730)	1.75e-41	1.21e-39
PEF	SeqId_3280_49	ACAN	2	94.9215	0.0257	-0.0659(-0.0875, -0.0444)	2.16e-09	1.28e-08
PEF	SeqId_3435_53	FN1	3	96.2398	0.0385	0.0533(0.0351, 0.0716)	9.33e-09	4.85e-08
PEF	SeqId_3481_87	XPNPEP1	1	290.9597	0.0388	-0.0434(-0.0617, -0.0252)	3.06e-06	8.27e-06
PEF	SeqId_4125_52	AGER	2	203.0782	0.0533	-0.0914(-0.1048, -0.0779)	2.00e-40	8.33e-39
PEF	SeqId_4148_49	PAPPA	1	19.8221	0.0027	0.3315(0.2663, 0.3968)	2.25e-23	4.25e-22
PEF	SeqId_5091_28	LILRB2	8	62.4270	0.0648	0.0183(0.0105, 0.0261)	3.85e-06	1.01e-05
PEF	SeqId_5102_55	MICB	9	229.1481	0.2226	0.0244(0.0006, 0.0483)	4.46e-02	4.91e-02
PEF	SeqId_6342_10	NPNT	5	111.1515	0.0716	0.0918(0.0436, 0.1400)	1.91e-04	3.55e-04
PEF	SeqId_6713_4	LRP11	21	65.2078	0.1600	-0.0119(-0.0164, -0.0073)	3.11e-07	1.11e-06
PEF	SeqId_8778_3	NOG	1	100.2441	0.0137	0.1018(0.0678, 0.1358)	4.45e-09	2.50e-08

PEF	SeqId_8956_96	SCARF2	4	107.6079	0.0564	0.0409(0.0259, 0.0558)	9.28e-08	3.78e-07
PEF	SeqId_8960_3	ERAP2	16	154.1279	0.2552	0.0131(0.0060, 0.0202)	2.79e-04	4.91e-04
PEF	SeqId_9076_25	PENK	12	188.4085	0.2390	-0.0181(-0.0255, -0.0107)	1.53e-06	4.62e-06
PEF	SeqId_9294_45	MFAP2	6	137.5095	0.1027	-0.0464(-0.0574, -0.0354)	1.70e-16	2.36e-15

Proteins are detected at the at an FDR threshold of 0.05. Effects of plasma proteins on lung functions are reported to be beta value, indicating the extent of the quantitative trait changes per one SD change in protein abundance. SNPN represents the number of instrumental variants of corresponding proteins. PVE is the proportion of the protein variance explained by instrumental variants.

Table S5 Detail results of sensitive analysis for discovery MR

Lung Function	Protein	Gene	SNPN	Effect (95% CI)	Egger.P	Intercept.P	R^2.exposure	R^2.outcome	Steiger.P	Effect Direction	Q.P	LOO
FEV1	SeqId_10833_64	HHIP	2	-	-	-	0.0179	4.60e-04	1.48e-21	TRUE	-	-
FEV1	SeqId_10974_20	SPINK7	2	-	-	-	0.0446	1.80e-04	3.78e-64	TRUE	-	-
FEV1	SeqId_11196_31	COL6A3	5	-0.0584(-0.1544, 0.0376)	0.2329	0.6152	0.0381	7.99e-05	9.14e-57	TRUE	0.0048	PASS
FEV1	SeqId_12675_14	DARS	1	-	-	-	0.0527	8.73e-05	1.54e-79	TRUE	-	-
FEV1	SeqId_12831_21	TESC	3	-0.1077(-0.3585, 0.1431)	0.4000	0.5012	0.0684	5.08e-05	1.40e-106	TRUE	0.0183	Excluded
FEV1	SeqId_13094_75	RSPO3	4	0.0059(-0.0379, 0.0496)	0.7923	0.4033	0.1204	6.47e-05	4.91e-195	TRUE	0.5551	PASS
FEV1	SeqId_14112_40	RELT	1	-	-	-	0.0083	8.95e-05	5.16e-12	TRUE	-	-
FEV1	SeqId_15336_7	SELM	1	-	-	-	0.0076	3.73e-05	8.17e-12	TRUE	-	-
FEV1	SeqId_15441_6	GM2A	8	-0.0204(-0.0397, -0.0010)	0.0392	0.7116	0.2916	2.00e-04	0	TRUE	0.0054	PASS
FEV1	SeqId_15513_108	PRSS8	1	-	-	-	0.0064	9.55e-05	2.72e-09	TRUE	-	-
FEV1	SeqId_16015_19	GPT	1	-	-	-	0.0096	5.38e-05	2.30e-14	TRUE	-	-
FEV1	SeqId_17224_12	OGN	6	0.0186(-0.0146, 0.0517)	0.2723	0.7488	0.1908	1.31 e-04	0	TRUE	0.0049	PASS
FEV1	SeqId_17682_1	CD46	9	0.0136(-0.0001, 0.0273)	0.0525	0.8595	0.0449	4.57e-05	7.11e-69	TRUE	0.6076	PASS
FEV1	SeqId_17692_2	BTN3A3	7	0.0251(0.0061, 0.0442)	0.0097	0.5244	0.2167	2.40 e-04	0	TRUE	0.0001	PASS
FEV1	SeqId_17722_5	FKBP4	4	0.0389(0.0230, 0.0548)	1.68e-06	0.6439	0.0825	1.29 e-04	2.33e-126	TRUE	0.8255	PASS
FEV1	SeqId_17761_2	NUDT5	1	-	-	-	0.0050	5.63e-05	1.04e-07	TRUE	-	-
FEV1	SeqId_18922_27	CD68	3	0.0034(-0.0391, 0.0459)	0.8750	0.1546	0.1090	8.08e-05	8.90e-174	TRUE	0.0636	PASS
FEV1	SeqId_19446_1	GMPR2	4	-0.0247(-0.0405, -0.0090)	0.0021	0.7065	0.0933	6.95e-05	2.47e-147	TRUE	0.5884	PASS
FEV1	SeqId_3181_50	CTSS	7	0.0121(-0.0051, 0.0293)	0.1695	0.1415	0.0583	1.55e-04	2.77e-86	TRUE	0.0078	PASS
FEV1	SeqId_3186_2	C2	8	-0.1117(-0.2477, 0.0243)	0.1076	0.3436	0.1008	4.00e-04	2.72e-148	TRUE	1.20e-15	PASS
FEV1	SeqId_3221_54	SFRP1	5	-0.0604(-0.1550, 0.0342)	0.2106	0.6186	0.0433	8.88e-05	1.46e-64	TRUE	0.0092	PASS
FEV1	SeqId_3331_8	RGMB	4	-0.0164(-0.0828, 0.0500)	0.6277	0.5302	0.0855	1.19e-04	9.97e-132	TRUE	0.0773	PASS
FEV1	SeqId_3339_33	THBS2	9	-0.0106(-0.0274, 0.0061)	0.2126	0.4608	0.2239	9.73e-05	0	TRUE	0.0058	PASS

FEV1	SeqId_3376_49	IL17RD	5	0.0167(-0.0152, 0.0485)	0.3045	0.7165	0.0370	1.23e-04	5.98e-54	TRUE	0.0046	PASS
FEV1	SeqId_3435_53	FN1	3	0.0631(0.0095, 0.1167)	0.0210	0.4238	0.0385	7.51e-05	1.27e-57	TRUE	0.1746	PASS
FEV1	SeqId_4125_52	AGER	2	-	-	-	0.0533	1.13e-04	1.26e-79	TRUE	-	-
FEV1	SeqId_4148_49	PAPPA	1	-	-	-	0.0027	1.43e-04	0.000666822	TRUE	-	-
FEV1	SeqId_4158_54	PLAU	5	0.0548(0.0301, 0.0795)	1.34e-05	0.4830	0.0861	1.85e-04	2.50e-130	TRUE	0.3687	PASS
FEV1	SeqId_4407_10	MST1	19	-0.0181(-0.0368, 0.0006)	0.0583	0.3765	0.2146	1.48 e-04	0	TRUE	0.0012	PASS
FEV1	SeqId_4559_64	KYNU	9	-0.0148(-0.0238, -0.0059)	0.0011	0.9260	0.0353	5.71e-05	2.58e-53	TRUE	0.8395	PASS
FEV1	SeqId_4961_17	ANXA2	4	0.0201(-0.0279, 0.0682)	0.4120	0.9751	0.0416	2.32e-05	7.96e-65	TRUE	0.4636	PASS
FEV1	SeqId_5102_55	MICB	9	0.0409(0.0134, 0.0683)	0.0035	0.1247	0.2226	4.25e-04	0	TRUE	1.89e-15	PASS
FEV1	SeqId_6431_68	PCYOX1	7	-0.0143(-0.0268, -0.0018)	0.0247	0.6228	0.0498	7.19e-05	1.54e-75	TRUE	0.4200	PASS
FEV1	SeqId_6451_64	ASPN	6	-0.0266(-0.0525, -0.0007)	0.0438	0.53725	0.1603	7.76e-05	1.53e-267	TRUE	0.0731	PASS
FEV1	SeqId_6462_12	TIMP4	6	0.0136(-0.0157, 0.0430)	0.3631	0.7013	0.1311	6.32e-05	1.91e-214	TRUE	0.1282	PASS
FEV1	SeqId_6897_38	B3GAT3	3	0.2627(-0.1929, 0.7182)	0.2585	0.1783	0.0104	6.43e-05	2.43e-15	TRUE	0.0001	PASS
FEV1	SeqId_6927_7	NDST1	3	-0.0389(-0.1094, 0.0317)	0.2805	0.6132	0.0474	4.56e-05	8.30e-73	TRUE	0.0168	PASS
FEV1	SeqId_8427_118	RSPO3	6	0.0059(-0.0221, 0.0339)	0.6795	0.2300	0.1555	7.46e-05	1.33e-258	TRUE	0.6217	PASS
FEV1	SeqId_8480_29	EFEMP1	4	-0.0468(-0.1287, 0.0351)	0.2623	0.8389	0.1009	1.66e-04	5.61e-156	TRUE	0.0081	PASS
FEV1	SeqId_8956_96	SCARF2	4	0.0650(0.0154, 0.1147)	0.0102	0.4645	0.0564	1.18 e-04	2.71e-84	TRUE	0.4803	PASS
FEV1	SeqId_9216_100	PLXNB2	18	-0.0050(-0.0189, 0.0088)	0.4769	0.1000	0.1137	1.42 e-04	5.48e-179	TRUE	0.0140	PASS
FEV1	SeqId_9950_229	LAG3	1	-	-	-	0.0141	2.85e-05	7.40e-22	TRUE	-	-
FEV1/FVC	SeqId_10816_150	PILRA	10	-0.0217(-0.0376, -0.0057)	0.0078	0.5523	0.2050	1.23e-04	0	TRUE	0.2971	PASS
FEV1/FVC	SeqId_10833_64	HHIP	2	-	-	-	0.0179	3.83e-04	3.18e-22	TRUE	-	-
FEV1/FVC	SeqId_10924_258	CYB5D2	2	-	-	-	0.0614	1.82e-04	2.44e-90	TRUE	-	-
FEV1/FVC	SeqId_10974_20	SPINK7	2	-	-	-	0.0446	3.82e-04	2.06e-60	TRUE	-	-
FEV1/FVC	SeqId_11196_31	COL6A3	5	-0.1082(-0.2172, 0.0008)	0.0517	0.2843	0.0381	1.50e-04	7.48e-55	TRUE	9.21e-05	PASS
FEV1/FVC	SeqId_11387_3	ATF6B	4	0.0169(-0.1724, 0.2063)	0.8609	0.3526	0.0483	5.12e-04	4.73e-64	TRUE	1.95e-07	PASS

FEV1/FVC	SeqId_12524_18	SAT2	3	-0.0132(-0.0584, 0.0320)	0.5671	0.1559	0.0218	3.91e-05	4.51e-33	TRUE	0.3441	PASS
FEV1/FVC	SeqId_13133_73	LTBP4	2	-	-	-	0.0149	4.69e-04	2.02e-17	TRUE	-	-
FEV1/FVC	SeqId_13423_94	FAM213A	1	-	-	-	0.0043	1.34e-04	5.97e-06	TRUE	-	-
FEV1/FVC	SeqId_13488_3	ARFIP1	2	-	-	-	0.0947	5.75e-05	1.75e-150	TRUE	-	-
FEV1/FVC	SeqId_14112_40	RELT	1	-	-	-	0.0083	9.30e-05	5.75e-12	TRUE	-	-
FEV1/FVC	SeqId_14133_93	IL1R2	6	0.0187(-0.0049, 0.0422)	0.1205	0.7874	0.1600	6.70e-05	1.11e-267	TRUE	0.0297	PASS
FEV1/FVC	SeqId_15314_49	CLIC4	2	-	-	-	0.0559	7.60e-05	4.69e-85	TRUE	-	-
FEV1/FVC	SeqId_15441_6	GM2A	8	-0.0191(-0.0351, -0.0030)	0.0197	0.2536	0.2916	2.17e-04	0	TRUE	0.0326	PASS
FEV1/FVC	SeqId_15594_47	HTRA1	5	-0.0477(-0.5222, 0.4267)	0.8437	0.9723	0.0324	1.64e-04	6.18e-46	TRUE	2.84e-06	PASS
FEV1/FVC	SeqId_16060_99	NID2	9	0.0213(0.0076, 0.0350)	0.0023	0.3116	0.1797	5.14e-05	2.25e-307	TRUE	0.6339	PASS
FEV1/FVC	SeqId_16583_8	NUDT2	9	-0.0071(-0.0171, 0.0029)	0.1645	0.3189	0.2124	6.42e-05	0	TRUE	0.9409	PASS
FEV1/FVC	SeqId_16781_2	ENGASE	12	0.0176(0.0047, 0.0304)	0.0073	0.9677	0.3336	1.46e-04	0	TRUE	0.0141	PASS
FEV1/FVC	SeqId_17692_2	BTN3A3	7	0.0124(-0.0045, 0.0294)	0.1510	0.5903	0.2167	1.63e-04	0	TRUE	0.0021	PASS
FEV1/FVC	SeqId_17761_2	NUDT5	1	-	-	-	0.0050	4.80e-05	7.97e-08	TRUE	-	-
FEV1/FVC	SeqId_18884_22	D-JB4	5	-0.0550(-0.1602, 0.0501)	0.3051	0.0549	0.0764	2.04e-04	6.30e-114	TRUE	6.41e-06	PASS
FEV1/FVC	SeqId_19207_119	ADK	1	-	-	-	0.0049	1.85e-05	2.68e-08	TRUE	-	-
FEV1/FVC	SeqId_2617_56	ERBB3	2	-	-	-	0.0311	4.75e-05	3.68e-47	TRUE	-	-
FEV1/FVC	SeqId_2743_5	SHH	2	-	-	-	0.0164	4.18e-05	8.86e-25	TRUE	-	-
FEV1/FVC	SeqId_2813_11	AGRP	2	-	-	-	0.0388	1.07e-04	4.42e-57	TRUE	-	-
FEV1/FVC	SeqId_2849_49	AIF1	5	-0.0901(-0.5560, 0.3757)	0.7045	0.6819	0.0387	2.29e-04	3.72e-37	TRUE	5.89e-87	PASS
FEV1/FVC	SeqId_2855_49	MAPK3	12	0.0229(0.0055, 0.0403)	0.0098	0.8349	0.0565	1.55e-04	1.89e-83	TRUE	0.1489	PASS
FEV1/FVC	SeqId_2994_71	IL1RL2	7	0.0196(-0.0213, 0.0604)	0.3479	0.6883	0.0931	9.37e-05	1.05e-145	TRUE	0.0264	PASS
FEV1/FVC	SeqId_3054_3	HP	15	0.0172(-0.0120, 0.0463)	0.2491	0.9215	0.3526	1.50e-04	0	TRUE	0.0070	PASS
FEV1/FVC	SeqId_3296_92	CNTN2	12	-0.0169(-0.0344, 0.0005)	0.0570	0.7334	0.1525	1.28e-04	9.89e-250	TRUE	0.0060	PASS
FEV1/FVC	SeqId_3331_8	RGMB	4	-0.0270(-0.0685, 0.0145)	0.2029	0.5768	0.0855	1.14e-04	6.24e-132	TRUE	0.4663	PASS

FEV1/FVC	SeqId_3376_49	IL17RD	5	0.0117(-0.0067, 0.0300)	0.2140	0.8360	0.0370	4.30e-05	1.45e-56	TRUE	0.3049	PASS
FEV1/FVC	SeqId_3481_87	XPNPEP1	1	-	-	-	0.0388	5.59e-05	9.25e-59	TRUE	-	-
FEV1/FVC	SeqId_4125_52	AGER	2	-	-	-	0.0533	2.19e-03	1.47e-56	TRUE	-	-
FEV1/FVC	SeqId_4148_49	PAPPA	1	-	-	-	0.0027	3.01e-04	3.19e-03	TRUE	-	-
FEV1/FVC	SeqId_4234_8	IL1RL1	10	0.0285(0.0075, 0.0496)	0.0078	0.2368	0.1058	1.31e-04	9.87e-166	TRUE	0.0061	PASS
FEV1/FVC	SeqId_4240_31	PKM2	1	-	-	-	0.0040	3.27e-05	1.37e-06	TRUE	-	-
FEV1/FVC	SeqId_4328_2	BOC	4	0.0535(-0.0056, 0.1126)	0.0759	0.8629	0.0509	1.06e-04	4.39e-76	TRUE	0.6647	PASS
FEV1/FVC	SeqId_4479_14	SERPING1	12	0.0201(0.0098, 0.0303)	0.0001	0.3976	0.0949	1.36e-04	7.98e-147	TRUE	0.1940	PASS
FEV1/FVC	SeqId_4487_1	FGF7	3	0.0472(-0.0183, 0.1127)	0.1579	0.5716	0.0356	2.75e-05	5.08e-55	TRUE	0.7020	PASS
FEV1/FVC	SeqId_5581_28	FGL1	8	0.0321(0.0111, 0.0531)	0.0028	0.2154	0.1055	1.73e-04	1.52e-163	TRUE	0.00028	PASS
FEV1/FVC	SeqId_6342_10	NPNT	5	0.2251(0.1221, 0.3280)	1.82e-05	0.0641	0.0716	1.66e-03	6.16e-86	TRUE	2.29e-29	PASS
FEV1/FVC	SeqId_6402_8	PILRA	14	-0.0294(-0.0567, -0.0020)	0.0351	0.5201	0.2458	2.35e-04	0	TRUE	0.0026	PASS
FEV1/FVC	SeqId_6451_64	ASPN	6	-0.0280(-0.0455, -0.0106)	0.0016	0.1126	0.1603	4.46e-05	2.88e-270	TRUE	0.4971	PASS
FEV1/FVC	SeqId_7905_30	HP	9	0.0386(0.0169, 0.0603)	0.0005	0.0930	0.1982	9.54e-05	0	TRUE	0.3913	PASS
FEV1/FVC	SeqId_7922_5	ADM	2	-	-	-	0.0070	2.87e-05	3.61e-11	TRUE	-	-
FEV1/FVC	SeqId_8269_327	ARSK	1	-	-	-	0.0084	3.83e-05	5.14e-13	TRUE	-	-
FEV1/FVC	SeqId_8296_117	KDELC2	6	-0.0196(-0.0403, 0.0012)	0.0648	0.9489	0.1634	7.25e-05	9.23e-274	TRUE	0.0509	PASS
FEV1/FVC	SeqId_8409_3	RSPO2	1	-	-	-	0.0028	1.10e-04	3.83e-04	TRUE	-	-
FEV1/FVC	SeqId_8480_29	EFEMP1	4	0.0984(0.0209, 0.1758)	0.0128	0.1709	0.1009	2.41e-04	2.09e-153	TRUE	0.0002	PASS
FEV1/FVC	SeqId_8778_3	NOG	1	-	-	-	0.0137	4.14e-05	8.05e-21	TRUE	-	-
FEV1/FVC	SeqId_8866_53	QPCTL	7	-0.0423(-0.0682, -0.0165)	0.0013	0.1373	0.1328	1.07e-04	1.03e-214	TRUE	0.0277	PASS
FEV1/FVC	SeqId_8956_96	SCARF2	4	0.0996(0.0492, 0.1500)	0.0001	0.2170	0.0564	2.35e-04	4.06e-81	TRUE	0.6373	PASS
FEV1/FVC	SeqId_8974_172	COL15A1	6	0.0475(0.0207, 0.0744)	0.0005	0.1849	0.1340	1.25e-04	7.47e-216	TRUE	0.2841	PASS
FEV1/FVC	SeqId_8992_1	TMEM2	1	-	-	-	0.0240	7.61e-05	1.99e-35	TRUE	-	-
FEV1/FVC	SeqId_9294_45	MFAP2	6	-0.0866(-0.1107, -0.0625)	1.87e-12	0.1396	0.1027	4.46e-04	4.13e-151	TRUE	0.2939	PASS

FEV1/FVC	SeqId_9578_263	MANSC4	11	-0.0163(-0.0267, -0.0060)	0.0019	0.9910	0.1017	9.10e-05	1.09e-160	TRUE	0.4720	PASS
FVC	SeqId_10396_6	MCL1	1	-	-	-	0.0445	9.54e-05	2.30e-66	TRUE	-	-
FVC	SeqId_10833_64	HHIP	2	-	-	-	0.0179	1.87e-04	2.19e-24	TRUE	-	-
FVC	SeqId_12675_14	DARS	1	-	-	-	0.0527	1.00e-04	4.40e-79	TRUE	-	-
FVC	SeqId_12831_21	TESC	3	-0.1138(-0.3766, 0.1490)	0.3959	0.5007	0.0684	5.81e-05	3.47e-106	TRUE	0.0124	Excluded
FVC	SeqId_13094_75	RSPO3	4	-0.0238(-0.0843, 0.0367)	0.4402	0.1280	0.1204	6.85e-05	8.92e-195	TRUE	0.0476	PASS
FVC	SeqId_15584_9	CFHR2	17	0.0114(-0.0011, 0.0238)	0.0735	0.7373	0.2822	1.41e-04	0	TRUE	0.0707	PASS
FVC	SeqId_16015_19	GPT	1	-	-	-	0.0096	6.79e-05	4.15e-14	TRUE	-	-
FVC	SeqId_17224_12	OGN	6	0.0093(-0.0234, 0.0420)	0.5783	0.6115	0.1908	8.81e-05	0	TRUE	0.0045	PASS
FVC	SeqId_17682_1	CD46	9	0.0150(0.0003, 0.0298)	0.0459	0.9123	0.0449	5.86e-05	2.68e-68	TRUE	0.4249	PASS
FVC	SeqId_17692_2	BTN3A3	7	0.0210(0.0044, 0.0376)	0.01307	0.2892	0.2167	1.36e-04	0	TRUE	0.00074	PASS
FVC	SeqId_17722_5	FKBP4	4	0.0489(0.0330, 0.0648)	1.68e-09	0.9362	0.0825	1.80e-04	1.46e-124	TRUE	0.8073	PASS
FVC	SeqId_17761_2	NUDT5	1	-	-	-	0.0050	2.21e-05	2.78e-08	TRUE	-	-
FVC	SeqId_17764_108	RHOC	2	-	-	-	0.0278	7.83e-05	5.50e-41	TRUE	-	-
FVC	SeqId_18922_27	CD68	3	0.0023(-0.0378, 0.0424)	0.9105	0.2101	0.1089	5.82e-05	3.57e-175	TRUE	0.1243	PASS
FVC	SeqId_19446_1	GMPR2	4	-0.0222(-0.0382, -0.0062)	0.0064	0.6112	0.0933	6.06e-05	7.41e-148	TRUE	0.5889	PASS
FVC	SeqId_19523_215	PARK7	9	-0.0217(-0.0317, -0.0116)	2.24e-05	0.5261	0.0416	1.06e-04	2.01e-61	TRUE	0.9213	PASS
FVC	SeqId_2644_11	PRKCA	5	-0.0725(-0.1489, 0.0038)	0.0626	0.2361	0.0227	2.01e-05	2.96e-35	TRUE	0.6364	PASS
FVC	SeqId_3041_55	MRC2	9	0.0090(-0.0290, 0.0470)	0.6410	0.3986	0.1085	8.18e-05	7.29e-173	TRUE	0.2608	PASS
FVC	SeqId_3181_50	CTSS	7	0.0140(-0.0078, 0.0359)	0.2083	0.2045	0.0583	2.05e-04	6.38e-85	TRUE	0.0002	PASS
FVC	SeqId_3186_2	C2	8	-0.0679(-0.1551, 0.0193)	0.1270	0.4641	0.1008	2.03e-04	1.90e-154	TRUE	2.67e-05	PASS
FVC	SeqId_3339_33	THBS2	9	-0.0102(-0.0291, 0.0086)	0.2872	0.5018	0.2239	1.05e-04	0	TRUE	0.0011	PASS
FVC	SeqId_3376_49	IL17RD	5	0.0126(-0.0129, 0.0382)	0.3329	0.6749	0.0370	7.87e-05	3.15e-55	TRUE	0.0435	PASS
FVC	SeqId_3617_80	HGFAC	11	0.0119(0.0011, 0.0228)	0.0315	0.6543	0.0855	1.23e-04	1.29e-131	TRUE	0.0122	PASS
FVC	SeqId_3799_11	CA3	1	-	-	-	0.0397	7.51e-05	1.54e-59	TRUE	-	-

FVC	SeqId_4125_52	AGER	2	-	-	-	0.0533	1.54e-04	2.16e-78	TRUE	-	-
FVC	SeqId_4158_54	PLAU	5	0.0460(0.0185, 0.0734)	0.0010	0.3249	0.0861	1.03e-04	2.21e-133	TRUE	0.2135	PASS
FVC	SeqId_4220_39	FER	3	0.0838(0.0387, 0.1288)	0.0003	0.1171	0.0281	7.14e-05	9.11e-42	TRUE	0.26014	PASS
FVC	SeqId_5102_55	MICB	9	0.0160(-0.002, 0.0339)	0.0816	0.5401	0.2226	1.21e-04	0	TRUE	0.0003	PASS
FVC	SeqId_5676_54	ASIP	2	-	-	-	0.1418	7.75e-05	4.84e-233	TRUE	-	-
FVC	SeqId_6388_21	CCDC126	5	-0.0094(-0.0590, 0.0402)	0.7113	0.4293	0.1174	1.39e-04	1.16e-185	TRUE	1.58e-05	PASS
FVC	SeqId_6431_68	PCYOX1	7	-0.0134(-0.0288, 0.0021)	0.0907	0.3300	0.0498	1.02e-04	1.90e-74	TRUE	0.1067	PASS
FVC	SeqId_6527_1	TRIL	2	-	-	-	0.0234	5.16e-05	3.44e-35	TRUE	-	-
FVC	SeqId_6927_7	NDST1	3	-0.0501(-0.1215, 0.0214)	0.1697	0.3816	0.0474	5.23e-05	1.72e-72	TRUE	0.0031	PASS
FVC	SeqId_8397_147	QSOX2	3	0.0318(-0.0983, 0.1619)	0.6320	0.8635	0.1267	1.84e-04	4.18e-200	TRUE	8.30e-12	PASS
FVC	SeqId_8427_118	RSPO3	6	-0.0069(-0.0395, 0.0258)	0.6790	0.0848	0.1555	7.68e-05	1.92e-258	TRUE	0.1027	PASS
FVC	SeqId_8480_29	EFEMP1	4	-0.1055(-0.1766, -0.0344)	0.0036	0.2560	0.1009	4.29e-04	2.01e-148	TRUE	0.0025	PASS
FVC	SeqId_9005_16	PLX-1	4	-0.0173(-0.0493, 0.0146)	0.2882	0.7925	0.0994	4.60e-05	2.87e-159	TRUE	0.3755	PASS
FVC	SeqId_9216_100	PLXNB2	18	-0.0043(-0.0165, 0.0078)	0.4848	0.1083	0.1137	1.05e-04	1.03e-180	TRUE	0.1074	PASS
FVC	SeqId_9294_45	MFAP2	6	0.0641(0.0341, 0.0941)	2.85e-05	0.2986	0.1027	2.43e-04	1.79e-156	TRUE	0.1352	PASS
FVC	SeqId_9348_1	C1RL	2	-	-	-	0.0069	1.51e-05	2.36e-11	TRUE	-	-
FVC	SeqId_9769_48	DNER	8	0.0515(-0.0326, 0.1357)	0.2301	0.7382	0.0731	1.83e-04	3.78e-109	TRUE	8.18e-06	PASS
FVC	SeqId_9950_229	LAG3	1	-	-	-	0.0141	1.63e-05	2.53e-22	TRUE	-	-
PEF	SeqId_10521_10	MXRA8	1	-	-	-	0.0078	4.26e-05	5.39e-12	TRUE	-	-
PEF	SeqId_10833_64	HHIP	2	-	-	-	0.0179	8.09e-04	4.07e-19	TRUE	-	-
PEF	SeqId_10974_20	SPINK7	2	-	-	-	0.0446	1.76e-04	4.27e-64	TRUE	-	-
PEF	SeqId_11387_3	ATF6B	4	0.0438(-0.0738, 0.1614)	0.4653	0.5392	0.0483	2.82e-04	1.49e-67	TRUE	0.0230	PASS
PEF	SeqId_12396_19	HIBCH	14	0.0070(-0.0114, 0.0255)	0.4546	0.3432	0.1961	1.88e-04	0	TRUE	7.36e-05	PASS
PEF	SeqId_12432_23	CACYBP	2	-	-	-	0.1130	5.24e-05	3.98e-182	TRUE	-	-
PEF	SeqId_12563_2	TNFAIP8	4	0.0010 (-0.0703, 0.0723)	0.9780	0.3496	0.0783	1.05e-04	5.36e-120	TRUE	0.0022	PASS

PEF	SeqId_13133_73	LTBP4	2	-	-	-	0.01487	9.92e-05	2.97e-21	TRUE	-	-
PEF	SeqId_15314_49	CLIC4	2	-	-	-	0.0559	4.32e-05	2.38e-86	TRUE	-	-
PEF	SeqId_15441_6	GM2A	8	-0.0174(-0.0345, -0.0004)	0.0455	0.5086	0.2916	1.65e-04	0	TRUE	0.0469	PASS
PEF	SeqId_15472_16	LRP11	18	-0.0162(-0.0251, -0.0072)	0.0004	0.3582	0.1154	1.15e-04	1.13e-182	TRUE	0.4394	PASS
PEF	SeqId_15526_33	GSS	4	0.0469(-0.0009, 0.0947)	0.0546	0.6682	0.1092	1.53e-04	3.27e-170	TRUE	0.0595	PASS
PEF	SeqId_17692_2	BTN3A3	7	0.0303(0.0056, 0.0550)	0.0164	0.6606	0.2167	3.91e-04	0	TRUE	1.18e-06	PASS
PEF	SeqId_17722_5	FKBP4	4	0.0303(0.0071, 0.0536)	0.0105	0.9763	0.0825	8.05e-05	4.02e-128	TRUE	0.2776	PASS
PEF	SeqId_17761_2	NUDT5	1	-	-	-	0.0050	1.05 e-04	3.77e-07	TRUE	-	-
PEF	SeqId_17765_3	SNUPN	3	0.0244(-0.0432, 0.0920)	0.4796	0.8952	0.0644	4.06e-05	2.16e-100	TRUE	0.0527	PASS
PEF	SeqId_18235_16	PGP	2	-	-	-	0.0639	4.47e-05	2.95e-99	TRUE	-	-
PEF	SeqId_2644_11	PRKCA	5	-0.0805(-0.2195, 0.0585)	0.2563	0.6384	0.0227	6.87e-05	1.86e-33	TRUE	0.0510	PASS
PEF	SeqId_2730_58	MICA	12	0.0079(-0.0229, 0.0387)	0.6149	0.1089	0.3156	2.43e-04	0	TRUE	0.0132	PASS
PEF	SeqId_2849_49	AIF1	5	-0.0141(-0.2439, 0.2156)	0.9040	0.3227	0.0387	8.78e-04	4.27e-46	TRUE	1.16e-22	PASS
PEF	SeqId_2855_49	MAPK3	12	0.0093(-0.0084, 0.0269)	0.3034	0.2896	0.0565	1.07e-04	9.87e-85	TRUE	0.1497	PASS
PEF	SeqId_3196_6	HAPLN1	2	-	-	-	0.0152	6.45e-04	1.11e-16	TRUE	-	-
PEF	SeqId_3280_49	ACAN	2	-	-	-	0.0257	1.54e-04	4.81e-36	TRUE	-	-
PEF	SeqId_3435_53	FN1	3	0.0831(0.0435, 0.1226)	3.87e-05	0.0972	0.0385	1.07e-04	1.66e-56	TRUE	0.2405	PASS
PEF	SeqId_3481_87	XPNPEP1	1	-	-	-	0.0388	6.38e-05	2.62e-58	TRUE	-	-
PEF	SeqId_4125_52	AGER	2	-	-	-	0.0533	5.92e-04	3.03e-70	TRUE	-	-
PEF	SeqId_4148_49	PAPPA	1	-	-	-	0.0027	2.80e-04	2.72e-3	TRUE	-	-
PEF	SeqId_5091_28	LILRB2	8	0.0155(0.0040, 0.0269)	0.0082	0.4911	0.0648	1.46e-04	1.11e-96	TRUE	0.0856	PASS
PEF	SeqId_5102_55	MICB	9	0.0471(0.0077, 0.0865)	0.0192	0.1691	0.2226	6.65e-04	0	TRUE	1.73e-28	PASS
PEF	SeqId_6342_10	NPNT	5	0.1409(0.0723, 0.2096)	5.76e-05	0.0861	0.0716	7.05e-04	3.16e-96	TRUE	6.97e-11	PASS
PEF	SeqId_6713_4	LRP11	21	-0.015(-0.0241, -0.0059)	0.0012	0.4369	0.1600	1.30e-04	6.36e-263	TRUE	0.4928	PASS
PEF	SeqId_8778_3	NOG	1	-	-	-	0.0137	1.05e-04	1.81e-19	TRUE	-	-

PEF	SeqId_8956_96	SCARF2	4	0.0448(-0.0092, 0.0988)	0.1037	0.8804	0.0564	8.73e-05	3.97e-85	TRUE	0.7303	PASS
PEF	SeqId_8960_3	ERAP2	16	0.0074(-0.0041, 0.0189)	0.2093	0.2160	0.2552	1.16e-04	0	TRUE	0.4563	PASS
PEF	SeqId_9076_25	PENK	12	-0.0175(-0.0326, -0.0024)	0.0231	0.9253	0.2390	8.41e-05	0	TRUE	0.8634	PASS
PEF	SeqId_9294_45	MFAP2	6	-0.0455(-0.0706, -0.0205)	0.0004	0.9397	0.1027	2.03e-04	2.62e-157	TRUE	0.7680	PASS

Effects of plasma proteins on lung functions are reported to be beta value, indicating the extent of the quantitative trait changes per one SD change in protein abundance. SNPN represents the number of instrumental variants of corresponding proteins. Egger.P is p-value for testing of causal effect from MR-Egger while Intercept.P is p-value for the testing of intercept from MR-egger suggesting of potential horizontal pleiotropy and Steiger.P is p-value in MR-Steiger analysis representing if instrumental variants account for a larger proportion of protein than outcomes. Q.P is the p-value in Q test for heterogeneity. R^2.exposure is the proportion of the protein variance explained by instrumental variants as well as R^2.outcome is the proportion of the outcome variance explained by instrumental variants. Effect Direction represents the consistency of directions of z-scores from PWAS and causal effect estimates in MR analysis of which TRUE means the consistent direction in two analysis. LOO is short for leave one out analysis of which PASS represents there are not instrument variables with strong influence while Excluded represents the protein-function pair is excluded due to the heterogeneity.

Table S6 Detail results of the colocalization analysis

Lung Function	Protein	Gene	SNPN	Protein.PP.H4	Lead SNP	SNP.PP.H4
FVC	SeqId_10396_6	MCL1	1989	2.65e-17	rs72700845	0.4081
FVC	SeqId_10833_64	HHIP	1751	2.29e-02	rs11727676	1.0000
FVC	SeqId_12675_14	DARS	1644	4.41e-02	rs2304371	0.6940
FEV1	SeqId_12831_21	TESC	2588	8.72e-01	rs2393124	0.2032
FVC	SeqId_13094_75	RSPO3	2027	1.11e-11	rs2154167	0.4237
FVC	SeqId_15584_9	CFHR2	1530	1.19e-01	rs4085749	1.0000
FVC	SeqId_16015_19	GPT	1051	6.48e-10	rs4244610	0.1331
FEV1	SeqId_15336_7	SELM	2495	9.63e-01	rs17822214	0.4323
FVC	SeqId_17682_1	CD46	2523	2.78e-01	rs2488252	0.2902
FVC	SeqId_17692_2	BTN3A3	2344	1.04e-02	rs9393711	0.9903
FEV1	SeqId_15441_6	GM2A	3164	9.81e-01	rs72794132	1.0000
FVC	SeqId_17761_2	NUDT5	3411	6.84e-01	rs7916879	0.3637
FEV1	SeqId_17224_12	OGN	2739	9.78e-01	rs10992291	0.4895
FVC	SeqId_18922_27	CD68	2486	3.89e-03	rs9901673	0.5176
FEV1	SeqId_17722_5	FKBP4	3030	1.0000	rs56196860	1.0000
FEV1	SeqId_19446_1	GMPR2	2688	9.91e-01	rs34354104	1.0000
FVC	SeqId_2644_11	PRKCA	3508	3.92e-01	rs17706845	0.1547
FEV1	SeqId_3221_54	SFRP1	3224	9.84e-01	rs147918758	0.5573
FVC	SeqId_3181_50	CTSS	2033	6.36e-22	rs41271951	1.0000
FVC	SeqId_3186_2	C2	4671	7.08e-07	rs9268149	0.8047
FVC	SeqId_3339_33	THBS2	3678	7.07e-07	rs74507247	0.6542
FEV1	SeqId_3435_53	FN1	2637	9.69e-01	rs1250259	0.5226
FVC	SeqId_3617_80	HGFAC	2426	2.18e-03	rs2498323	1.0000

FEV1	SeqId_4559_64	KYNU	2446	9.59e-01	rs112211045	0.2948
FVC	SeqId_4125_52	AGER	4364	7.16e-03	rs2070600	0.9789
FVC	SeqId_4158_54	PLAU	1375	3.85e-01	rs2633322	0.8744
FEV1	SeqId_6451_64	ASPN	2629	8.00e-01	rs2516568	0.4981
FVC	SeqId_5102_55	MICB	7791	7.08e-09	rs3094010	0.4439
FVC	SeqId_5676_54	ASIP	1455	4.27e-02	rs6059655	1.0000
FEV1	SeqId_8480_29	EFEMP1	3294	9.76e-01	rs3791679	0.8674
FVC	SeqId_6431_68	PCYOX1	2487	6.19e-01	rs2706762	1.0000
FEV1	SeqId_8956_96	SCARF2	2697	9.99e-01	rs2108746	0.3657
FVC	SeqId_6927_7	NDST1	2457	3.84e-01	rs11746198	0.2089
FVC	SeqId_8397_147	QSOX2	2386	4.86e-10	rs10858248	1.0000
FVC	SeqId_8427_118	RSPO3	2027	1.05e-11	rs1892172	0.4831
FEV1/FVC	SeqId_11196_31	COL6A3	3248	1.0000	rs1050785	1.0000
FVC	SeqId_9005_16	PLXNA1	3050	5.78e-01	rs9822602	0.3950
FVC	SeqId_9216_100	PLXNB2	3240	1.56e-02	rs28573806	0.5643
FVC	SeqId_9294_45	MFAP2	2144	5.18e-01	rs761422	0.9398
FVC	SeqId_9348_1	C1RL	2146	1.59e-03	rs11613834	0.9979
FEV1/FVC	SeqId_13133_73	LTBP4	2741	1.0000	rs34093919	1.0000
FVC	SeqId_9950_229	LAG3	2203	6.68e-02	rs3782735	0.5138
PEF	SeqId_10521_10	MXRA8	969	6.05e-01	rs1240713	0.1762
PEF	SeqId_10833_64	HHIP	1751	5.73e-18	rs13107186	0.1616
PEF	SeqId_10974_20	SPINK7	2625	6.81e-16	rs71580489	0.0767
PEF	SeqId_11387_3	ATF6B	4207	2.95e-26	rs6449	1.0000
PEF	SeqId_12396_19	HIBCH	2215	3.31e-08	rs291466	0.9998
FEV1/FVC	SeqId_13423_94	FAM213A	3645	9.83e-01	rs1538822	0.1687

PEF	SeqId_12563_2	TNFAIP8	2419	1.01e-14	rs1032859	0.4594
FEV1/FVC	SeqId_13488_3	ARFIP1	2476	9.76e-01	rs4619875	1.0000
PEF	SeqId_15314_49	CLIC4	2516	1.76e-05	rs4649017	0.8083
FEV1/FVC	SeqId_15441_6	GM2A	3164	7.70e-01	rs72794132	1.0000
PEF	SeqId_15472_16	LRP11	2776	7.44e-01	rs3805753	0.4208
FEV1/FVC	SeqId_16060_99	NID2	3649	8.68e-01	rs941622	0.7495
PEF	SeqId_17692_2	BTN3A3	2345	3.21e-04	rs9393711	0.9629
FEV1/FVC	SeqId_17692_2	BTN3A3	2344	8.59e-01	rs9393711	0.9670
PEF	SeqId_17761_2	NUDT5	3411	6.16e-01	rs2271804	0.9714
PEF	SeqId_17765_3	SNUPN	1528	2.06e-05	rs7170787	0.7721
PEF	SeqId_18235_16	PGP	1976	2.82e-02	rs116977380	1.0000
PEF	SeqId_2644_11	PRKCA	3508	3.05e-01	rs17706845	0.1512
PEF	SeqId_2730_58	MICA	8022	1.25e-41	rs2256174	0.5898
PEF	SeqId_2849_49	AIF1	6870	3.63e-08	rs137893789	0.3568
PEF	SeqId_2855_49	MAPK3	988	4.94e-01	rs9932466	0.8968
FEV1/FVC	SeqId_2855_49	MAPK3	988	9.99e-01	rs9932466	0.9349
FEV1/FVC	SeqId_3481_87	XPNPEP1	2155	9.33e-01	rs3862006	0.0888
FEV1/FVC	SeqId_4125_52	AGER	4364	1.0000	rs2070600	1.0000
FEV1/FVC	SeqId_4148_49	PAPPA	2342	9.93e-01	rs10983184	0.9990
FEV1/FVC	SeqId_4234_8	IL1RL1	2813	1.0000	rs13020553	0.5564
PEF	SeqId_4148_49	PAPPA	2342	4.89e-01	rs811689	0.1641
FEV1/FVC	SeqId_4328_2	BOC	3247	9.88e-01	rs3856718	0.3565
PEF	SeqId_5102_55	MICB	7791	8.34e-03	rs3094010	0.3608
FEV1/FVC	SeqId_4479_14	SERPING1	2136	9.96e-01	rs10896631	0.2859
PEF	SeqId_6713_4	LRP11	2776	3.72e-03	rs7763718	0.9796

FEV1/FVC	SeqId_6342_10	NPNT	2493	1.0000	rs34712979	1.0000
FEV1/FVC	SeqId_7905_30	HP	2196	9.73e-01	rs34042070	0.9994
PEF	SeqId_8960_3	ERAP2	3270	4.78e-01	rs2910686	0.5499
PEF	SeqId_9076_25	PENK	2865	3.64e-01	rs2089984	0.1754
PEF	SeqId_9294_45	MFAP2	2144	8.04e-02	rs9435731	0.6121
FEV1/FVC	SeqId_10816_150	PILRA	1483	2.27e-09	rs1859788	1.0000
FEV1/FVC	SeqId_10833_64	HHIP	1751	8.72e-18	rs13113591	0.3387
FEV1/FVC	SeqId_10924_258	CYB5D2	2860	6.56e-18	rs530093989	0.9609
FEV1/FVC	SeqId_10974_20	SPINK7	2625	2.69e-45	rs7733410	0.8400
FEV1/FVC	SeqId_8480_29	EFEMP1	3294	8.91e-01	rs3791679	0.5544
FEV1/FVC	SeqId_11387_3	ATF6B	4207	8.97e-37	rs2070600	1.0000
FEV1/FVC	SeqId_12524_18	SAT2	2466	7.03e-02	rs12942088	0.4704
FEV1/FVC	SeqId_8778_3	NOG	2111	9.95e-01	rs12951993	0.5639
FEV1/FVC	SeqId_8956_96	SCARF2	2697	9.99e-01	rs5763025	0.4207
FEV1/FVC	SeqId_8992_1	TMEM2	2461	9.98e-01	rs7046050	0.3075
FEV1/FVC	SeqId_14112_40	RELT	1672	9.06e-03	rs7107905	0.0845
FEV1/FVC	SeqId_14133_93	IL1R2	2762	1.32e-09	rs2310170	0.7330
FEV1/FVC	SeqId_15314_49	CLIC4	2516	7.71e-05	rs4649017	0.8038
FEV1/FVC	SeqId_9578_263	MANSC4	3086	8.56e-01	rs12368818	0.2874
FEV1/FVC	SeqId_15594_47	HTRA1	3116	4.07e-05	rs7074542	0.4235
FVC	SeqId_12831_21	TESC	2588	9.59e-01	rs2393124	0.1850
FEV1/FVC	SeqId_16583_8	NUDT2	2290	8.17e-02	rs10971956	0.9999
FEV1/FVC	SeqId_16781_2	ENGASE	3155	3.02e-02	rs56107536	1.0000
FVC	SeqId_17224_12	OGN	2739	7.93e-01	rs10992291	0.5860
FEV1/FVC	SeqId_17761_2	NUDT5	3411	4.02e-01	rs57062879	0.4590

FEV1/FVC	SeqId_18884_22	DNAJB4	2242	1.33e-01	rs7514180	0.6292
FEV1/FVC	SeqId_19207_119	ADK	1339	8.51e-03	rs12217834	0.1583
FEV1/FVC	SeqId_2617_56	ERBB3	1365	1.46e-01	rs2292238	0.3933
FEV1/FVC	SeqId_2743_5	SHH	3485	9.51e-10	rs872723	0.5116
FEV1/FVC	SeqId_2813_11	AGRP	1153	1.29e-04	rs9922624	0.1184
FEV1/FVC	SeqId_2849_49	AIF1	6870	9.59e-04	rs34562262	0.9998
FVC	SeqId_17722_5	FKBP4	3030	1.0000	rs56196860	1.0000
FEV1/FVC	SeqId_2994_71	IL1RL2	2731	5.07e-07	rs1960510	0.2937
FEV1/FVC	SeqId_3054_3	HP	2196	5.96e-03	rs77303550	1.0000
FEV1/FVC	SeqId_3296_92	CNTN2	2782	3.60e-03	rs3753847	1.0000
FEV1/FVC	SeqId_3331_8	RGMB	2622	5.92e-05	rs1840494	0.1406
FEV1/FVC	SeqId_3376_49	IL17RD	2596	3.01e-04	rs6776722	0.5937
FVC	SeqId_17764_108	RHOC	2184	9.89e-01	rs7415820	0.1708
FVC	SeqId_19446_1	GMPR2	2688	9.63e-01	rs34354104	1.0000
FVC	SeqId_19523_215	PARK7	2672	9.78e-01	rs17523802	0.9974
FVC	SeqId_3041_55	MRC2	1842	9.56e-01	rs146385050	1.0000
FEV1/FVC	SeqId_4240_31	PKM2	1338	6.95e-02	rs28379368	0.5014
FVC	SeqId_3376_49	IL17RD	2596	8.20e-01	rs6776722	0.5605
FVC	SeqId_3799_11	CA3	1363	9.97e-01	rs1543852	0.2840
FEV1/FVC	SeqId_4487_1	FGF7	2494	3.23e-12	rs17478785	0.4823
FEV1/FVC	SeqId_5581_28	FGL1	5268	3.49e-01	rs3739406	0.6612
FVC	SeqId_4220_39	FER	3157	9.97e-01	rs71592765	0.0764
FEV1/FVC	SeqId_6402_8	PILRA	1483	2.27e-09	rs1859788	1.0000
FEV1/FVC	SeqId_6451_64	ASPN	2629	7.19e-01	rs2516568	0.4983
FVC	SeqId_6388_21	CCDC126	2691	8.80e-01	rs35121828	0.6565

FEV1/FVC	SeqId_7922_5	ADM	3198	1.31e-01	rs10770085	0.1303
FEV1/FVC	SeqId_8269_327	ARSK	2279	1.13e-06	rs987068	0.4809
FEV1/FVC	SeqId_8296_117	KDELC2	1744	4.60e-01	rs141379009	0.9855
FEV1/FVC	SeqId_8409_3	RSPO2	2790	4.32e-01	rs6997695	0.0401
FVC	SeqId_6527_1	TRIL	3266	9.80e-01	rs740250	0.2714
FVC	SeqId_8480_29	EFEMP1	3294	9.99e-01	rs3791679	0.8789
FEV1/FVC	SeqId_8866_53	QPCTL	2714	1.61e-15	rs17850756	1.0000
FVC	SeqId_9769_48	DNER	2730	9.98e-01	rs35975053	0.6414
FEV1/FVC	SeqId_8974_172	COL15A1	2751	6.61e-01	rs7874948	0.4367
PEF	SeqId_12432_23	CACYBP	2586	9.44e-01	rs16847450	0.7117
FEV1/FVC	SeqId_9294_45	MFAP2	2144	5.09e-02	rs2284746	0.5410
PEF	SeqId_13133_73	LTBP4	2739	1.0000	rs34093919	0.9999
FEV1	SeqId_10833_64	HHIP	1751	6.41e-18	rs7663740	0.1808
FEV1	SeqId_10974_20	SPINK7	2625	1.41e-25	rs7713986	0.0468
FEV1	SeqId_11196_31	COL6A3	3248	1.69e-01	rs1050785	1.0000
FEV1	SeqId_12675_14	DARS	1644	9.15e-02	rs2304371	0.7336
PEF	SeqId_15441_6	GM2A	3164	9.51e-01	rs72794132	1.0000
FEV1	SeqId_13094_75	RSPO3	2027	7.14e-03	rs2154167	0.4726
FEV1	SeqId_14112_40	RELT	1672	6.17e-01	rs7123298	0.0819
PEF	SeqId_15526_33	GSS	1747	8.86e-01	rs6088642	0.1451
PEF	SeqId_17722_5	FKBP4	3031	9.98e-01	rs56196860	1.0000
FEV1	SeqId_15513_108	PRSS8	1245	4.23e-05	rs4889599	0.0815
FEV1	SeqId_16015_19	GPT	1051	2.26e-08	rs4244610	0.2526
PEF	SeqId_3196_6	HAPLN1	2156	9.76e-01	rs7735476	0.1610
FEV1	SeqId_17682_1	CD46	2523	3.93e-02	rs2488252	0.2823

FEV1	SeqId_17692_2	BTN3A3	2344	4.57e-02	rs9393711	0.9872
PEF	SeqId_3280_49	ACAN	2696	1.0000	rs34949187	1.0000
FEV1	SeqId_17761_2	NUDT5	3411	4.99e-01	rs2271804	0.6301
FEV1	SeqId_18922_27	CD68	2486	1.62e-02	rs9901673	0.5229
PEF	SeqId_3435_53	FN1	2637	9.95e-01	rs1250258	0.5075
FEV1	SeqId_3181_50	CTSS	2033	8.62e-13	rs41271951	1.0000
FEV1	SeqId_3186_2	C2	4671	1.04e-04	rs1150752	0.3834
PEF	SeqId_3481_87	XPNPEP1	2155	9.24e-01	rs3862006	0.1186
FEV1	SeqId_3331_8	RGMB	2622	2.56e-03	rs2368553	0.1133
FEV1	SeqId_3339_33	THBS2	3678	3.22e-04	rs74507247	0.6530
FEV1	SeqId_3376_49	IL17RD	2596	3.98e-04	rs6776722	0.5079
PEF	SeqId_4125_52	AGER	4364	1.0000	rs2070600	0.7811
FEV1	SeqId_4125_52	AGER	4364	3.47e-16	rs2070600	0.9508
FEV1	SeqId_4148_49	PAPPA	2342	1.60e-01	rs803912	0.1111
FEV1	SeqId_4158_54	PLAU	1375	5.49e-04	rs2633322	0.8671
FEV1	SeqId_4407_10	MST1	1353	2.09e-01	rs3197999	1.0000
PEF	SeqId_5091_28	LILRB2	2027	9.98e-01	rs383925	1.0000
FEV1	SeqId_4961_17	ANXA2	2553	5.01e-02	rs12904906	0.1400
FEV1	SeqId_5102_55	MICB	7791	4.60e-04	rs3094010	0.3681
FEV1	SeqId_6431_68	PCYOX1	2487	5.22e-01	rs2706762	1.0000
PEF	SeqId_6342_10	NPNT	2493	1.0000	rs34712979	1.0000
FEV1	SeqId_6462_12	TIMP4	2824	2.99e-01	rs184262	0.4749
FEV1	SeqId_6897_38	B3GAT3	2177	5.63e-05	rs3923805	0.3816
FEV1	SeqId_6927_7	NDST1	2457	5.68e-01	rs253294	0.2257
FEV1	SeqId_8427_118	RSPO3	2027	6.14e-03	rs1892172	0.4680

PEF	SeqId_8778_3	NOG	2111	9.59e-01	rs1029723	0.3762
PEF	SeqId_8956_96	SCARF2	2697	9.95e-01	rs5763025	0.3457
FEV1	SeqId_9216_100	PLXNB2	3240	2.08e-03	rs28573806	0.5278
FEV1	SeqId_9950_229	LAG3	2203	3.48e-01	rs3782735	0.8665

Significant colocalization result is considered significant at PP.H4 > 0.75. Protein.PP.H4 is the posterior of the cis-region of protein-coding genes. Lead SNP is the SNP residing in the cis-region with the highest posterior and SNP.PP.H4 is the corresponding posterior. SNP: single nucleotide polymorphism

Table S7 Detail significant results in replication MR analysis

Lung Function	Protein	Gene	SNPN	F-statistics	PVE	Effect (95% CI)	P value	FDR
PEF	SeqId_12432_23	CACYBP	1	220.7267	0.0062	-0.0798 (-0.1173, -0.0424)	2.92e-05	6.78e-05
PEF	SeqId_13133_73	LTBP4	3	36.2225	0.0031	-0.1113(-0.1725, -0.0501)	3.67e-04	6.65e-04
PEF	SeqId_15526_33	GSS	3	162.9682	0.0136	0.1026(0.0740, 0.1311)	1.84e-12	3.56e-11
PEF	SeqId_3196_6	HAPLN1	5	81.6376	0.0114	-0.0642(-0.1131, -0.0152)	1.02e-02	1.29e-02
PEF	SeqId_3280_49	ACAN	4	76.7412	0.0086	-0.1333(-0.2146, -0.0519)	1.32e-03	1.96e-03
PEF	SeqId_3481_87	XPNPEP1	1	55.8870	0.0016	-0.1806(-0.2600, -0.1012)	8.31e-06	2.41e-05
PEF	SeqId_5091_28	LILRB2	35	215.8166	0.1761	0.0144(0.0076, 0.0211)	3.16e-05	7.05e-05
PEF	SeqId_6342_10	NPNT	5	124.8951	0.0174	0.1622(0.0538, 0.2706)	3.37e-03	4.54e-03
PEF	SeqId_8956_96	SCARF2	4	81.3313	0.0091	0.0917(0.0515, 0.1318)	7.70e-06	2.35e-05
FEV1/FVC	SeqId_11196_31	COL6A3	2	45.0797	0.0025	-0.1877(-0.2510, -0.1244)	6.08e-09	5.04e-08
FEV1/FVC	SeqId_13133_73	LTBP4	3	36.2225	0.0031	-0.2704(-0.3282, -0.2126)	5.02e-20	1.46e-18
FEV1/FVC	SeqId_13423_94	FAM213A	2	81.1305	0.0046	-0.1695(-0.2173, -0.1217)	3.62e-12	5.26e-11
FEV1/FVC	SeqId_15441_6	GM2A	14	138.77901	0.0521	-0.0542(-0.0707, -0.0377)	1.33e-10	1.29e-09
FEV1/FVC	SeqId_16060_99	NID2	29	177.7640	0.1273	0.0149(0.0057, 0.0241)	1.50e-03	2.18e-03
FEV1/FVC	SeqId_17692_2	BTN3A3	17	277.4731	0.1177	0.0161(0.0097, 0.0225)	8.87e-07	3.68e-06
FEV1/FVC	SeqId_2855_49	MAPK3	8	145.3673	0.0318	0.0514(0.0340, 0.0689)	8.14e-09	5.25e-08
FEV1/FVC	SeqId_3481_87	XPNPEP1	1	55.8870	0.0016	-0.1829(-0.2577, -0.1080)	1.69e-06	6.52e-06
FEV1/FVC	SeqId_4234_8	IL1RL1	20	423.1213	0.1932	0.0120(0.0039, 0.0200)	3.57e-03	4.71e-03
FEV1/FVC	SeqId_4328_2	BOC	10	104.1384	0.0286	0.0366(0.0161, 0.0572)	4.71e-04	8.03e-04
FEV1/FVC	SeqId_4479_14	SERPING1	22	203.0207	0.1122	0.0221(0.0135, 0.0307)	5.33e-07	2.58e-06
FEV1/FVC	SeqId_6342_10	NPNT	5	124.8951	0.0174	0.2679(0.1077, 0.4281)	1.05e-03	1.69e-03
FEV1/FVC	SeqId_8480_29	EFEMP1	6	87.2700	0.0146	0.1058(0.0524, 0.1591)	1.02e-04	2.05e-04
FEV1/FVC	SeqId_8778_3	NOG	12	168.4744	0.0541	-0.0156(-0.0299, -0.0013)	3.19e-02	3.56e-02

FEV1/FVC	SeqId_8956_96	SCARF2	4	81.3313	0.0091	0.1580(0.1249, 0.1911)	8.11e-21	4.70e-19
FEV1/FVC	SeqId_8992_1	TMEM2	3	140.4913	0.0118	-0.0816(-0.1102, -0.053)	2.15e-08	1.25e-07
FEV1/FVC	SeqId_9578_263	MANSC4	21	189.1386	0.1010	-0.0268(-0.0391, -0.0144)	2.23e-05	5.88e-05
FEV1	SeqId_15336_7	SELM	2	34.3415	0.0019	-0.1707(-0.2366, -0.1048)	3.86e-07	2.04e-06
FEV1	SeqId_17224_12	OGN	17	136.4241	0.0616	0.0350(0.0188, 0.0512)	2.31e-05	5.58e-05
FEV1	SeqId_17722_5	FKBP4	2	71.6164	0.0040	0.0706(0.0072, 0.1341)	2.92e-02	3.32e-02
FEV1	SeqId_19446_1	GMPR2	8	143.3959	0.0314	-0.0474(-0.0661, -0.0288)	6.25e-07	2.79e-06
FEV1	SeqId_3221_54	SFRP1	10	115.8189	0.0317	-0.0404(-0.0726, -0.0083)	1.37e-02	1.66e-02
FEV1	SeqId_4559_64	KYNU	11	101.3626	0.0306	-0.0226(-0.0363, -0.0090)	1.18e-03	1.85e-03
FEV1	SeqId_8480_29	EFEMP1	6	87.2700	0.0146	-0.0608(-0.1174, -0.0043)	3.49e-02	3.82e-02
FEV1	SeqId_8956_96	SCARF2	4	81.3313	0.0091	0.1111(0.0790, 0.1432)	1.19e-11	1.38e-10
FVC	SeqId_17224_12	OGN	17	136.4241	0.0616	0.0293(0.0137, 0.0449)	2.34e-04	4.38e-04
FVC	SeqId_17722_5	FKBP4	2	71.6164	0.0040	0.1051(0.0415, 0.1687)	1.19e-03	1.83e-03
FVC	SeqId_17764_108	RHOC	3	70.8553	0.0060	0.0914(0.0519, 0.1309)	5.72e-06	1.95e-05
FVC	SeqId_19446_1	GMPR2	8	143.3959	0.0314	-0.0439(-0.0629, -0.0250)	5.55e-06	2.01e-05
FVC	SeqId_19523_215	PARK7	3	89.03166	0.0075	-0.0806(-0.1167, -0.0445)	1.20e-05	3.32e-05
FVC	SeqId_3041_55	MRC2	14	112.5189	0.0427	0.0273(0.0049, 0.0498)	1.71e-02	2.02e-02
FVC	SeqId_3376_49	IL17RD	14	186.2120	0.0687	0.0282(0.0160, 0.0404)	5.84e-06	1.88e-05
FVC	SeqId_4220_39	FER	2	52.1273	0.0029	0.0955(0.0399, 0.1510)	7.50e-04	1.24e-03
FVC	SeqId_6388_21	CCDC126	8	201.2892	0.0436	-0.0294(-0.0523, -0.0065)	1.19e-02	1.46e-02
FVC	SeqId_6527_1	TRIL	2	44.6445	0.0025	0.1189(0.0639, 0.1739)	2.29e-05	5.77e-05
FVC	SeqId_8480_29	EFEMP1	6	87.2700	0.0146	-0.1207(-0.1976, -0.0438)	2.10e-03	2.97e-03
FVC	SeqId_9769_48	DNER	12	115.1441	0.0376	0.0443(0.0138, 0.0749)	4.47e-03	5.77e-03

All explanations are the same as that for Table S3

Table S8 Detail results of sensitive analysis for replication MR

Lung Function	Protein	Gene	SNPN	Effect (95% CI)	Egger.P	Intercept.P	R^2.exposure	R^2.outcome	Steiger.P	Effect Direction	Q.P	LOO
PEF	SeqId_12432_23	CACYBP	1	-	-	-	0.0062	4.98e-05	6.43e-38	TRUE	-	-
PEF	SeqId_13133_73	LTBP4	3	-0.3185(-0.6907, 0.0536)	0.0934	0.2100	0.0031	1.03e-04	5.19e-16	TRUE	1.04e-05	Excluded
PEF	SeqId_15526_33	GSS	3	-0.0016(-0.3642, 0.3610)	0.9931	0.5723	0.0136	1.45e-04	2.74e-79	TRUE	0.7217	PASS
PEF	SeqId_3196_6	HAPLN1	5	-0.0075(-0.1437, 0.1286)	0.9136	0.3801	0.0114	6.39e-05	1.09e-70	TRUE	0.0847	PASS
PEF	SeqId_3280_49	ACAN	4	-0.1235(-0.3728, 0.1258)	0.3317	0.9331	0.0086	2.04e-04	3.59e-45	TRUE	0.0010	PASS
PEF	SeqId_3481_87	XPNPEP1	1	-	-	-	0.0016	5.65e-05	7.85e-09	TRUE	-	-
PEF	SeqId_5091_28	LILRB2	35	0.0152(0.0050, 0.0253)	0.0034	0.8372	0.1761	1.26e-04	0	TRUE	0.8426	PASS
PEF	SeqId_6342_10	NPNT	5	0.2563(0.0514, 0.4611)	0.0142	0.2913	0.0174	7.01e-04	2.09e-80	TRUE	1.52e-15	Excluded
PEF	SeqId_8956_96	SCARF2	4	0.0824(-0.1213, 0.2861)	0.4279	0.9269	0.0091	8.72e-05	4.74e-54	TRUE	0.2724	PASS
FEV1/FVC	SeqId_11196_31	COL6A3	2	-	-	-	0.0025	1.32e-04	2.18e-12	TRUE	-	-
FEV1/FVC	SeqId_13133_73	LTBP4	3	-0.6260(-1.5330, 0.2809)	0.1761	0.3770	0.0031	4.72e-04	1.27e-09	TRUE	7.59e-24	Excluded
FEV1/FVC	SeqId_13423_94	FAM213A	2	-	-	-	0.0046	1.29e-04	3.45e-24	TRUE	-	-
FEV1/FVC	SeqId_15441_6	GM2A	14	-0.0653(-0.0951, -0.0354)	1.81e-05	0.3808	0.0521	2.13e-04	0	TRUE	0.0945	PASS
FEV1/FVC	SeqId_16060_99	NID2	29	0.0319(0.0122, 0.0516)	0.0015	0.0584	0.1273	1.11e-04	0	TRUE	0.2438	PASS
FEV1/FVC	SeqId_17692_2	BTN3A3	17	0.0172(0.0047, 0.0297)	0.0071	0.8368	0.1177	1.13e-04	0	TRUE	0.3100	PASS
FEV1/FVC	SeqId_2855_49	MAPK3	8	0.0779(0.0157, 0.1400)	0.0141	0.3853	0.0318	9.32e-05	7.61e-208	TRUE	0.7006	PASS
FEV1/FVC	SeqId_3481_87	XPNPEP1	1	-	-	-	0.0016	5.63e-05	6.28e-09	TRUE	-	-
FEV1/FVC	SeqId_4234_8	IL1RL1	20	0.0212(0.0010, 0.0414)	0.0401	0.3302	0.1932	1.12e-04	0	TRUE	0.0424	PASS
FEV1/FVC	SeqId_4328_2	BOC	10	0.0574(0.0235, 0.0913)	0.0009	0.1433	0.0286	7.33e-05	8.23e-188	TRUE	0.2043	PASS
FEV1/FVC	SeqId_4479_14	SERPING1	22	0.0073(-0.0109, 0.0255)	0.4302	0.0709	0.1122	1.07e-04	0	TRUE	0.7101	PASS
FEV1/FVC	SeqId_6342_10	NPNT	5	0.4008(0.0973, 0.7044)	0.0096	0.3125	0.0174	1.69e-03	5.56e-61	TRUE	2.27e-37	PASS
FEV1/FVC	SeqId_8480_29	EFEMP1	6	0.1790(0.0278, 0.3302)	0.0204	0.3107	0.0146	1.98e-04	2.43e-83	TRUE	0.0015	PASS
FEV1/FVC	SeqId_8778_3	NOG	12	-0.0170(-0.0341, 0.0001)	0.0507	0.7733	0.0541	2.51e-05	0	TRUE	0.9105	Excluded

FEV1/FVC	SeqId_8956_96	SCARF2	4	0.2243(0.0862, 0.3623)	0.0015	0.3326	0.0091	2.22e-04	4.20e-48	TRUE	0.5389	PASS
FEV1/FVC	SeqId_8992_1	TMEM2	3	-0.1041(-0.1634, -0.0449)	0.0006	0.3949	0.0118	7.93e-05	9.25e-73	TRUE	0.6166	PASS
FEV1/FVC	SeqId_9578_263	MANSC4	21	-0.0433(-0.0638, -0.0228)	3.61e-05	0.0558	0.1010	1.58e-04	0	TRUE	0.0263	PASS
FEV1	SeqId_15336_7	SELM	2	-	-	-	0.0019	6.48e-05	8.41e-11	TRUE	-	-
FEV1	SeqId_17224_12	OGN	17	0.0256(-0.0302, 0.0815)	0.3681	0.7303	0.0616	1.24e-04	0	TRUE	0.1126	PASS
FEV1	SeqId_17722_5	FKBP4	2	-	-	-	0.0040	1.20e-05	2.31e-27	TRUE	-	-
FEV1	SeqId_19446_1	GMPR2	8	-0.0510(-0.0752, -0.0268)	3.64e-05	0.6503	0.0314	6.51e-05	7.16e-209	TRUE	0.9778	PASS
FEV1	SeqId_3221_54	SFRP1	10	-0.0493(-0.1684, 0.0697)	0.4168	0.8787	0.0317	1.38e-04	6.93e-202	TRUE	0.0001	PASS
FEV1	SeqId_4559_64	KYNU	11	-0.0313(-0.0521, -0.0104)	0.0033	0.2837	0.0306	6.37e-05	7.39e-203	TRUE	0.2620	PASS
FEV1	SeqId_8480_29	EFEMP1	6	-0.0462(-0.2268, 0.1344)	0.6162	0.8652	0.0146	1.07e-04	4.80e-89	TRUE	0.0004	PASS
FEV1	SeqId_8956_96	SCARF2	4	0.1425(-0.0118, 0.2968)	0.0703	0.6818	0.0091	1.18e-04	7.28e-53	TRUE	0.4168	PASS
FVC	SeqId_17224_12	OGN	17	0.0344(-0.0195, 0.0882)	0.2107	0.8459	0.0616	9.84e-05	0	TRUE	0.1678	PASS
FVC	SeqId_17722_5	FKBP4	2	-	-	-	0.0040	2.64e-05	5.97e-26	TRUE	-	-
FVC	SeqId_17764_108	RHOC	3	-0.0196(-0.9318, 0.8926)	0.9665	0.8094	0.0060	8.80e-05	1.38e-34	TRUE	0.0015	PASS
FVC	SeqId_19446_1	GMPR2	8	-0.0494(-0.0740, -0.0247)	8.54e-05	0.4978	0.0314	5.39e-05	1.27e-210	TRUE	0.9963	PASS
FVC	SeqId_19523_215	PARK7	3	0.0174(-0.1418, 0.1765)	0.8307	0.1983	0.0075	6.43e-05	8.68e-46	TRUE	0.0451	PASS
FVC	SeqId_3041_55	MRC2	14	0.0267(-0.0248, 0.0782)	0.3088	0.9804	0.0427	1.01e-04	1.07e-282	TRUE	0.0100	PASS
FVC	SeqId_3376_49	IL17RD	14	0.0322(0.0070, 0.0574)	0.0122	0.7221	0.0687	7.86e-05	0	TRUE	0.6506	PASS
FVC	SeqId_4220_39	FER	2	-	-	-	0.0029	2.89e-05	1.25e-18	TRUE	-	-
FVC	SeqId_6388_21	CCDC126	8	-0.0573(-0.1002, -0.0143)	0.0089	0.1432	0.0436	1.08e-04	9.13e-289	TRUE	0.0178	PASS
FVC	SeqId_6527_1	TRIL	2	-	-	-	0.0025	5.01e-05	7.55e-15	TRUE	-	-
FVC	SeqId_8480_29	EFEMP1	6	-0.1446(-0.3905, 0.1013)	0.2492	0.8390	0.0146	2.97e-04	1.40e-78	TRUE	7.16e-08	PASS
FVC	SeqId_9769_48	DNER	12	0.0410(-0.0290, 0.1111)	0.2508	0.9173	0.0376	1.82e-04	3.10e-238	TRUE	1.89e-05	PASS

All explanations are the same as that for Table S4

Table S9 Detail results of MR analysis for lung diseases

Gene	Protein	Outcome	NSNP	F-statistics	PVE	Effect (95% CI)	P value	FDR
NPNT	SeqId_6342_10	COPD	3	0.0642	164.9483	(-0.5422, -0.3683)	1.05E-24	7.16E-23
MAPK3	SeqId_2855_49	Asthma	12	0.0565	35.9631	(-0.0063, -0.0025)	6.20E-06	2.11E-04
BTN3A3	SeqId_17692_2	Asthma	6	0.2113	321.6903	(-0.0049, -0.0019)	1.18E-05	2.68E-04
FAM213A	SeqId_13423_94	Asthma	1	0.0043	30.8668	(0.0108, 0.0382)	4.42E-04	6.01E-03
KYNU	SeqId_4559_64	Asthma	9	0.0353	29.3088	(0.0017, 0.0057)	3.77E-04	6.01E-03
EFEMP1	SeqId_8480_29	COPD	4	0.1009	202.2568	(-0.22, -0.0566)	9.06E-04	1.03E-02
GM2A	SeqId_15441_6	COPD	6	0.2760	457.8061	(0.0336, 0.1459)	1.73E-03	1.68E-02
CACYBP	SeqId_12432_23	Asthma	2	0.1130	459.0801	(0.0013, 0.0068)	3.67E-03	3.12E-02
FKBP4	SeqId_17722_5	COPD	3	0.0762	198.3066	(-0.1832, -0.0269)	8.42E-03	5.82E-02
GM2A	SeqId_15441_6	Asthma	8	0.2916	370.6939	(6e-04, 0.0039)	9.41E-03	5.82E-02
FER	SeqId_4220_39	COPD	2	0.0065	23.4702	(0.0928, 0.6584)	9.24E-03	5.82E-02
GMPR2	SeqId_19446_1	COPD	3	0.0890	234.7285	(0.0228, 0.1762)	1.10E-02	6.21E-02
OGN	SeqId_17224_12	COPD	6	0.1908	283.1512	(-0.1187, -0.0102)	1.99E-02	9.13E-02
SERPING1	SeqId_4479_14	Asthma	13	0.0949	58.0334	(-0.0032, -3e-04)	1.86E-02	9.13E-02
HAPLN1	SeqId_3196_6	Asthma	2	0.0152	55.7960	(0.0014, 0.0162)	2.01E-02	9.13E-02
IL1RL1	SeqId_4234_8	Asthma	10	0.1058	85.1931	(-0.0102, -6e-04)	2.76E-02	1.10E-01
ACAN	SeqId_3280_49	COPD	1	0.0141	103.4266	(0.0239, 0.4006)	2.72E-02	1.10E-01
SCARF2	SeqId_8956_96	COPD	3	0.0515	130.5000	(-0.2055, -0.0061)	3.75E-02	1.38E-01
FAM213A	SeqId_13423_94	COPD	1	0.0043	30.8668	(0.0157, 0.7067)	4.05E-02	1.38E-01
GSS	SeqId_15526_33	Asthma	4	0.1092	220.8335	(1e-04, 0.0057)	3.97E-02	1.38E-01
BOC	SeqId_4328_2	COPD	3	0.0477	120.3274	(-0.2174, -0.0037)	4.26E-02	1.38E-01
PARK7	SeqId_19523_215	Asthma	10	0.0416	31.2284	(-0.0065, 0)	4.67E-02	1.44E-01

SFRP1	SeqId_3221_54	Asthma	5	0.0433	65.1600	(-2e-04, 0.0088)	6.33E-02	1.87E-01
MAPK3	SeqId_2855_49	COPD	11	0.0455	31.2161	(-0.1288, 0.0064)	7.59E-02	1.98E-01
GSS	SeqId_15526_33	COPD	3	0.1062	285.4696	(-0.1369, 0.0064)	7.41E-02	1.98E-01
HAPLN1	SeqId_3196_6	COPD	1	0.0112	81.7069	(-0.0176, 0.4224)	7.14E-02	1.98E-01
FKBP4	SeqId_17722_5	Asthma	3	0.0762	198.3066	(-0.006, 4e-04)	9.06E-02	2.20E-01
LILRB2	SeqId_5091_28	Asthma	8	0.0648	62.4270	(-0.0046, 3e-04)	9.01E-02	2.20E-01
BOC	SeqId_4328_2	Asthma	4	0.0509	96.7220	(-0.0073, 8e-04)	1.19E-01	2.80E-01
COL6A3	SeqId_11196_31	COPD	5	0.0381	57.0257	(-0.0344, 0.2579)	1.34E-01	2.87E-01
NPNT	SeqId_6342_10	Asthma	5	0.0716	111.1515	(-0.0152, 0.002)	1.35E-01	2.87E-01
RHOC	SeqId_17764_108	COPD	2	0.0278	102.9231	(-0.032, 0.2411)	1.34E-01	2.87E-01
EFEMP1	SeqId_8480_29	Asthma	4	0.1009	202.2568	(-0.0055, 0.0012)	2.03E-01	3.71E-01
IL1RL1	SeqId_4234_8	COPD	8	0.0985	98.4327	(-0.0801, 0.0167)	1.99E-01	3.71E-01
MANSC4	SeqId_9578_263	COPD	10	0.0982	78.4017	(-0.0161, 0.0718)	2.14E-01	3.71E-01
NID2	SeqId_16060_99	Asthma	9	0.1797	175.3256	(-0.0036, 8e-04)	2.05E-01	3.71E-01
SERPING1	SeqId_4479_14	COPD	9	0.0780	67.6708	(-0.1445, 0.029)	1.92E-01	3.71E-01
DNER	SeqId_9769_48	Asthma	8	0.0731	71.0413	(-0.0013, 0.0055)	2.24E-01	3.71E-01
MRC2	SeqId_3041_55	Asthma	9	0.1085	97.4304	(-0.001, 0.0045)	2.10E-01	3.71E-01
CACYBP	SeqId_12432_23	COPD	2	0.1130	459.0801	(-0.0263, 0.1144)	2.20E-01	3.71E-01
LILRB2	SeqId_5091_28	COPD	7	0.0517	56.1059	(-0.0797, 0.015)	1.81E-01	3.71E-01
IL17RD	SeqId_3376_49	COPD	5	0.0370	55.4456	(-0.1492, 0.0406)	2.62E-01	4.24E-01
ACAN	SeqId_3280_49	Asthma	2	0.0257	94.9215	(-0.0026, 0.0086)	2.91E-01	4.60E-01
OGN	SeqId_17224_12	Asthma	6	0.1908	283.1512	(-0.0033, 0.0011)	3.17E-01	4.60E-01
SELM	SeqId_15336_7	Asthma	1	0.0076	55.1246	(-0.0163, 0.0053)	3.18E-01	4.60E-01
NID2	SeqId_16060_99	COPD	8	0.1766	193.2016	(-0.0849, 0.0264)	3.03E-01	4.60E-01
TMEM2	SeqId_8992_1	COPD	1	0.0240	177.6408	(-0.0713, 0.2245)	3.10E-01	4.60E-01

SELM	SeqId_15336_7	COPD	1	0.0076	55.1246	(-0.1329, 0.3975)	3.28E-01	4.65E-01
RHOC	SeqId_17764_108	Asthma	2	0.0278	102.9231	(-0.0028, 0.008)	3.45E-01	4.79E-01
SCARF2	SeqId_8956_96	Asthma	4	0.0564	107.6079	(-0.008, 0.003)	3.77E-01	5.13E-01
BTN3A3	SeqId_17692_2	COPD	4	0.0664	128.2252	(-0.1467, 0.0647)	4.47E-01	5.85E-01
PARK7	SeqId_19523_215	COPD	7	0.0288	30.4705	(-0.0307, 0.0695)	4.47E-01	5.85E-01
FER	SeqId_4220_39	Asthma	3	0.0281	69.5799	(-0.0035, 0.0075)	4.68E-01	6.00E-01
TRIL	SeqId_6527_1	COPD	2	0.0234	86.4571	(-0.198, 0.097)	5.02E-01	6.32E-01
XPNPEP1	SeqId_3481_87	COPD	1	0.0388	290.9597	(-0.0791, 0.1567)	5.19E-01	6.41E-01
MANSC4	SeqId_9578_263	Asthma	11	0.1017	74.1433	(-0.0022, 0.0012)	5.53E-01	6.71E-01
XPNPEP1	SeqId_3481_87	Asthma	1	0.0388	290.9597	(-0.0056, 0.0035)	6.52E-01	7.78E-01
IL17RD	SeqId_3376_49	Asthma	5	0.0370	55.4456	(-0.0038, 0.0025)	6.86E-01	8.04E-01
TMEM2	SeqId_8992_1	Asthma	1	0.0240	177.6408	(-0.0049, 0.0068)	7.50E-01	8.42E-01
CCDC126	SeqId_6388_21	Asthma	5	0.1174	191.7302	(-0.0023, 0.0032)	7.55E-01	8.42E-01
MRC2	SeqId_3041_55	COPD	8	0.1057	106.3855	(-0.0598, 0.0827)	7.54E-01	8.42E-01
KYNU	SeqId_4559_64	COPD	7	0.0286	30.2783	(-0.1181, 0.1578)	7.78E-01	8.53E-01
TRIL	SeqId_6527_1	Asthma	2	0.0234	86.4571	(-0.0051, 0.0065)	8.13E-01	8.77E-01
DNER	SeqId_9769_48	COPD	4	0.0190	34.9421	(-0.2151, 0.2482)	8.89E-01	9.44E-01
COL6A3	SeqId_11196_31	Asthma	5	0.0381	57.0257	(-0.0091, 0.008)	9.08E-01	9.47E-01
CCDC126	SeqId_6388_21	COPD	4	0.1115	226.1343	(-0.0975, 0.0879)	9.19E-01	9.47E-01
GMPR2	SeqId_19446_1	Asthma	4	0.0933	185.3720	(-0.0031, 0.0034)	9.36E-01	9.50E-01
SFRP1	SeqId_3221_54	COPD	3	0.0187	45.6812	(-0.1707, 0.1645)	9.71E-01	9.71E-01

Proteins are detected at the at an FDR threshold of 0.05. SNPN represents the number of instrumental variants of corresponding proteins. PVE is the proportion of the protein variance explained by instrumental variants.

Table S10 Detail results of sensitive analysis for lung diseases

Gene	Protein	outcome	SNPN	Effect (95% CI)	Egger.P	Intercept.P	R2.exposure	R2.outcome	Steiger.P	Effect Direction	Q.P
ACAN	SeqId_3280_49	Asthma	2	NA	NA	NA	0.0257	4.43E-06	3.61E-41	TRUE	NA
ACAN	SeqId_3280_49	COPD	1	NA	NA	NA	0.0141	2.43E-05	1.22E-21	TRUE	NA
BOC	SeqId_4328_2	COPD	3	(-2.9095, 3.8572)	0.7837	0.7348	0.0477	2.15E-05	1.86E-73	TRUE	9.07E-01
BOC	SeqId_4328_2	Asthma	4	(-0.019, 0.0129)	0.7057	0.9845	0.0509	6.81E-06	1.32E-81	TRUE	8.81E-01
BTN3A3	SeqId_17692_2	COPD	4	(-0.3381, 0.3008)	0.9090	0.8809	0.0664	2.43E-05	2.38E-103	TRUE	2.52E-01
BTN3A3	SeqId_17692_2	Asthma	6	(-0.0062, -2e-04)	0.0385	0.8939	0.2113	7.18E-05	0.00E+00	TRUE	2.52E-01
CACYBP	SeqId_12432_23	Asthma	2	NA	NA	NA	0.1130	2.24E-05	8.59E-186	TRUE	NA
CACYBP	SeqId_12432_23	COPD	2	NA	NA	NA	0.1130	1.37E-05	3.34E-183	TRUE	NA
CCDC126	SeqId_6388_21	Asthma	5	(-0.001, 0.009)	0.1149	0.0969	0.1174	1.05E-05	2.16E-195	TRUE	3.61E-01
CCDC126	SeqId_6388_21	COPD	4	(-0.1794, 0.2273)	0.8174	0.7412	0.1115	2.72E-05	4.63E-179	TRUE	1.42E-01
COL6A3	SeqId_11196_31	Asthma	5	(-0.0033, 0.0325)	0.1110	0.0742	0.0381	2.75E-05	4.18E-59	TRUE	1.03E-02
COL6A3	SeqId_11196_31	COPD	5	(-0.0922, 0.6696)	0.1374	0.3238	0.0381	4.65E-05	4.81E-57	TRUE	2.00E-01
DNER	SeqId_9769_48	COPD	4	(-0.1813, 1.7025)	0.1135	0.1140	0.0190	2.95E-05	9.30E-29	TRUE	1.17E-01
DNER	SeqId_9769_48	Asthma	8	(-0.0095, 0.0097)	0.9785	0.6680	0.0731	7.55E-06	1.91E-118	TRUE	9.29E-01
EFEMP1	SeqId_8480_29	COPD	4	(-0.5352, 0.0429)	0.0951	0.4426	0.1009	8.76E-05	1.02E-156	TRUE	2.88E-01
EFEMP1	SeqId_8480_29	Asthma	4	(-0.0158, 0.0107)	0.7041	0.9512	0.1009	1.37E-05	1.63E-165	TRUE	2.57E-01
FAM213A	SeqId_13423_94	Asthma	1	NA	NA	NA	0.0043	2.43E-05	3.49E-07	TRUE	NA
FAM213A	SeqId_13423_94	COPD	1	NA	NA	NA	0.0043	2.09E-05	3.92E-07	TRUE	NA
FER	SeqId_4220_39	Asthma	3	(-0.0291, 0.0185)	0.6646	0.5035	0.0281	1.26E-05	2.27E-44	TRUE	7.17E-02
FER	SeqId_4220_39	COPD	2	NA	NA	NA	0.0065	3.38E-05	4.40E-10	TRUE	NA
FKBP4	SeqId_17722_5	COPD	3	(-0.2863, 0.1171)	0.4113	0.7705	0.0762	5.38E-05	2.04E-117	TRUE	1.45E-01
FKBP4	SeqId_17722_5	Asthma	3	(-0.0081, 0.0029)	0.3516	0.9164	0.0762	8.78E-06	1.55E-123	TRUE	4.56E-01
GM2A	SeqId_15441_6	COPD	6	(-0.0582, 0.1713)	0.3338	0.5076	0.2760	1.22E-04	0.00E+00	TRUE	1.43E-01

GM2A	SeqId_15441_6	Asthma	8	(-3e-04, 0.0057)	0.0819	0.7432	0.2916	1.83E-05	0.00E+00	TRUE	9.70E-01
GMPR2	SeqId_19446_1	Asthma	4	(-0.0067, 0.0022)	0.3118	0.1556	0.0933	8.60E-06	6.82E-153	TRUE	2.88E-01
GMPR2	SeqId_19446_1	COPD	3	(-0.2633, 0.4247)	0.6457	0.8928	0.0890	6.98E-05	1.28E-137	TRUE	2.32E-02
GSS	SeqId_15526_33	COPD	3	(-0.159, 0.1816)	0.8964	0.3312	0.1062	2.55E-05	5.37E-170	TRUE	3.81E-01
GSS	SeqId_15526_33	Asthma	4	(-0.0046, 0.0088)	0.5325	0.7936	0.1092	1.07E-05	1.40E-180	TRUE	6.82E-01
HAPLN1	SeqId_3196_6	Asthma	2	NA	NA	NA	0.0152	1.27E-05	3.06E-24	TRUE	NA
HAPLN1	SeqId_3196_6	COPD	1	NA	NA	NA	0.0112	1.62E-05	1.49E-17	TRUE	NA
IL17RD	SeqId_3376_49	Asthma	5	(-0.0093, -5e-04)	0.0283	0.0270	0.0370	2.21E-05	8.17E-58	TRUE	4.51E-02
IL17RD	SeqId_3376_49	COPD	5	(-0.2581, 0.1689)	0.6820	0.9177	0.0370	8.89E-05	5.31E-54	TRUE	8.80E-03
IL1RL1	SeqId_4234_8	COPD	8	(-0.2191, -0.017)	0.0221	0.0628	0.0985	5.19E-05	9.18E-155	TRUE	2.96E-01
IL1RL1	SeqId_4234_8	Asthma	10	(-0.018, 0.0033)	0.1762	0.6837	0.1058	2.31E-04	1.86E-162	TRUE	1.24E-11
KYNU	SeqId_4559_64	Asthma	9	(2e-04, 0.0059)	0.0342	0.4896	0.0353	5.29E-05	1.25E-53	TRUE	2.71E-01
KYNU	SeqId_4559_64	COPD	7	(-0.2354, 0.3164)	0.7737	0.8659	0.0286	4.55E-06	6.55E-45	TRUE	9.91E-01
LILRB2	SeqId_5091_28	Asthma	8	(-0.0038, 0.0028)	0.7500	0.1847	0.0648	5.40E-05	7.21E-101	TRUE	9.55E-03
LILRB2	SeqId_5091_28	COPD	7	(-0.0707, 0.0587)	0.8553	0.2540	0.0517	5.94E-05	1.01E-77	TRUE	1.64E-01
MANSC4	SeqId_9578_263	Asthma	11	(-0.0022, 0.0033)	0.6939	0.3378	0.1017	1.20E-05	3.41E-167	TRUE	8.80E-01
MANSC4	SeqId_9578_263	COPD	10	(-0.0482, 0.1036)	0.4747	0.9966	0.0982	5.76E-05	8.90E-154	TRUE	3.62E-01
MAPK3	SeqId_2855_49	Asthma	12	(-0.0095, -0.0019)	0.0033	0.4445	0.0565	6.04E-05	4.76E-87	TRUE	7.53E-01
MAPK3	SeqId_2855_49	COPD	11	(-0.179, 0.1407)	0.8142	0.5660	0.0455	1.22E-04	5.99E-66	TRUE	4.45E-02
MRC2	SeqId_3041_55	COPD	8	(-0.2325, 0.3342)	0.7250	0.7780	0.1057	2.20E-05	1.87E-169	TRUE	7.44E-01
MRC2	SeqId_3041_55	Asthma	9	(-0.0057, 0.0108)	0.5478	0.8417	0.1085	1.59E-05	1.11E-178	TRUE	6.54E-01
NID2	SeqId_16060_99	COPD	8	(-0.1715, 0.0226)	0.1325	0.2648	0.1766	1.73E-05	3.41E-300	TRUE	9.34E-01
NID2	SeqId_16060_99	Asthma	9	(-0.005, 0.0024)	0.4820	0.9590	0.1797	1.79E-05	0.00E+00	TRUE	7.15E-01
NPNT	SeqId_6342_10	COPD	3	(-0.9589, -0.6434)	0.0000	0.0000	0.0642	6.58E-04	1.79E-84	TRUE	1.57E-06
NPNT	SeqId_6342_10	Asthma	5	(-0.0274, -0.0042)	0.0078	0.0575	0.0716	8.56E-05	1.81E-110	TRUE	4.22E-05

OGN	SeqId_17224_12	Asthma	6	(-0.0047, 0.0052)	0.9200	0.5381	0.1908	1.25E-05	0.00E+00	TRUE	3.89E-01
OGN	SeqId_17224_12	COPD	6	(-0.1906, 0.04)	0.2004	0.8345	0.1908	4.55E-05	0.00E+00	TRUE	5.91E-01
PARK7	SeqId_19523_215	Asthma	10	(-0.0095, -7e-04)	0.0236	0.2457	0.0416	6.93E-05	8.98E-63	TRUE	4.04E-03
PARK7	SeqId_19523_215	COPD	7	(-0.0801, 0.0715)	0.9121	0.4048	0.0288	3.27E-05	2.31E-43	TRUE	4.25E-01
RHOC	SeqId_17764_108	Asthma	2	NA	NA	NA	0.0278	4.01E-06	1.47E-44	TRUE	NA
RHOC	SeqId_17764_108	COPD	2	NA	NA	NA	0.0278	1.20E-05	5.75E-43	TRUE	NA
SCARF2	SeqId_8956_96	COPD	3	(-0.8119, -0.1252)	0.0075	0.0305	0.0515	4.54E-05	4.30E-78	TRUE	9.13E-02
SCARF2	SeqId_8956_96	Asthma	4	(-0.0304, -0.0016)	0.0289	0.0548	0.0564	1.70E-05	2.16E-89	TRUE	1.04E-01
SELM	SeqId_15336_7	Asthma	1	NA	NA	NA	0.0076	2.67E-06	5.15E-13	TRUE	NA
SELM	SeqId_15336_7	COPD	1	NA	NA	NA	0.0076	4.76E-06	1.22E-12	TRUE	NA
SERPING1	SeqId_4479_14	Asthma	13	(-0.0034, 0.0012)	0.3443	0.4729	0.0949	4.09E-05	2.68E-152	TRUE	3.85E-01
SERPING1	SeqId_4479_14	COPD	9	(-0.2391, 0.2244)	0.9505	0.6427	0.0780	5.50E-05	3.46E-120	TRUE	3.34E-01
SFRP1	SeqId_3221_54	COPD	3	(-0.2564, 1.025)	0.2398	0.2158	0.0187	1.53E-05	8.04E-29	TRUE	2.15E-01
SFRP1	SeqId_3221_54	Asthma	5	(-0.011, 0.0195)	0.5850	0.9935	0.0433	1.68E-05	4.11E-68	TRUE	3.65E-01
TMEM2	SeqId_8992_1	Asthma	1	NA	NA	NA	0.0240	2.32E-07	2.13E-39	TRUE	NA
TMEM2	SeqId_8992_1	COPD	1	NA	NA	NA	0.0240	5.14E-06	8.39E-38	TRUE	NA
TRIL	SeqId_6527_1	Asthma	2	NA	NA	NA	0.0234	6.40E-06	1.96E-37	TRUE	NA
TRIL	SeqId_6527_1	COPD	2	NA	NA	NA	0.0234	3.67E-06	5.29E-37	TRUE	NA
XPNPEP1	SeqId_3481_87	Asthma	1	NA	NA	NA	0.0388	4.08E-07	4.58E-63	TRUE	NA
XPNPEP1	SeqId_3481_87	COPD	1	NA	NA	NA	0.0388	2.07E-06	2.34E-61	TRUE	NA

SNPN represents the number of instrumental variants of corresponding proteins. Egger.P is p-value for testing of causal effect from MR-Egger while Intercept.P is p-value for the testing of intercept from MR-egger suggesting of potential horizontal pleiotropy and Steiger.P is p-value in MR-Steiger analysis representing if instrumental variants account for a larger proportion of protein than outcomes. Q.P is the p-value in Q test for heterogeneity. R^2.exposure is the proportion of the protein variance explained by instrumental variants as well as R^2.outcome is the proportion of the outcome variance explained by instrumental variants. Effect Direction represents the consistency of directions of z-scores from PWAS and causal effect estimates in MR analysis of which TRUE means the consistent direction in two analysis.

Table S11 Significant proteins served as potential drug targets

Gene	Match Type	Drug Category	Drugdata Sources
ACAN	Definite	G PROTEIN COUPLED RECEPTOR	HopkinsGroom
	Definite	DRUGGABLE GENOME	HopkinsGroom<br>HingoraniCasas
BOC	Definite	DRUGGABLE GENOME	HingoraniCasas
BTN3A3	Definite	DRUGGABLE GENOME	HingoraniCasas<br>HopkinsGroom
	Definite	B30_2 SPRY DOMAIN	HopkinsGroom
	Definite	EXTERNAL SIDE OF PLASMA MEMBRANE	GO
COL6A3	Definite	DRUGGABLE GENOME	RussLampel<br>HingoraniCasas
	Definite	PROTEASE INHIBITOR	dGene
EFEMP1	Definite	DRUGGABLE GENOME	HingoraniCasas
	Definite	GROWTH FACTOR	GO
FER	Definite	KINASE	HopkinsGroom<br>dGene<br>Pharos
	Definite	DRUGGABLE GENOME	HopkinsGroom<br>HingoraniCasas<br>RussLampel
	Definite	TYROSINE KINASE	GO<br>dGene
FKBP4	Definite	DRUGGABLE GENOME	HingoraniCasas<br>HopkinsGroom

			RussLampel
GM2A	Definite	ENZYME	Pharos
GMPR2	Definite	DRUGGABLE GENOME	HopkinsGroom<br>RussLampel
	Definite	ENZYME	Pharos
GSS	Definite	DRUGGABLE GENOME	HingoraniCasas
HAPLN1	Definite	DRUGGABLE GENOME	HingoraniCasas
IL1RL1	Definite	DRUGGABLE GENOME	HingoraniCasas
	Definite	EXTERNAL SIDE OF PLASMA MEMBRANE	GO
KYNU	Definite	DRUGGABLE GENOME	HingoraniCasas
	Definite	ENZYME	HumanProteinAtlas<br>Pharos
LILRB2	Definite	DRUGGABLE GENOME	HingoraniCasas
	Definite	CELL SURFACE	GO
MAPK3	Definite	DRUGGABLE GENOME	RussLampel<br>HingoraniCasas<br>HopkinsGroom
	Definite	KINASE	HopkinsGroom<br>dGene<br>Pharos
	Definite	SERINE THREONINE KINASE	GO<br>dGene
	Definite	TRANSCRIPTION FACTOR	Pharos
MRC2	Definite	DRUGGABLE GENOME	HingoraniCasas
NID2	Definite	DRUGGABLE GENOME	HingoraniCasas

NPNT	Definite	DRUGGABLE GENOME	HingoraniCasas
OGN	Definite	DRUGGABLE GENOME	HingoraniCasas
	Definite	GROWTH FACTOR	GO
PARK7	Definite	ENZYME	HumanProteinAtlas
	Definite	PROTEASE	GO|dGene
	Definite	HISTONE MODIFICATION	GO
	Definite	DNA REPAIR	GO
SERPING1	Definite	PROTEASE INHIBITOR	HopkinsGroom<br>dGene
	Definite	DRUGGABLE GENOME	HopkinsGroom<br>RussLampel<br>HingoraniCasas
SFRP1	Definite	DRUGGABLE GENOME	HingoraniCasas
	Definite	CELL SURFACE	GO
TMEM2	Definite	TRANSCRIPTION FACTOR	Pharos
XPNPEP1	Definite	DRUGGABLE GENOME	RussLampel<br>HopkinsGroom
	Definite	PROTEASE	HopkinsGroom<br>dGene

Table S12 Significant protein with available drugs

Gene	Match Type	Drug	Interaction Types	Sources	PMID
FKBP4	Definite	SIROLIMUS		NCI	1700475
FER	Definite	HESPERADIN	inhibitor	DTC	19035792
	Definite	ALISERTIB		DTC	
	Definite	SOTRASTAURIN		DTC	
	Definite	CENISERTIB		DTC	
	Definite	LINIFANIB		DTC	
	Definite	GW441756X		DTC	
	Definite	CEDIRANIB		DTC	
	Definite	R-406		DTC	
	Definite	RG-1530		DTC	
	Definite	CYC-116		DTC	
	Definite	ILORASERTIB		DTC	
	Definite	SP-600125		DTC	
	Definite	ENTRECTINIB		DTC	
	Definite	OSI-632		DTC	
DNER	Definite	MIVEBRESIB		TTD	
	Definite	ALOBRESIB		TTD	
	Definite	BIRABRESIB		TTD	
	Definite	BMS-986158		TTD	
	Definite	MOLIBRESIB		TTD	
XPNPEP1	Definite	TOSEDOSTAT	inhibitor	ChemblInteractions	
COL6A3	Definite	OCRIPLASMIN		ChemblInteractions	
	Definite	COLLAGENASE CLOSTRIDIUM HISTOLYTICUM		ChemblInteractions	

GM2A	Definite	BIW-8962		TTD	
MAPK3	Definite	MK-8353	inhibitor	ChemblInteractions	
	Definite	RAVOXERTINIB	inhibitor	ChemblInteractions<br>TTD	25320010
	Definite	ULIXERTINIB	inhibitor	ChemblInteractions<br>TTD	28939558
	Definite	CHEMBL2088730		DTC	22982122
	Definite	WITHAFERIN A		DTC	20726569
	Definite	PRASTERONE		NCI	17102100
	Definite	SPINASAPONIN A METHYL ESTER		DTC	20951582
	Definite	WEDELOLACTONE		DTC	22926226
	Definite	NITROGLYCERIN		NCI	16612556
	Definite	ARISTOLACTONE		DTC	
	Definite	CHEMBL389685		DTC	26492576
	Definite	GAMBOGIC ACID		DTC	17911251
	Definite	MADOLIN T		DTC	
	Definite	QUERCETIN		NCI	17045724
	Definite	XANTHOHUMOL		DTC	22111577
	Definite	CHEMBL2333543		DTC	
	Definite	CHEMBL2333548		DTC	
	Definite	CHEMBL2088731		DTC	22982122
	Definite	ARISTOYUNNOLIN A		DTC	
	Definite	PESUDOGINSENOSIDE RP1 METHYL ESTER		DTC	20951582
	Definite	COMBRETASTATIN A4		DTC	22982122
	Definite	ETOPOSIDE		DTC	23999041

	Definite	VOLVALERENAL D		DTC	
	Definite	GAMBOGIC AMIDE		DTC	17911251
	Definite	CIMIRACEMATE A		DTC	19835377
	Definite	YUANHUADINE		DTC	21916433
	Definite	CHEMBL35482		DTC	
	Definite	OPHIOBOLIN O		DTC	22130129
	Definite	PHENETHYLISOTHIOCYANATE		DTC	22607231
	Definite	ANDROSTANOLONE		NCI	16773205
	Definite	MADOLIN A		DTC	
	Definite	SORAFENIB		PharmGKB	20124951
	Definite	CYCLOPHOSPHAMIDE		NCI	15193230
	Definite	CHEMBL329785		DTC	22982122
	Definite	CHEMBL2063681		DTC	22283430
	Definite	DEMETHYLWEDELOLACTONE		DTC	22926226
	Definite	MADOLIN U		DTC	
	Definite	CHEMBL2063687		DTC	22283430
	Definite	CHEMBL269881		DTC	
	Definite	CHEMBL29197		DTC	17681471
SERPING1	Definite	CINRYZE		TTD	
	Definite	RHUCIN		TTD	

TTD: Therapeutic Target Database, NCI: NCI Cancer Gene Index, DTC: Drug Target Commons, ChemblInteractions: The ChEMBL Bioactivity Database, PharmGKB: Pharmacogenetics and Pharmacogenomics Knowledge Base

Table S13 Detail results of molecular docking

Gene	PDB ID	Drug	PubChem ID	Binding Energy
MAPK3	2ZOQ	ANDROSTANOLONE	10635	-9.2
FER	6KC4	ENTRECTINIB	25141092	-9.0
MAPK3	2ZOQ	CHEMBL35482	4713	-9.0
FER	6KC4	SOTRASTAURIN	10296883	-8.7
MAPK3	2ZOQ	SORAFENIB	216239	-8.7
FER	6KC4	HESPERADIN	135421442	-8.6
FER	6KC4	R-406	11213558	-8.6
MAPK3	2ZOQ	DEMETHYLWEDELOLACTONE	5489605	-8.6
MAPK3	2ZOQ	MK-8353	58282870	-8.5
MAPK3	2ZOQ	YUANHUADINE	6440572	-8.3
FER	6KC4	CENISERTIB	11569967	-8.2
MAPK3	2ZOQ	ETOPOSIDE	36462	-8.2
FER	6KC4	CEDIRANIB	9933475	-8.0
FER	6KC4	RG-1530	135398512	-7.9
MAPK3	2ZOQ	QUERCETIN	5280343	-7.9
MAPK3	2ZOQ	WEDELOLACTONE	5281813	-7.9
MAPK3	2ZOQ	CHEMBL2088730	60143342	-7.8
MAPK3	2ZOQ	XANTHOHUMOL	639665	-7.8
FER	6KC4	ILORASERTIB	46207586	-7.7

MAPK3	2ZOQ	ARISTOLACTONE	5315198	-7.5
MAPK3	2ZOQ	CHEMBL2063681	56963903	-7.5
FER	6KC4	LINIFANIB	11485656	-7.3
XPNPEP1	3CTZ	TOSEDOSTAT	15547703	-7.3
MAPK3	2ZOQ	CHEMBL2088731	60148069	-7.2
FER	6KC4	CYC-116	6420138	-7.1
MAPK3	2ZOQ	PRASTERONE	5881	-7.1
FER	6KC4	GW441756X	16219401	-7.0
FER	6KC4	SP-600125	8515	-6.9
FER	6KC4	ALISERTIB	24771867	-6.8
MAPK3	2ZOQ	CHEMBL2333543	71665736	-6.8
MAPK3	2ZOQ	RAVOXERTINIB	71727581	-6.8
MAPK3	2ZOQ	ULIXERTINIB	11719003	-6.8
MAPK3	2ZOQ	CHEMBL269881	4564	-6.7
MAPK3	2ZOQ	CHEMBL29197	4705	-6.4
DNER	AF-Q8NFT8-F1-v4	BIRABRESIB	9936746	-6.1
DNER	AF-Q8NFT8-F1-v4	ALOBRESIB	86281210	-6.0
MAPK3	2ZOQ	CHEMBL2063687	70684410	-6.0
DNER	AF-Q8NFT8-F1-v4	BMS-986158	118196485	-5.9

MAPK3	2ZOQ	CHEMBL2333548	13818876	-5.9
MAPK3	2ZOQ	CHEMBL329785	8706	-5.9
DNER	AF-Q8NFT8-F1-v4	MOLIBRESIB	46943432	-5.6
DNER	AF-Q8NFT8-F1-v4	MIVEBRESIB	71600087	-5.5
MAPK3	2ZOQ	NITROGLYCERIN	4510	-5.4
MAPK3	2ZOQ	CHEMBL389685	9222	-4.9
MAPK3	2ZOQ	PESUDOGINSENOSIDE RP1 METHYL ESTER	52943314	-4.7
MAPK3	2ZOQ	CYCLOPHOSPHAMIDE	2907	-4.5

PDBID: Proteins ID obtained from Protein Data Bank PubChem ID: Drug ID obtained from PubChem Binding Energy:The binding affinity between protein and drug compounds quantified by calculating the binding free energy (Δ G, kcal/mol) using AutoDock Vina
